# WIP1 Contributes to the Adaptation of Fanconi Anemia Cells to DNA Damage as Determined by the Regulatory Network of the Fanconi Anemia and Checkpoint Recovery Pathways

**DOI:** 10.3389/fgene.2019.00411

**Published:** 2019-05-03

**Authors:** Alfredo Rodríguez, J. Jesús Naveja, Leda Torres, Benilde García de Teresa, Ulises Juárez-Figueroa, Cecilia Ayala-Zambrano, Eugenio Azpeitia, Luis Mendoza, Sara Frías

**Affiliations:** ^1^Laboratorio de Citogenética, Departamento de Investigación en Genética Humana, Instituto Nacional de Pediatría, Mexico City, Mexico; ^2^PECEM, Facultad de Medicina, Universidad Nacional Autónoma de México, Mexico City, Mexico; ^3^Instituto de Investigaciones Biomédicas, Universidad Nacional Autónoma de México, Mexico City, Mexico; ^4^Department of Evolutionary Biology and Environmental Studies, University of Zurich, Zurich, Switzerland

**Keywords:** Fanconi anemia, ICLs, FA/BRCA pathway, CHKREC, WIP1, DNA damage adaptation

## Abstract

DNA damage adaptation (DDA) allows the division of cells with unrepaired DNA damage. DNA repair deficient cells might take advantage of DDA to survive. The Fanconi anemia (FA) pathway repairs DNA interstrand crosslinks (ICLs), and deficiencies in this pathway cause a fraction of breast and ovarian cancers as well as FA, a chromosome instability syndrome characterized by bone marrow failure and cancer predisposition. FA cells are hypersensitive to ICLs; however, DDA might promote their survival. We present the FA-CHKREC Boolean Network Model, which explores how FA cells might use DDA. The model integrates the FA pathway with the G2 checkpoint and the checkpoint recovery (CHKREC) processes. The G2 checkpoint mediates cell-cycle arrest (CCA) and the CHKREC activates cell-cycle progression (CCP) after resolution of DNA damage. Analysis of the FA-CHKREC network indicates that CHKREC drives DDA in FA cells, ignoring the presence of unrepaired DNA damage and allowing their division. Experimental inhibition of WIP1, a CHKREC component, in FA lymphoblast and cancer cell lines prevented division of FA cells, in agreement with the prediction of the model.

## 1. Introduction

Interstrand crosslinks (ICLs) are severe DNA lesions that interfere with essential cellular processes, such as DNA replication and gene transcription. ICLs activate the Fanconi anemia (FA) pathway (also known as the FA/BRCA pathway) and the cell cycle checkpoint, which induces cell cycle arrest (CCA) providing time for the restoration of DNA integrity (Giglia-Mari et al., 2011). While the FA pathway combines homologous recombination repair (HRR), translesion synthesis (TLS) and nucleotide excision repair (NER) for restoration of DNA integrity (Ceccaldi et al., 2016b), the checkpoint activation during G2 avoids the inheritance of unrepaired DNA damage (Zhou and Elledge, 2000; Lobrich and Jeggo, 2007).

Defects in the FA pathway are associated with several human diseases. Germline inactivation of any of the FA pathway genes causes FA, a chromosome instability syndrome with congenital malformations, early-onset bone marrow failure and cancer predisposition (Auerbach, 2009). Somatic inactivation of the FA pathway has been identified in breast, ovarian, and pancreatic tumors (Ceccaldi et al., 2016b). Heterozygous females for certain FA pathway mutations, such as *BRCA1* and *BRCA2*, have an increased risk of breast and ovarian cancer (Tung, 2015; Nielsen et al., 2016).

The DNA damage checkpoint is coordinated during G2 by the Ataxia Telangiectasia Mutated (ATM) and the Ataxia Telangiectasia and Rad3-Related Protein (ATR) kinases (Andreassen et al., 2004), as well as their downstream effectors p53 and p21 (Macleod et al., 1995). p21 directly blocks the mitosis promoting factor, composed by Cyclin B and CDK1 (Cyclin B/CDK1 complex), also known as MPF (mitosis promoting factor), thus avoiding cell division (Charrier-Savournin et al., 2004). When DNA damage has been fixed, the checkpoint recovery (CHKREC) inactivates the G2 checkpoint and promotes mitosis entry (van Vugt et al., 2001; Tsvetkov and Stern, 2005; Medema and Macurek, 2011). CHKREC components include the Wild-Type P53-Induced Phosphatase 1 (WIP1) and the Cell Division Cycle 25 (CDC25) family of phosphatases that, through dephosphorylation, inhibit the checkpoint mediators (Ray and Kiyokawa, 2008; Lindqvist et al., 2009a; Shaltiel et al., 2014); and the kinases Cyclin Dependent Kinase 1 (CDK1), Aurora A kinase, Polo Like Kinase 1 (PLK1) and Microtubule Associated Serine/Threonine Kinase Like (MAST-L) (Mamely et al., 2006; Seki et al., 2008; Lindqvist et al., 2009b; Peng, 2013).

Interestingly, during ongoing CCA, basal CHKREC activity remains, possibly to maintain the cell poised for an eventual cell-cycle reentry after DNA repair. This basal CHKREC activity does not include the activation of Cyclin B/CDK1 complex, which starts an irreversible entry into mitosis (Cha et al., 2010; Liang et al., 2014). The balance between the checkpoint and the CHKREC is critical to avoid the inheritance of unrepaired DNA damage; however, DNA damage adaptation (DDA)–a mechanism that allows cell division with unrepaired DNA damage–might occur. DDA is mediated by some CHKREC effectors, which are considered oncogenes (Gritsko et al., 2003; Yamada et al., 2004; Belova et al., 2005; Ray and Kiyokawa, 2008).

Several tumors are deficient in the FA pathway, including high-grade ovarian carcinomas, triple negative breast cancers and metastatic prostate cancer (Ceccaldi, 2015), which can be compensated by activation of alternative DNA repair pathways, such as DNA polymerase -PARP1-mediated alternative-end joining (Ceccaldi, 2015). However, FA pathways deficient cells might also enhance DDA as a mechanism that allows cell survival, a role that has not been explored.

Given that the components of the checkpoint and the CHKREC are well-known (Lu et al., 2005; Lindqvist et al., 2009b; Peng, 2013), and there is vast knowledge about the FA pathway, a computational model of the regulatory interactions among these processes should be possible. Boolean network models (BNMs), the simplest form of discrete dynamical systems, have shown to be straightforward, robust, comprehensive, and integrative tools for studying the dynamical behavior of several cellular processes. Despite their apparent simplicity, these models qualitatively describe experimental published data (Faure et al., 2006; Mendoza, 2006; Zhang et al., 2010; Rios et al., 2015).

Relevant computational models have studied how p53 decides cell fate upon DNA damage induction (Ciliberto et al., 2014) and how the G2 checkpoint is controlled (Kesseler et al., 2013). Furthermore, our group has previously published BNMs on the functioning of the FA pathway (Rodriguez et al., 2012, 2015). We hereby present the FA-CHKREC BNM, a model that focuses on DDA adaptation in FA pathway deficient cells. This BNM integrates the connectivity among the FA pathway, the checkpoint and the CHKREC, and was used to explore how FA cells survive to DNA damage by simulating several combinations of checkpoint and CHKREC mutants so as to propose the necessary conditions for DDA in FA cells.

We found that FA pathway deficient cells might promote DDA through several mechanisms, including (1) promotion of an alternative ICL unhooking pathway that enables replication fork collapse and generation of double strand breaks (DSBs), and (2) over-dependence on the CHKREC, which might inactivate the DNA damage repair and checkpoint proteins, thus ignoring the presence of unrepaired DSBs and promoting cell division. To verify the over-dependence that FA cells might have on the CHKREC we chemically inhibited several of its components, including WIP1, PLK1, Aurora Kinase A, and CDC25. WIP1 is of particular relevance since it is a CHKREC phosphatase that dephosphorylates ATM, ATR and γH2AX (Cha et al., 2010) and notably is emerging as a potential target in oncology. In accordance with our BNM simulations, inhibition of WIP1 in FA cells avoided cell division, thus proving that the CHKREC is critical for DDA in FA pathway deficient cells.

## 2. Methods

**Figure 1:**
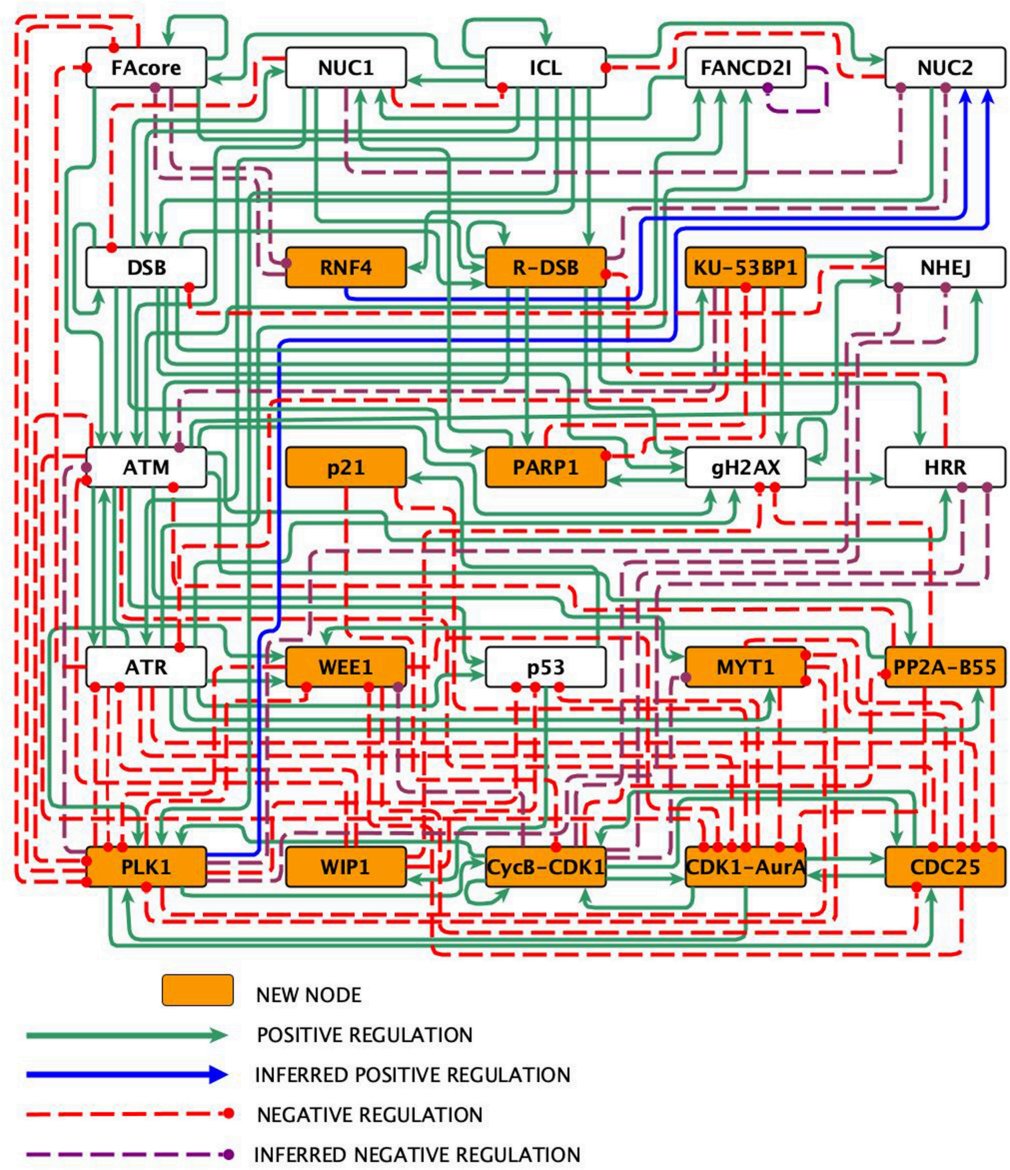
The FA-CHKREC network. Nodes represent proteins or protein complexes, solid pointed arrows are positive regulatory interactions, whereas dashed blunt arrows are negative regulatory interactions.

Experimental data from the literature on the FA pathway, the checkpoint, and the CHKREC were thoroughly analyzed to construct the FA-CHKREC network (Figure 1). The network incorporates the mechanisms that are activated when an ICL is detected by the FA pathway, and how the checkpoint and CHKREC respond to this damage. This network consists of 25 nodes and 123 regulatory interactions, 80 of them positive and 42 negative (Figure 1 and Table 1). Not all the interactions in the FA-CHKREC network have been previously reported. Indeed, 15 of these interactions were included during the modeling process so as to reproduce the current experimental data. This was the minimal set of new interactions that allowed the network to recover a dynamical behavior in agreement with current knowledge. The function of these 15 interactions can be ascribed to three relevant processes: (1) Fork replication collapse and generation of DSBs by pathways alternative to the FA/BRCA pathway, (2) Inactivation of checkpoint proteins, and (3) Inactivation of DNA repair pathways. Proteins and complexes included per node, as well as their function in the network are summarized in Table S1, whereas the most important modifications with respect to former FA pathway BNMs (Rodriguez et al., 2012, 2015) are summarized in Table S2.

**Table 1:** Boolean functions for the nodes in the FA-CHKREC network.

ICL ← ICL ∧¬ (NUC1 ∨ NUC2)
FAcore ← ICL ∧¬ ((RNF4 ∧ PLK1) ∨ (FAcore ∧¬ ATR))
FANCD2I ← FAcore ∧ (ATM ∨ ATR) ∧¬ FANCD2I
NUC1 ← (ICL ∧ FANCD2I) ∨ (DSB ∧ PARP-1)
RNF4 ← ICL ∧¬ FAcore
NUC2 ← ICL ∧ RNF4 ∧ PLK1 ∧¬ (R-DSB ∨ NUC1)
DSB ← (DSB ∨ ICL ∧ NUC2) ∧¬ (NHEJ ∨ NUC1)
PARP-1 ← (DSB ∨ R-DSB) ∧ gH2AX ∧¬ (KU-53BP1)
R-DSB ← (R-DSB ∨ ((ICL ∨ DSB) ∧ NUC1)) ∧¬ (HRR)
HRR ← gH2AX ∧ R-DSB ∧ ATM ∧¬ (PLK1 ∧ CycB-CDK1)
KU-53BP1 ← DSB ∧¬ PARP-1
NHEJ ← KU-53BP1 ∧ DSB ∧ ATM ∧¬ (PLK1 ∧ CycB-CDK1)
gH2AX ← (DSB ∨ R-DSB) ∧ (ATM ∨ ATR ∨ gH2AX ∨ KU-53BP1)
∧¬ (WIP1 ∧ PP2A-B55)
ATR ← (ICL ∨ ATM) ∧¬ (WIP1 ∨ (PLK1 ∧ KU-53BP1))
ATM ← (ATR ∨ DSB ∨ R-DSB ∨ NUC1 ∨ FAcore)
∧¬ (WIP1 ∨ PP2A-B55 ∨ (PLK1 ∧ KU-53BP1))
MYT1 ← (ATM ∨ ATR) ∧¬ (CDC25 ∨ CycB-CDK1 ∨ PLK1)
WEE1 ← (ATM ∨ ATR ∨ PP2A-B55) ∧¬ (CDC25 ∨ CycB-CDK1 ∨ PLK1)
p53 ← (ATM ∨ ATR) ∧¬ (WIP1 ∧ (PLK1 ∨ CDK1-AurA))
p21 ← p53
PP2A-B55 ← (ATM ∨ ATR) ∧¬ CycB-CDK1
WIP1 ← p53
CDK1-AurA ← CycB-CDK1 ∨ CDC25 ∨¬ (p21 ∧ PP2A-B55)
∧¬ (WEE1 ∨ MYT1 ∨ ATM ∨ ATR)
PLK1 ← CycB-CDK1 ∨ (ICL ∧ ATR ∧¬ FAcore) ∨ ((CDK1-AurA)
∧¬ (MYT1 ∨ WEE1 ∨ ATR ∨ ATM))
CDC25 ← CycB-CDK1 ∨ (PLK1 ∧ (CycB-CDK1 ∨ CDK1-AurA)
∧¬ ((WEE1 ∨ MYT1) ∧ (PP2A-B55 ∨ ATM ∨ ATR)))
CycB-CDK1 ← CycB-CDK1 ∨ (CDC25 ∧ Plk1 ∧ CDK1-AurA) ∧¬ p21

### 2.1. Reconstruction of the FA-CHKREC Network

DNA double strand breaks (DSBs) can be generated by both endogenous and exogenous sources of damage. DSBs can be induced directly (DSB node) by, for example, ionizing radiation or can be originated as a byproduct during the repair of another types of DNA damage, such as an interstrand crosslink (ICL) (Knipscheer et al., 2009; Ceccaldi et al., 2016b). The current evidence indicates that the non-homologous end-joining (NHEJ) pathway repairs most direct-DSBs, irrespective of the cell cycle phase in which they appear; whereas the homologous recombination repair (HRR) pathway repairs ICL-derived DSBs, the repair of these lesions requires end-resection (R-DSB node) mediated by DNA exonucleases (Ceccaldi et al., 2016a).

When a DSB appears in the DNA molecule it is recognized first by the KU70-KU80 heterodimer and the 53BP1 protein (KU-53BP1 node). KU70/80 ensures the DNA ends and 53BP1 represses the function of HRR proteins, such as FANCS/BRCA1, thus funneling the repair of the DSB to the NHEJ pathway, composed by DNA-PKcs kinase, the Artemis nuclease and the XLF-Lig4 complex (NHEJ node), that directly seal the DSB and restore the DNA continuity. Although the NHEJ is considered highly mutagenic, it is the main DSB repair pathway along the cell cycle given its promptness (Lieber, 2010; Ceccaldi et al., 2016a).

An R-DSB is generated during the repair of an ICL. Briefly, an ICL encountered during S-phase stalls the DNA replication fork and activates the FA/BRCA pathway through the FANCM-FAAP24 complex (Xue et al., 2008; Schwab et al., 2010; Blackford et al., 2012). The FANCM-FAAP24 complex recruits the FA core complex (FAcore node) (Kottemann and Smogorzewska, 2013) which, through its E3 ubiquitin ligase, activates the FANCD2-FANCI heterodimer (FANCD2-I) (FANCD2I node) (Meetei et al., 2004). The monoubiquitinated FANCD2-I complex remains in the chromatin and promotes the recruitment of FANCP/SLX4, a scaffold for the nucleases associated to the FA/BRCA pathway: MUS81, FANCQ/XPF, and SLX1 (Kottemann and Smogorzewska, 2013) (NUC1 node). These nucleases unhook the ICL and generate a DNA adduct in one of the previously joined strands, and a DSB in the opposing strand that will be resected by DNA exonucleases to generate a R-DSB (Ceccaldi et al., 2016b). The adduct is bypassed by translesion synthesis polymerases and repaired by nucleotide excision repair, whereas the R-DSB is funneled to the HRR pathway (HRR node) (Rodriguez et al., 2012).

Both DSBs and R-DSBs activate the phosphorylation of the histone variant H2AX at S139, producing γH2AX (γH2AX node), the main histone modification associated to DSBs signaling that also functions as a scaffold for the recruitment of additional DNA repair factors (Celeste et al., 2003; Kolas et al., 2007). As mentioned, the R-DSB was generated by DNA exonucleases to generate a single-stranded 3'DNA overhang (ssDNA) that is rapidly covered by RPA subunits (Cejka, 2015; Ceccaldi et al., 2016b). RPA is subsequently substituted by RAD51 that in association with the recombination proteins FANCD1/BRCA2 and other RAD51 family members would compose the pre-synaptic filament that searches homology for DNA repair (Taylor et al., 2015; Ceccaldi et al., 2016a,b; Godin et al., 2016). This strand invasion will mediate HRR and is considered the process with the highest fidelity for DSB repair (Jasin and Rothstein, 2013). This entire process is included in the HRR node. As previously mentioned, the NHEJ is the main DSB repair pathway (Wu et al., 2008), however the proteins of the FA/BRCA pathway block the activity of the NHEJ proteins during ICL processing thus ensuring that the HRR will be the only mechanism repairing R-DSBs (Bunting and Nussenzweig, 2010; Pace et al., 2010).

Importantly, error-prone alternative pathways for the processing of ICLs and the repair of ICL-derived DSBs might be activated when the FA/BRCA pathway is non-functional. We included these possibilities in the FA-CHKREC BNM. First, as the generation of ICL-derived DSBs in FA cells is not completely abolished, we hypothesized that an alternative ICL-unhooking pathway might emerge to improve the tolerance of these cells to ICL-arrested replication forks. In ATR mutant cells, for example, the constant replication stress due to defective DNA damage response (DDR) activates PLK1 and RNF4 (RNF4 node) that cooperatively take the control of the stalled replication fork and coordinate the recruitment of alternative DNA nucleases, thus collapsing the replication fork for continuation of DNA synthesis (Ragland et al., 2013). Although this mechanism has not been proven in FA cells, we hypothesize that the replication stress due to unrepaired ICLs could activate the same or a similar cascade of events for collapsing the replication forks in absence of the FA pathway. The NHEJ, on the other hand, is well-known to be over-activated in FA cells as a backup DNA repair pathway that might collaborate in the repair of the DSBs accumulated in FA cells (Adamo et al., 2010; Bunting and Nussenzweig, 2010; Pace et al., 2010). Finally, when inhibited, the alternative-NHEJ initiated by PARP-1, displays synthetic lethality in FA mutants and PARP-1 has been shown to have an important role protecting the HRR process from NHEJ interference (Hochegger et al., 2006).

Whilst DNA damage is present, it is critical to avoid cell cycle progression from G2 to M, the last checkpoint before cell division (Giglia-Mari et al., 2011). As the G2/M progression is promoted by the mitosis promoting factor, also known as Cyclin B/CDK1 complex, (Cyclin B-CDK1 node), a tight regulation over this complex is necessary, above all when the cell cycle has been perturbed by DNA damage (Charrier-Savournin et al., 2004).

Cyclin B-CDK1 blockage is accomplished through several mechanisms, in the presence of a DSB, cell cycle arrest is initiated through the ATM kinase (ATM node), while in presence of an ICL, the ATR kinase through HCLK2 and RPA accumulation (ATR node) takes charge on signaling (Yazinski and Zou, 2016). These kinases spread the DNA damage signal through their downstream effectors CHK2, CHK1 and p53 (p53 node) (Collis, 2008; Schwab et al., 2010). In addition, ATM and ATR phosphorylate additional components of the FA/BRCA pathway (Traven and Heierhorst, 2005; Matsuoka et al., 2007). Once triggered, p53 activates the expression of p21, the main cell cycle blocker that inhibits the kinase activity of the Cyclin B/CDK1 complex and avoids cell cycle progression while the DNA repair transactions are performed (Charrier-Savournin et al., 2004).

Another branch for cell cycle blockage is composed by the WEE1 (WEE1 node) and MYT1 (MYT1 node) kinases that directly inhibit the Cyclin B/CDK1 complex formation by phosphorylating the Thr14 and Tyr15 residues of CDK1 (Wang et al., 2004). Removal of these inhibitory modifications is controlled by members of the CDC25 phosphatases and leads to a rapid activation of the Cyclin B/CDK1 complex. In turn, the initial activation of the Cyclin B/CDK1 complex stimulates the activity of CDC25 and inactivates WEE1, creating a feedback that increases the Cyclin B/CDK1 complex activity. Activation of the DNA damage checkpoint in G2 leads to a reduction in the activity of the CDC25 family and increases WEE1 function thus keeping the Cyclin B/CDK1 complex limited and preventing mitotic entry (Takizawa and Morgan, 2000; Mailand et al., 2002; Ray and Kiyokawa, 2008).

When the DNA repair process has been completed, the cell needs to switch off the checkpoint, which in turn would allow the division of the cell (Bartek and Lukas, 2007; Medema and Macurek, 2011). To accomplish this, several phosphatases and kinases of the checkpoint recovery (CHKREC) take charge and inactivate the checkpoint components as well as activate the promoters of cell division, such as the Cyclin B/CDK1 complex (Shaltiel et al., 2014). The control of this switch is of central importance given that failing its execution might conduct to the delivery of unrepaired DNA. One of the critical steps in this process is the inactivation of p53 by the WIP1 phosphatase (WIP1 node) and the MDM2 ubiquitin ligase (Bose and Ghosh, 2007), which themselves are direct transcriptional targets of p53. WIP1 dephosphorylates p53 at S15 and prevents its activity (Belova et al., 2005).

WIP1 dephosphorylates γH2AX, ATM, CHK2, and CHK1 thus inactivating the checkpoint (Cha et al., 2010). Other phosphatases have been shown to collaborate with WIP1 to dismantle the checkpoint including PP2A-B55, PP4, and PP6 (PP2A-B55 node) (Schmitz, 2010). Importantly, WIP1 deficiency results in a failure in checkpoint recovery and the cell remains arrested in G2 (Belova et al., 2005). Aside from the reversal of phosphorylations by phosphatases, the CHKREC is also regulated by several kinases (Peng, 2013), including PLK1 (PLK1 node) which is of the highest importance (Seki et al., 2008). PLK1 phosphorylates several DNA repair and checkpoint components in such a way that the targeted proteins are recognized by βTrCP- ubiquitin proteosome system and degraded (Mamely et al., 2006). Well-recognized PLK1 targets include WEE1, Claspin, 53BP1, CHK2, and p53 (van Vugt et al., 2010). During CHKREC, PLK1 becomes essential for re-entry into the cell cycle as well as its upstream activators Aurora A and Bora (CDK1-AurA node) (Seki et al., 2008). Finally, the Cyclin B/CDK1 complex is reactivated by the CDC25 phosphatase family (CDC25 node) (Boutros et al., 2006). Given the pro-mitotic activity of Cyclin B, PLK1 and Bora, just to name a few, they are actively targeted for degradation or remain down-regulated whereas the cell cycle arrest is active (He et al., 2005; Tsvetkov and Stern, 2005).

In conclusion, cell cycle arrest and DNA repair are two mechanisms that act in a concerted way in presence of DNA damage, the proteins that mediate both processes are inactivated by the CHKREC once the DNA damage has been eliminated. The CHKREC consists of two concerted mechanisms: (a) the phosphatase negative pathway that inactivates DDR and its effectors and (b) the kinase cascade that mediates cell cycle progression (Shaltiel et al., 2014).

### 2.2. The Discrete Dynamical System

We modeled the FA-CHKREC network as a Boolean network using the nodes and logical rules that appear in Table 1. The implementation of the network as a dynamical system followed standard procedures (Rodriguez et al., 2012, 2015; Ramírez and Mendoza, 2018), and the simulations were performed using BoolNet (Müssel et al., 2010) under a synchronous updating scheme. We simulated the wild type network as well as all possible gain-of-function mutants and null mutants of the model. The null and constitutive activation mutants were simulated by fixing the mutant node at 0 or 1, respectively.

Two biologically relevant conditions were simulated: namely, a short exposure to DNA damage (represented by the node ICL), which is assumed to be repaired fast and efficiently, and a persistent exposure to DNA damage, which results in a saturation of the DNA repair pathways. The response to short DNA damage exposure was simulated synchronously both in the *wild type* and the mutants by setting the ICL activation state to 1 only at the initial state, whereas a continuous exposure to DNA damage was simulated by fixing the DNA damage node activation state to 1. The effect of removing interactions was also evaluated when considered pertinent in combination with null/persistent activation mutants and in response to short/persistent exposures to DNA damage. The trajectories from all possible initial states were analyzed until the system reached an attractor. The model is available as the Supplementary files *FA-CHKREC_for_BoolNet.txt* and *FA-CHKREC.smbl*.

### 2.3. Cell Culture and Survival Curves

The EUFA316+EV, EUFA316+G, VU817 (kindly donated by Dr. Hans Joenje, VU University Medical Center) and NL53 (DNA repair proficient) lymphoblast cell lines were maintained in RPMI 1640 medium supplemented with 10% fetal calf serum (GIBCO, Waltham, Massachusetts, USA). Cancer derived cell lines TOV21G+EV and TOV21G+F, as well as MCF7 cell lines were maintained in RPMI medium 1640 supplemented with 10% fetal calf serum (GIBCO, Waltham, Massachusetts, USA); HeLa cells were maintained in DMEM medium supplemented with supplemented with 10% fetal calf serum (GIBCO, Waltham, Massachusetts, USA) For survival curves, cells were seeded in 96-well plates at 2,000, or 5,000 cells per well, treated with serial dilutions of GSK2830371, BI2536, TC-S 7010, CDC25 phosphatase inhibitor II, Carboplatin, Olaparib, and/or MMC. Cell survival was analyzed after 5 days of culture using Cell Titer Glo (Promega) following manufacturer instructions.

### 2.4. Chromosome Aberration Analysis, Cell Cycle Analysis, Mitotic Index, Apoptosis, and Gene Expression Analysis

Cells were treated with MMC and CCT007093 (WIP1 inhibitor) (both from Sigma-Aldrich Co, St. Louis MO, USA) during 96 h and harvested every 24 h. For chromosome aberrations analysis, colchicine (Sigma-Aldrich Co, St. Louis MO, USA) (final concentration 0.1 μg/ml) was added to cell cultures 1 h before harvesting. Twenty-five metaphases per experimental condition were scored and the number of chromatid breaks, chromosome breaks and radial figures was assessed. For cell cycle and DNA damage analysis, cells were fixed with the Cytofix/Cytoperm buffer (BD Biosciences), washed with the Perm-Wash buffer (BD Biosciences) and stained with anti- H3S10ph antibody (Biolegend) and anti-γH2AX antibody (BD Biosciences). For DNA content analysis cells were counterstained with Propidium Iodide (Sigma-Aldrich). A total of 20,000 events were acquired in a BD FACSCalibur or a FACScan cytometer (BD Biosciences) and analyzed in the FlowJo software version 10.1. For gene expression analysis RNA was extracted with the QIAGEN mini kit, PCR arrays were performed following the manufacturers instructions, using the RT 2 Profiler™PCR Array “Human DNA Repair” kit (QIAGEN). PCR reaction was performed in an ABI Prism 7000 Real-Time PCR System (Applied Biosystem) and data were analyzed using the QIAGEN on-line tool (https://www.qiagen.com/us/shop/genes-and-pathways/data-analysis-center-overview-page/) Experiments were run by triplicate.

## 3. Results and Discussion

The reconstructed FA-CHKREC regulatory network (Figure 1) and its associated logical functions (Table 1), synthesize the experimental knowledge with regard to the FA pathway and its interactions with the checkpoint and CHKREC. The FA-CHKREC network includes 25 nodes and 122 regulatory interactions (68 positive and 55 negative). We incorporated to the model 13 nodes, mainly CHKREC nodes, not previously considered to be relevant for FA pathway deficient cells, and 15 inferred interactions, which have not been yet reported but that are necessary for the model to recover the observed dynamical behavior. Novel nodes and interactions are indicated in Figure 1. The incorporation of these nodes and interactions constitutes veritable predictions that can experimentally be validated and will be described below.

### 3.1. Dynamical Properties of the Network

The attractors obtained with the FA-CHKREC model in every DNA damage simulation were considered to be a cell fate decision. These attractors were classified, according to the activation of critical nodes, in two categories: (1) cell-cycle arrest (CCA) attractor and (2) cell-cycle progression (CCP) attractor. The attractors obtained in every mutant simulation were not necessarily identical. However, a “signature” for every attractor category can be defined based on the activity of critical nodes. The CCP attractor is defined by activation of the cell division promoter nodes (CDK1-AurA, PLK1, CDC25, and Cyclin B-CDK1) and inactivation of any other node in the network. The CCA attractor is defined by the activity of the DNA damage nodes (ICL, DSB, and R-DSB), activity of the cell cycle arrest nodes (ATM, ATR, p53, p21, WEE1, and MYT1) and inactivation of the critical node CyclinB-CDK1. It is important to mention that deviations from these two broad attractor categories exist. For example, CCP-DDA (Cell Cycle Progression with DNA Damage Adaptation Attractor), the one that is critical for this work. This attractor shows activation of cell division nodes along with activation of DNA damage nodes (see also Table 2).

**Table 2:** Biological meaning of the attractors obtained in the FA-CHKREC network.

**Attractor**	**Biological meaning**	**Activation state of the nodes in the attractor**	**Variants**
Cell cycle progression (CCP)	CCP occurs when the Cyclin B-CDK1 complex is activated. In normal conditions this only occurs after completion of DNA damage repair. However, under circumstances of excessive DNA damage or DNA repair null mutants, CCP can occur with unrepaired DNA damage.	Nodes ON: CDK1-AurA, PLK1, CDC25 an CyclinB-CDK1. Nodes OFF: the remaining.	Any DNA damage node (ICL, RDSB) can be activated along with the Cyclin B-CDK1 node in the same attractor. This condition in biological terms reflects CCP with DNA damage adaptation (DDA).
Cell Cycle Arrest (CCA)	CCA occurs in the G2-M transition when DNA damage has not been repaired. CCA is dependent on the proteins of the checkpoint and can be triggered by different types of unrepaired DNA damage. During an ongoing CCA, activation of DNA repair nodes and CHKREC nodes can occur, however the system will remain in CCA as long as the Cyclin B-CDK1 node is not activated.	Nodes ON: PIKK-CHK, P53, P21, and any DNA damage node (ICL, RDSB, DSB). Nodes OFF: Cyclin B-CDK1.	In addition to the nodes previously listed, other nodes can be activated however, the Cyclin B-CDK1 node is never activated in this type of attractor.

#### 3.1.1. The FA-CHKREC BNM Recapitulates the Wild Type Behavior in Response to ICLs

**Figure 2:**
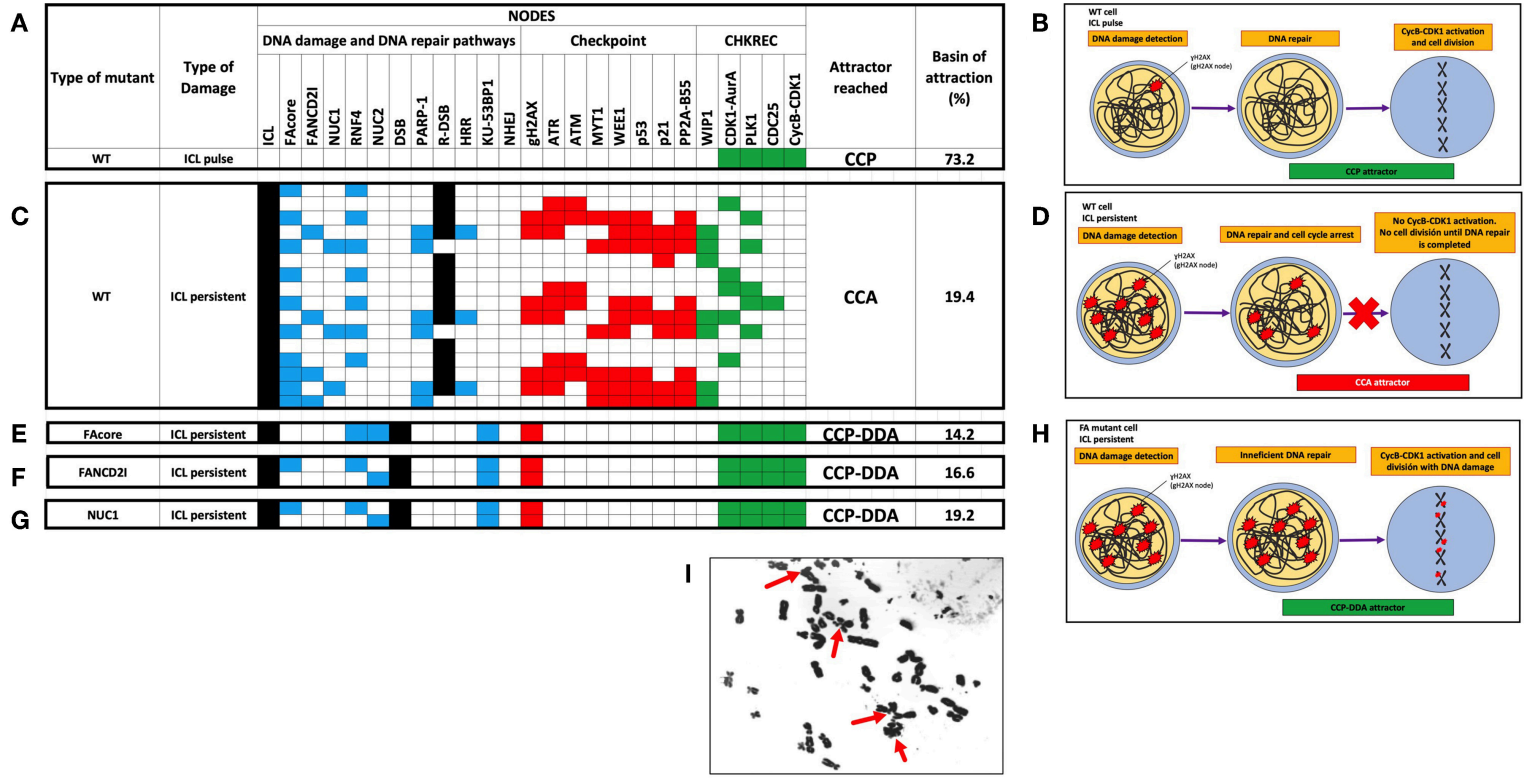
Selected attractors showing that the FA-CHKREC BNM recapitulates the current knowledge on the FA/BRCA pathway and the response of cell cycle checkpoints to DNA damage. **(A)** In the WT network detection and repair of an ICL pulse by the FA/BRCA pathway allows activation of the CHKREC nodes, reaching a fixed-point CCP attractor. Cell division strictly requires CycB-CDK1 node activation. **(B)** Schematics of the DNA damage detection and repair cellular processes activated upon DNA damage induction. WT cells efficiently detect and repair small pulses of DNA damage and reach the CCP attractor, which implies cell division. **(C)** In presence of a persistent ICL the WT network remains in a cyclic CCA attractor, is not able to activate the CycB-CDK1 node, until the source of damage is removed. **(D)** Schematics showing that WT cells will remain arrested in a CCA attractor, implying a blocking in cell division, until the DNA damage source is removed and DNA damage is fully repaired. **(E-G)** Representative FA mutants activate the CHKREC full process despite persistent DNA damage (ICLs and DSBs), arriving to a fixed-point attractor, that we denominate the CCP-DDA (Cell Cycle Progression with DNA Damage Adaptation) attractor. **(E)** FAcore mutant, **(F)** FANCD2I mutant, **(G)** NUC1 mutant. **(H)** Schematics showing that despite unrepaired DNA damage FA cells reach cell division (CCP-DDA attractor). **(I)** Representative metaphase from the FA cell line VU817 (*FANCA* mutant, FAcore mutant) showing unrepaired DNA damage in the form of chromosome breakage that reached cell division (red arrows). Only attractors are shown. Nodes in the simulations are grouped by color, according to functional categories: DNA damage in black, DNA repair pathways in blue, Checkpoint in red and CHKREC in green. Inactive nodes are colorless, whereas active nodes are colored according to their functional category. Refer to Supplementary Material S1 to see the whole trajectories to attractors of these and other mutants.

We simulated the FA-CHKREC BNM using all possible initial conditions under synchronous conditions and observed that the model can reach 8 attractors spanning two main categories CCP and CCP-DDA attractors (Figure S1). Then we simulated the response of the WT FA-CHKREC network facing DNA damage. In this simulation we observe that the FA-CHKREC BNM recapitulates the expected behavior of a wild-type cell: a single pulse of ICL leads to activation of the FA pathway and the checkpoint (Zhou and Elledge, 2000; Lobrich and Jeggo, 2007). When the DNA damage is fixed, the CHKREC turns-off the checkpoint, and the system proceeds into a CCP attractor (Figures 2A,B). On the other hand, persistent ICL in the WT network conducts the system toward a CCA attractor, characterized by recurrent activation of the FA pathway and the checkpoint, which are followed by activation of certain CHKREC nodes. Nonetheless, the activation of the CyclinB-CDK1 node does not occur, thus avoiding cell division (Figures 2C,D).

#### 3.1.2. The FA-CHKREC Simulations Show That Multiple Pathways of DNA Damage Tolerance Might Exist in FA Pathway Deficient Cells

To investigate the process that is responsible for DDA in FA pathway deficient cells we simulated the dynamics of different FA pathway mutants. In Figures 2E–G we show that FAcore, FANCD2 and NUC1 mutants reach a CCP attractor with DDA, in which the system activates the CycB-CDK1 node despite the presence of ICLs, DSBs and gH2AX, thus the model recapitulates the capability that FA pathway deficient cells have to divide with unrepaired DNA damage, schematically represented in Figure 2H. A representative metaphase from a FA cell with unrepaired DNA damage in form of chromosome breakages is shown in Figure 2I.

**Figure 3:**
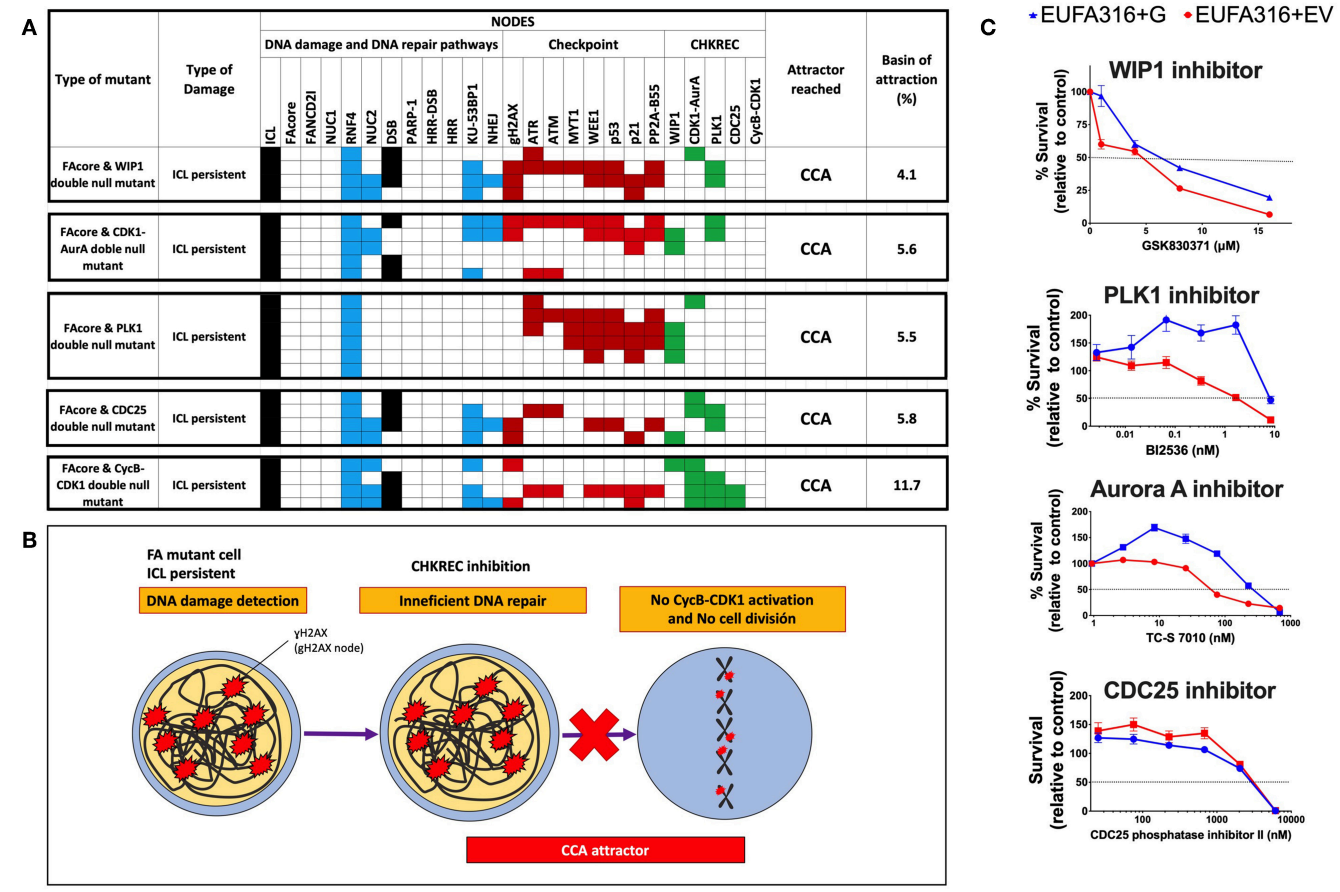
Inactivation of CHKREC nodes in FA mutants promotes CCA and reduces FA cell survival. **(A)** Double KO simulations of the FAcore and components of the CHKREC (WIP1, CDK1-AurA, PLK1, CDC25, and CycB-CDK1) showing that FA cell division will be blocked since the CycB-CDK1 node cannot be activated, driving the system to a cyclic CCA attractors. Only attractors are shown. Nodes in the simulations are grouped by color according to functional categories: DNA damage in black, DNA repair pathways in blue, Checkpoint in red and CHKREC in green. Inactive nodes are colorless, whereas active nodes are colored according to their functional category. **(B)** Schematics showing that upon CHKREC inhibition, the division of FA mutant cells with unrepaired DNA damage will be blocked and the cell will remain in a CCA attractor. In biological terms, cell division blockade may drive the cell to senescence or cell dead. **(C)** Screening of multiple CHKREC chemical inhibitors showing that the FAcore mutant cell line EUFA316+EV (*FANCG* deficient) is more sensitive to CHKREC inhibition than its corrected counterpart EUFA316+G. Refer to Supplementary Materials S2, S3, to see the trajectories followed by these and other FAcore and FANCD2I double null mutants, respectively, before arriving to an attractor.

To identify nodes relevant for DDA in FA pathways deficient cells, we simulated the FAcore null mutant in combination with all the other possible null mutants of the model, an approach that has been previously used to find potential therapeutic targets using BNMs (Poret and Boissel, 2014). Figure 3A shows that in the FAcore and CHKREC double null mutants inactivation of the checkpoint is no longer possible, thus driving the system to CCA attractors, in biological terms the cell is arrested with no possibilities to divide, as schematically represented in Figure 3B. Refer to Supplementary Materials S2, S3 for a complete FAcore and FANCD2I double null mutant simulations.

### 3.2. New Interactions

As previously mentioned, we included in the FA-CHKREC BNM 15 unreported interactions. These are indicated in Figure 1 as blue arrows for inferred positive regulations, and as dashed purple arrows for inferred negative regulations (see also Table S2). Importantly, if these 15 interactions are removed the model is not able to recover all the attractors relevant for the cellular dynamics studied in this work. Without these 15 interactions all the initial conditions of the system are driven exclusively toward a CCP attractor (see Table S1B). The relevance of these 15 interactions in cellular context will be described below.

#### 3.2.1. Fork Replication Collapse and Generation of DSBs by Pathways Alternative to the FA/BRCA Pathway

The FA pathway mutants are inefficient at repairing ICLs. However, they do not seem to be totally deficient in the generation of DSBs (Rothfuss and Grompe, 2004), and might activate alternative ICL-unhooking pathways. To reflect this aspect in the FA-CHKREC BNM, we added positive interactions of RNF4 and PLK1 nodes over the alternative endonucleases node (NUC2). RNF4 is an ubiquitin E3 ligase that promotes the formation of DSBs in ATR-depleted cells and promotes the collapse of replication forks into DSBs. RNF4 accomplishes this through the recruitment and activation of SLX4 Structure-Specific Endonuclease Subunit (SLX4)-associated endonucleases or through replisome remodeling, thus making the stalled replication fork accessible to DNA-processing enzymes and endonucleases (Galanty, 2012; Ragland et al., 2013).

PLK1 is a kinase that participates in several processes, including the resolution of stalled replication forks (van Vugt et al., 2010); it has been shown that PLK1 regulates the activation of MUS81 Structure-Specific Endonuclease Subunit (MUS81) and Essential Meiotic Structure-Specific Endonuclease 1 (EME1) endonucleases and stimulates its association with SLX4 (Szakal and Branzei 2013). Other endonucleases that might also take charge of the alternative ICL unhooking include FANCD2/FANCI-Associated Nuclease 1 (FAN1) and Exonuclease 3'-5' Domain Containing 2 (EXDL2) (Smogorzewska, 2010). In the wild-type network, this alternative ICL-unhooking pathway must be inhibited to avoid any possible interference with the canonical FA pathway. In our model the R-DSB and NUC1 nodes inhibit the NUC2 node, whereas FAcore inhibits RNF4. The existence of an alternative ICL-unhooking pathway deserves additional research since its presence might collaborate in the chemo-resistance observed in FA pathway deficient tumors.

#### 3.2.2. Inactivation of DNA Repair Proteins: the CHKREC Components Might be Important to Negatively Regulate the DNA Damage Repair Proteins

A requisite for entering mitosis, besides inactivating the checkpoint, is the inactivation of the DNA damage repair response, so as to avoid, for example, illegitimate repair of the telomeric chromosome ends during mitosis (Orthwein, 2014). The biochemistry of this process has been previously studied and some proteins that negatively regulate the FA pathway have been described (Nijman, 2005; Kee et al., 2009), however during the reconstruction of the FA-CHKREC BNM we observed that a negative regulation arising from the components of the CHKREC seems to exist and we included negative interactions above HRR arising from PLK1 and CycB-CDK1 node.

PLK1 and CyclinB-CDK1 appear as negative regulators of the HRR and NHEJ nodes. To the best of our knowledge, there is no evidence about a direct inhibition of PLK1 over these DNA repair proteins before entering mitosis. However, mitotic entry has been associated with high activity of the CyclinB-CDK1 complex. CyclinB-CDK1 seems to be responsible, directly or indirectly, for the inhibition of Ring Finger Protein 8 (RNF8) (Orthwein, 2014), an E3 ubiquitin ligase that in association with Ring Finger Protein 168 (RNF168) recruits DNA repair factors (Fradet-Turcotte A., 2013). In addition, the recruitment of 53BP1, another member of the NHEJ pathway, seems to be actively inhibited during mitosis by the CyclinB-CDK1 complex (Orthwein, 2014), therefore we included inhibitory interactions over the NHEJ node arising also from PLK1 and CycB-CDK1 nodes. The mechanistic details of this inhibition remain to be described.

#### 3.2.3. Inactivation of Checkpoint Proteins: the CHKREC in Conjunction With the NHEJ Might Negatively Regulate the G2 Checkpoint Components

For DDA, cells should inactivate the components that repress its division, namely the G2/M checkpoint. Accordingly, we included inhibitory interactions emerging from PLK1, KU-53BP1, and CycB-CDK1 to collaborate with the inactivation of the checkpoint. PLK1 and 53BP1 might inhibit ATM, whereas Cyclin B-CDK1 might inhibit MYT1 and WEE1. Interestingly, when KU-53BP1 is inhibited in FA cells, the cell recovers its DNA repair capabilities (Pace et al., 2010; Renaud et al., 2016), this has been adjudicated to inactivation of the error-prone NHEJ, however and according to our model, the inhibition of KU-53BP1 might in addition set loose any inhibition over ATM, and allow enough time for completion of alternative DNA repair. The consequences that the inhibition of PLK1 and CyclinB-CDK1 might have in FA cells have not been experimentally assessed.

### 3.3. Chemical Inhibition of CHKREC Components Avoids FA Pathway Deficient Cells to Reach Cell Division, in Accordance With the FA-CHKREC BNM

According to our simulations (Figure 3A), turning off the CHKREC nodes dampens cell division capacity and drives the simulation to CCA attractor, therefore, chemical inhibition of CHKREC components might prevent FA mutants, such as FAcore, from reaching cell division (Figure 3B). We tested this model-derived hypothesis in the EUFA316+EV cell line, a lymphoblast cell line derived from a FA patient with inherited mutations in *FANCG*, using chemical inhibitors against the components of the CHKREC nodes, namely GSK830371 (Sigma-Aldrich) for WIP1 inhibition, TC-S 7010 (Selleckchem) for inhibiting Aurora A, BI2536 (Selleckchem) for inhibiting PLK1 and CDC25 Phosphatase Inhibitor II (Santa Cruz Biotechnologies) for inhibiting CDC25. Figure 3C shows survival curves from the different CHKREC inhibitors tested. Of note, FA deficient cells were more sensitive than their corrected counterparts (EUFA3156+G) to chemical inhibition of WIP1, PLK1 and Aurora A, and to a lesser extent to CDC25 inhibition. We decided to keep studying WIP1 phosphatase since it remains poorly explored and WIP1 inhibitors are gaining relevance in the oncology field, WIP1 inhibition, for example, has been associated to reduced tumorigenesis and induction of cell cycle arrest in cancer cells (Belova et al., 2005; Lu et al., 2005; Shaltiel et al., 2014).

**Figure 4:**
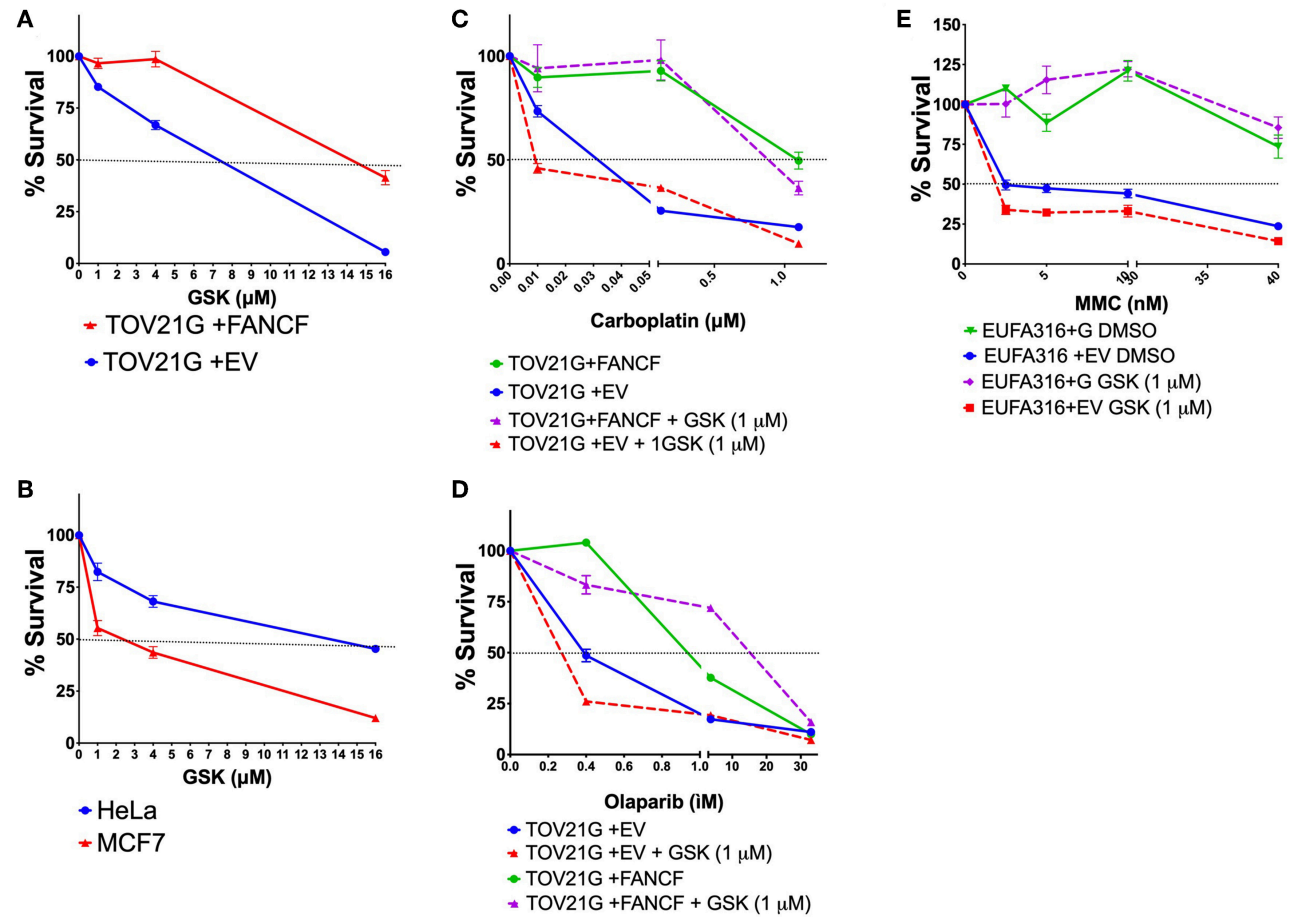
WIP1 is required for DDA in FA pathway deficient cell lines. **(A)** The ovarian cancer cell line TOV21G+EV deficient in FANCF shows sensitivity to GSK2830371. **(B)** The MCF7 cell line has been previously shown to be sensitive to WIP1 inhibition, whereas the HeLa cell line has been reported to be non-sensitive. **(C)** WIP1 inhibition with GSK2830371 sensitizes the TOV21G+EV ovarian cancer cell line to Carboplatin. **(D)** WIP1 inhibition with GSK2830371 sensitizes the TOV21G+EV ovarian cancer cell line to Olaparib. **(E)** WIP1 inhibition with GSK2830371 sensitizes the EUFA316+EV cell line to MMC.

In addition to the EUFA316+EV cell line, other different FAcore mutants were found to be sensitive to WIP1 inhibition with GSK2830371, including the TOV21G+EV, an ovarian cancer cell line with epigenetic silencing of the *FANCF* gene (Figure 4A), and the HeLa and MCF7 cancer cell lines that have previously been shown to be resistant and sensitive to GSK2830371, respectively (Figure 4B). In addition, co-treatment with GSK2830371 sensitizes the TOV21G+EV cell line to treatment with carboplatin, an inductor of interstrand-crosslinks (Figure 4C), and Olaparib, a PARP inhibitor (Figure 4D); GSK2830371 also sensitizes the EUFA316 cell line to MMC (Figure 4E).

**Figure 5:**
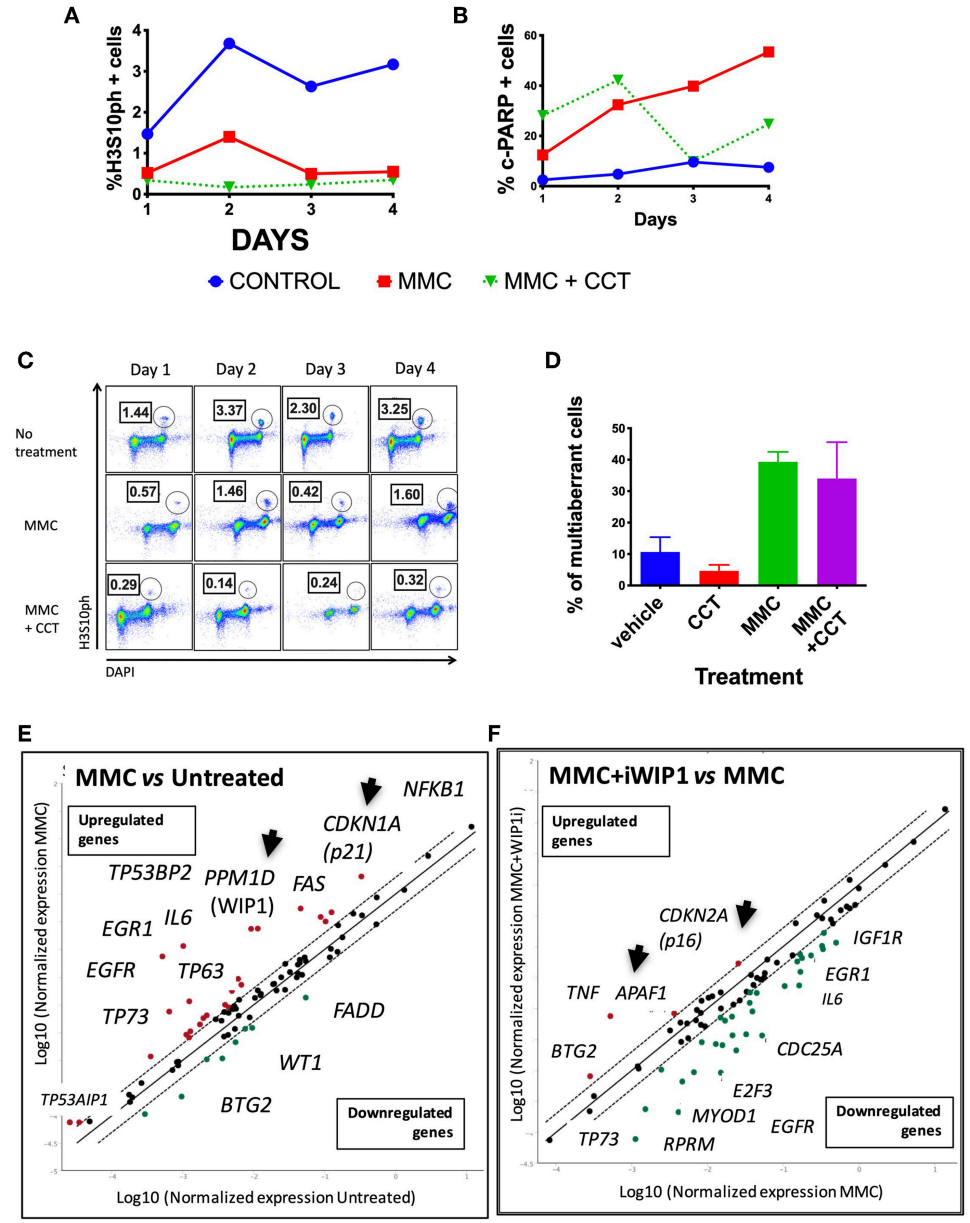
FA pathway deficient cells have a threshold of tolerance to DNA damage that might be sustained by the CHKREC. Co-treatment with the WIP1 inhibitor CCT007093 along with MMC reduces the number of cells in M phase (H3S10PH+) **(A)** and increases the numbers of c-PARP+ cells (a marker of apoptosis) in the VU817 cell line **(B)**, thus indicating that failing to reach the CCP-DDA attractor drives the cells toward cell cycle arrest or activation of cell death mechanisms. **(C)** Representative FACS plots showing how the mitotic index of the VU817 cell line is reduced by WIP1 inhibition with CCT007093. **(D)** Co-treatment of FA cells with CCT007093 does not modify the number of CA observed in MMC-treated FA-A cells. **(E)** Upon MMC treatment the VU817 FA-A cell line simultaneously over-expresses the genes codifying for WIP1 (*PPM1D*) and for p21 (*CDKN1A*). **(F)** Inhibition of WIP1 with CCT007093 in MMC treated cells changes the expression pattern toward apoptotic genes, such as *APAF1*, and definitive arrest genes, such as *CDKN2A*, which codifies for p16.

Seven different concentrations of MMC (4–1,000 ng/ml) were evaluated during 4 days of continuous treatment to monitor the maximum amount of DNA damage that a FAcore mutant cell line can tolerate, in this case the VU817 cell line, mutant in the *FANCA* gene (Figure S2). 10 ng/ml of MMC allows the division of 100% multi-aberrant cells with up to 10 chromosomal aberrations per cell, in addition, almost the entire culture is positive for γH2AX staining, a marker of unrepaired DSBs. Despite the dramatic amount of unrepaired DNA damage, an important number of cells reaches mitosis and cell death remains below 10%, suggesting that this is an amount of DNA damage in which DDA occurs (Figure S2). The effect of WIP1 inhibition was studied in this setting and we evaluated cell division parameters. We observed that, effectively WIP1 might contribute to DDA in FA-A cells, since its inhibition with the chemical inhibitor CCT007093 (Sigma-Aldrich) reduces the mitotic index, measured by cells positive for phosphorylation of the mitotic marker H3S10, and activates apoptosis, measured by PARP-cleavage positive cells (Figures 5A,B). Figure 5C shows how treatment with CCT007096 reduces the mitotic index (H3S10ph+ circled population) of FA cells with respect to untreated cultures and MMC-treated cultures. WIP1 inhibition with CCT007093 does not modify the number of chromosome aberrations (Figure 5D), suggesting that WIP1 phosphatase is not directly involved in DNA repair mechanisms, but controls cell cycle transitions.

WIP1 collaborates turning-off the checkpoint through dephosphorylation of several of its components including p53, CHK1, CHK2, γH2AX, and the ATR and ATM kinase target motif *p(S/T)Q* (Lindqvist et al., 2009a; Shaltiel et al., 2014), therefore in its absence the checkpoint machinery remains active and the continuation of the cell cycle is not allowed. In such a situation of long lasting unrepaired DNA damage two scenarios are feasible, a DNA damage induced senescence, which has previously been described in other contexts of unrepairable DNA damage (von Zglinicki et al., 2005; Kim et al., 2014) or activation of apoptosis. We confirmed the gene expression landscape for these scenarios using a real time PCR array that simultaneously detects 84 DNA damage related transcripts (QIAGEN). First we found that both *PPM1D* and *CDKN1A*, which codify for WIP1 and p21 proteins, respectively, become activated in FA-A cells after exposure to MMC, thus demonstrating that the CHKREC program becomes activated in presence of DNA damage (Figure 5E). Furthermore, chemical inhibition of WIP1 in MMC-treated FA-A cells activated the expression of definitive cell cycle arrest genes and apoptotic genes, namely *CDKN2A* (which codifies for p16) and *APAF1*, respectively (Figure 5F), supporting our notion that WIP1 is critical for division and survival of FA cells with unrepaired DNA damage.

In summary, our results show that FA cells with a large amount of DNA damage preserve the capacity of cell division after DNA damage induction, a result that is consistent with the FA-CHKREC BNM simulations. The finding that inhibition of WIP1 in particular, and of the CHKREC in general, prevents cell cycle progression in FA-A cells deserves further exploration so as to elucidate a potential larger impact of CHKREC studies in the FA field. CHKREC stimulation would allow survival of the hematopoietic stem cell compartment and amelioration of the total cell count numbers, however overexpression of the same components may conduct to stem cell pool attrition and potential selection of malignant cell clones. Given this, a profound knowledge of this process is necessary to understand the balance between the normal hematopoiesis and the hematopoiesis resulting from CHKREC hyper-activation, the later in a context of DNA repair deficiency might result in hematopoietic stem cells with high amounts of DNA damage and potentially contribute to cancer.

## 4. Conclusion and Perspectives

In this study we propose the FA-CHKREC Boolean network model and use it as a tool to suggest hypotheses to explain the imbalance between the checkpoint and CHKREC that generates DDA in FA pathway mutant cells. We consider that understanding how the FA pathway and its mutants interact with the checkpoint and CHKREC processes is fundamental to develop innovative therapeutics for diseases in which this DNA repair pathway is disrupted, such as FA and many types of cancer. Our model was designed to study the mechanisms that allow the escape of cells with unrepaired DSBs in the FA cellular phenotype, and observed that the ectopic activity of the components of the CHKREC and the NHEJ seems to be critical for DDA in FA cells. In a first approach we observed that, as anticipated by the model, WIP1 is necessary for DDA and that its inhibition prevents the division of FA-A cells. Importantly, WIP1 and several CHKREC components are considered to be oncogenes, therefore their function deserves to be scrutinized in different settings, such as FA and cancer.

## Author Contributions

AR and JJN developed the model. AR, LT, BGT, UJ-F, and CA-Z performed experiments. EA and LM supervised modeling development. AR, LM, and SF wrote the manuscript. SF supervised the entire work. All authors approved the final manuscript.

### Conflict of Interest Statement

The authors declare that the research was conducted in the absence of any commercial or financial relationships that could be construed as a potential conflict of interest.
