# Supplementary material for: WIP1 Contributes to the Adaptation of Fanconi Anemia Cells to DNA Damage as Determined by the Regulatory Network of the Fanconi Anemia and Checkpoint Recovery Pathways
Source: Front Genet. 2019 May 3;10:411. doi: 10.3389/fgene.2019.00411 (PMC6509935; doi:10.3389/fgene.2019.00411)
Supplement: Supplementary file 1 [file Data_Sheet_1.pdf]

Supplementary Material: WIP1 adapts Fanconi anemia cells to DNA damage as determined by the regulatory network of the Fanconi anemia and checkpoint recovery pathways.

1 SUPPLEMENTARY TABLES AND FIGURES

1.1 Tables

Node	Proteins included in the node	Function
ICL	—	DNA damage
FAcore	FANCM, FANCA, FANCB, FANCC, FANCE, FANCF, FANCG, FANCL, FAAP24, FAAP100	FA/BRCA
FANCD2I	FANCD2, FANCI, UBE2T	FA/BRCA
NUC1	SLX4, MUS81, EXO1, DNA2, FAN1, CtIP, MRE11, Rad50, NBS1	FA/BRCA and DDR
RNF4	RNF4	SRF collapse
NUC2	SLX4, FAN1, EXDL2	SRF collapse
DSB	—	DNA damage
PARP-1	PARP-1	A-NHEJ
R-DSB	RAD51, TIP60 (includes resected DNA, H4K16 acetylation and other epigenetic marks not yet identified)	FA/BRCA
HRR	BRCA1, BRCA2, BRIP, PALB2, RAD51, RAD51C, BLM	FA/BRCA
KU-53BP1	Ku70, Ku80, 53BP1	NHEJ
NHEJ	DNA-PKcs, Artemis, XRCC4	NHEJ
γH2AX	H2AX	DDR
ATR	ATR, CHK1	DDR
ATM	ATM, CHK2	DDR
MYT1	MYT1	DDR
WEE1	WEE1	DDR
p53	p53	DDR
p21	p21	DDR
PP2A-B55	PP2A-B55	DDR
WIP1	WIP1	CHKREC
CDK1-AurA	CDK1, AURKA, BORA	CHKREC
PLK1	PLK1	CHKREC
CDC25	CDC25	CHKREC
CycB-CDK1	CCNB1, CDK1	CHKREC

Table S1. Functional classification of the proteins and nodes included in the FA-CHKREC BNM

Logical rule	Functional process	Modifications**
ICL ← ICL ∧ ¬ (NUC1 ∨ NUC2)	DNA damage	Rewritten. Simplified
FAcore ← ICL ∧ ¬ ((RNF4* ∧ PLK1) ∨ (FAcore ∧ ¬ ATR))	Upstream FA/BRCA	Rewritten. Simplified
FANCD2I ← FAcore ∧ (ATM ∨ ATR) ∧ ¬ FANCD2I*	Upstream FA/BRCA	Rewritten
NUC1 ← (ICL ∧ FANCD2I) ∨ (DSB ∧ PARP-1)	Downstream FA/BRCA	DSB and PARP-1 interactions added
RNF4 ← ICL ∧ ¬ FAcore*	Alternative ICL unhooking	New node
NUC2 ← ICL ∧ RNF4* ∧ PLK1* ∧ ¬ (R-DSB* ∨ NUC1*)	Alternative ICL unhooking	Rewritten. New CHKREC interactions.
DSB ← (DSB ∨ ICL ∧ NUC2) ∧ ¬ (NHEJ ∨ NUC1)	DNA damage	Rewritten. Simplified
PARP-1 ← (DSB ∨ R-DSB) ∧ gH2AX ∧ ¬ (KU-53BP1)	Alternative ICL repair	New node
R-DSB ← (R-DSB ∨ ((ICL ∨ DSB) ∧ NUC1)) ∧ ¬ (HRR)	DNA damage	New node
HRR ← gH2AX ∧ R-DSB ∧ ATM ∧ ¬ (PLK1* ∧ CycB-CDK1*)	Downstream FA/BRCA	Rewritten. New CHKREC interactions
KU-53BP1 ← DSB ∧ ¬ PARP-1	Upstream NHEJ	New node
NHEJ ← KU-53BP1 ∧ DSB ∧ ATM ∧ ¬ (PLK1* ∧ CycB-CDK1*)	Downstream NHEJ	Rewritten. New CHKREC interactions
gH2AX ← (DSB ∨ R-DSB) ∧ (ATM ∨ ATR ∨ gH2AX ∨ KU-53BP1) ∧ ¬ (WIP1 ∧ PP2A-B55)	Checkpoint	Rewritten
ATR ← (ICL ∨ ATM) ∧ ¬ (WIP1 ∨ (PLK1 ∧ KU-53BP1))	Checkpoint	New CHKREC interactions. KU-53BP1 interaction added
ATM ← (ATR ∨ DSB ∨ R-DSB ∨ NUC1 ∨ FAcore) ∧ ¬ (WIP1 ∨ PP2A-B55 ∨ (PLK1* ∧ KU-53BP1*))	Checkpoint	New CHKREC interactions. New DDR and NHEJ interactions
MYT1 ← (ATM ∨ ATR) ∧ ¬ (CDC25 ∨ CycB-CDK1* ∨ PLK1)	Checkpoint	New node

WEE1 ← (ATM ∨ ATR ∨ PP2A-B55) ∧ ¬ (CDC25 ∨ CycB-CDK1* ∨ PLK1)	Checkpoint	New node
p53 ← (ATM ∨ ATR) ∧ ¬ (WIP1 ∧ (PLK1 ∨ CDK1-AurA))	Checkpoint	New CHKREC interactions
p21 ← p53	Checkpoint	New node
PP2A-B55 ← (ATM ∨ ATR) ∧ ¬ CycB-CDK1	Checkpoint	New node
WIP1 ← p53	CHKREC	New node
CDK1-AurA ← CycB-CDK1 ∨ CDC25 ∨ ¬ (p21 ∧ PP2A-B55) ∧ ¬ (WEE1 ∨ MYT1 ∨ ATM ∨ ATR)	CHKREC	New node
PLK1 ← CycB-CDK1 ∨ (ICL ∧ ATR ∧ ¬ FAcore) ∨ ((CDK1-AurA) ∧ ¬ (MYT1 ∨ WEE1 ∨ ATR ∨ ATM))	CHKREC	New node
CDC25 ← CycB-CDK1 ∨ (PLK1 ∧ (CycB-CDK1 ∨ CDK1-AurA) ∧ ¬ ((WEE1 ∨ MYT1) ∧ (PP2A-B55 ∨ ATM ∨ ATR)))	CHKREC	New node
CycB-CDK1 ← CycB-CDK1 ∨ (CDC25 ∧ Plk1 ∧ CDK1-AurA) ∧ ¬ p21	CHKREC	New node

Table S2: Functions used in the FA-CHKREC BNM. (*)Inferred interactions. (**) Modifications from previous BNM.

1.2 Figures

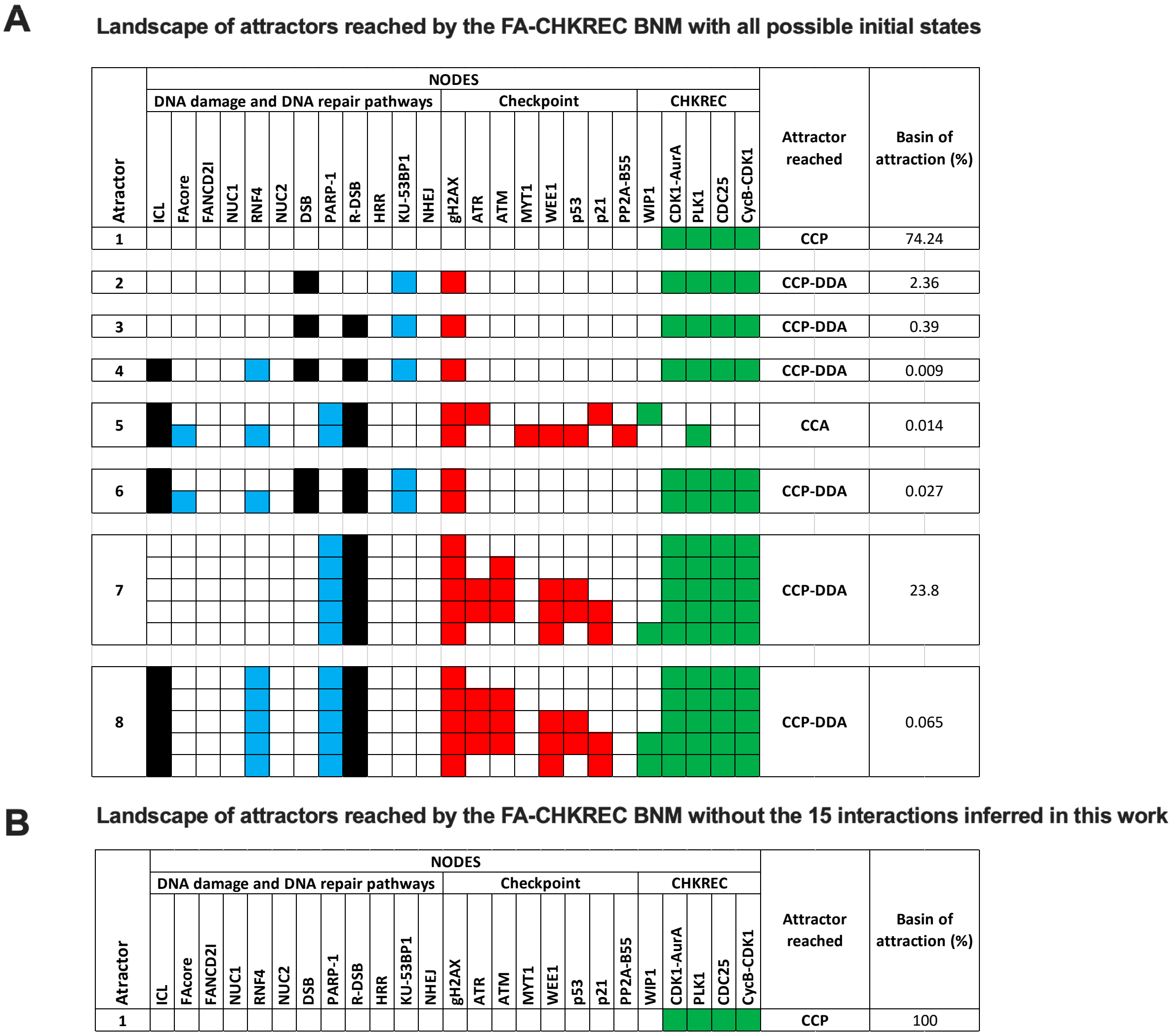

Figure S1: Landscape of attractors of the FA-CHKREC BNM. (A) All possible initial conditions for the FA-CHKREC BNM model were simulated and the landscape of attractors was obtained. (B) The 15 interactions inferred in this work were removed from the FA-CHKREC BNM and all the possible initial conditions were simulated to identify how the landscape of attractors was modified without these interactions.

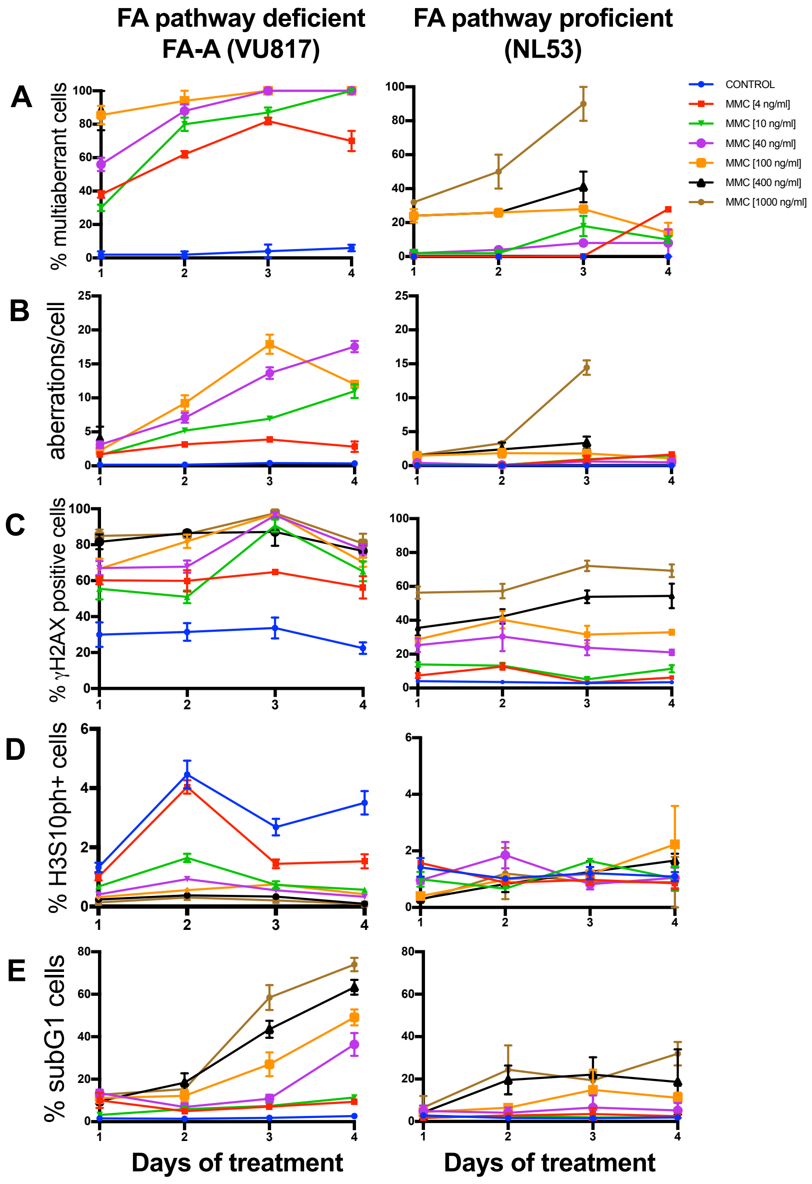

Figure S2: FA pathway deficient cells have a threshold of tolerance to DNA damage that might be sustained by CHKREC. (A) In comparison to normal cells, FA-A (VU817) cell line has an increased amount of multi-aberrant cells ($\geqslant$ 2 ab/cell) arriving to metaphase from very small concentrations of MMC. FA cells do not resist the highest concentrations of MMC as normal cells seem to do. At the highest MMC concentration normal cells (NL53) also allow the escape of unrepaired DSBs. (B) Since small amounts of MMC, FA-A cells have far more chromosome aberrations per cell than normal cells. (C) Around 40% of untreated FA-A cells are positive for γH2AX staining, demonstrating endogenous DNA damage, this amount rises up to 100% with increasing amounts of MMC. (D) The mitotic index (measured by H3S10ph positive cells) of FA-A cells is reduced by MMC treatment, however more than 80% of these cells arrive to mitosis with a large amount of chromosome aberrations (compare with A and B in this same figure). (E) MMC activates cell death (measured by the subG1 fraction of PI stained cells) in a time and dose dependent manner, however small concentrations of MMC do not immediately kill FA-A cells and allow several cell divisions with unrepaired DNA damage, corresponding in biological terms to the CCP-DDA attractor observed in our simulations.
