# Supplementary material for: WIP1 Contributes to the Adaptation of Fanconi Anemia Cells to DNA Damage as Determined by the Regulatory Network of the Fanconi Anemia and Checkpoint Recovery Pathways
Source: Front Genet. 2019 May 3;10:411. doi: 10.3389/fgene.2019.00411 (PMC6509935; doi:10.3389/fgene.2019.00411)
Supplement: Supplementary file 2 [file Data_Sheet_2.pdf]

Supplementary material S1. Single mutant simulations. All the simulations presented in this section have inactivation of the node specified in every page. These simulations were run in presence of a single pulse of ICL or persistent presence of ICL.

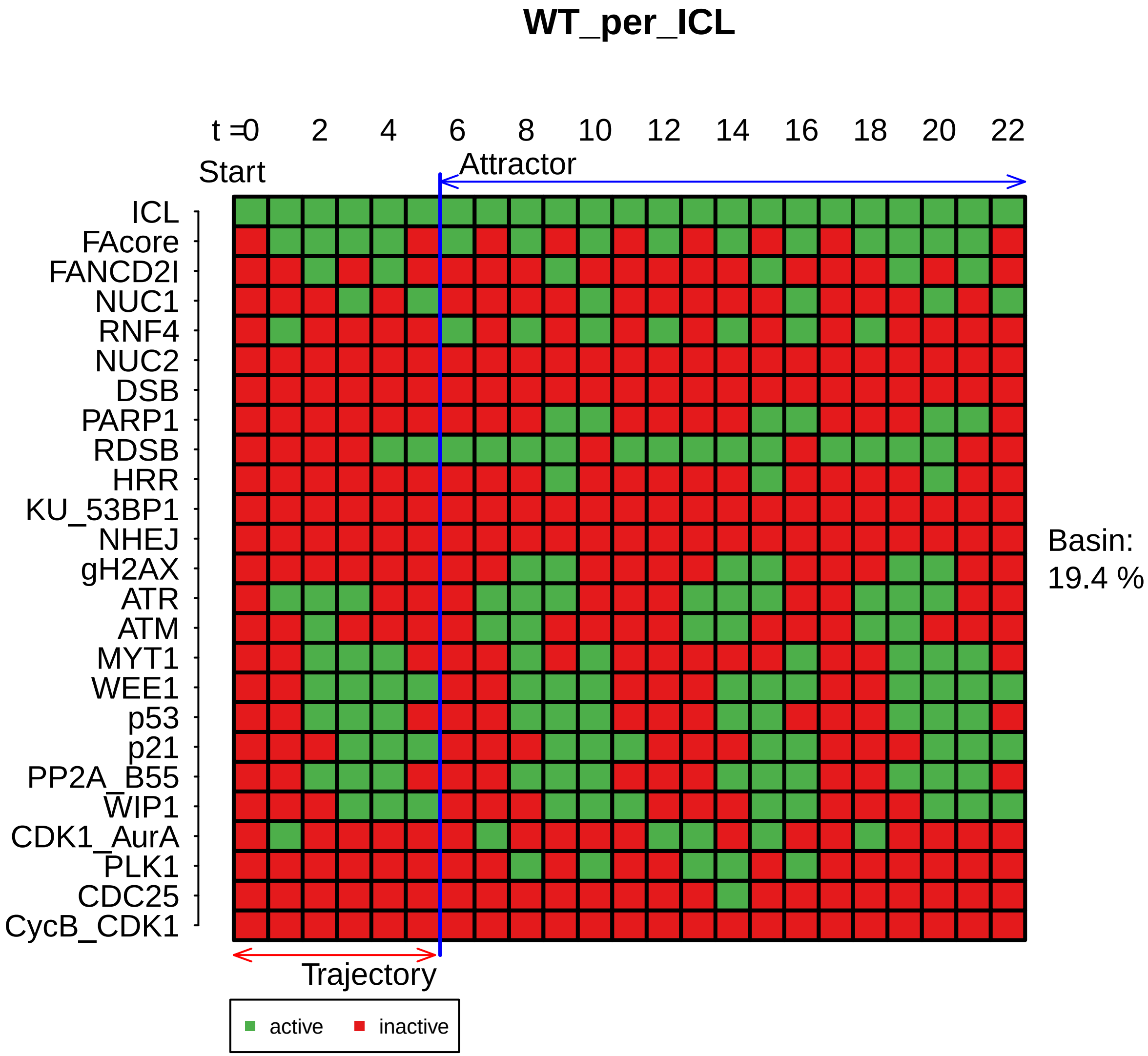

WT_per_ICL
t = 0
2
4
6
8
10
12
14
16
18
20
22
Start
Attractor
ICL
FAcore
FANCD2I
NUC1
RNF4
NUC2
DSB
PARP1
RDSB
HRR
KU_53BP1
NHEJ
gH2AX
ATR
ATM
MYT1
WEE1
p53
p21
PP2A_B55
WIP1
CDK1_AurA
PLK1
CDC25
CycB_CDK1
Basin:
19.4 %
Trajectory
active
inactive

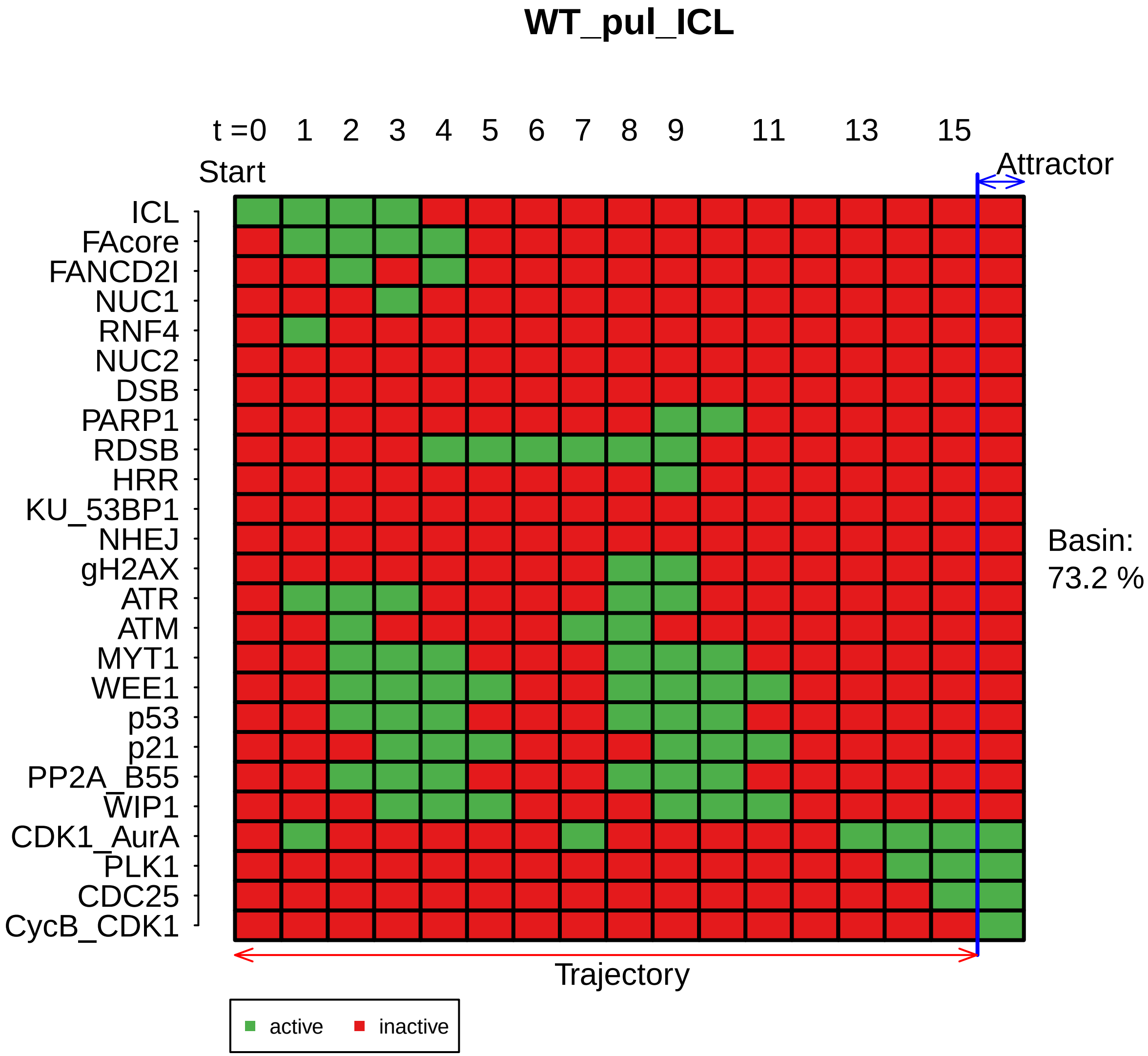
WT_pul_ICL
t =0
1
2
3
4
5
6
7
8
9
11
13
15
Start
Attractor
ICL
FAcore
FANCD2I
NUC1
RNF4
NUC2
DSB
PARP1
RDSB
HRR
KU_53BP1
NHEJ
gH2AX
ATR
ATM
MYT1
WEE1
p53
p21
PP2A_B55
WIP1
CDK1_AurA
PLK1
CDC25
CycB_CDK1
Basin:
73.2 %
Trajectory
active
inactive

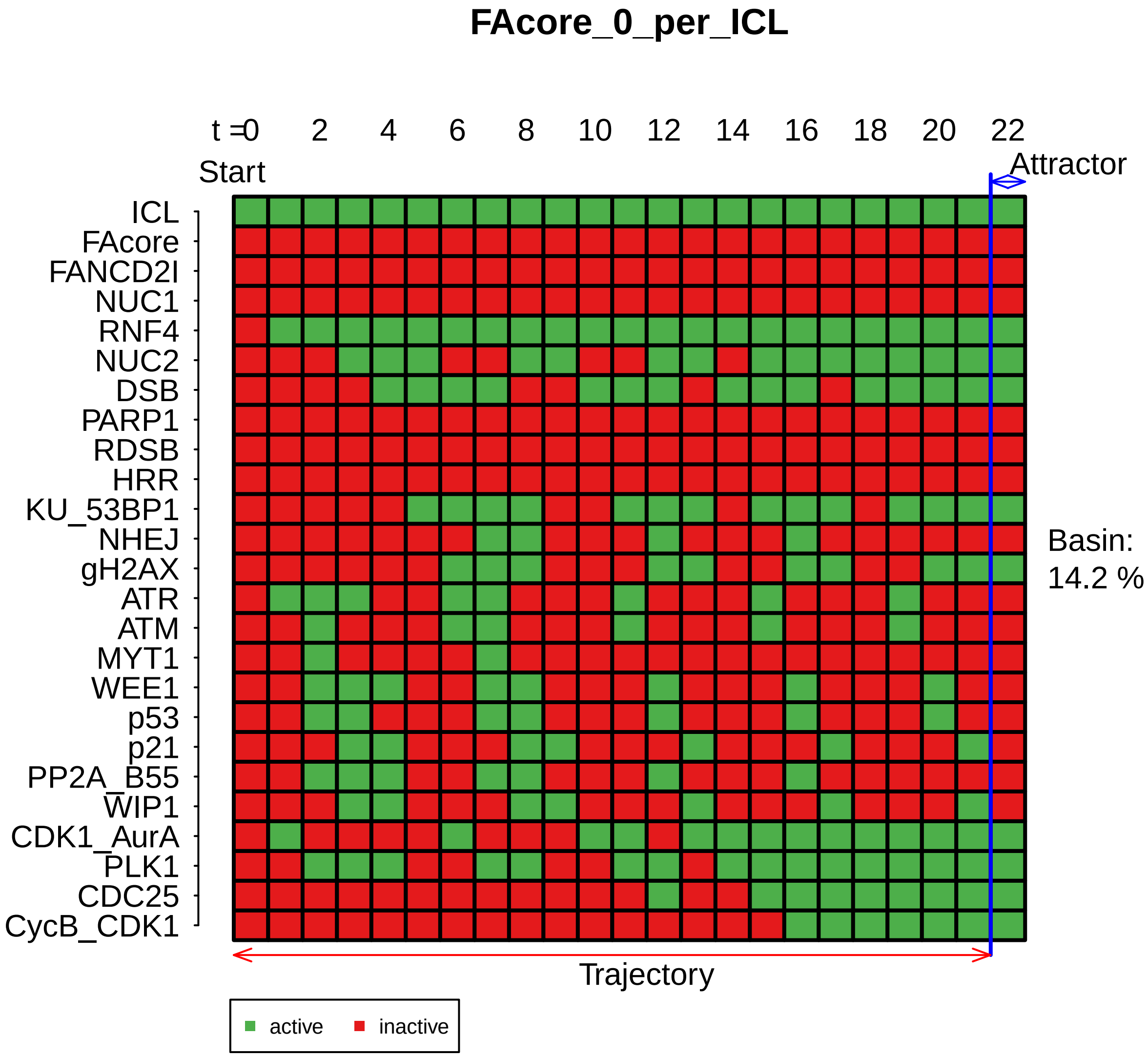

FAcore_0_per_ICL
t = 0 2 4 6 8 10 12 14 16 18 20 22
Start
Attractor
ICL
FAcore
FANCD2I
NUC1
RNF4
NUC2
DSB
PARP1
RDSB
HRR
KU_53BP1
NHEJ
gH2AX
ATR
ATM
MYT1
WEE1
p53
p21
PP2A_B55
WIP1
CDK1_AurA
PLK1
CDC25
CycB_CDK1
Basin:
14.2 %
Trajectory
active
inactive

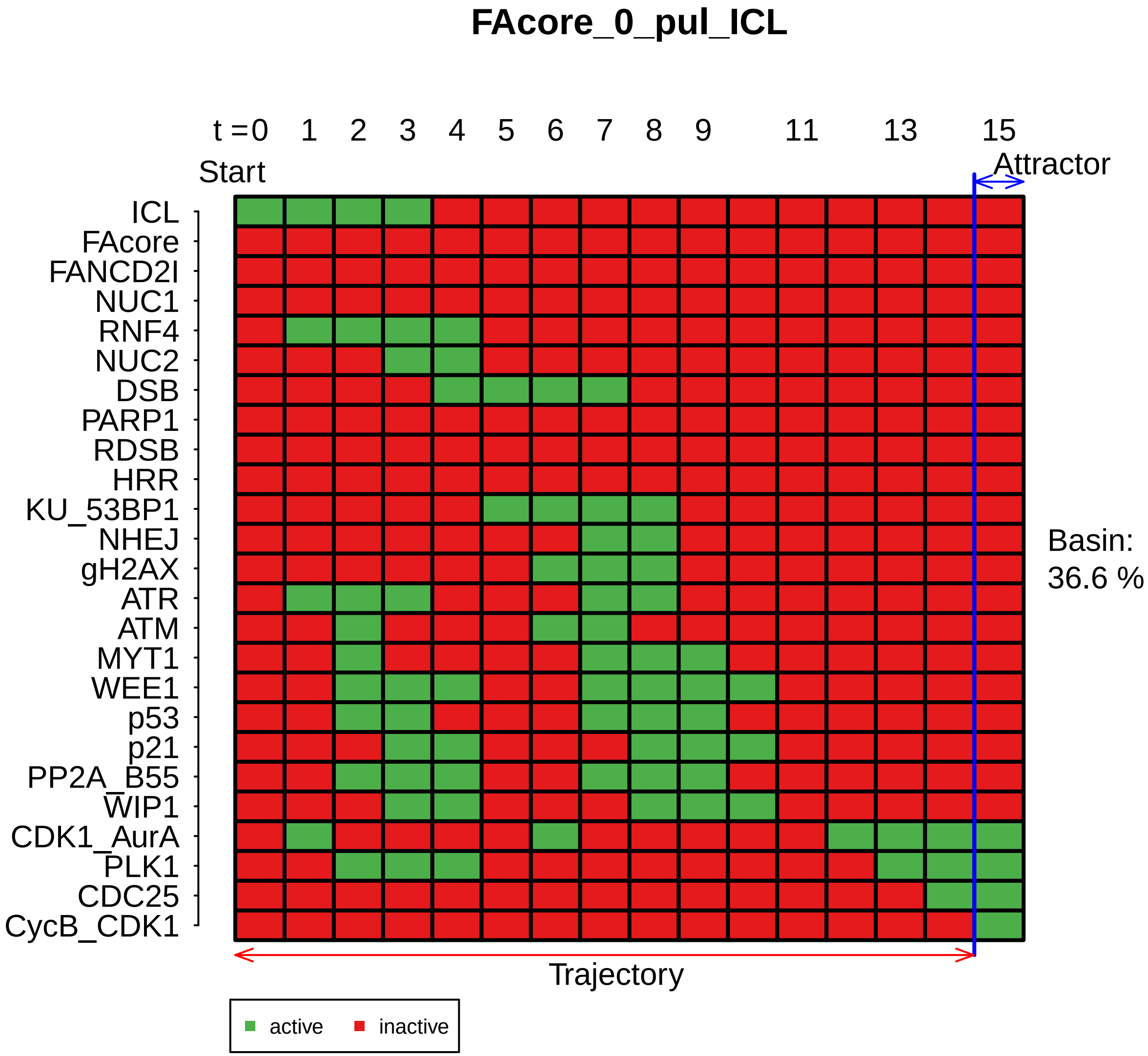

FAcore_0_pul_ICL
t =0 1 2 3 4 5 6 7 8 9 11 13 15
Start
Attractor
ICL
FAcore
FANCD2I
NUC1
RNF4
NUC2
DSB
PARP1
RDSB
HRR
KU_53BP1
NHEJ
gH2AX
ATR
ATM
MYT1
WEE1
p53
p21
PP2A_B55
WIP1
CDK1_AurA
PLK1
CDC25
CycB_CDK1
Basin:
36.6 %
Trajectory
active
inactive

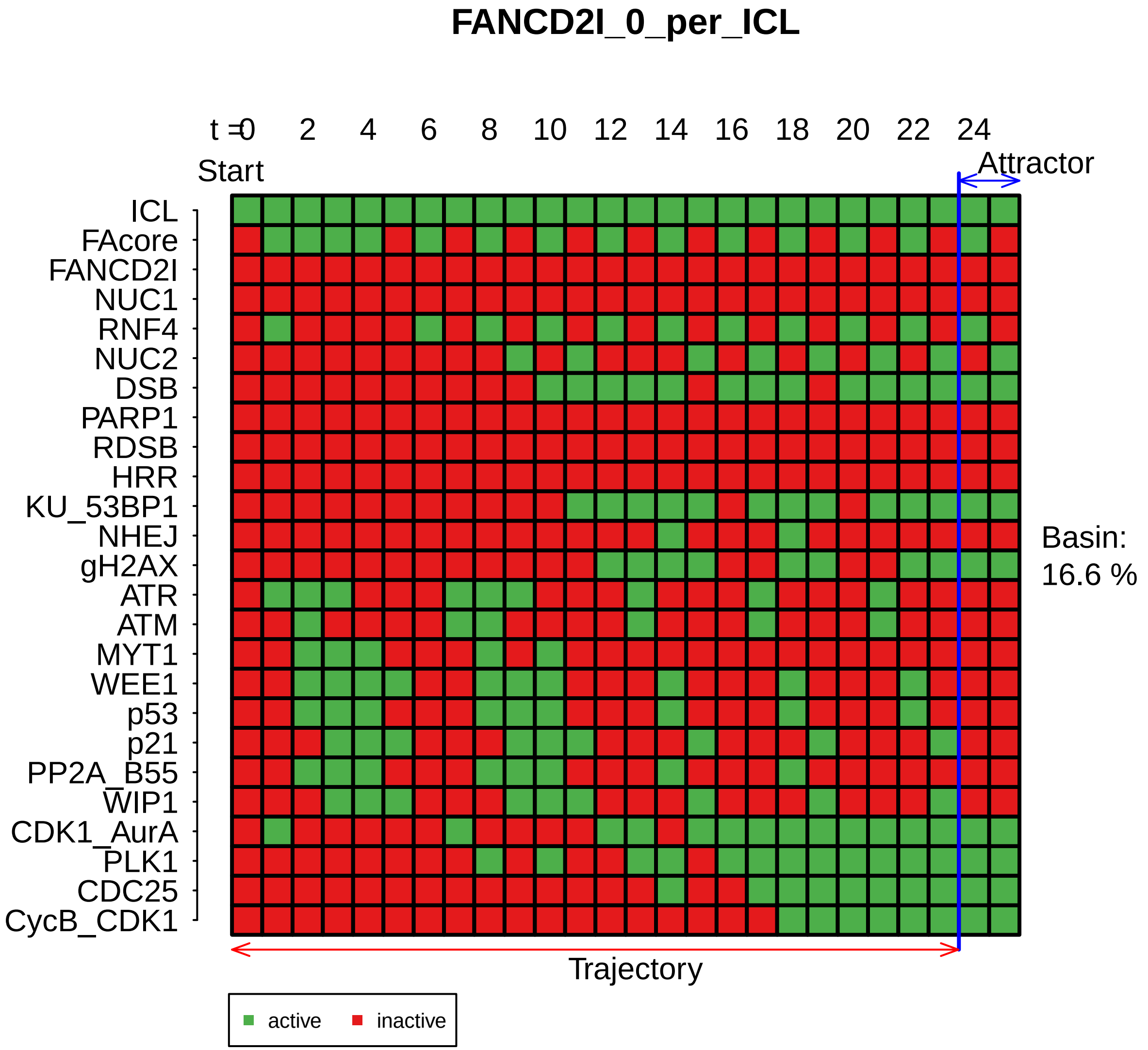
FANCD2I_0_per_ICL
t = 0
2
4
6
8
10
12
14
16
18
20
22
24
Start
Attractor
ICL
FAcore
FANCD2I
NUC1
RNF4
NUC2
DSB
PARP1
RDSB
HRR
KU_53BP1
NHEJ
gH2AX
ATR
ATM
MYT1
WEE1
p53
p21
PP2A_B55
WIP1
CDK1_AurA
PLK1
CDC25
CycB_CDK1
Basin:
16.6 %
Trajectory
active
inactive

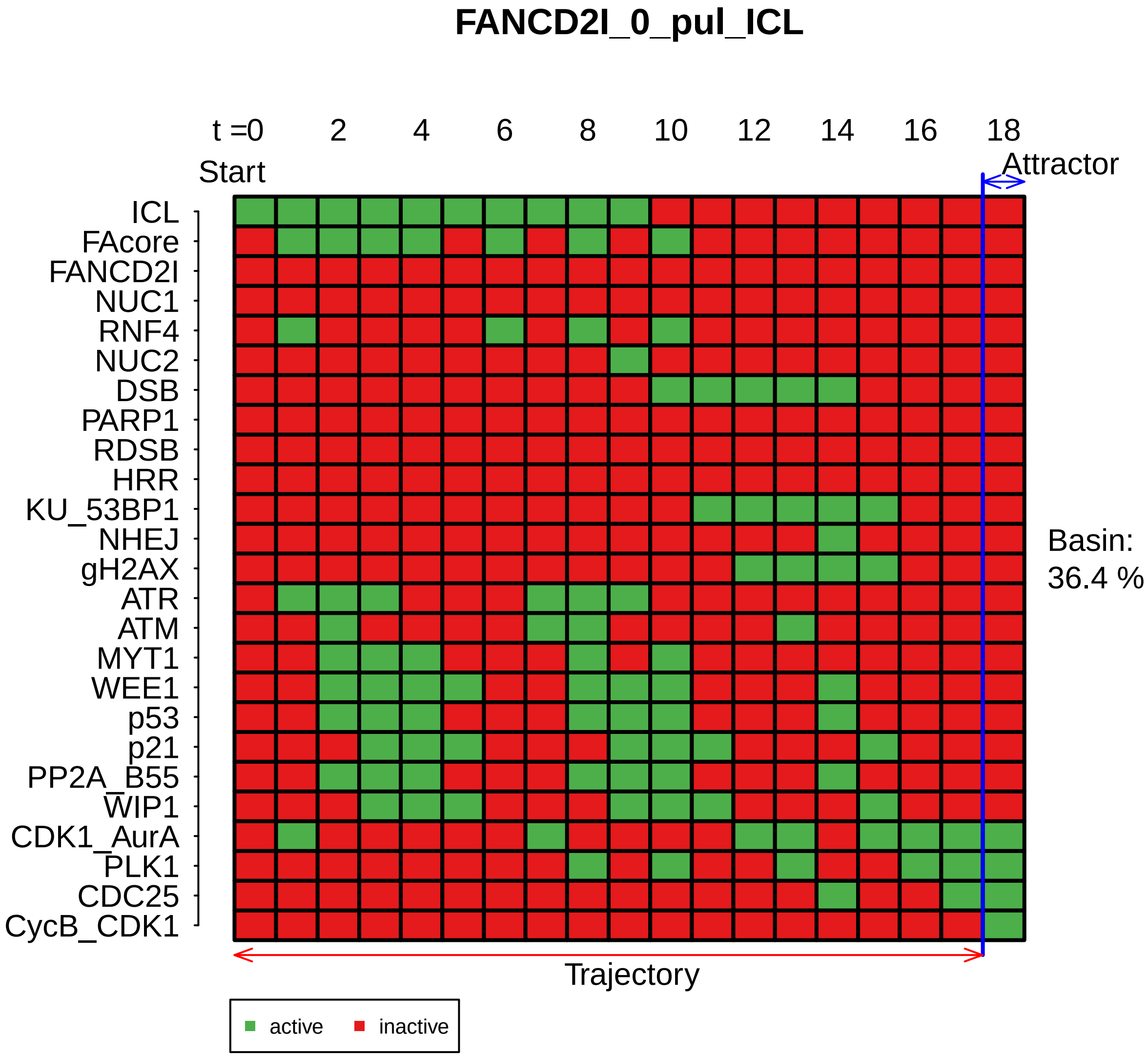
FANCD2I_0_pul_ICL
t =0
2
4
6
8
10
12
14
16
18
Start
Attractor
ICL
FAcore
FANCD2I
NUC1
RNF4
NUC2
DSB
PARP1
RDSB
HRR
KU_53BP1
NHEJ
gH2AX
ATR
ATM
MYT1
WEE1
p53
p21
PP2A_B55
WIP1
CDK1_AurA
PLK1
CDC25
CycB_CDK1
Basin:
36.4 %
Trajectory
active
inactive

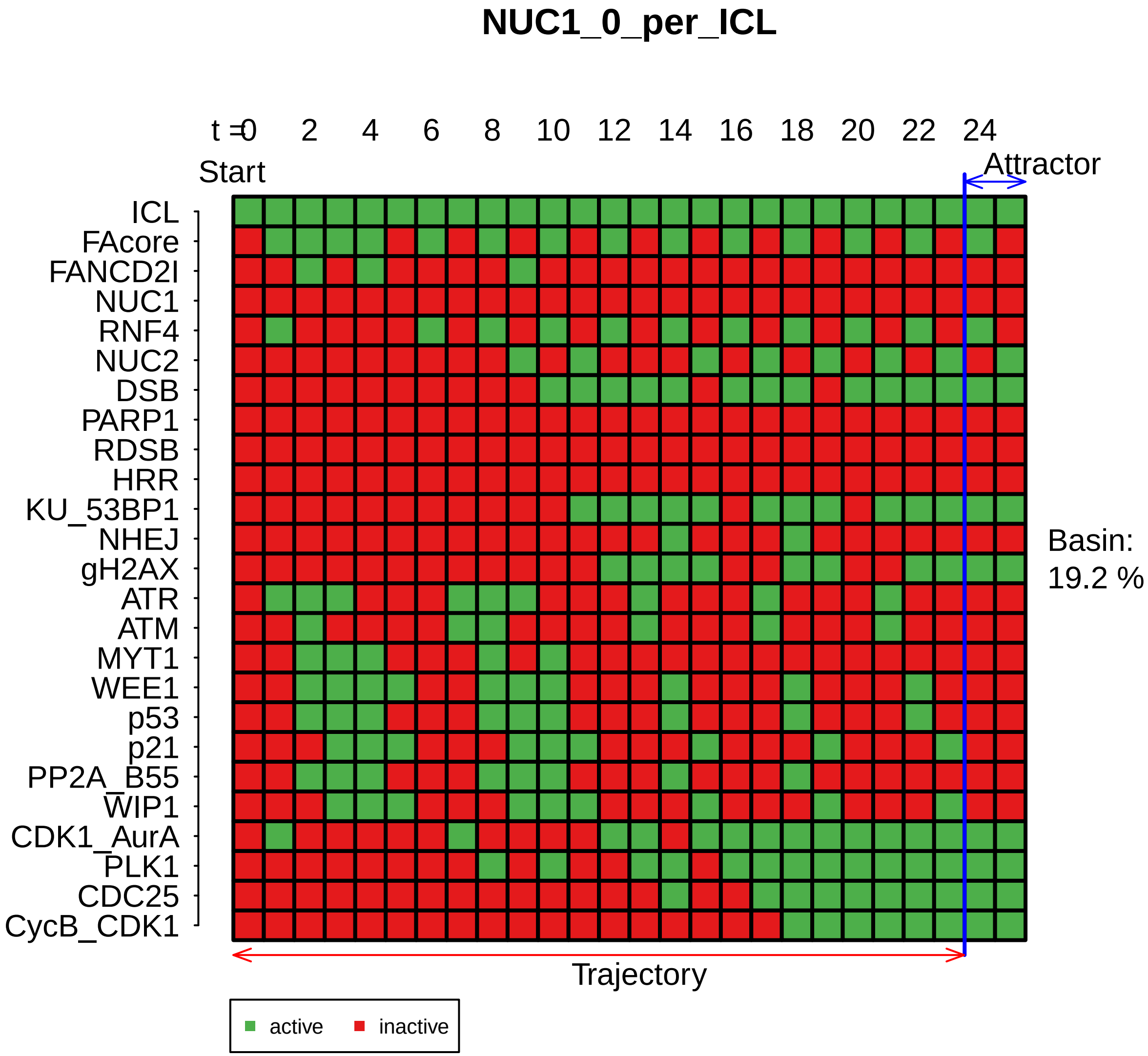
NUC1_0_per_ICL
t = 0 2 4 6 8 10 12 14 16 18 20 22 24
Start
Attractor
ICL
FAcore
FANCD2I
NUC1
RNF4
NUC2
DSB
PARP1
RDSB
HRR
KU_53BP1
NHEJ
gH2AX
ATR
ATM
MYT1
WEE1
p53
p21
PP2A_B55
WIP1
CDK1_AurA
PLK1
CDC25
CycB_CDK1
Basin:
19.2 %
Trajectory
active
inactive

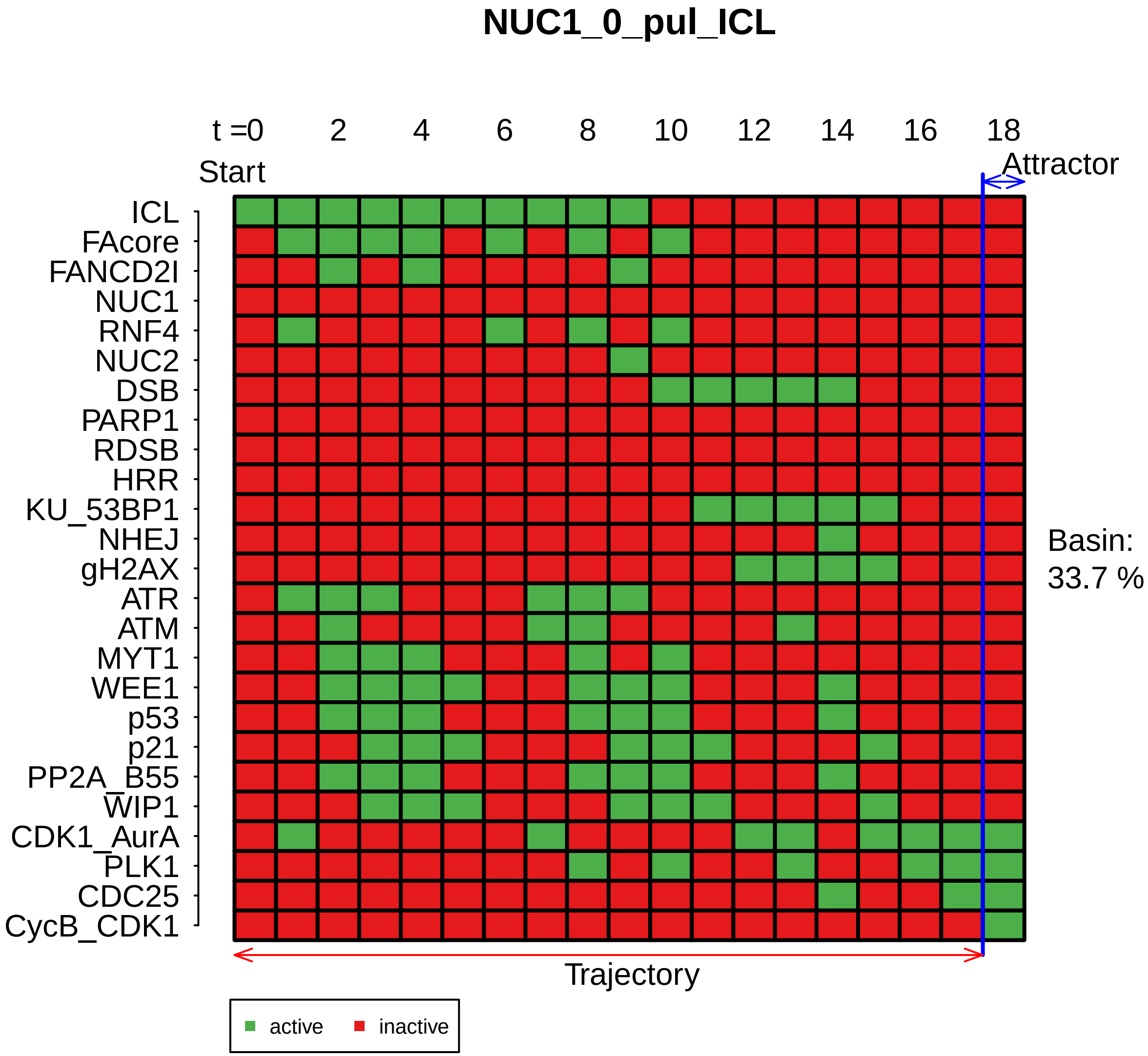

NUC1_0_pul_ICL
t =0
2
4
6
8
10
12
14
16
18
Start
Attractor
ICL
FAcore
FANCD2I
NUC1
RNF4
NUC2
DSB
PARP1
RDSB
HRR
KU_53BP1
NHEJ
gH2AX
ATR
ATM
MYT1
WEE1
p53
p21
PP2A_B55
WIP1
CDK1_AurA
PLK1
CDC25
CycB_CDK1
Basin:
33.7 %
Trajectory
active
inactive

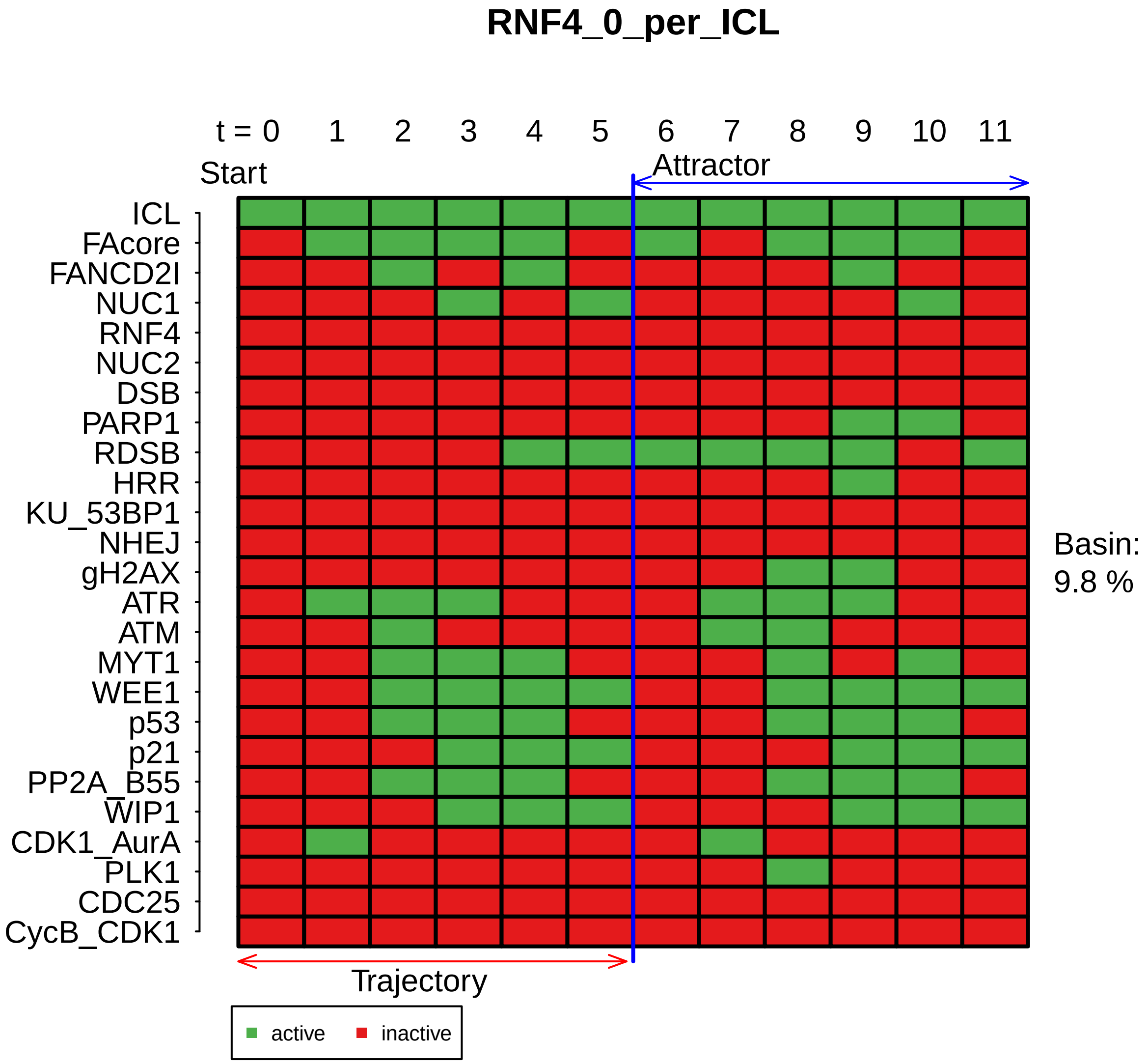

RNF4_0_per_ICL
t = 0
1
2
3
4
5
6
7
8
9
10
11
Start
Attractor
ICL
FAcore
FANCD2I
NUC1
RNF4
NUC2
DSB
PARP1
RDSB
HRR
KU_53BP1
NHEJ
gH2AX
ATR
ATM
MYT1
WEE1
p53
p21
PP2A_B55
WIP1
CDK1_AurA
PLK1
CDC25
CycB_CDK1
Basin:
9.8 %
Trajectory
active
inactive

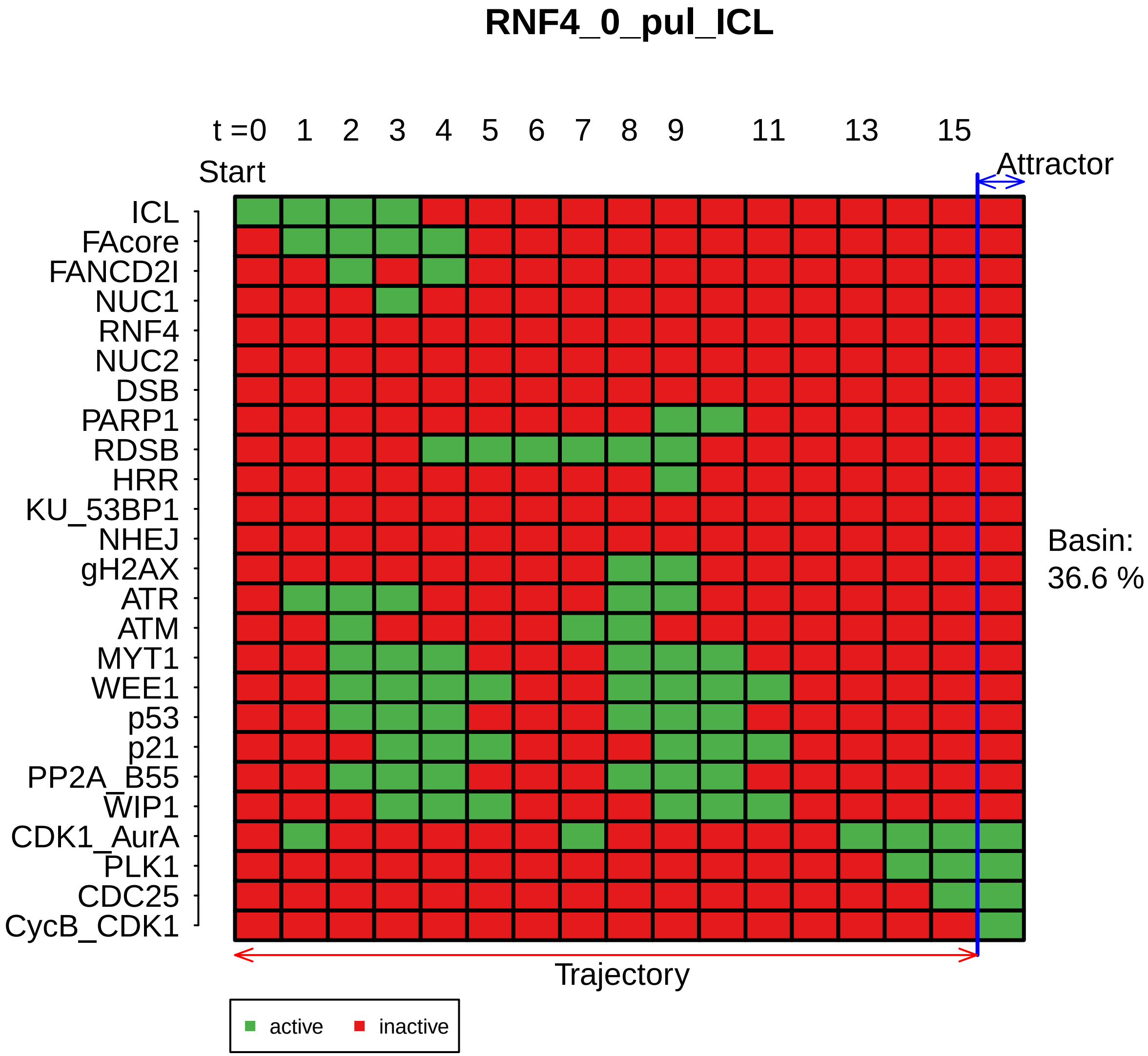
RNF4_0_pul_ICL
t =0 1 2 3 4 5 6 7 8 9 11 13 15
Start
Attractor
ICL
FAcore
FANCD2I
NUC1
RNF4
NUC2
DSB
PARP1
RDSB
HRR
KU_53BP1
NHEJ
gH2AX
ATR
ATM
MYT1
WEE1
p53
p21
PP2A_B55
WIP1
CDK1_AurA
PLK1
CDC25
CycB_CDK1
Basin:
36.6 %
Trajectory
active
inactive

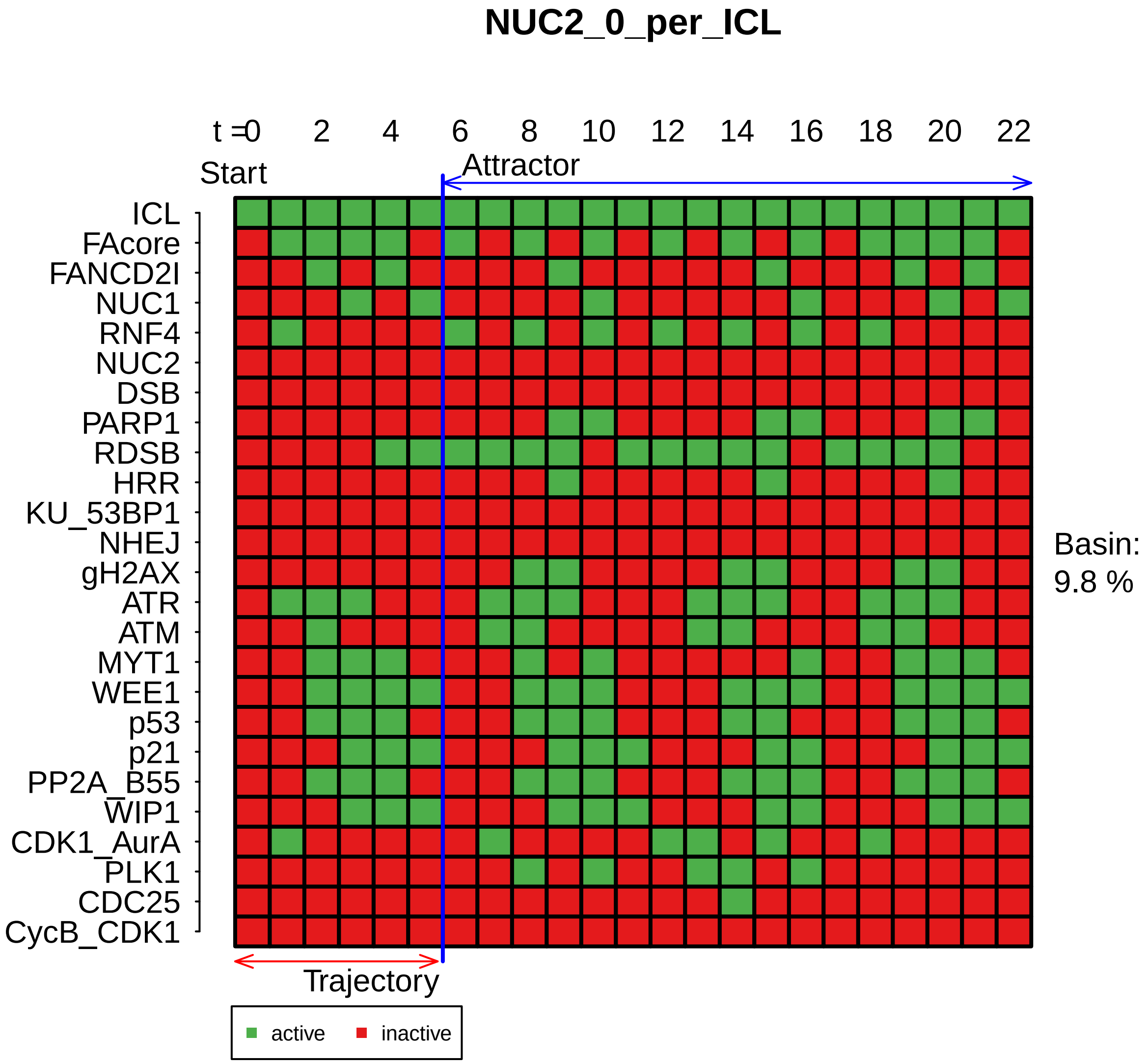

NUC2_0_per_ICL
t =0
2
4
6
8
10
12
14
16
18
20
22
Start
Attractor
ICL
FAcore
FANCD2I
NUC1
RNF4
NUC2
DSB
PARP1
RDSB
HRR
KU_53BP1
NHEJ
gH2AX
ATR
ATM
MYT1
WEE1
p53
p21
PP2A_B55
WIP1
CDK1_AurA
PLK1
CDC25
CycB_CDK1
Basin:
9.8 %
Trajectory
active
inactive

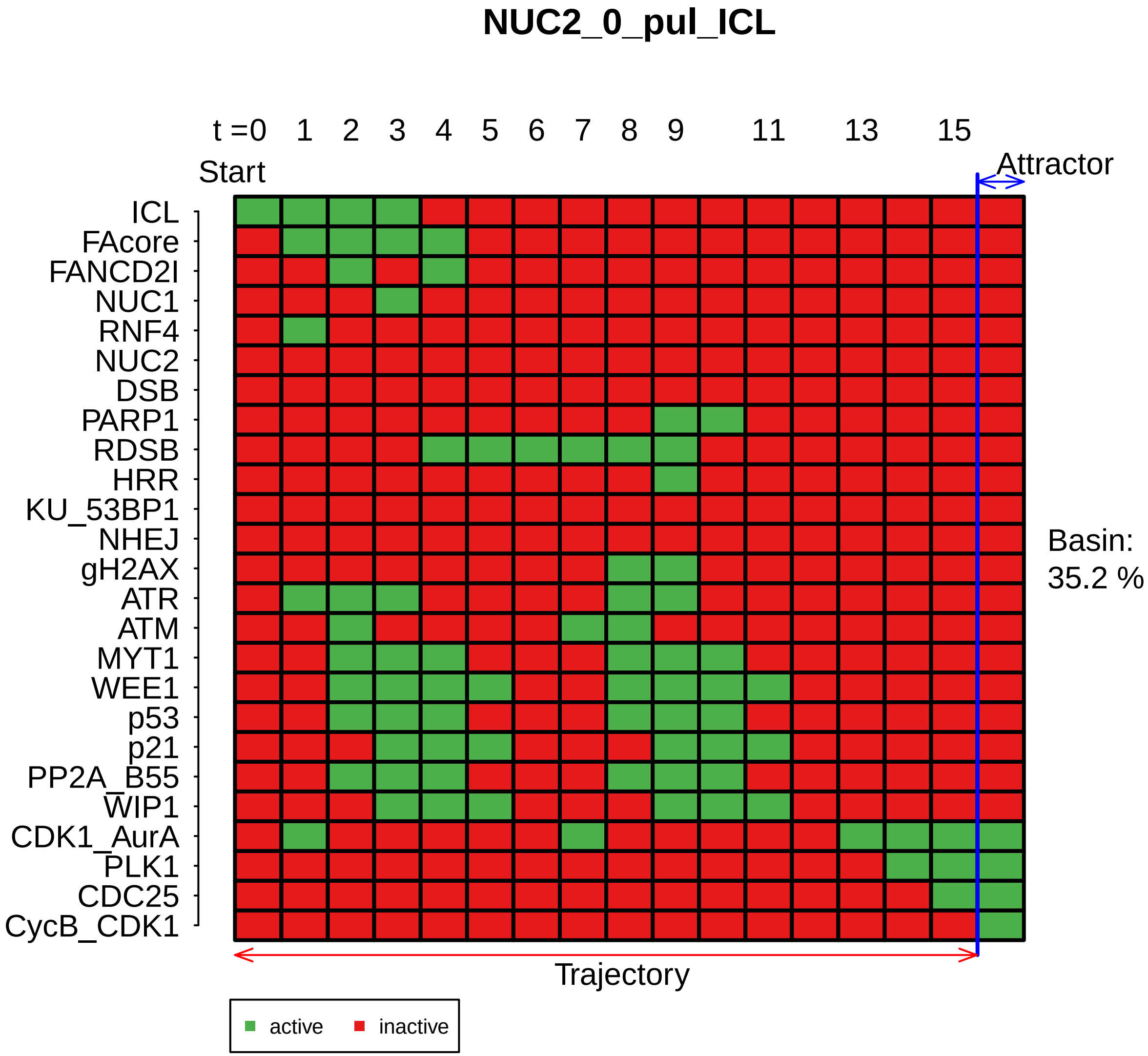

NUC2_0_pul_ICL
t =0
1
2
3
4
5
6
7
8
9
11
13
15
Start
Attractor
ICL
FAcore
FANCD2I
NUC1
RNF4
NUC2
DSB
PARP1
RDSB
HRR
KU_53BP1
NHEJ
gH2AX
ATR
ATM
MYT1
WEE1
p53
p21
PP2A_B55
WIP1
CDK1_AurA
PLK1
CDC25
CycB_CDK1
Basin:
35.2 %
Trajectory
active
inactive

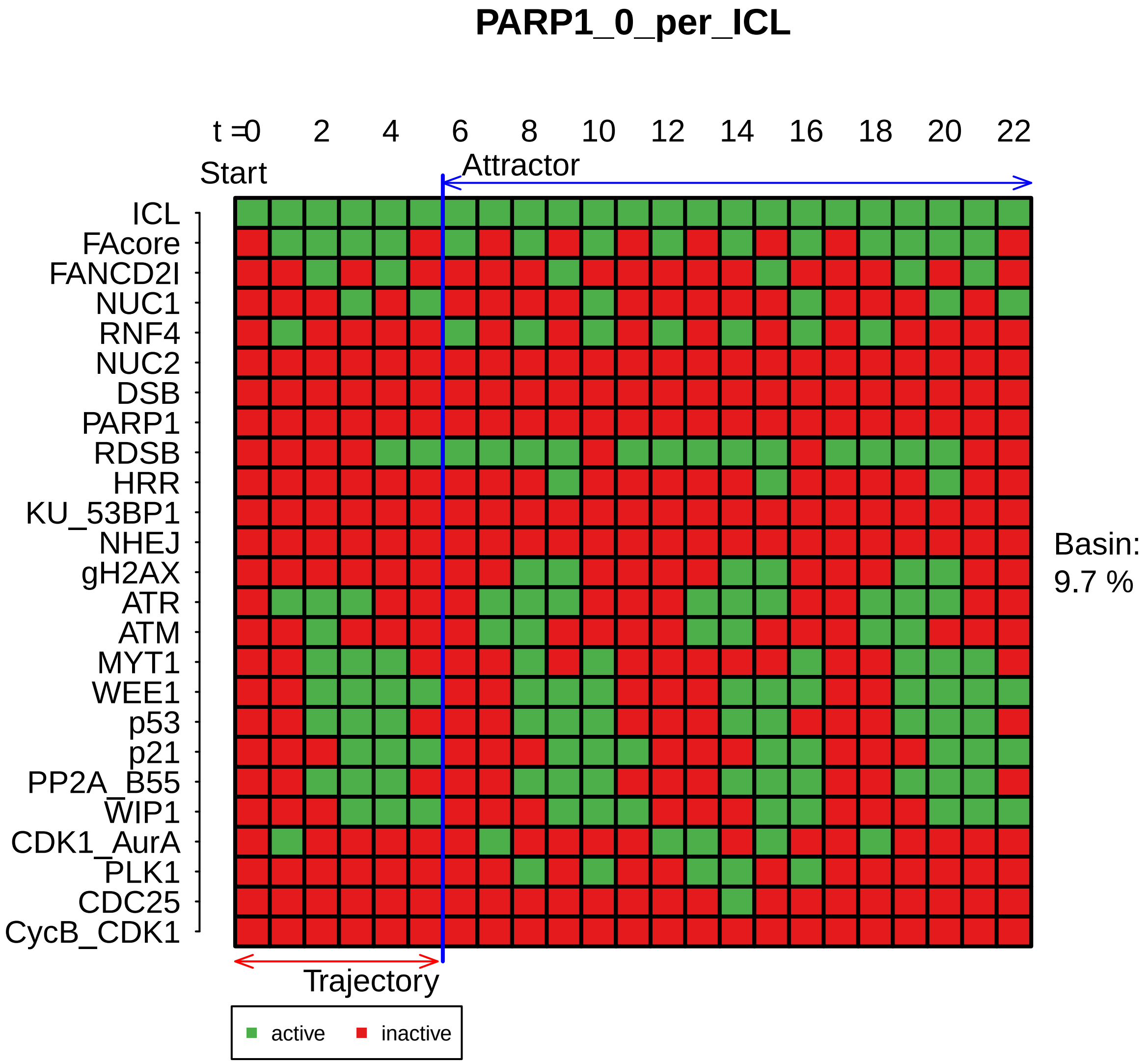

PARP1_0_per_ICL
t = 0
2
4
6
8
10
12
14
16
18
20
22
Start
Attractor
ICL
FAcore
FANCD2I
NUC1
RNF4
NUC2
DSB
PARP1
RDSB
HRR
KU_53BP1
NHEJ
gH2AX
ATR
ATM
MYT1
WEE1
p53
p21
PP2A_B55
WIP1
CDK1_AurA
PLK1
CDC25
CycB_CDK1
Basin:
9.7 %
Trajectory
active
inactive

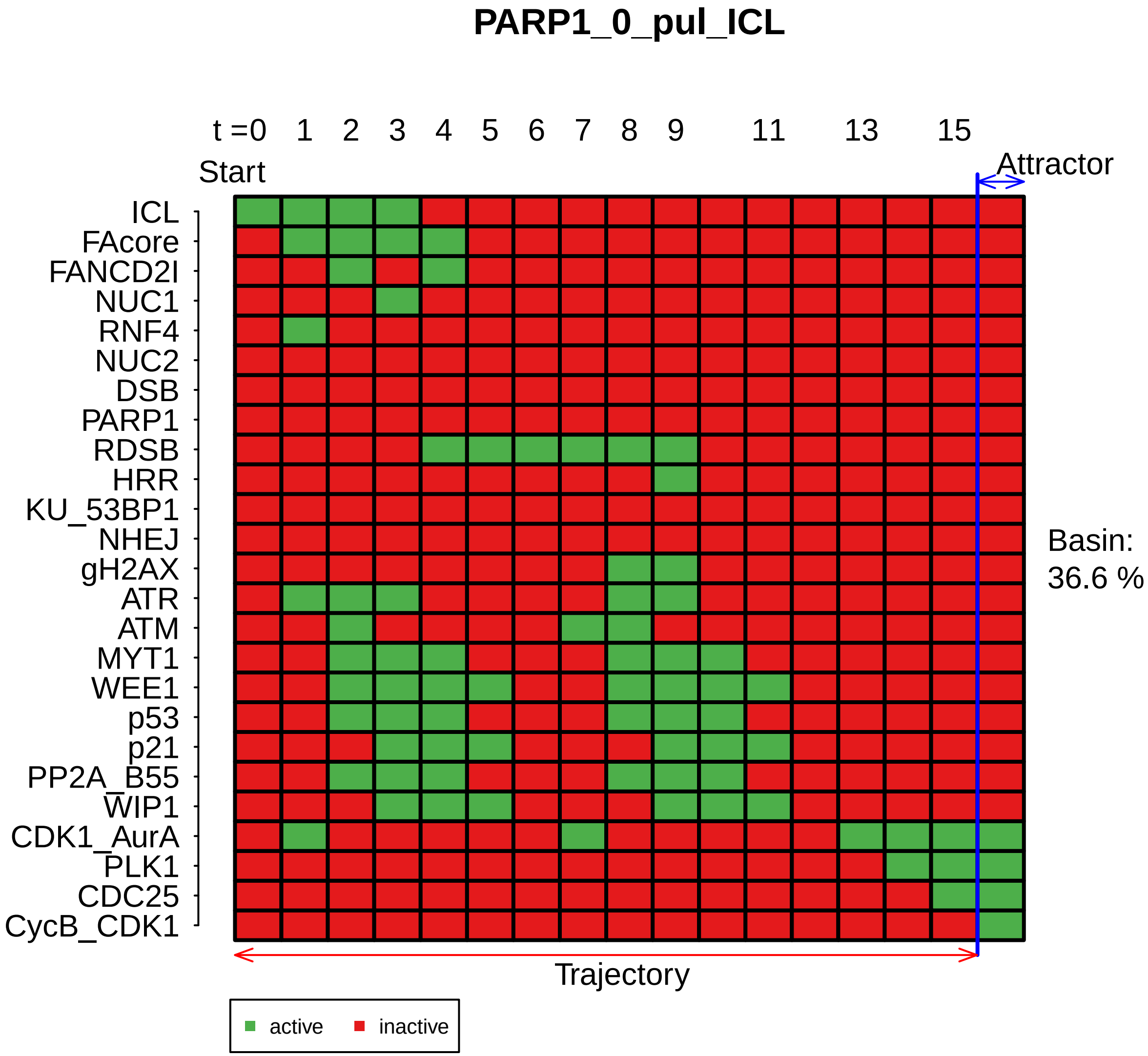
PARP1_0_pul_ICL
t =0
1
2
3
4
5
6
7
8
9
11
13
15
Start
Attractor
ICL
FAcore
FANCD2I
NUC1
RNF4
NUC2
DSB
PARP1
RDSB
HRR
KU_53BP1
NHEJ
gH2AX
ATR
ATM
MYT1
WEE1
p53
p21
PP2A_B55
WIP1
CDK1_AurA
PLK1
CDC25
CycB_CDK1
Basin:
36.6 %
Trajectory
active
inactive

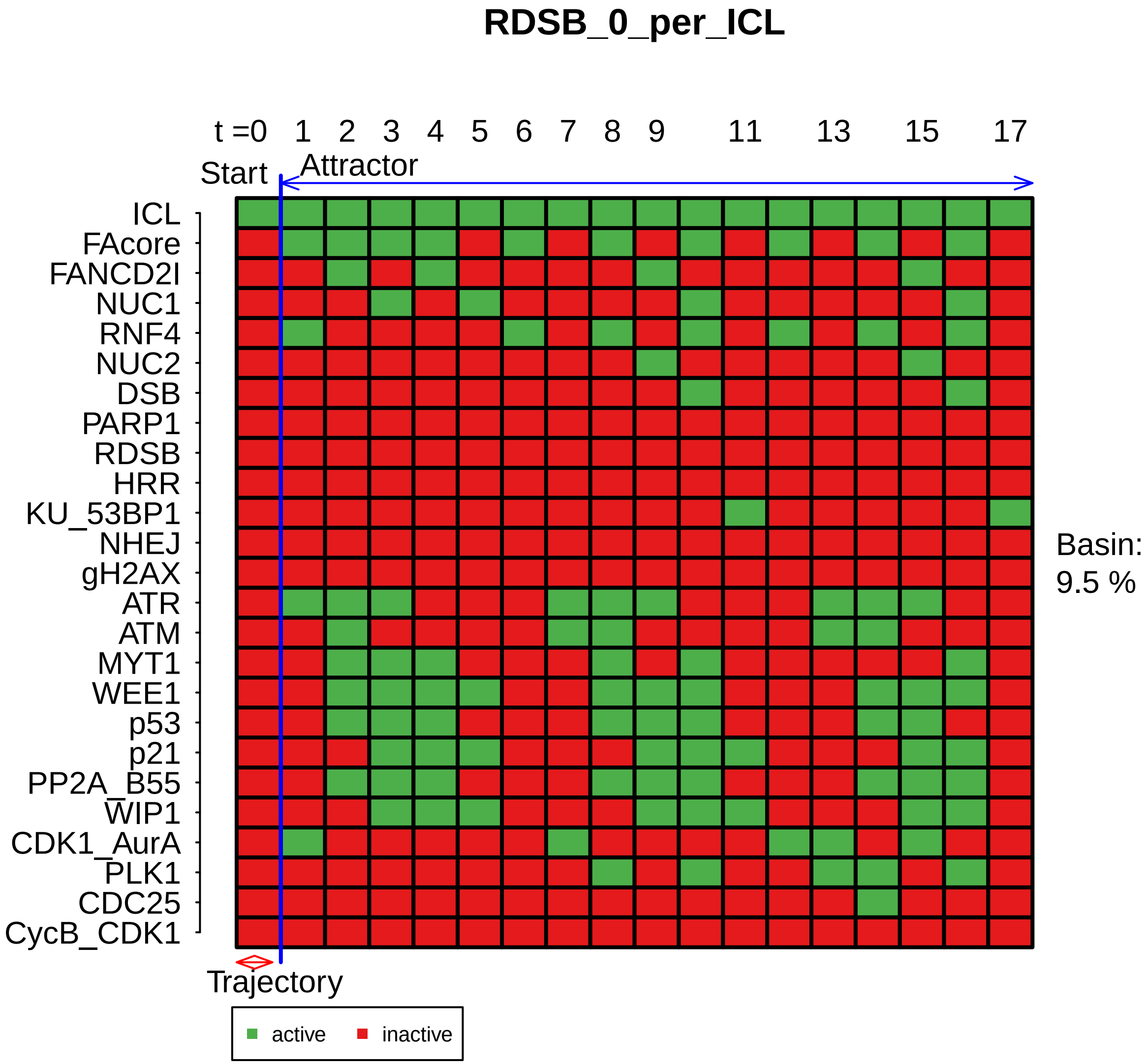

RDSB_0_per_ICL
t =0 1 2 3 4 5 6 7 8 9 11 13 15 17
Start
Attractor
ICL
FAcore
FANCD2I
NUC1
RNF4
NUC2
DSB
PARP1
RDSB
HRR
KU_53BP1
NHEJ
gH2AX
ATR
ATM
MYT1
WEE1
p53
p21
PP2A_B55
WIP1
CDK1_AurA
PLK1
CDC25
CycB_CDK1
Basin:
9.5 %
Trajectory
active
inactive

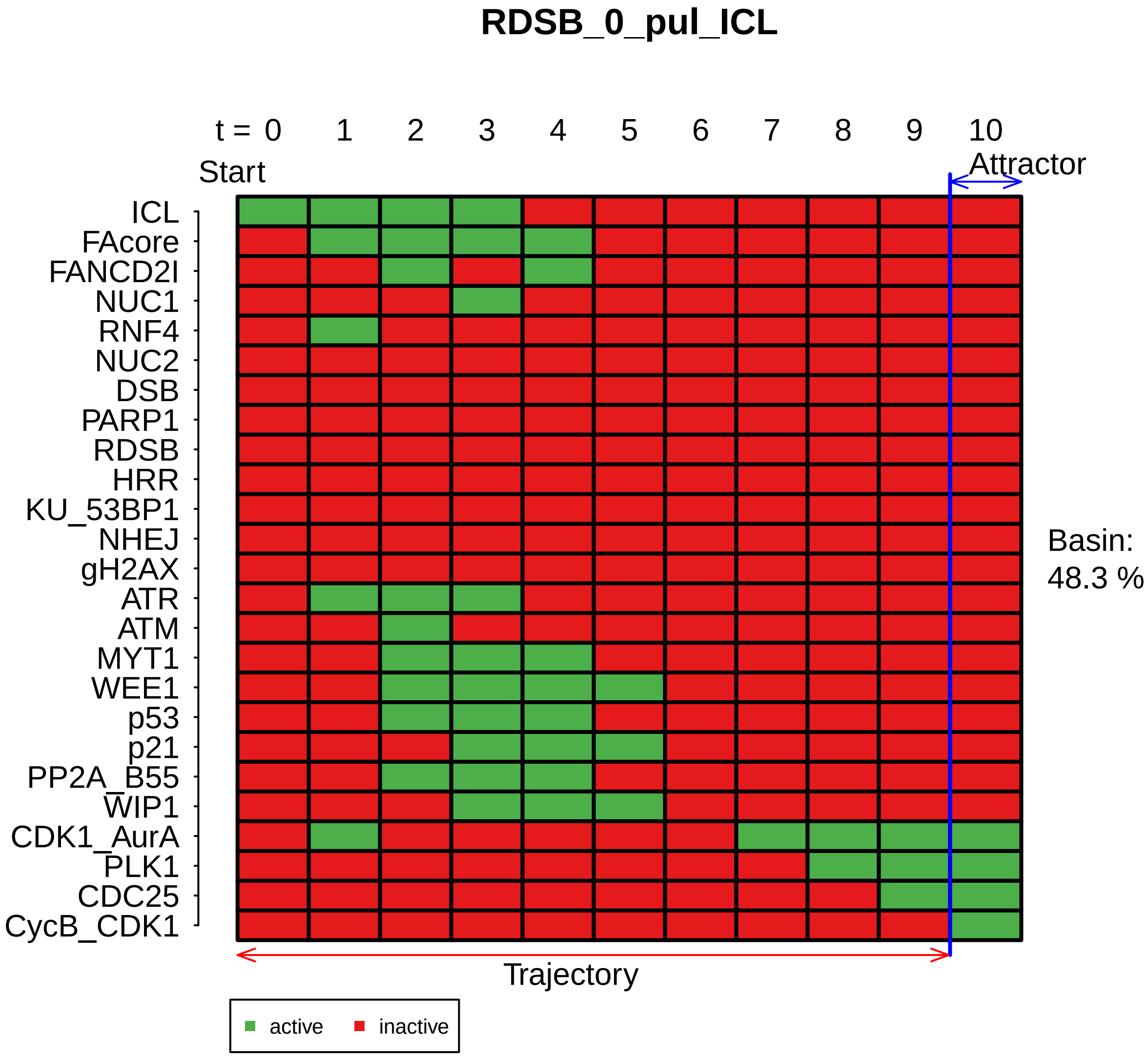
RDSB_0_pul_ICL
t = 0 1 2 3 4 5 6 7 8 9 10
Start
Attractor
ICL
FAcore
FANCD2I
NUC1
RNF4
NUC2
DSB
PARP1
RDSB
HRR
KU_53BP1
NHEJ
gH2AX
ATR
ATM
MYT1
WEE1
p53
p21
PP2A_B55
WIP1
CDK1_AurA
PLK1
CDC25
CycB_CDK1
Basin:
48.3 %
Trajectory
active
inactive

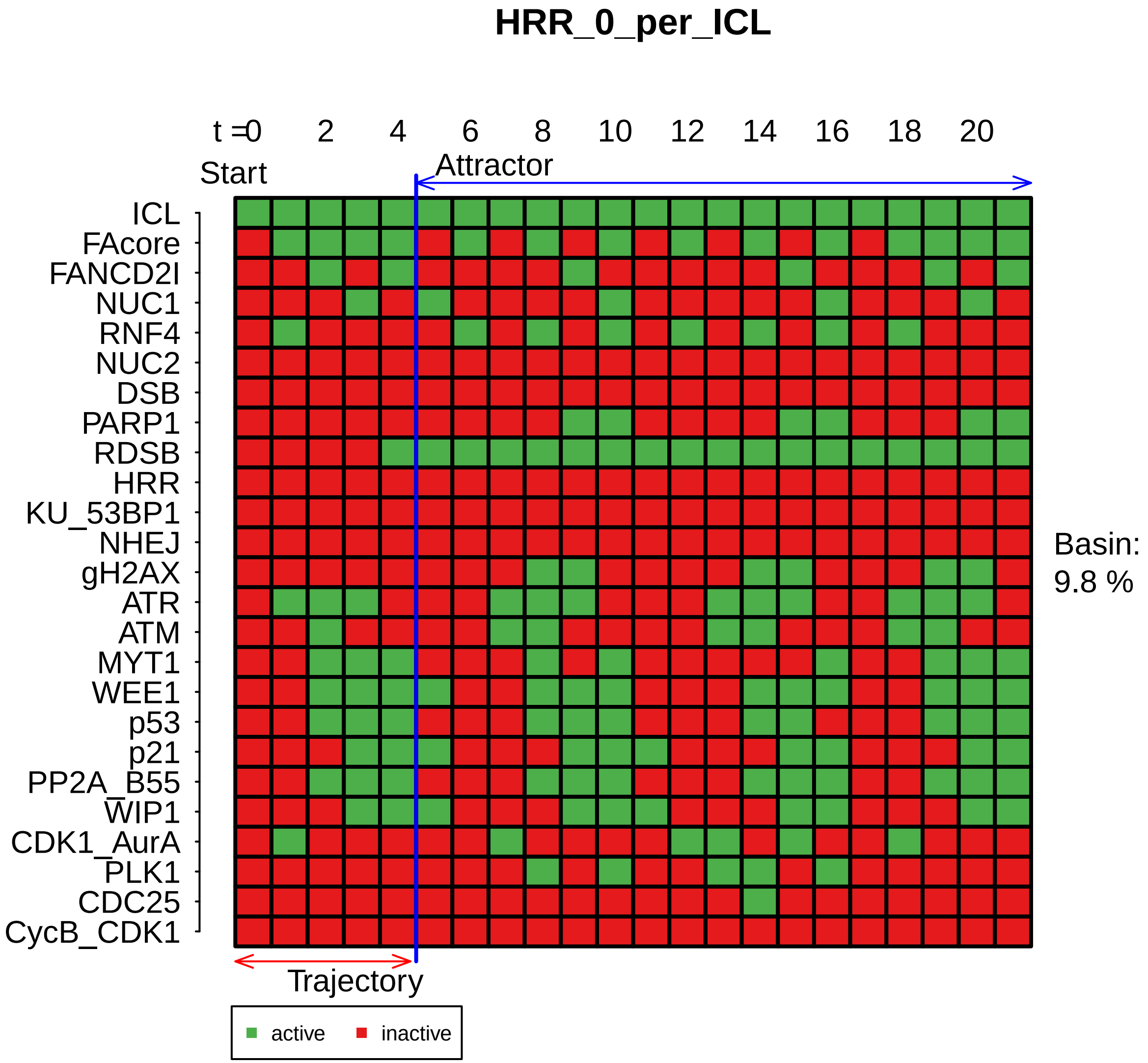

HRR_0_per_ICL
t = 0
2
4
6
8
10
12
14
16
18
20
Start
Attractor
ICL
FAcore
FANCD2I
NUC1
RNF4
NUC2
DSB
PARP1
RDSB
HRR
KU_53BP1
NHEJ
gH2AX
ATR
ATM
MYT1
WEE1
p53
p21
PP2A_B55
WIP1
CDK1_AurA
PLK1
CDC25
CycB_CDK1
Basin:
9.8 %
Trajectory
active
inactive

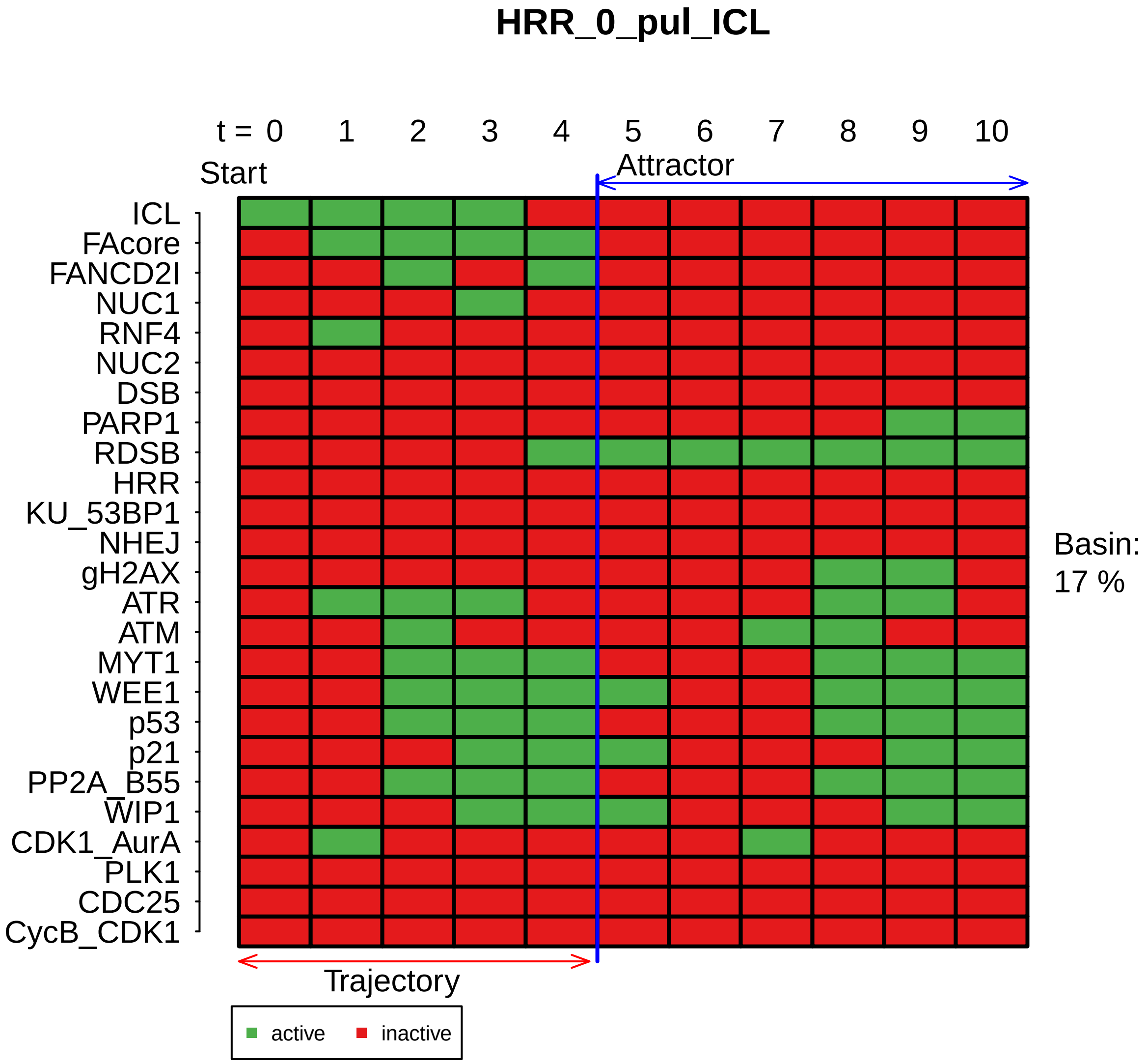
HRR_0_pul_ICL
t = 0
1
2
3
4
5
6
7
8
9
10
Start
Attractor
ICL
FAcore
FANCD2I
NUC1
RNF4
NUC2
DSB
PARP1
RDSB
HRR
KU_53BP1
NHEJ
gH2AX
ATR
ATM
MYT1
WEE1
p53
p21
PP2A_B55
WIP1
CDK1_AurA
PLK1
CDC25
CycB_CDK1
Basin:
17 %
Trajectory
active
inactive

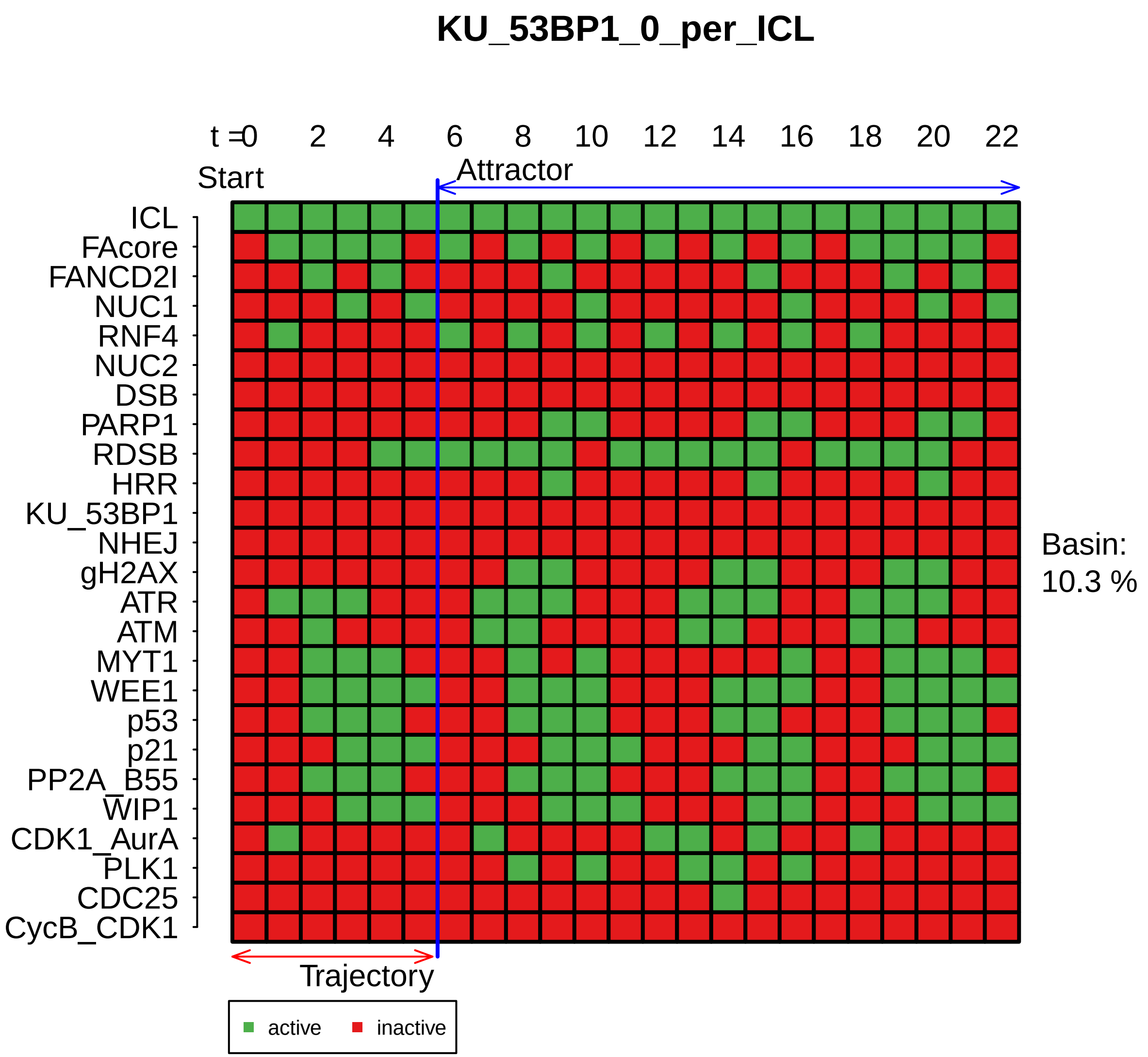

KU_53BP1_0_per_ICL
t = 0
2
4
6
8
10
12
14
16
18
20
22
Start
Attractor
ICL
FAcore
FANCD2I
NUC1
RNF4
NUC2
DSB
PARP1
RDSB
HRR
KU_53BP1
NHEJ
gH2AX
ATR
ATM
MYT1
WEE1
p53
p21
PP2A_B55
WIP1
CDK1_AurA
PLK1
CDC25
CycB_CDK1
Basin:
10.3 %
Trajectory
active
inactive

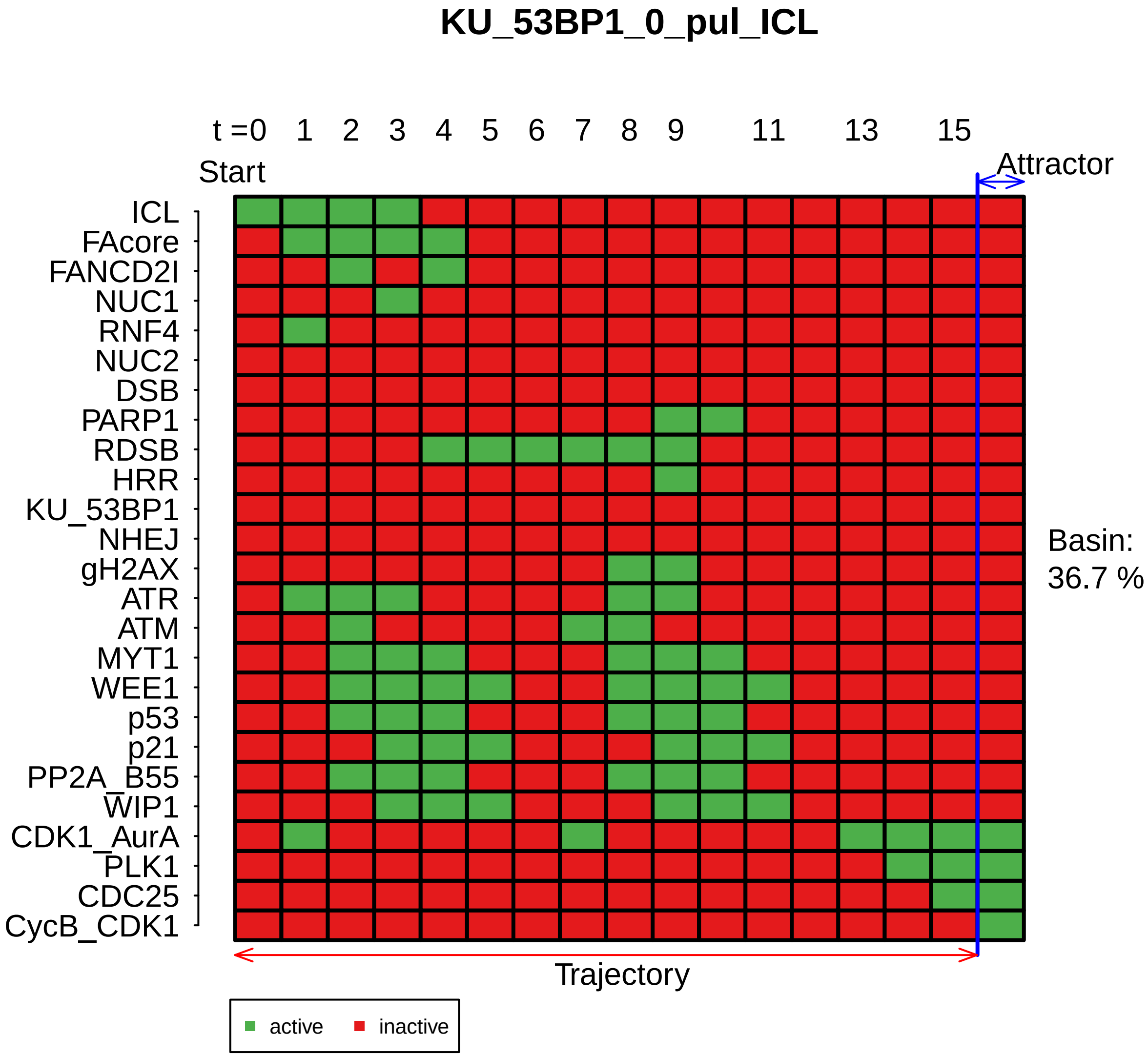

KU_53BP1_0_pul_ICL
t =0 1 2 3 4 5 6 7 8 9 11 13 15
Start
Attractor
ICL
FAcore
FANCD2I
NUC1
RNF4
NUC2
DSB
PARP1
RDSB
HRR
KU_53BP1
NHEJ
gH2AX
ATR
ATM
MYT1
WEE1
p53
p21
PP2A_B55
WIP1
CDK1_AurA
PLK1
CDC25
CycB_CDK1
Basin:
36.7 %
Trajectory
active
inactive

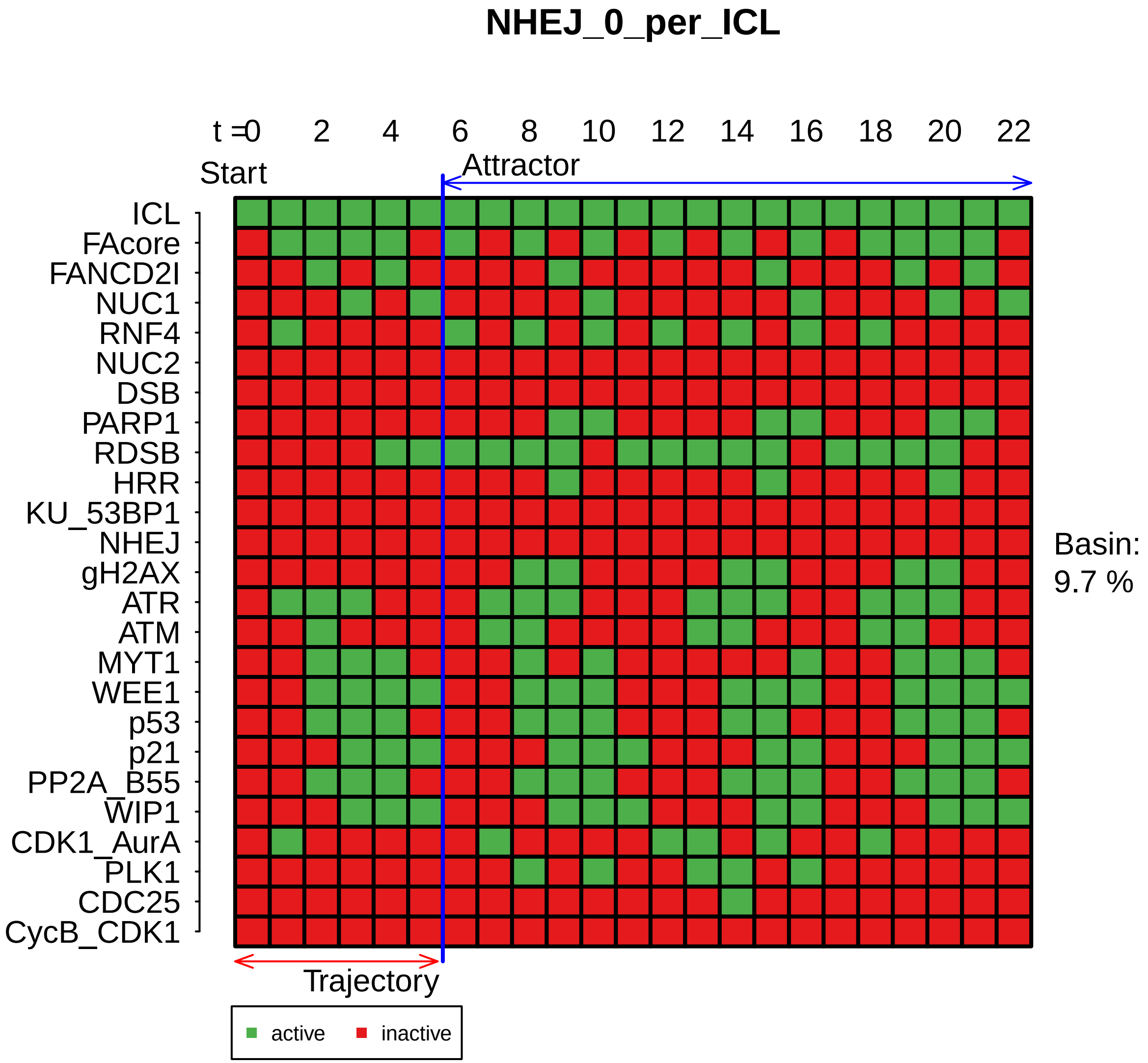
NHEJ_0_per_ICL
t =0
2
4
6
8
10
12
14
16
18
20
22
Start
Attractor
ICL
FAcore
FANCD2I
NUC1
RNF4
NUC2
DSB
PARP1
RDSB
HRR
KU_53BP1
NHEJ
gH2AX
ATR
ATM
MYT1
WEE1
p53
p21
PP2A_B55
WIP1
CDK1_AurA
PLK1
CDC25
CycB_CDK1
Basin:
9.7 %
Trajectory
active
inactive

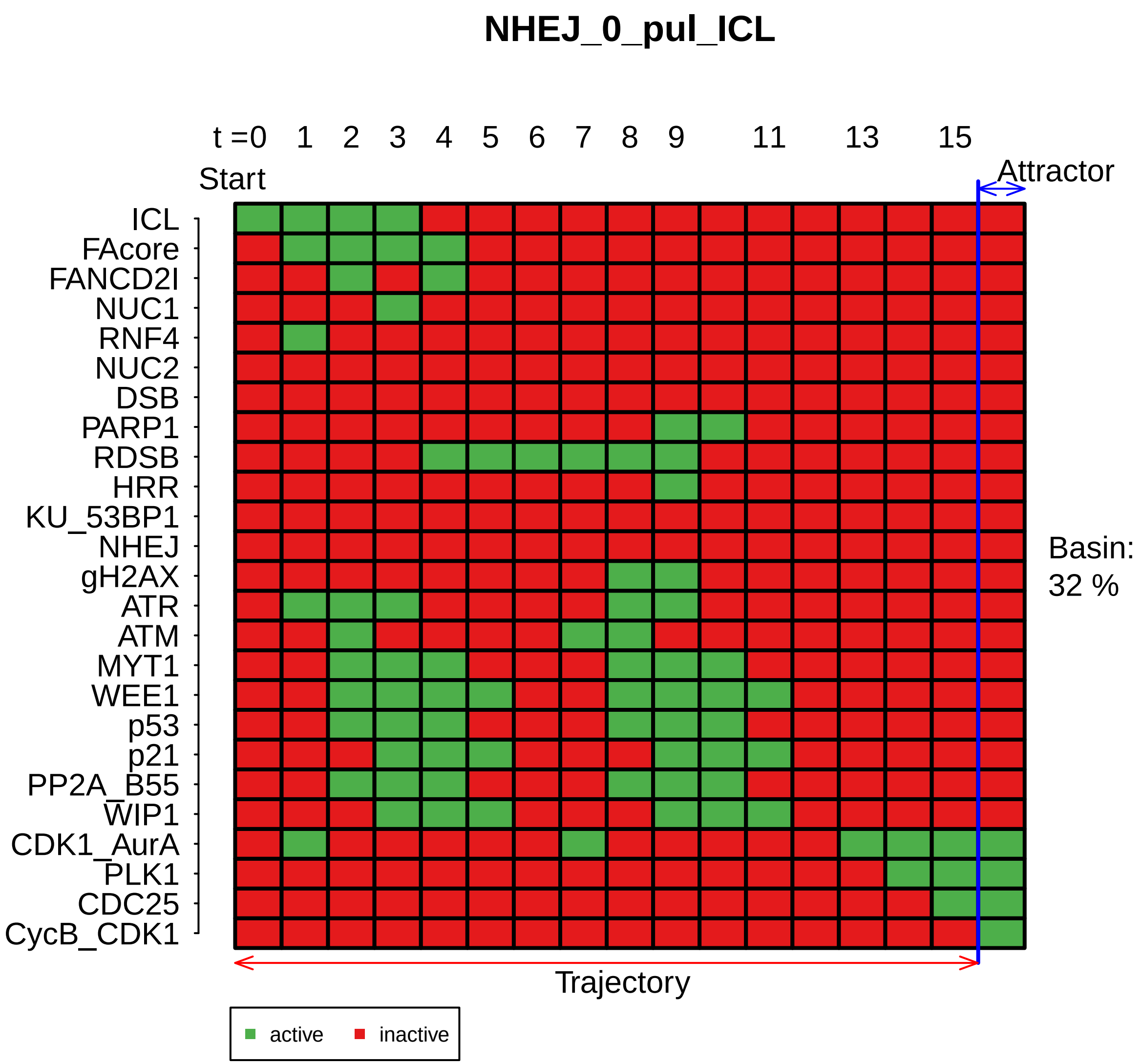
NHEJ_0_pul_ICL
t =0 1 2 3 4 5 6 7 8 9 11 13 15
Start
Attractor
ICL
FAcore
FANCD2I
NUC1
RNF4
NUC2
DSB
PARP1
RDSB
HRR
KU_53BP1
NHEJ
gH2AX
ATR
ATM
MYT1
WEE1
p53
p21
PP2A_B55
WIP1
CDK1_AurA
PLK1
CDC25
CycB_CDK1
Basin:
32 %
Trajectory
active
inactive

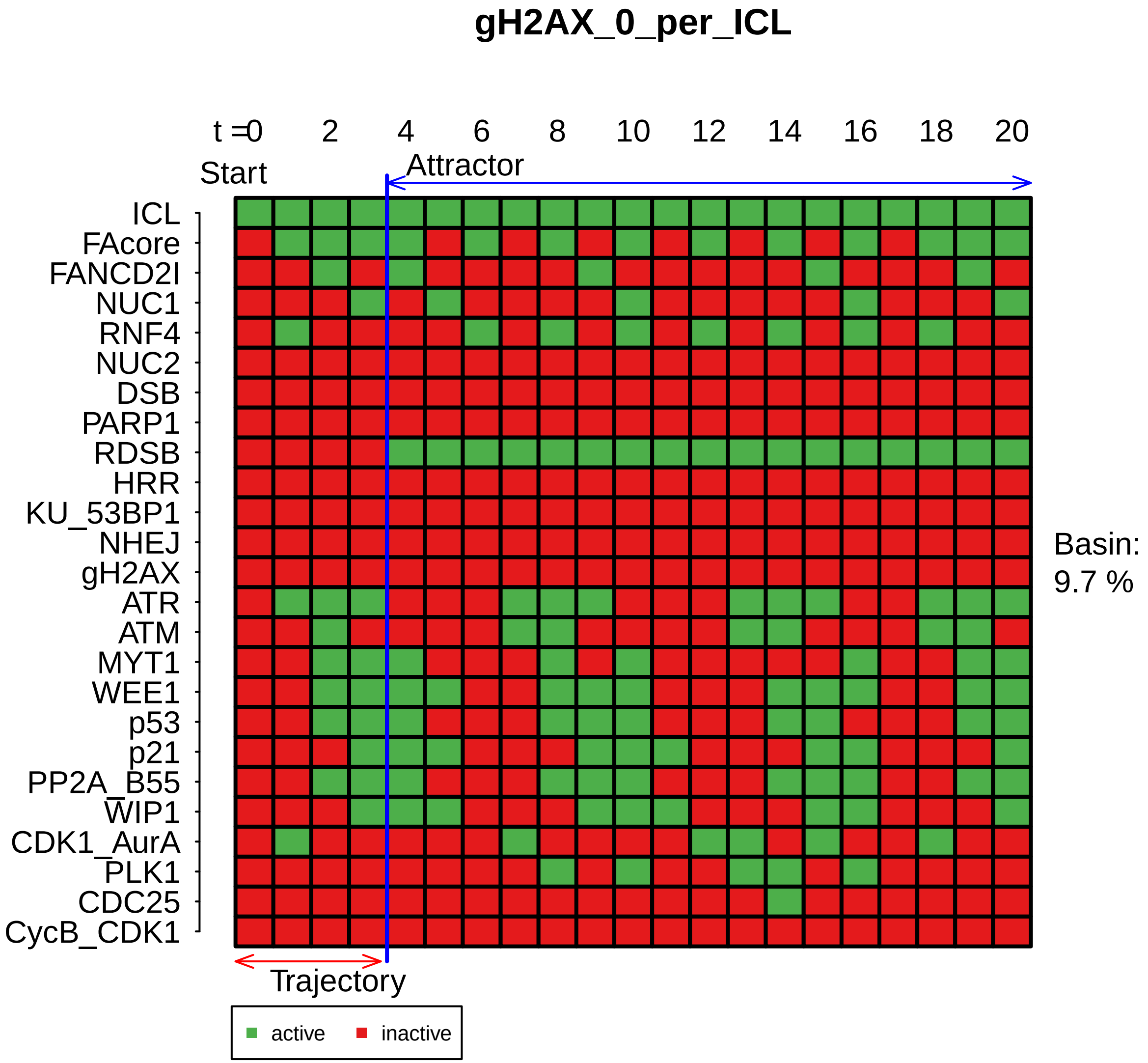

gH2AX_0_per_ICL
t = 0 2 4 6 8 10 12 14 16 18 20
Start
Attractor
ICL
FAcore
FANCD2I
NUC1
RNF4
NUC2
DSB
PARP1
RDSB
HRR
KU_53BP1
NHEJ
gH2AX
ATR
ATM
MYT1
WEE1
p53
p21
PP2A_B55
WIP1
CDK1_AurA
PLK1
CDC25
CycB_CDK1
Basin:
9.7 %
Trajectory
active
inactive

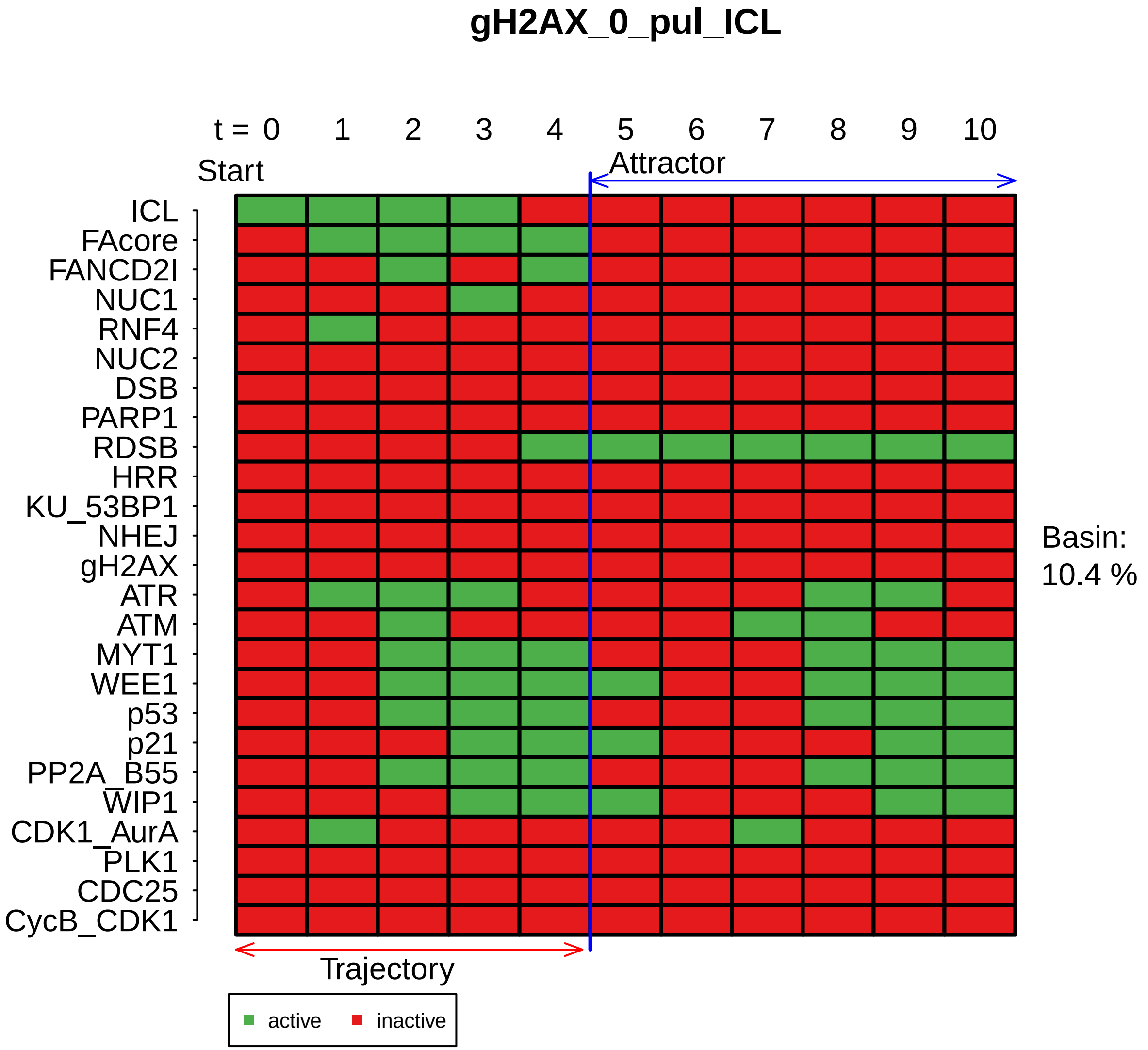

gH2AX_0_pul_ICL
t = 0
1
2
3
4
5
6
7
8
9
10
Start
Attractor
ICL
FAcore
FANCD2I
NUC1
RNF4
NUC2
DSB
PARP1
RDSB
HRR
KU_53BP1
NHEJ
gH2AX
ATR
ATM
MYT1
WEE1
p53
p21
PP2A_B55
WIP1
CDK1_AurA
PLK1
CDC25
CycB_CDK1
Basin:
10.4 %
Trajectory
active
inactive

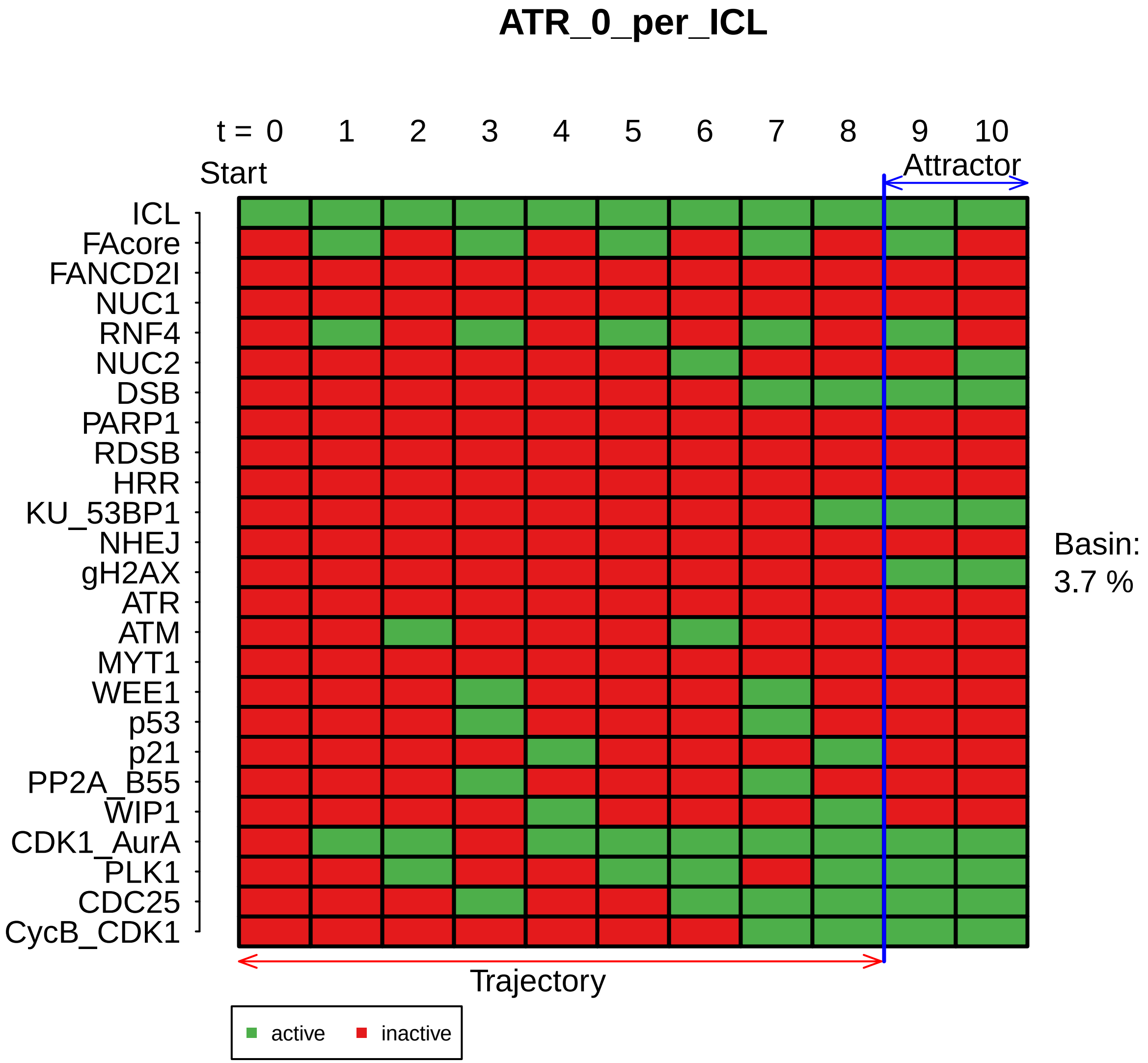
ATR_0_per_ICL
t = 0
1
2
3
4
5
6
7
8
9
10
Start
Attractor
ICL
FAcore
FANCD2I
NUC1
RNF4
NUC2
DSB
PARP1
RDSB
HRR
KU_53BP1
NHEJ
gH2AX
ATR
ATM
MYT1
WEE1
p53
p21
PP2A_B55
WIP1
CDK1_AurA
PLK1
CDC25
CycB_CDK1
Basin:
3.7 %
Trajectory
active
inactive

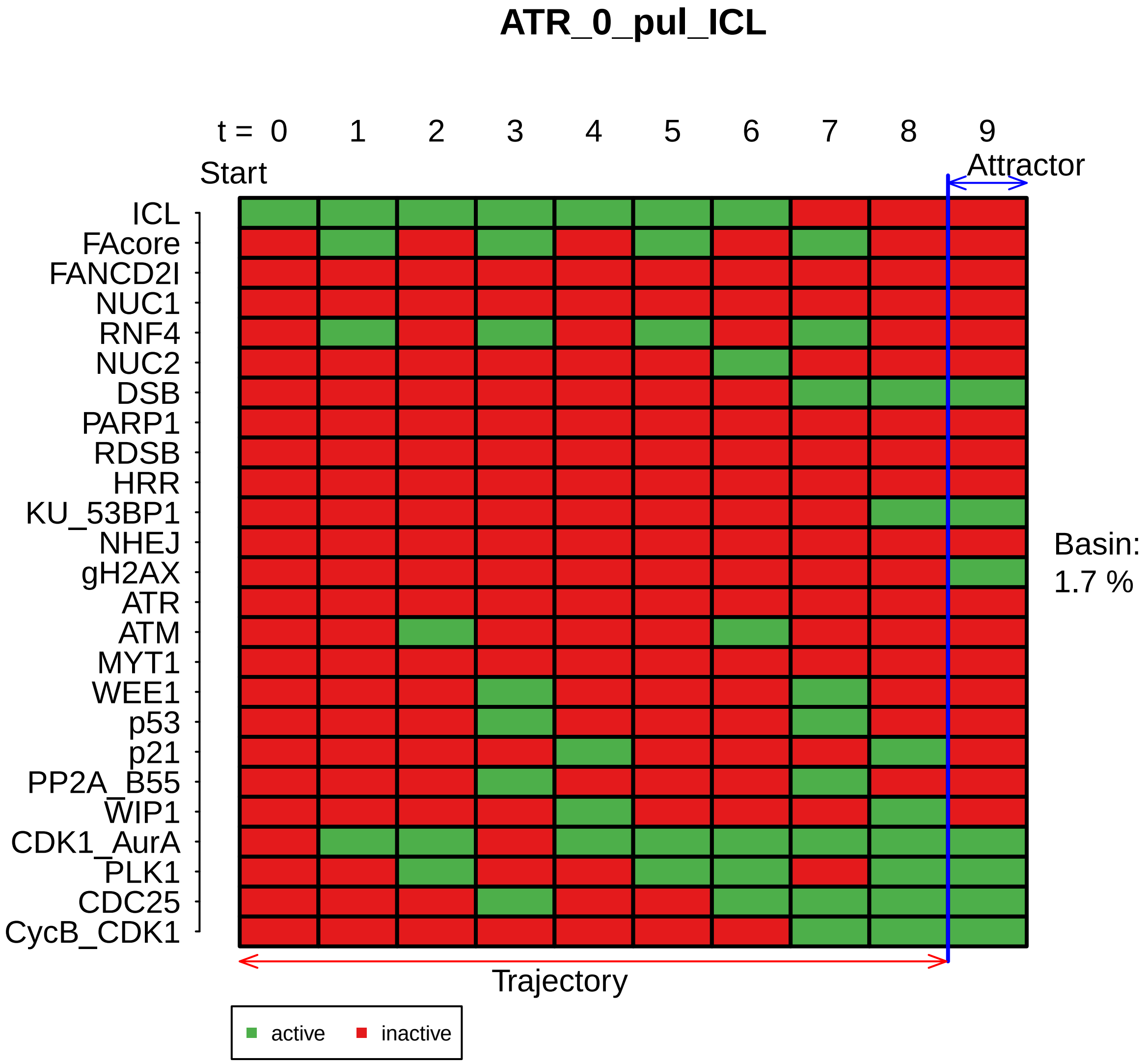

ATR_0_pul_ICL
t = 0
1
2
3
4
5
6
7
8
9
Start
Attractor
ICL
FAcore
FANCD2I
NUC1
RNF4
NUC2
DSB
PARP1
RDSB
HRR
KU_53BP1
NHEJ
gH2AX
ATR
ATM
MYT1
WEE1
p53
p21
PP2A_B55
WIP1
CDK1_AurA
PLK1
CDC25
CycB_CDK1
Basin:
1.7 %
Trajectory
active
inactive

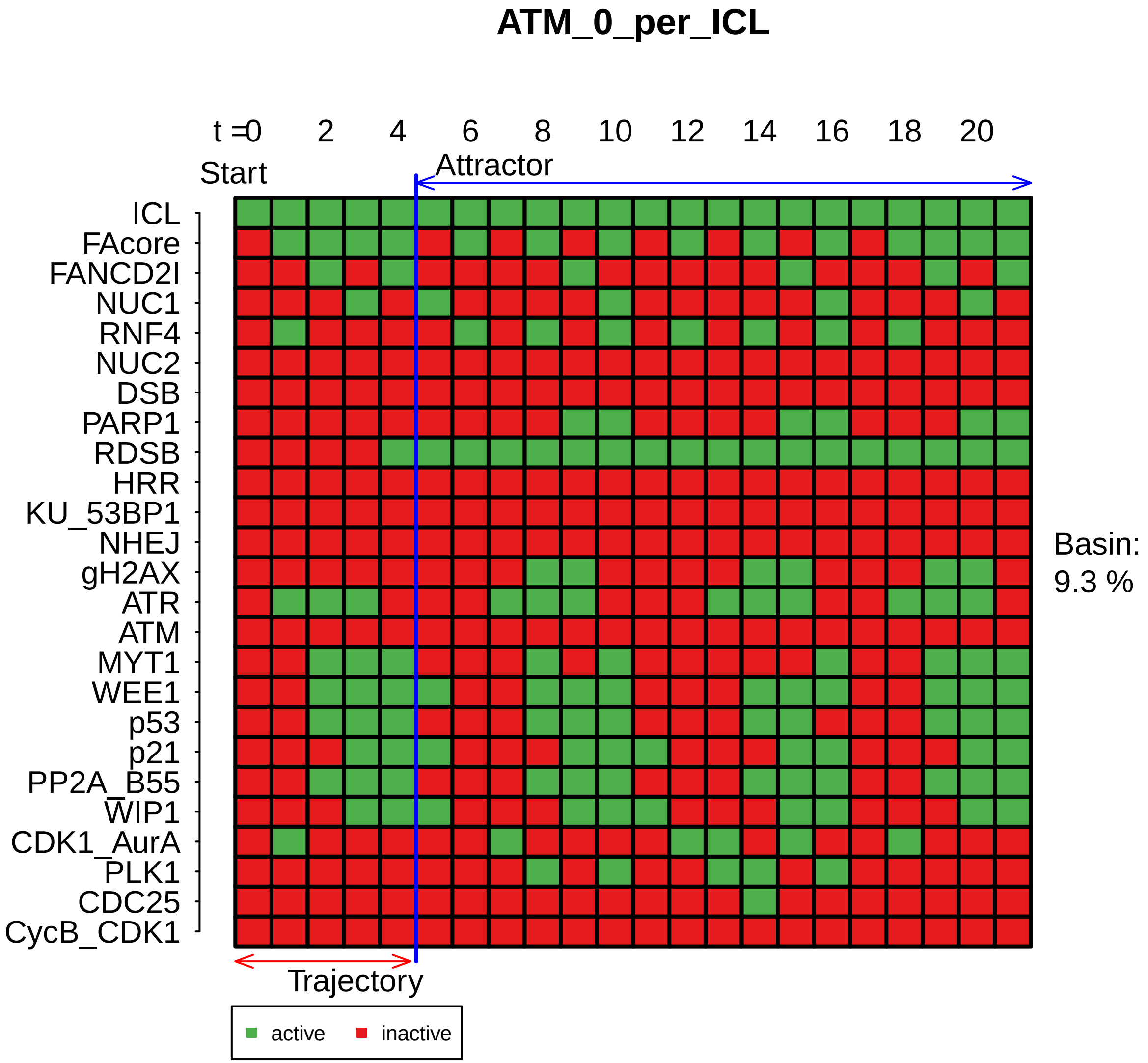

ATM_0_per_ICL
t = 0 2 4 6 8 10 12 14 16 18 20
Start
Attractor
ICL
FAcore
FANCD2I
NUC1
RNF4
NUC2
DSB
PARP1
RDSB
HRR
KU_53BP1
NHEJ
gH2AX
ATR
ATM
MYT1
WEE1
p53
p21
PP2A_B55
WIP1
CDK1_AurA
PLK1
CDC25
CycB_CDK1
Basin:
9.3 %
Trajectory
active
inactive

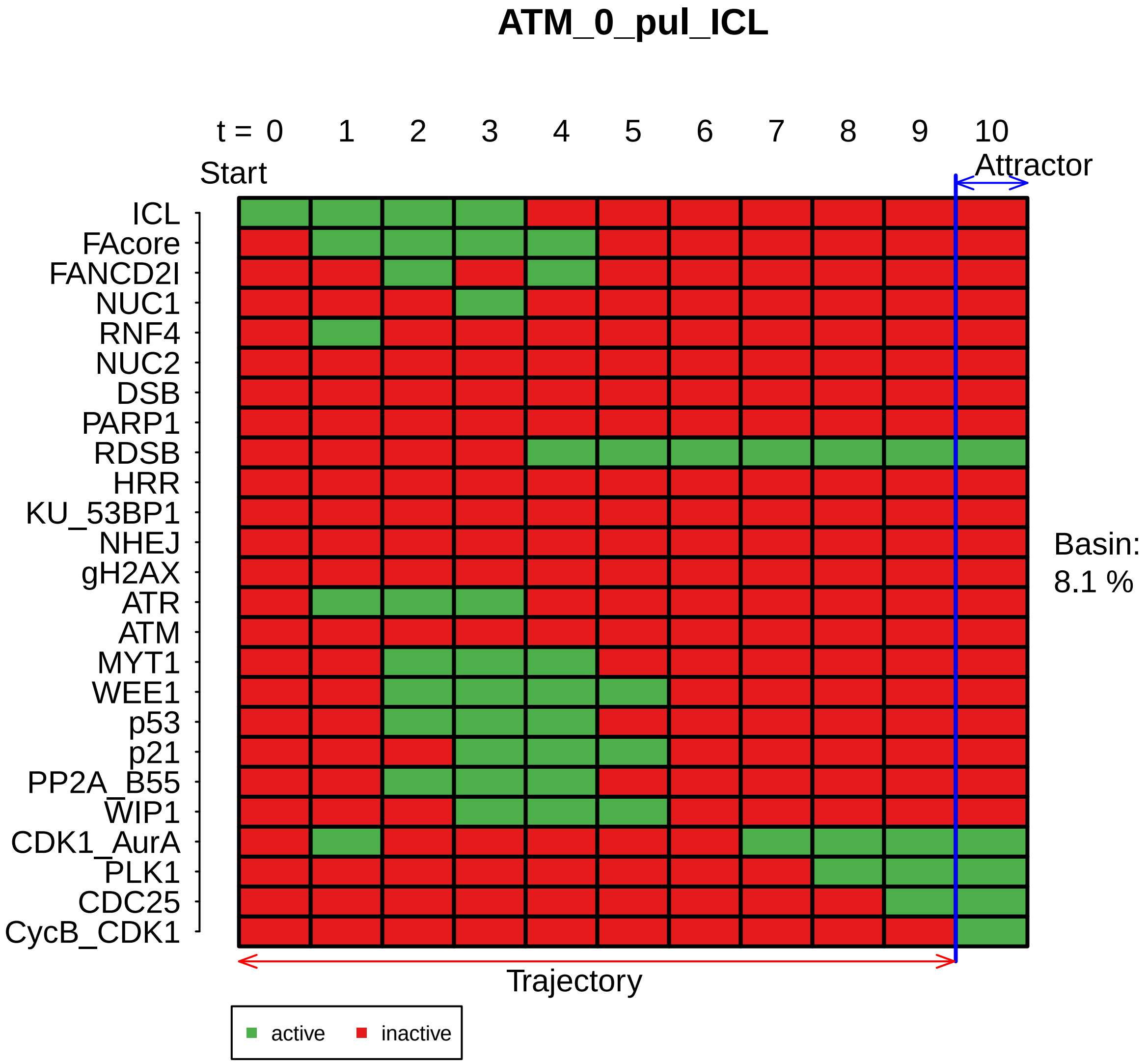
ATM_0_pul_ICL
t = 0 1 2 3 4 5 6 7 8 9 10
Start
Attractor
ICL
FAcore
FANCD2I
NUC1
RNF4
NUC2
DSB
PARP1
RDSB
HRR
KU_53BP1
NHEJ
gH2AX
ATR
ATM
MYT1
WEE1
p53
p21
PP2A_B55
WIP1
CDK1_AurA
PLK1
CDC25
CycB_CDK1
Basin:
8.1 %
Trajectory
active
inactive

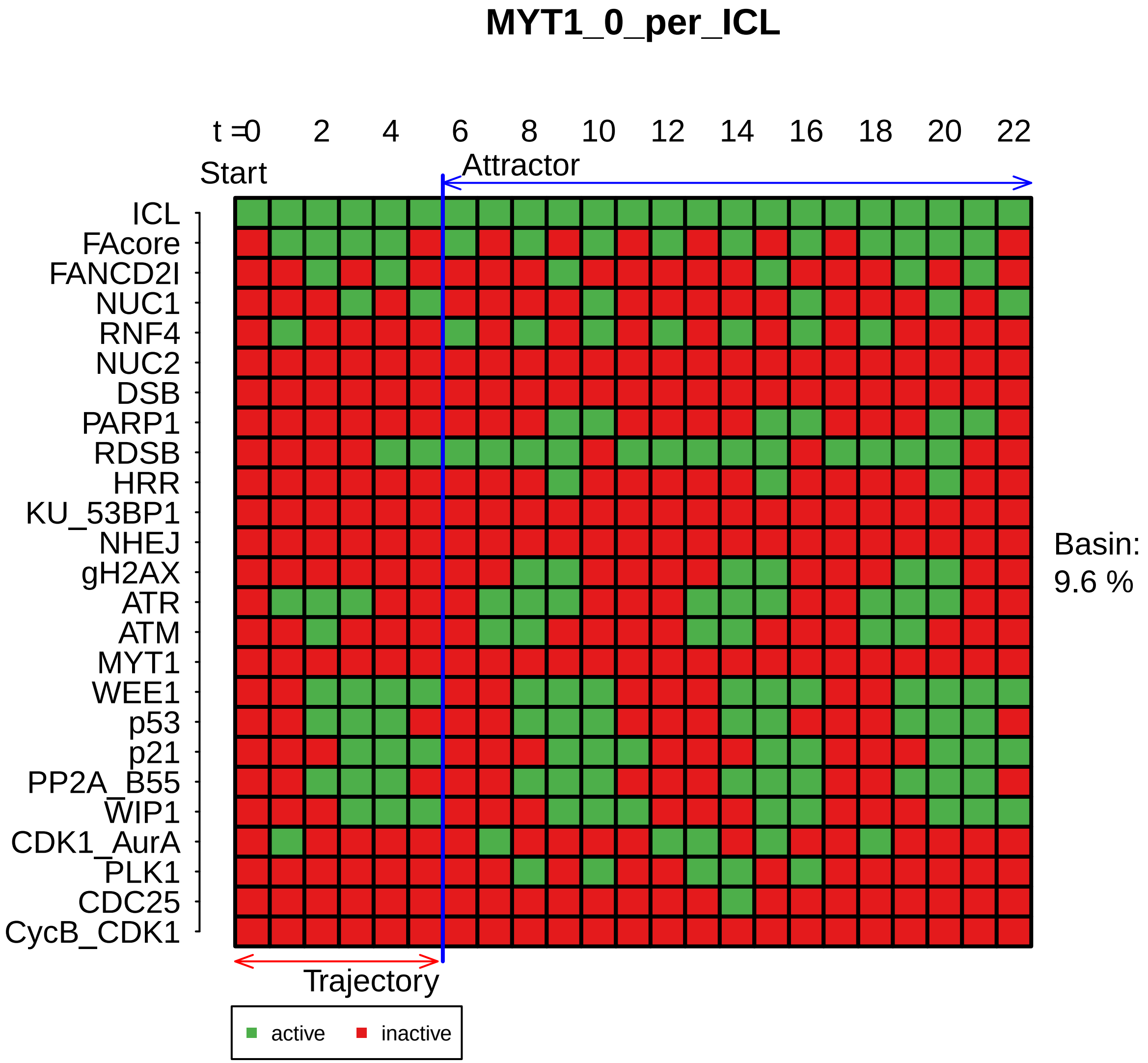
MYT1_0_per_ICL
t = 0 2 4 6 8 10 12 14 16 18 20 22
Start
Attractor
ICL
FAcore
FANCD2I
NUC1
RNF4
NUC2
DSB
PARP1
RDSB
HRR
KU_53BP1
NHEJ
gH2AX
ATR
ATM
MYT1
WEE1
p53
p21
PP2A_B55
WIP1
CDK1_AurA
PLK1
CDC25
CycB_CDK1
Basin:
9.6 %
Trajectory
active
inactive

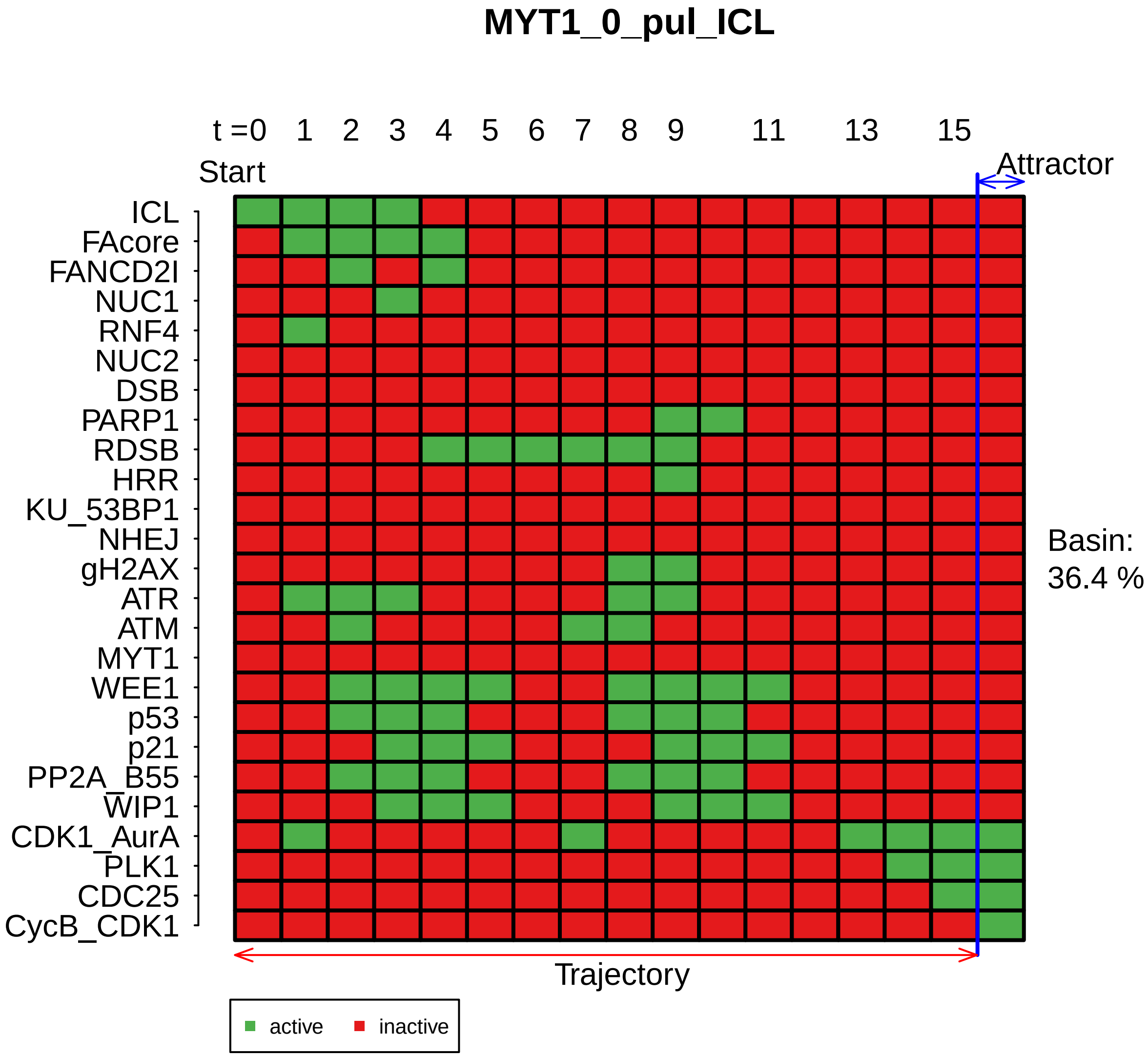

MYT1_0_pul_ICL
t =0
1
2
3
4
5
6
7
8
9
11
13
15
Start
Attractor
ICL
FAcore
FANCD2I
NUC1
RNF4
NUC2
DSB
PARP1
RDSB
HRR
KU_53BP1
NHEJ
gH2AX
ATR
ATM
MYT1
WEE1
p53
p21
PP2A_B55
WIP1
CDK1_AurA
PLK1
CDC25
CycB_CDK1
Basin:
36.4 %
Trajectory
active
inactive

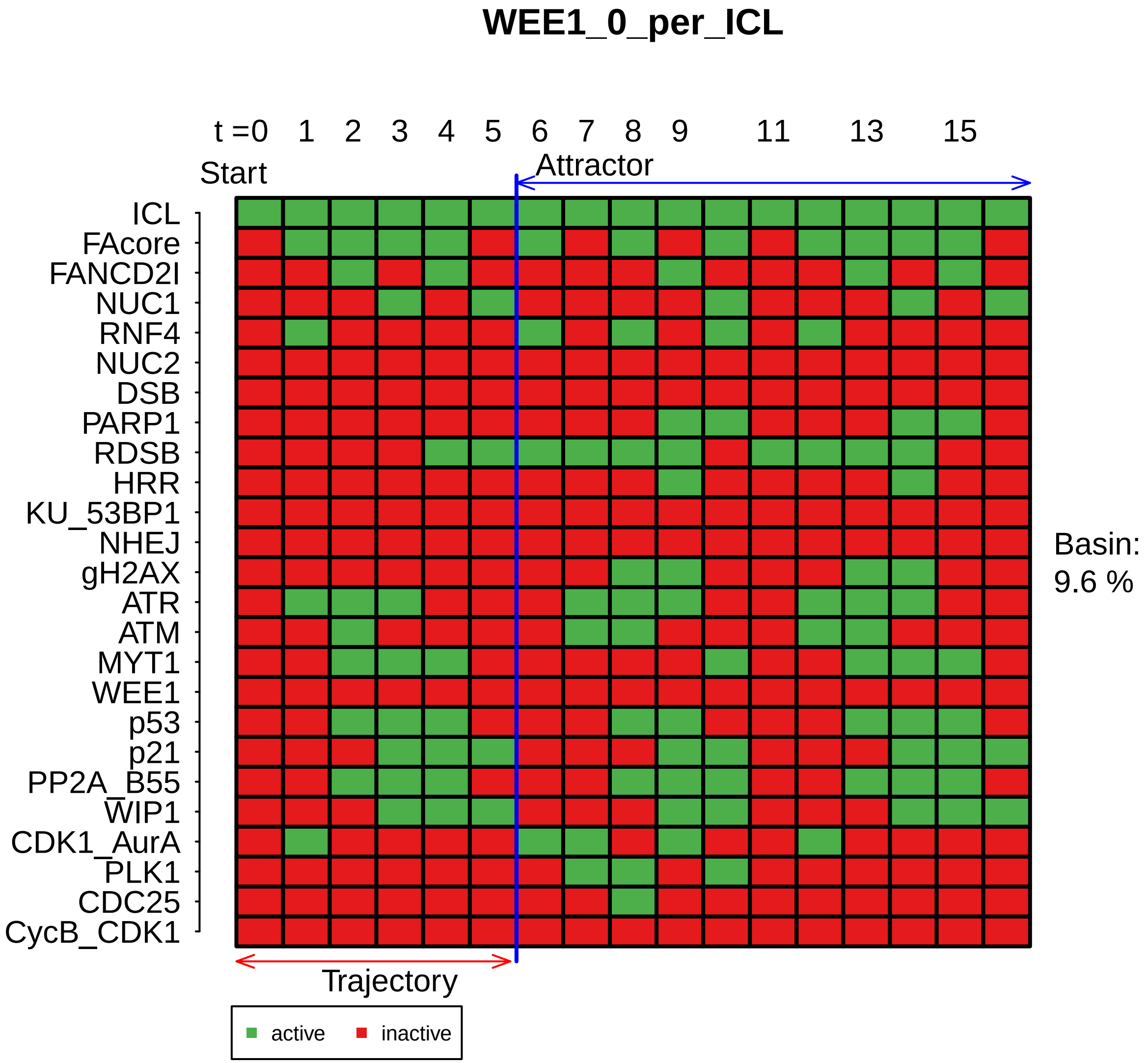

WEE1_0_per_ICL
t =0 1 2 3 4 5 6 7 8 9 11 13 15
Start
Attractor
ICL
FAcore
FANCD2I
NUC1
RNF4
NUC2
DSB
PARP1
RDSB
HRR
KU_53BP1
NHEJ
gH2AX
ATR
ATM
MYT1
WEE1
p53
p21
PP2A_B55
WIP1
CDK1_AurA
PLK1
CDC25
CycB_CDK1
Basin:
9.6 %
Trajectory
active
inactive

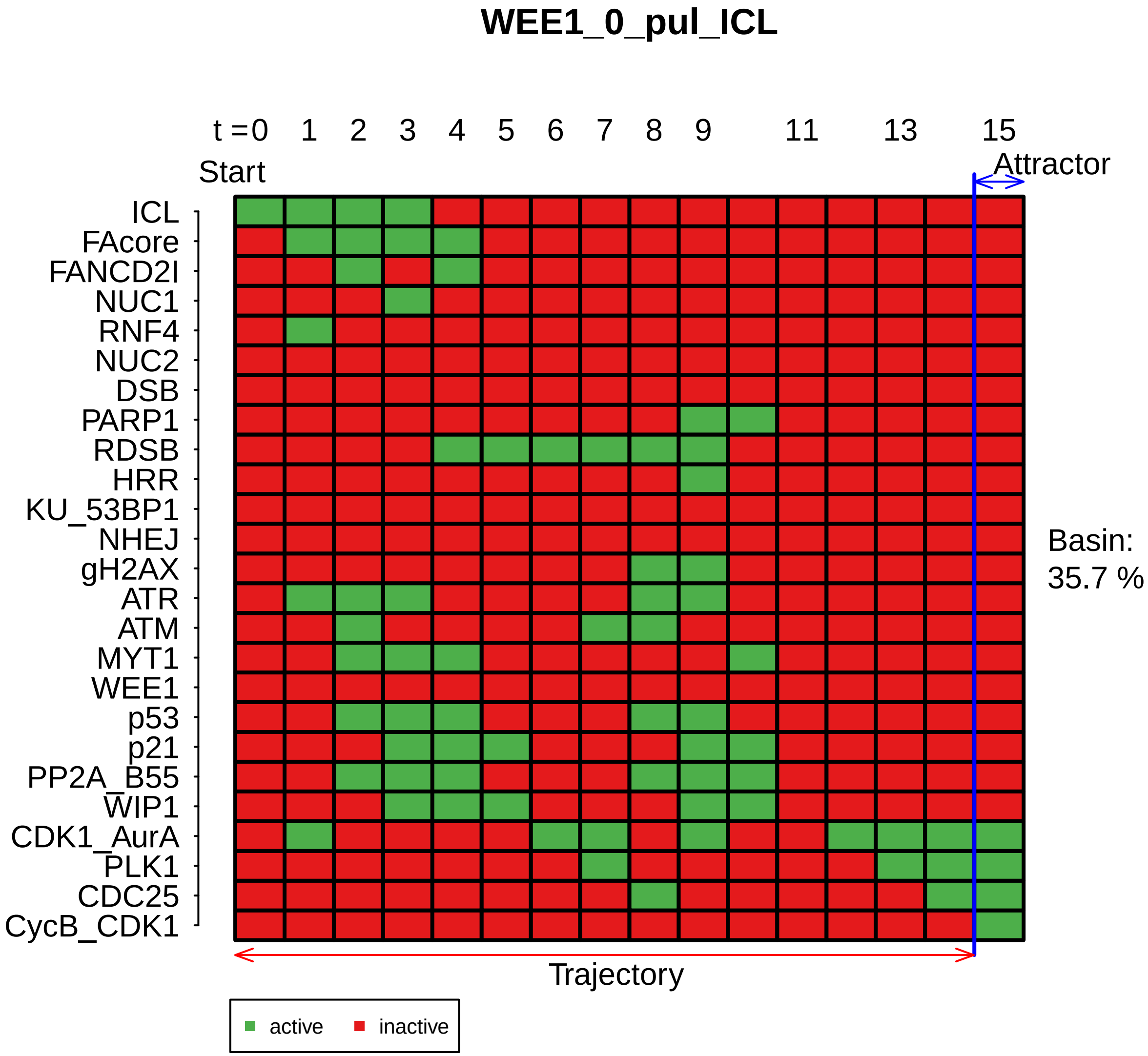

WEE1_0_pul_ICL
t =0
1
2
3
4
5
6
7
8
9
11
13
15
Start
Attractor
ICL
FAcore
FANCD2I
NUC1
RNF4
NUC2
DSB
PARP1
RDSB
HRR
KU_53BP1
NHEJ
gH2AX
ATR
ATM
MYT1
WEE1
p53
p21
PP2A_B55
WIP1
CDK1_AurA
PLK1
CDC25
CycB_CDK1
Basin:
35.7 %
Trajectory
active
inactive

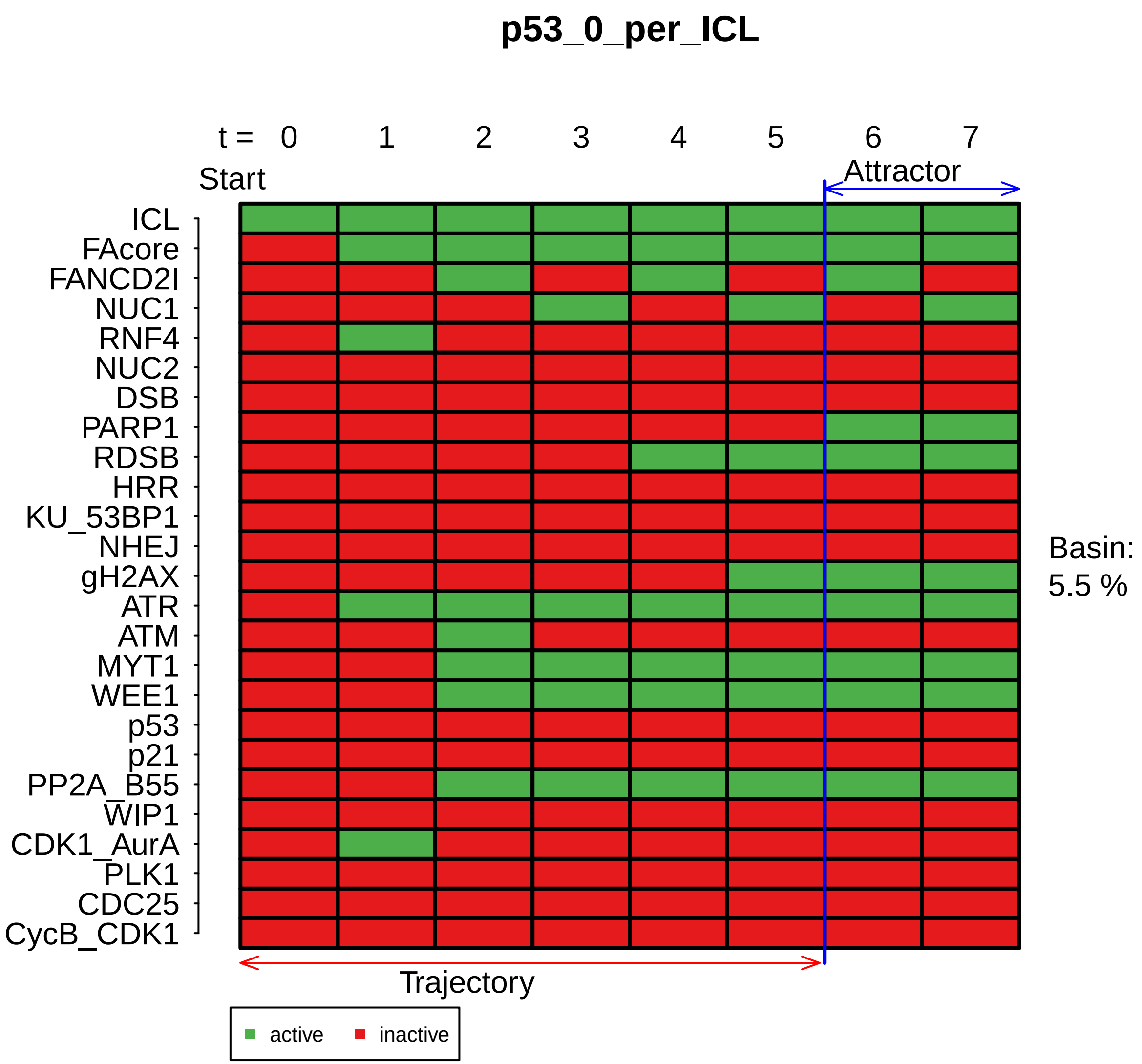
p53_0_per_ICL
t = 0 1 2 3 4 5 6 7
Start
Attractor
ICL
FAcore
FANCD2I
NUC1
RNF4
NUC2
DSB
PARP1
RDSB
HRR
KU_53BP1
NHEJ
gH2AX
ATR
ATM
MYT1
WEE1
p53
p21
PP2A_B55
WIP1
CDK1_AurA
PLK1
CDC25
CycB_CDK1
Basin:
5.5 %
Trajectory
active
inactive

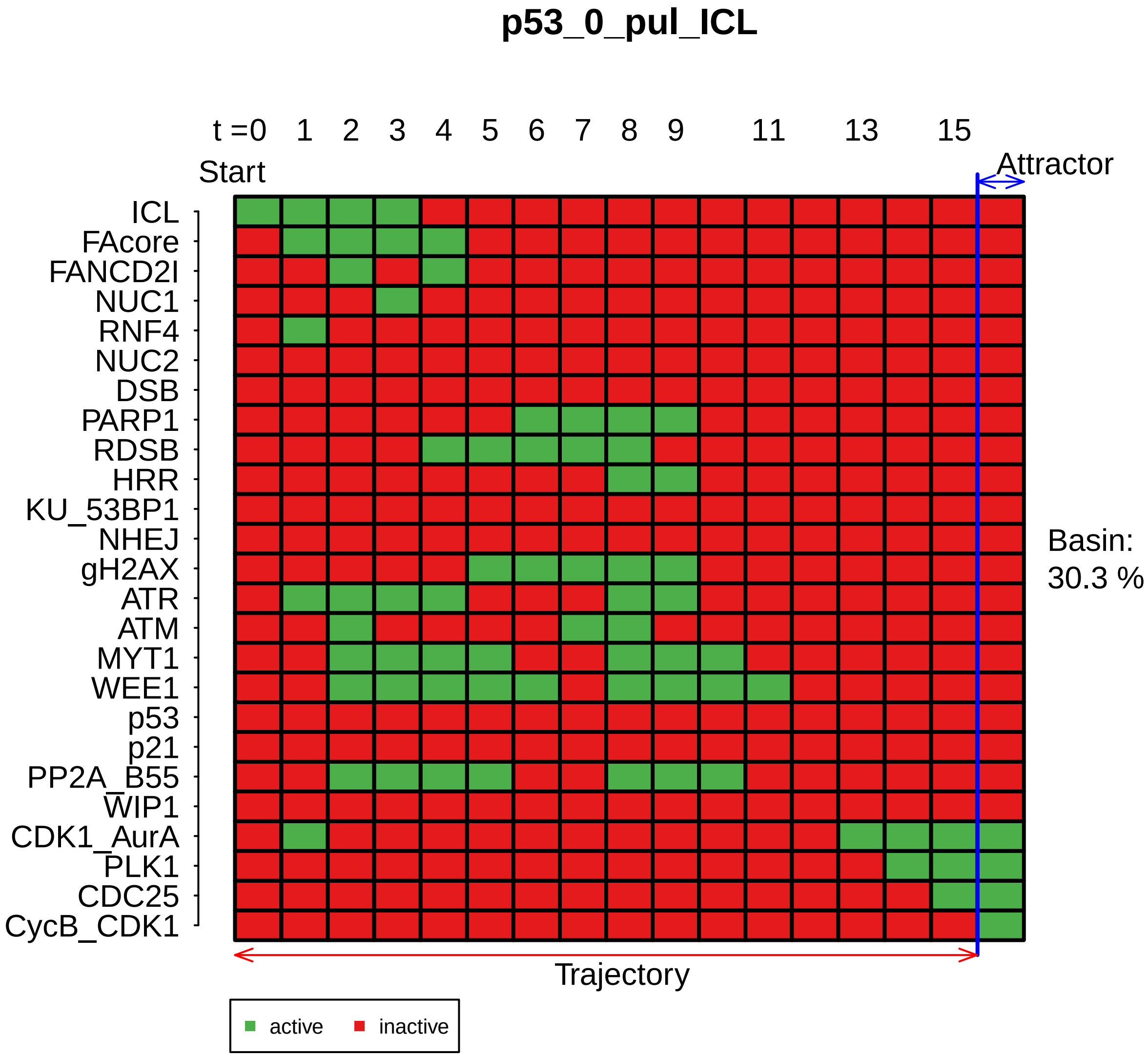

p53_0_pul_ICL
t =0
1
2
3
4
5
6
7
8
9
11
13
15
Start
Attractor
ICL
FAcore
FANCD2I
NUC1
RNF4
NUC2
DSB
PARP1
RDSB
HRR
KU_53BP1
NHEJ
gH2AX
ATR
ATM
MYT1
WEE1
p53
p21
PP2A_B55
WIP1
CDK1_AurA
PLK1
CDC25
CycB_CDK1
Basin:
30.3 %
Trajectory
active
inactive

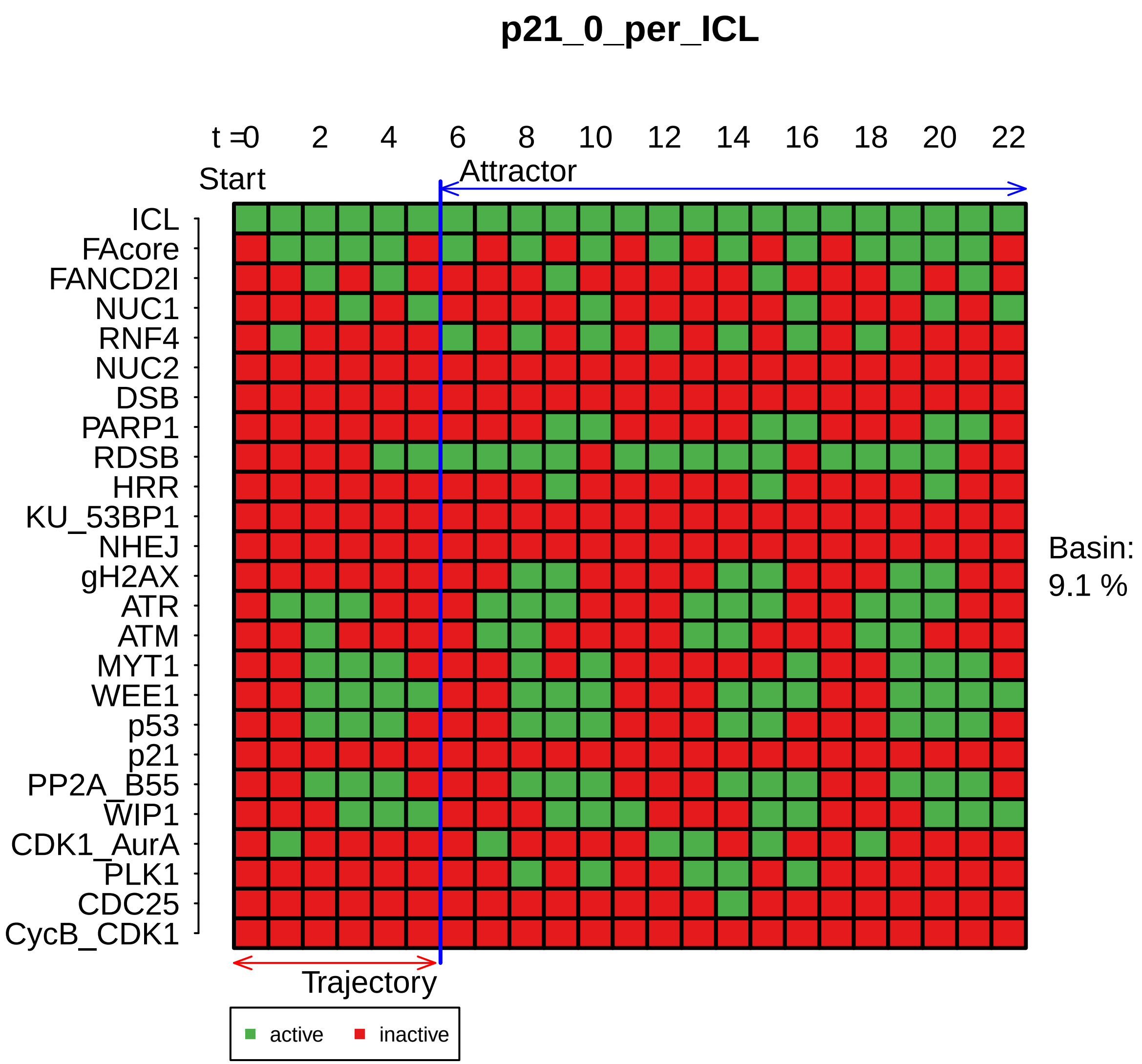

p21_0_per_ICL
t =0
2
4
6
8
10
12
14
16
18
20
22
Start
Attractor
ICL
FAcore
FANCD2I
NUC1
RNF4
NUC2
DSB
PARP1
RDSB
HRR
KU_53BP1
NHEJ
gH2AX
ATR
ATM
MYT1
WEE1
p53
p21
PP2A_B55
WIP1
CDK1_AurA
PLK1
CDC25
CycB_CDK1
Basin:
9.1 %
Trajectory
active
inactive

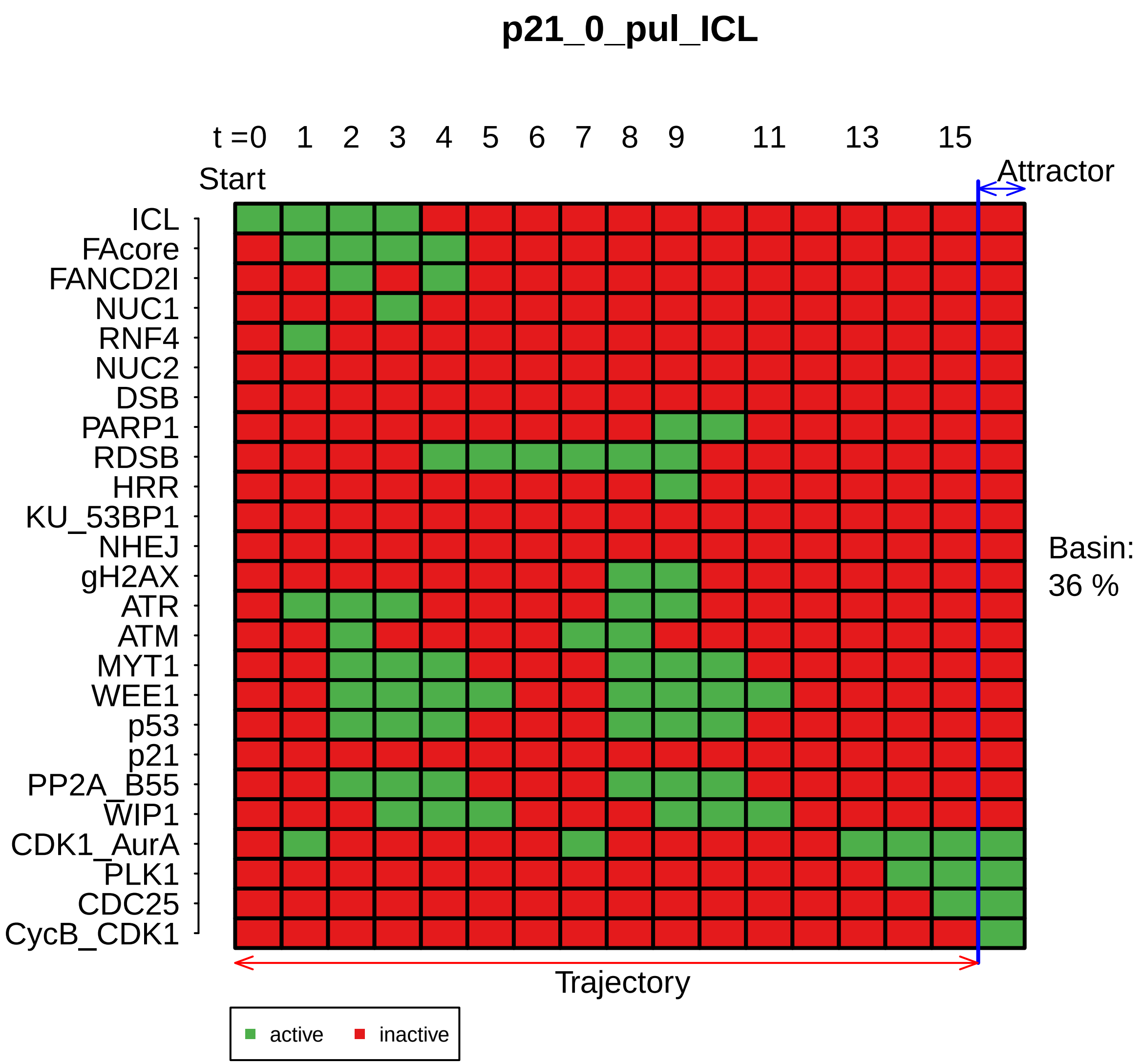
p21_0_pul_ICL
t =0 1 2 3 4 5 6 7 8 9 11 13 15
Start
Attractor
ICL
FAcore
FANCD2I
NUC1
RNF4
NUC2
DSB
PARP1
RDSB
HRR
KU_53BP1
NHEJ
gH2AX
ATR
ATM
MYT1
WEE1
p53
p21
PP2A_B55
WIP1
CDK1_AurA
PLK1
CDC25
CycB_CDK1
Basin:
36 %
Trajectory
active
inactive

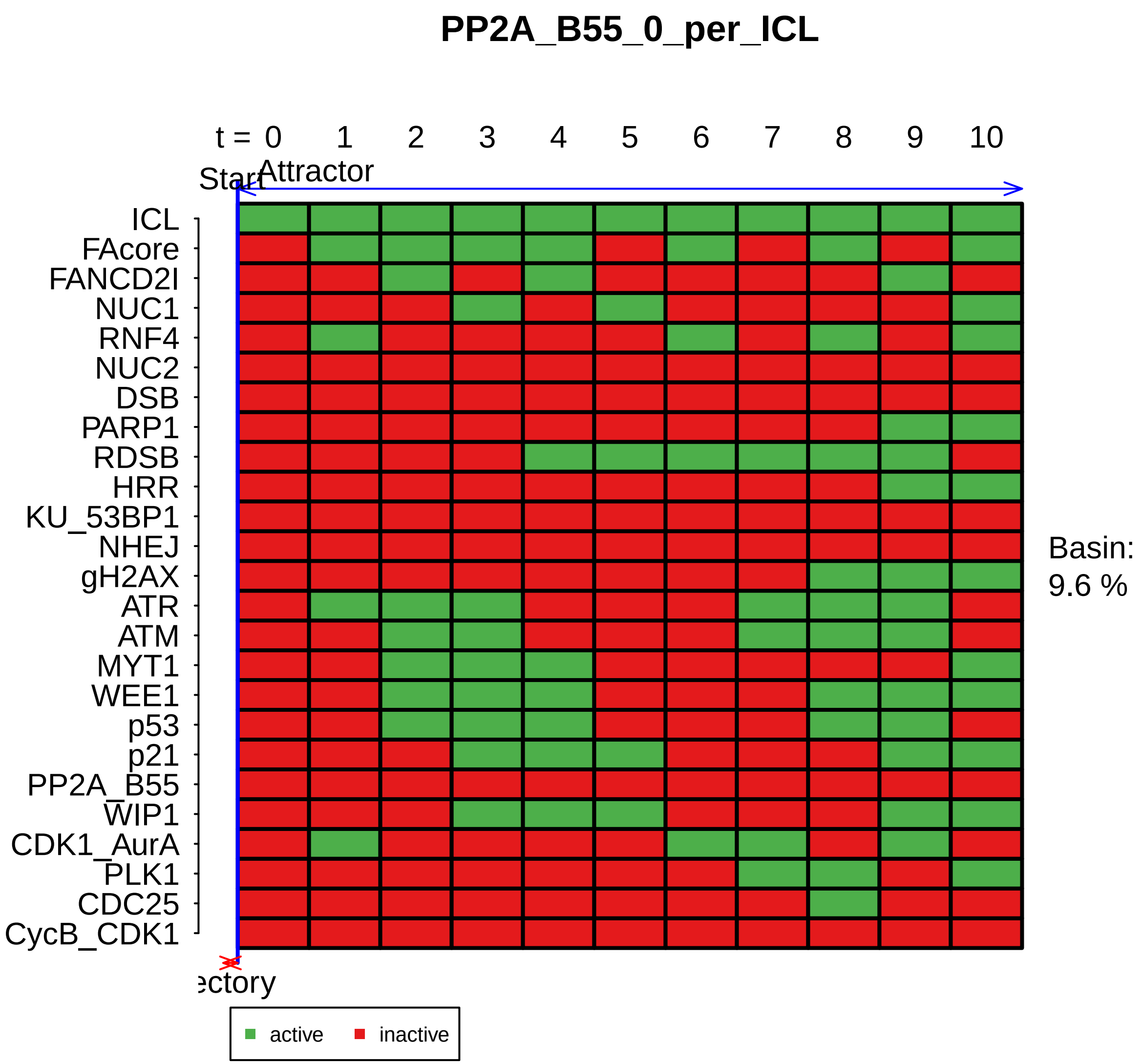

PP2A_B55_0_per_ICL
t = 0 1 2 3 4 5 6 7 8 9 10
Start
Attractor
ICL
FAcore
FANCD2I
NUC1
RNF4
NUC2
DSB
PARP1
RDSB
HRR
KU_53BP1
NHEJ
gH2AX
ATR
ATM
MYT1
WEE1
p53
p21
PP2A_B55
WIP1
CDK1_AurA
PLK1
CDC25
CycB_CDK1
Basin:
9.6 %
ectory
active
inactive

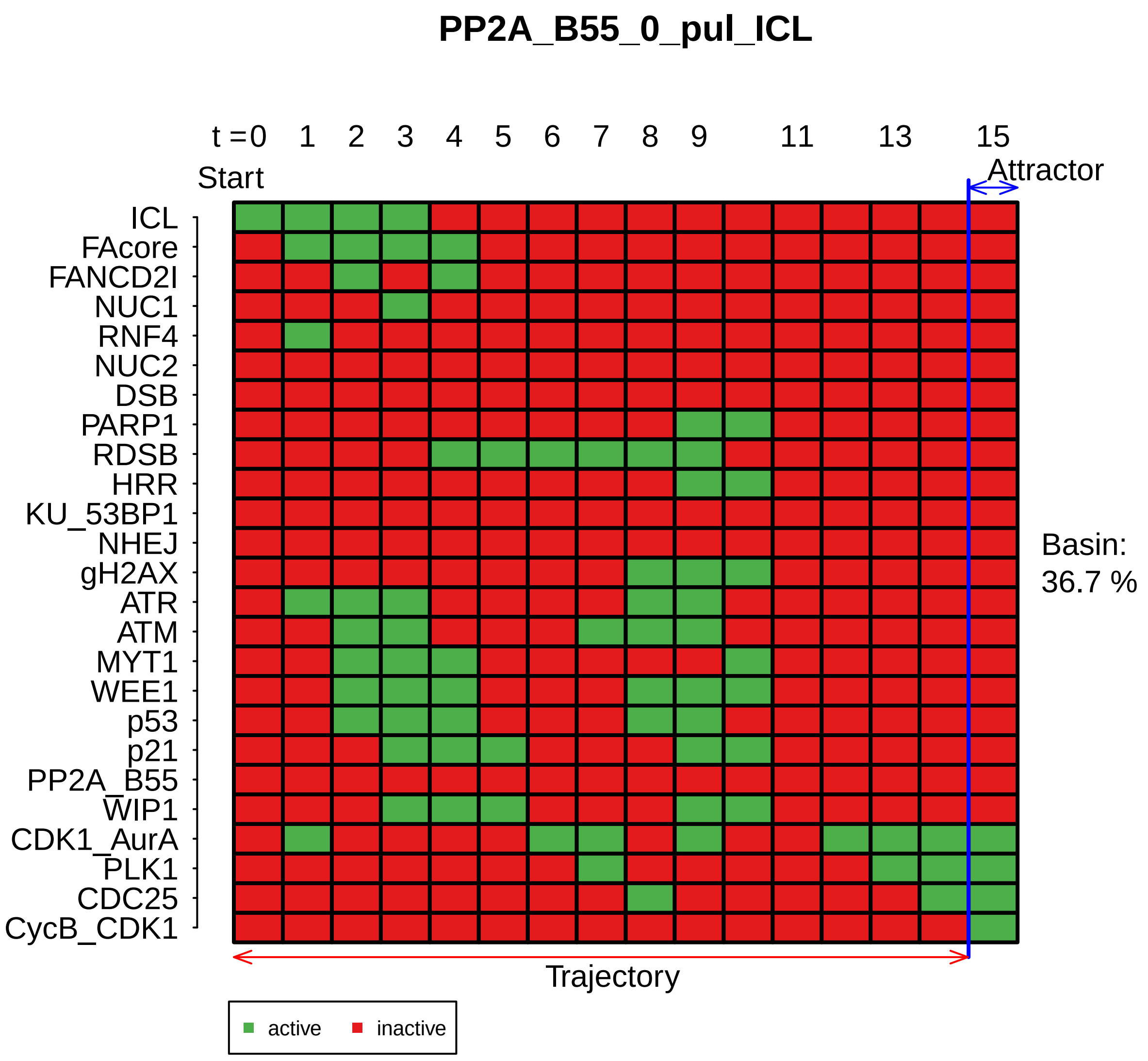
PP2A_B55_0_pul_ICL
t =0 1 2 3 4 5 6 7 8 9 11 13 15
Start
Attractor
ICL
FAcore
FANCD2I
NUC1
RNF4
NUC2
DSB
PARP1
RDSB
HRR
KU_53BP1
NHEJ
gH2AX
ATR
ATM
MYT1
WEE1
p53
p21
PP2A_B55
WIP1
CDK1_AurA
PLK1
CDC25
CycB_CDK1
Basin:
36.7 %
Trajectory
active
inactive

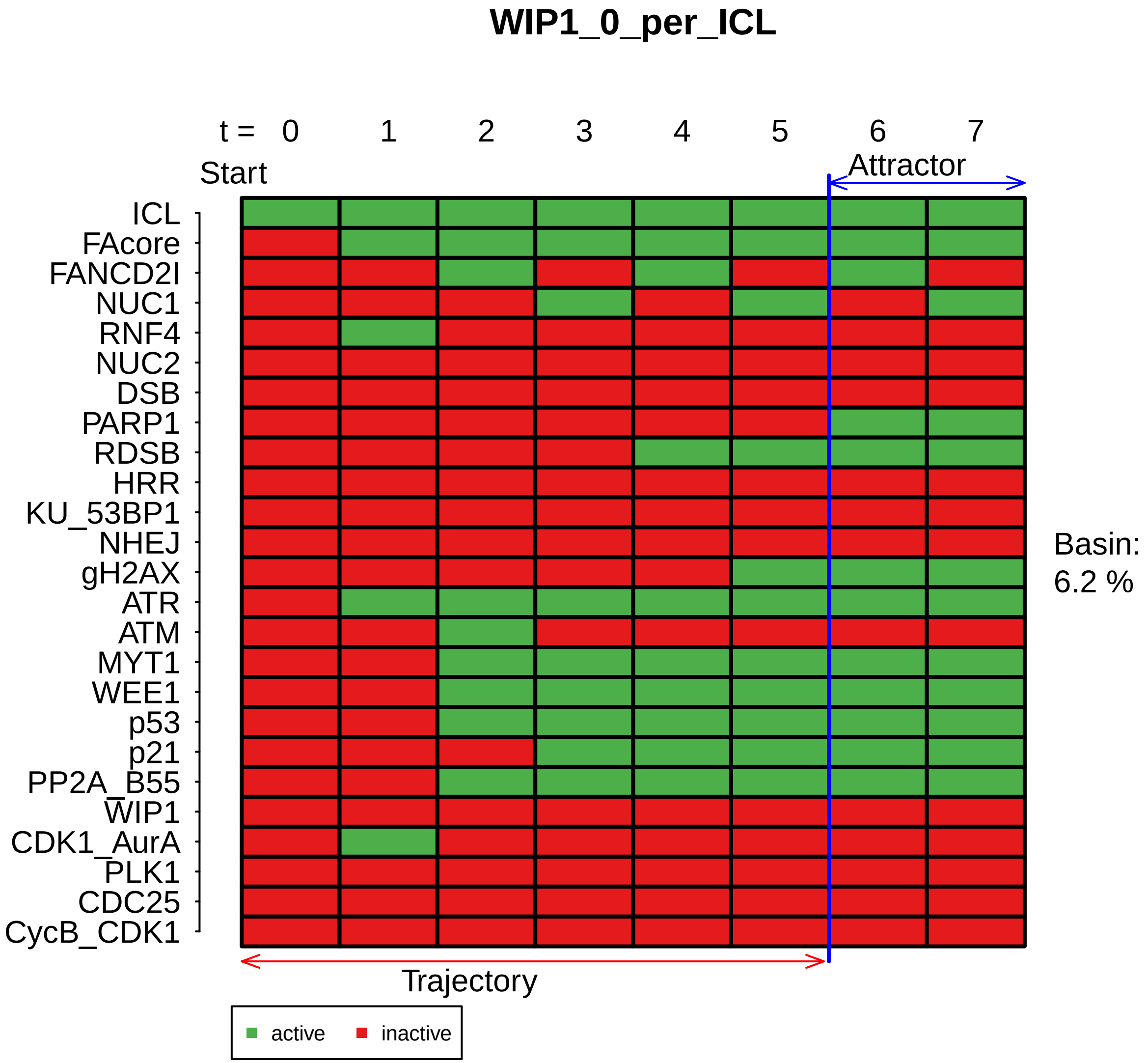
WIP1_0_per_ICL
t = 0 1 2 3 4 5 6 7
Start
Attractor
ICL
FAcore
FANCD2I
NUC1
RNF4
NUC2
DSB
PARP1
RDSB
HRR
KU_53BP1
NHEJ
gH2AX
ATR
ATM
MYT1
WEE1
p53
p21
PP2A_B55
WIP1
CDK1_AurA
PLK1
CDC25
CycB_CDK1
Basin:
6.2 %
Trajectory
active
inactive

WIP1_0_pul_ICL

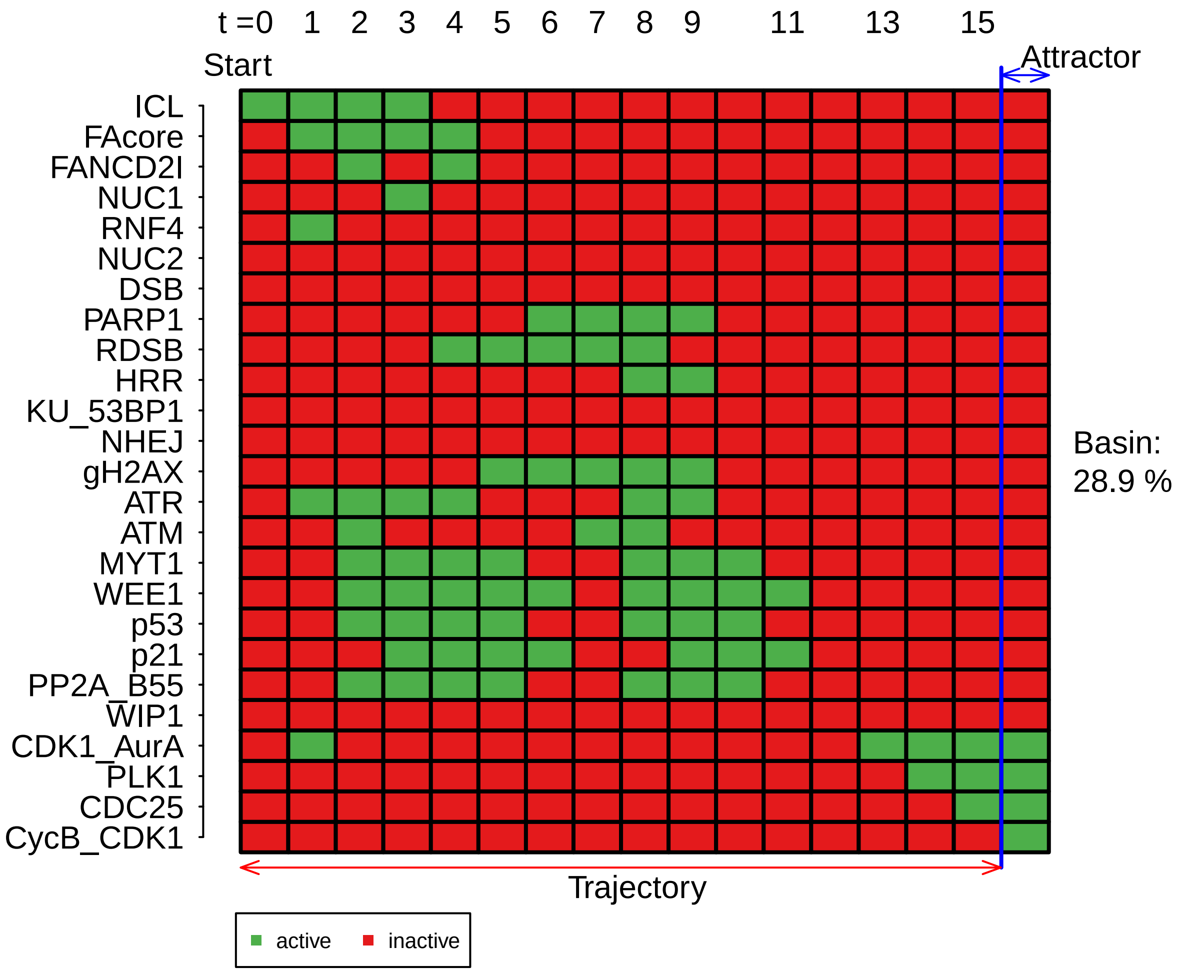

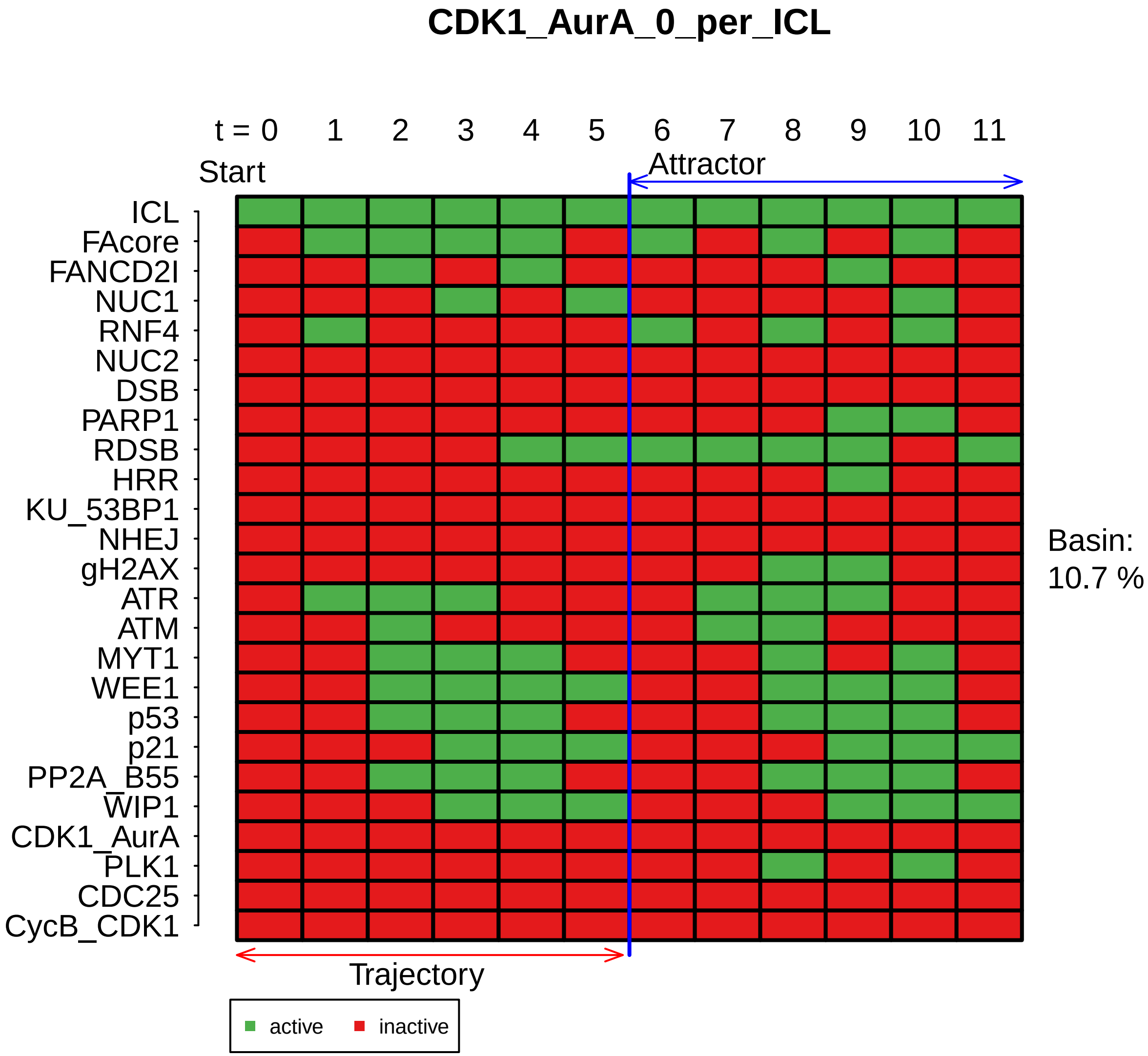

CDK1_AurA_0_per_ICL
t = 0
1
2
3
4
5
6
7
8
9
10
11
Start
Attractor
ICL
FAcore
FANCD2I
NUC1
RNF4
NUC2
DSB
PARP1
RDSB
HRR
KU_53BP1
NHEJ
gH2AX
ATR
ATM
MYT1
WEE1
p53
p21
PP2A_B55
WIP1
CDK1_AurA
PLK1
CDC25
CycB_CDK1
Basin:
10.7 %
Trajectory
active
inactive

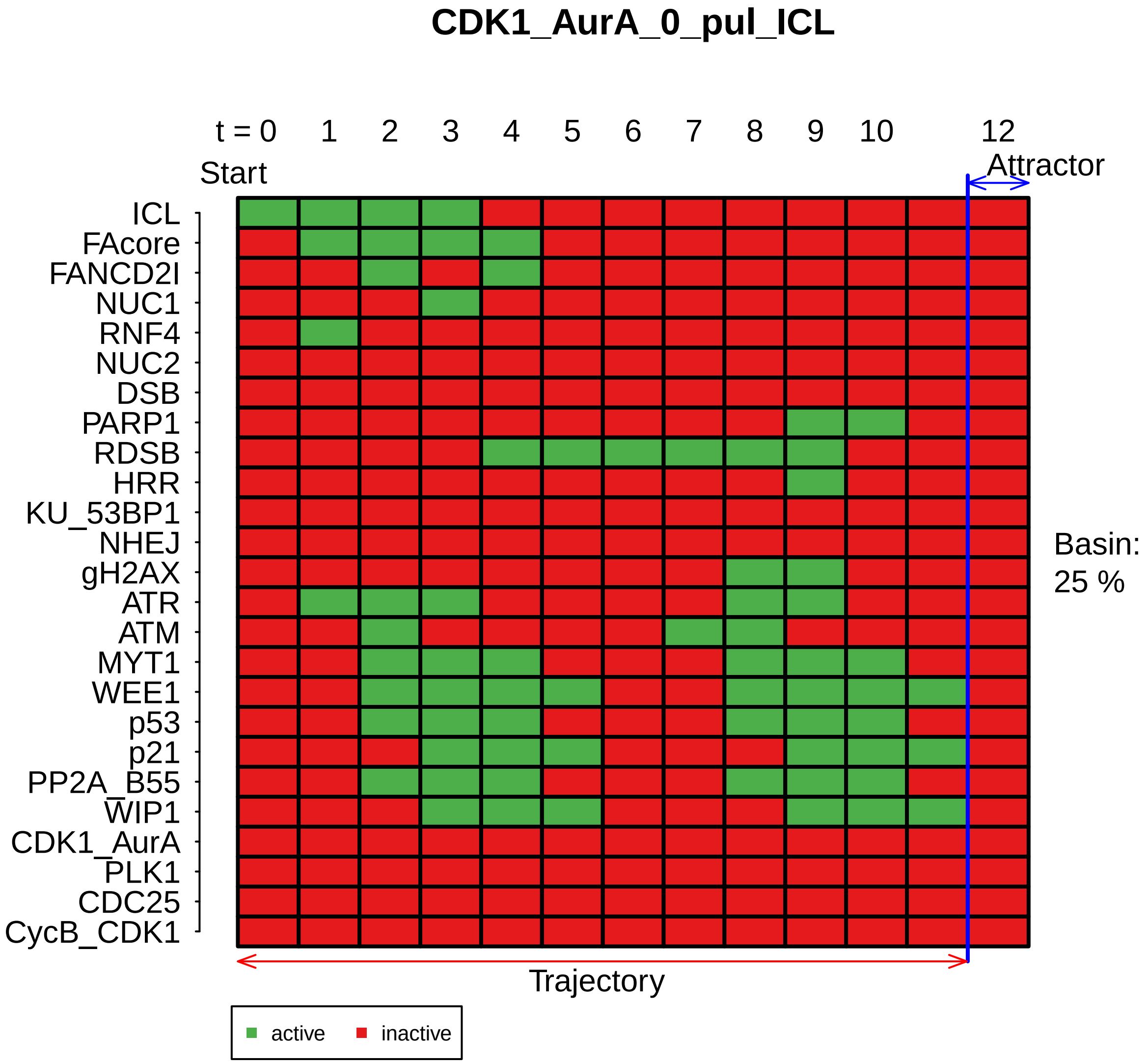
CDK1_AurA_0_pul_ICL
t = 0
1
2
3
4
5
6
7
8
9
10
12
Start
Attractor
ICL
FAcore
FANCD2I
NUC1
RNF4
NUC2
DSB
PARP1
RDSB
HRR
KU_53BP1
NHEJ
gH2AX
ATR
ATM
MYT1
WEE1
p53
p21
PP2A_B55
WIP1
CDK1_AurA
PLK1
CDC25
CycB_CDK1
Basin:
25 %
Trajectory
active
inactive

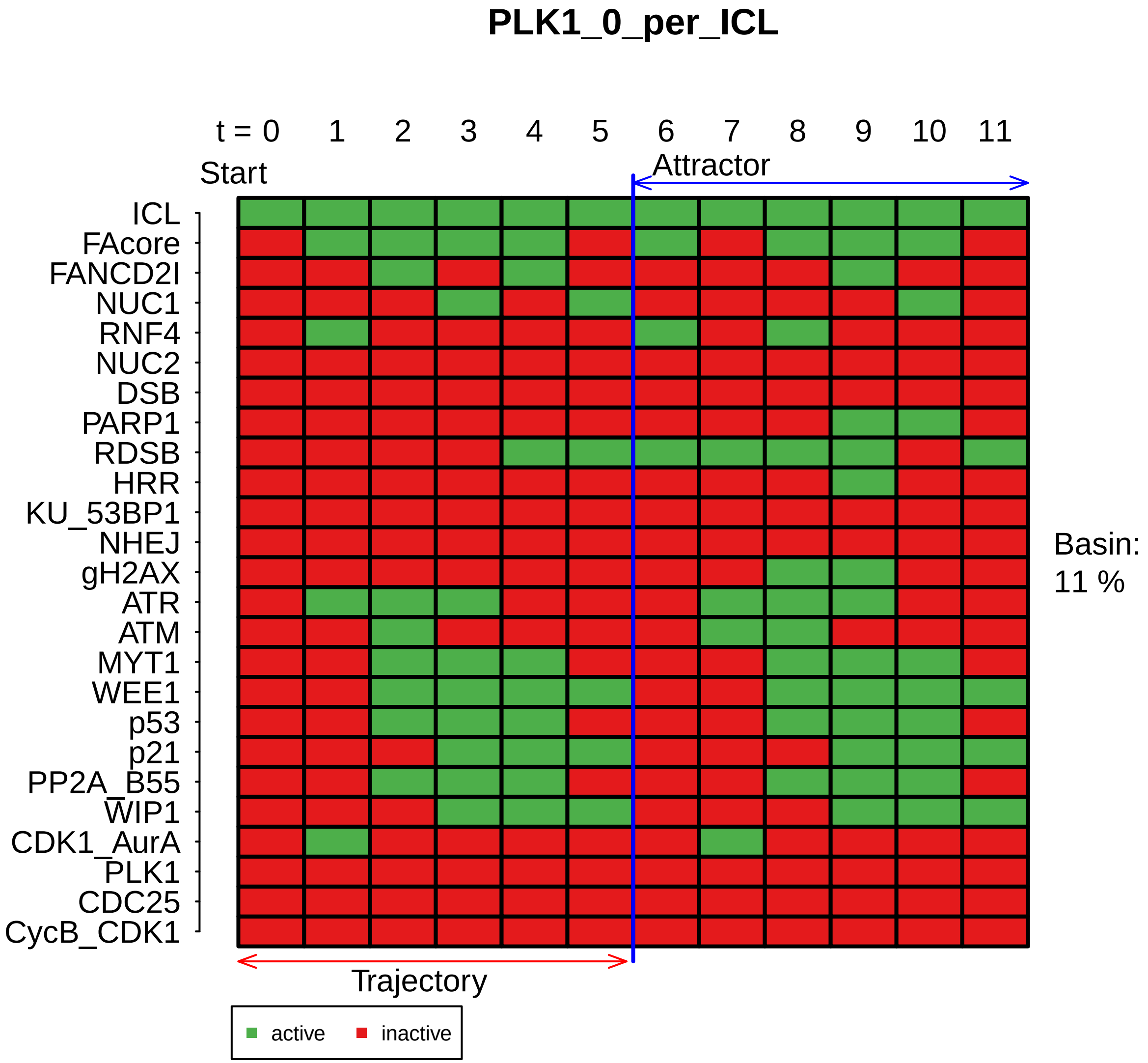

PLK1_0_per_ICL
t = 0
1
2
3
4
5
6
7
8
9
10
11
Start
Attractor
ICL
FAcore
FANCD2I
NUC1
RNF4
NUC2
DSB
PARP1
RDSB
HRR
KU_53BP1
NHEJ
gH2AX
ATR
ATM
MYT1
WEE1
p53
p21
PP2A_B55
WIP1
CDK1_AurA
PLK1
CDC25
CycB_CDK1
Basin:
11 %
Trajectory
active
inactive

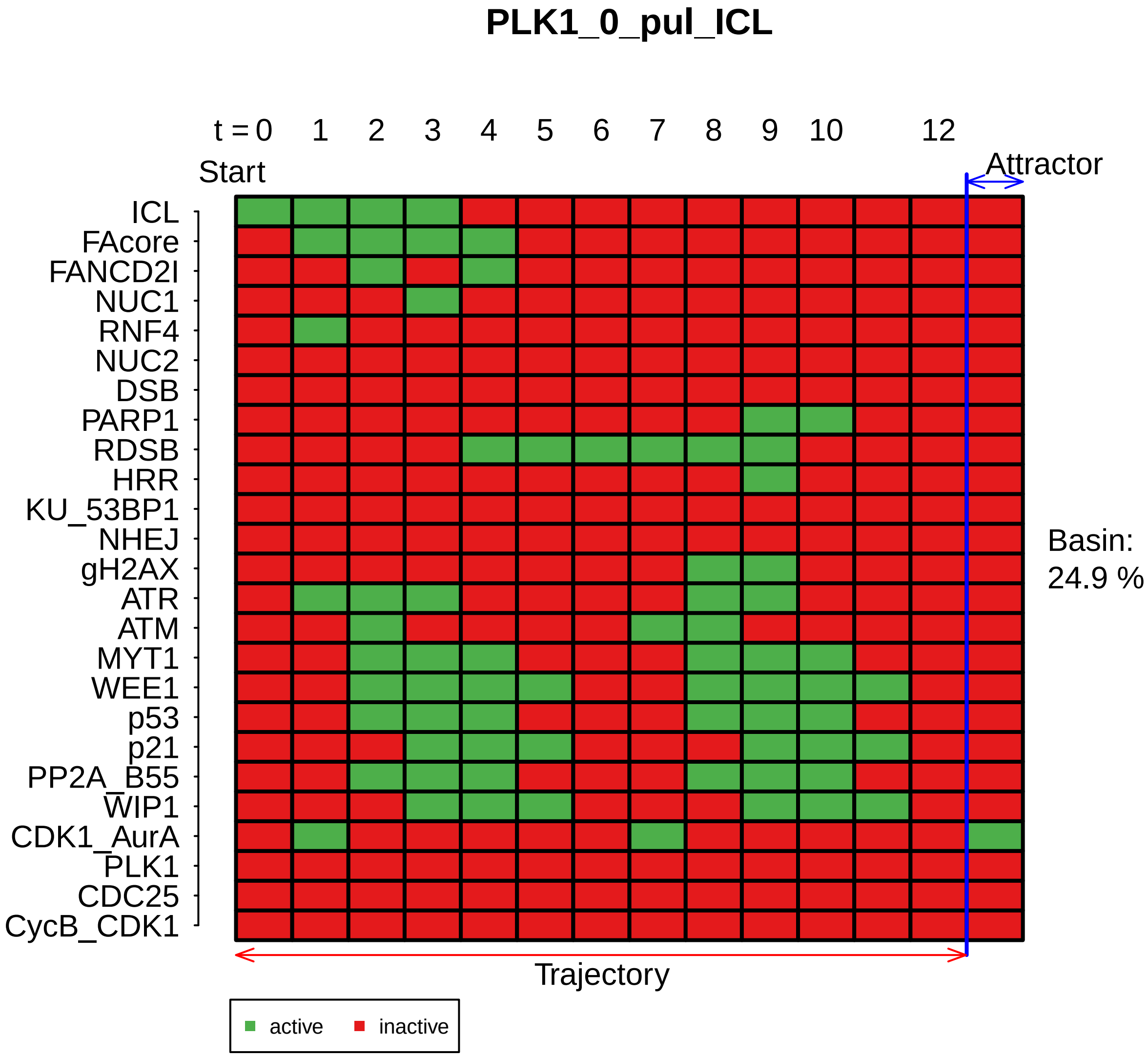
PLK1_0_pul_ICL
t = 0 1 2 3 4 5 6 7 8 9 10 12
Start
Attractor
ICL
FAcore
FANCD2I
NUC1
RNF4
NUC2
DSB
PARP1
RDSB
HRR
KU_53BP1
NHEJ
gH2AX
ATR
ATM
MYT1
WEE1
p53
p21
PP2A_B55
WIP1
CDK1_AurA
PLK1
CDC25
CycB_CDK1
Basin:
24.9 %
Trajectory
active
inactive

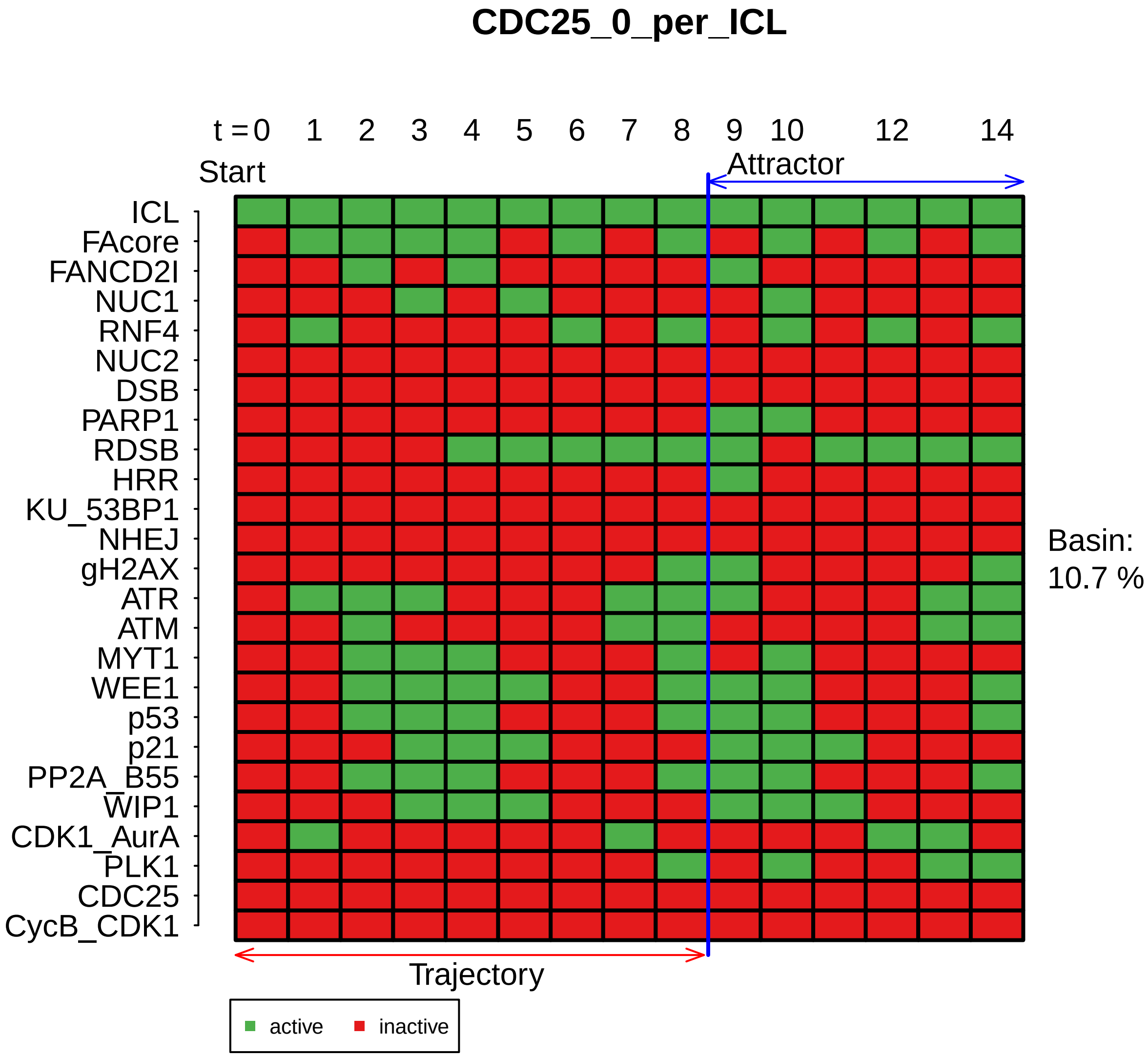
CDC25_0_per_ICL
t = 0 1 2 3 4 5 6 7 8 9 10 12 14
Start
Attractor
ICL
FAcore
FANCD2I
NUC1
RNF4
NUC2
DSB
PARP1
RDSB
HRR
KU_53BP1
NHEJ
gH2AX
ATR
ATM
MYT1
WEE1
p53
p21
PP2A_B55
WIP1
CDK1_AurA
PLK1
CDC25
CycB_CDK1
Basin:
10.7 %
Trajectory
active
inactive

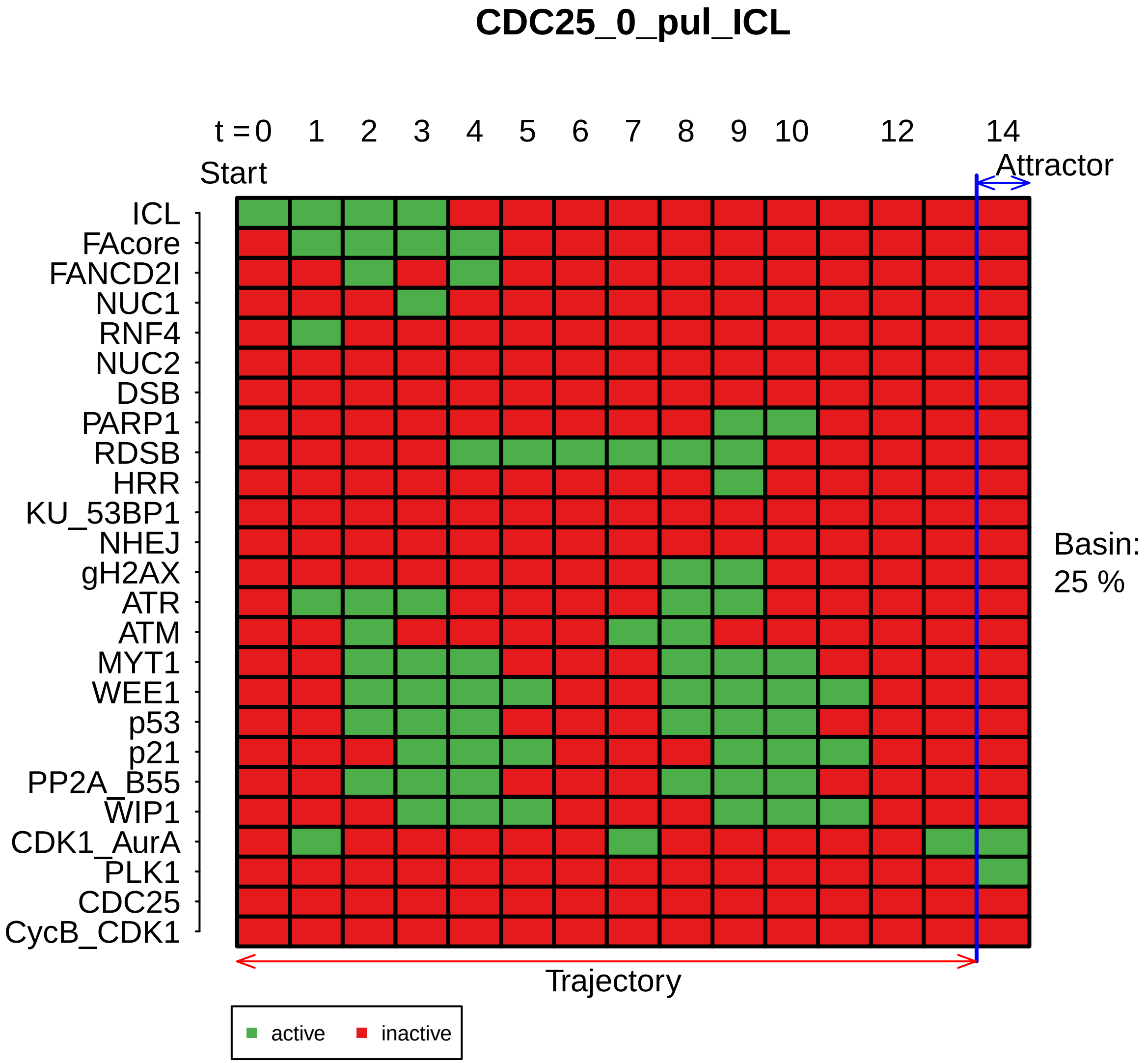

CDC25_0_pul_ICL
t = 0 1 2 3 4 5 6 7 8 9 10 12 14
Start
Attractor
ICL
FAcore
FANCD2I
NUC1
RNF4
NUC2
DSB
PARP1
RDSB
HRR
KU_53BP1
NHEJ
gH2AX
ATR
ATM
MYT1
WEE1
p53
p21
PP2A_B55
WIP1
CDK1_AurA
PLK1
CDC25
CycB_CDK1
Basin:
25 %
Trajectory
active
inactive

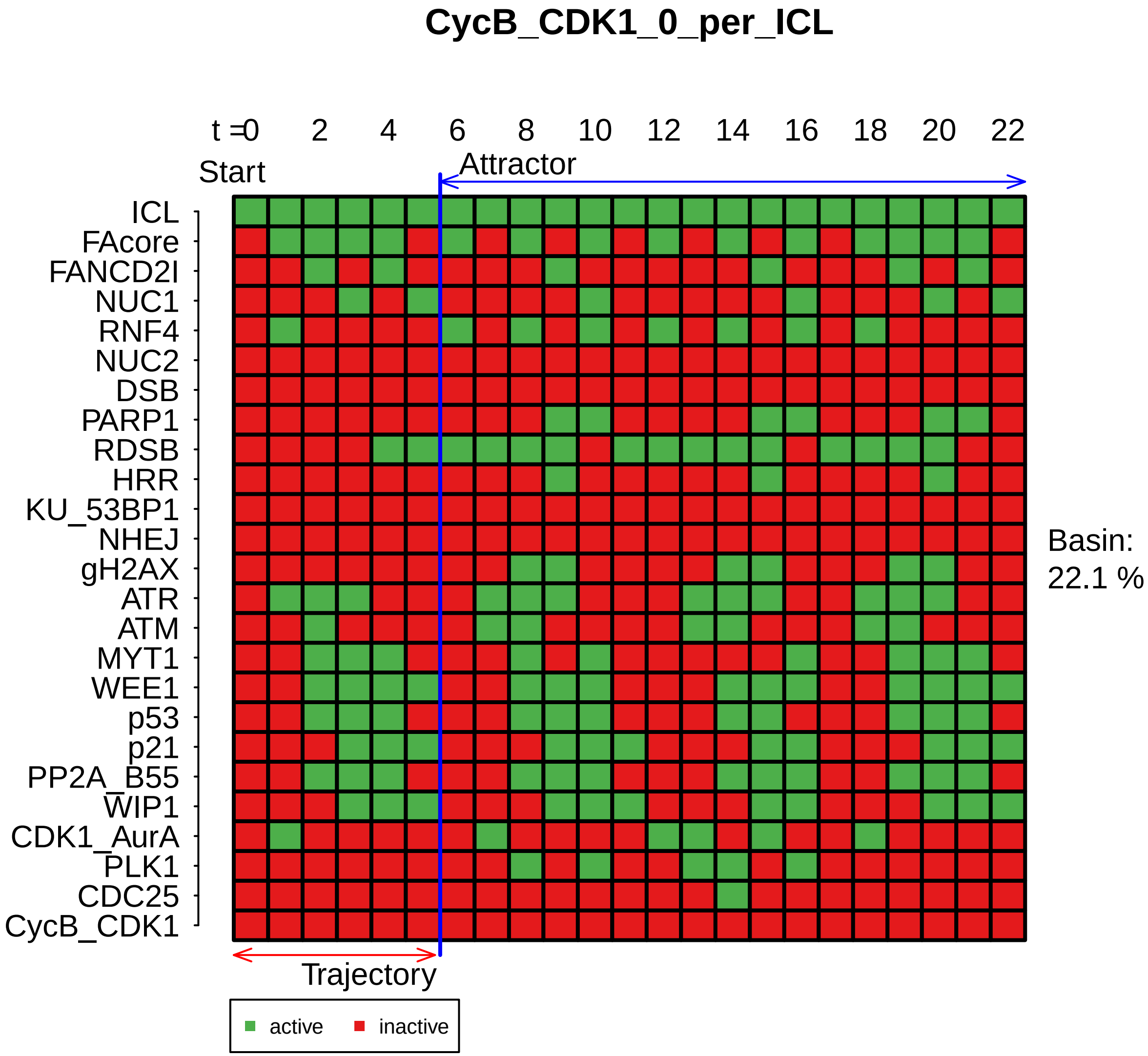

CycB_CDK1_0_per_ICL
t = 0 2 4 6 8 10 12 14 16 18 20 22
Start
Attractor
ICL
FAcore
FANCD2I
NUC1
RNF4
NUC2
DSB
PARP1
RDSB
HRR
KU_53BP1
NHEJ
gH2AX
ATR
ATM
MYT1
WEE1
p53
p21
PP2A_B55
WIP1
CDK1_AurA
PLK1
CDC25
CycB_CDK1
Basin:
22.1 %
Trajectory
active
inactive

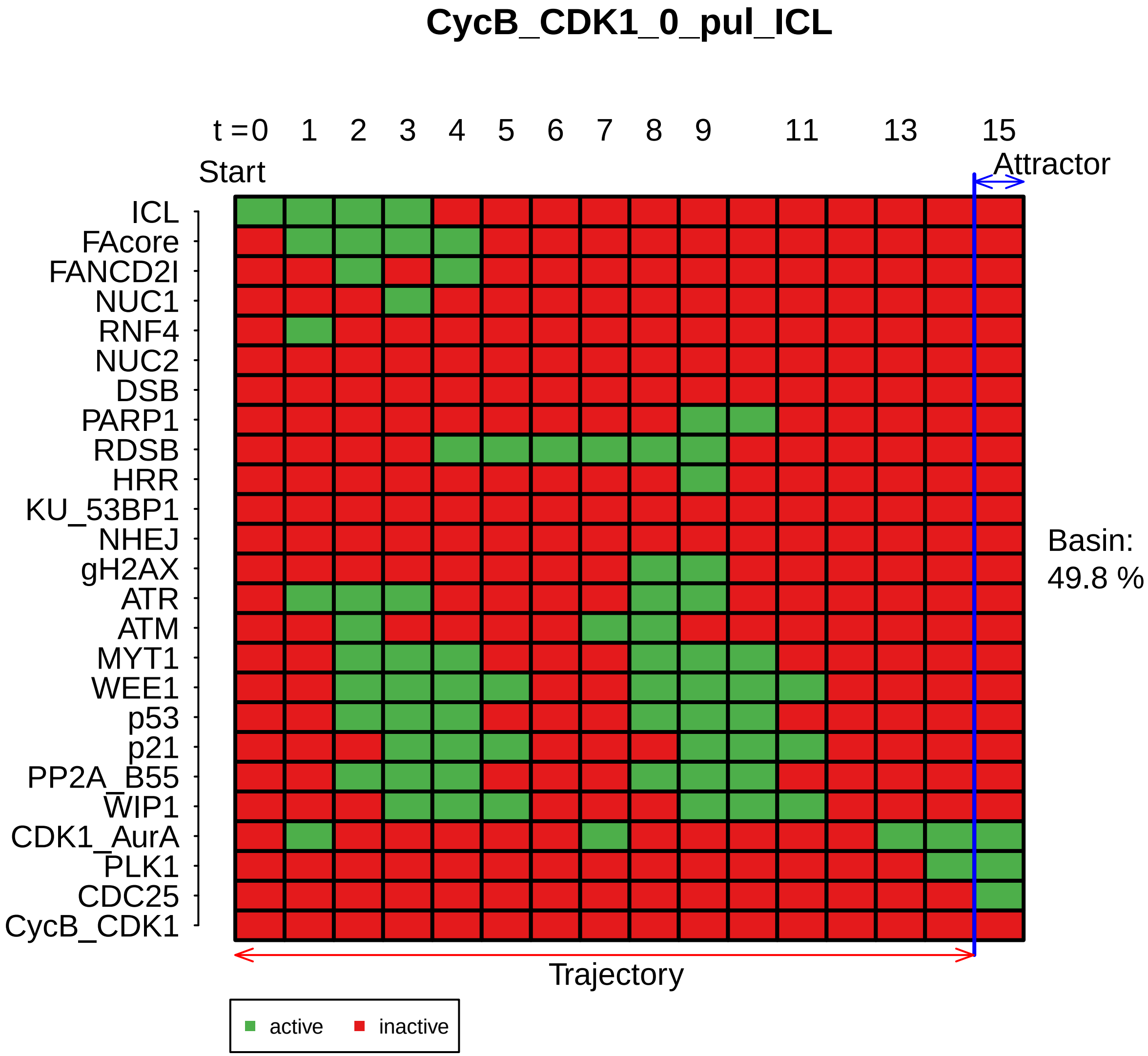
CycB_CDK1_0_pul_ICL
t =0
1
2
3
4
5
6
7
8
9
11
13
15
Start
Attractor
ICL
FAcore
FANCD2I
NUC1
RNF4
NUC2
DSB
PARP1
RDSB
HRR
KU_53BP1
NHEJ
gH2AX
ATR
ATM
MYT1
WEE1
p53
p21
PP2A_B55
WIP1
CDK1_AurA
PLK1
CDC25
CycB_CDK1
Basin:
49.8 %
Trajectory
active
inactive
