# Supplementary material for: WIP1 Contributes to the Adaptation of Fanconi Anemia Cells to DNA Damage as Determined by the Regulatory Network of the Fanconi Anemia and Checkpoint Recovery Pathways
Source: Front Genet. 2019 May 3;10:411. doi: 10.3389/fgene.2019.00411 (PMC6509935; doi:10.3389/fgene.2019.00411)
Supplement: Supplementary file 4 [file Data_Sheet_4.pdf]

Supplementary material S3. FANCD2I double null mutant simulations. All the simulations presented in this section have inactivation of the node FANCD2I and additional inactivation of the node specified in every page. These simulations were run in presence of a single pulse of ICL or persistent presence of ICL.

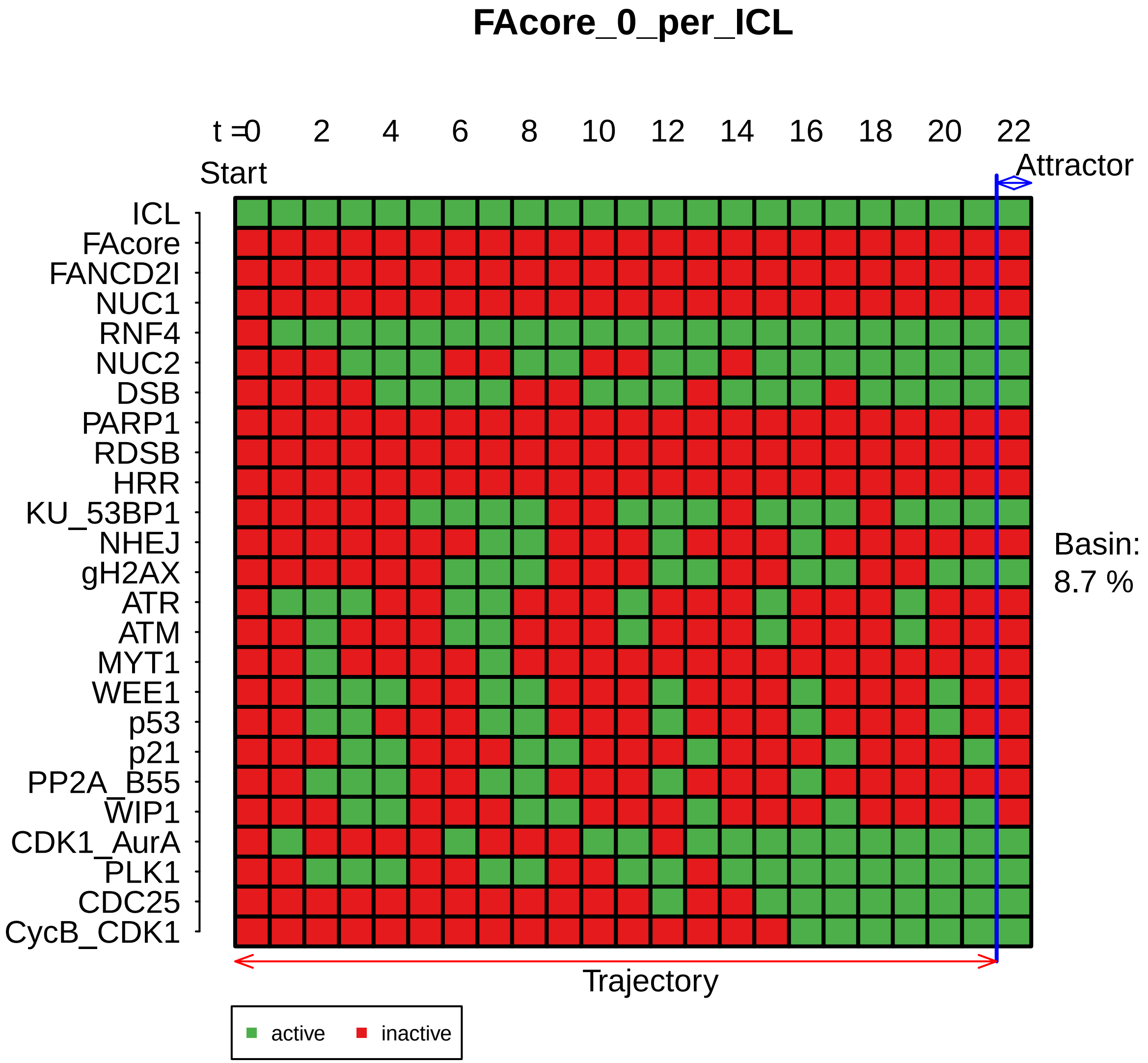
FAcore_0_per_ICL
t = 0
2
4
6
8
10
12
14
16
18
20
22
Start
Attractor
ICL
FAcore
FANCD2I
NUC1
RNF4
NUC2
DSB
PARP1
RDSB
HRR
KU_53BP1
NHEJ
gH2AX
ATR
ATM
MYT1
WEE1
p53
p21
PP2A_B55
WIP1
CDK1_AurA
PLK1
CDC25
CycB_CDK1
Basin:
8.7 %
Trajectory
active
inactive

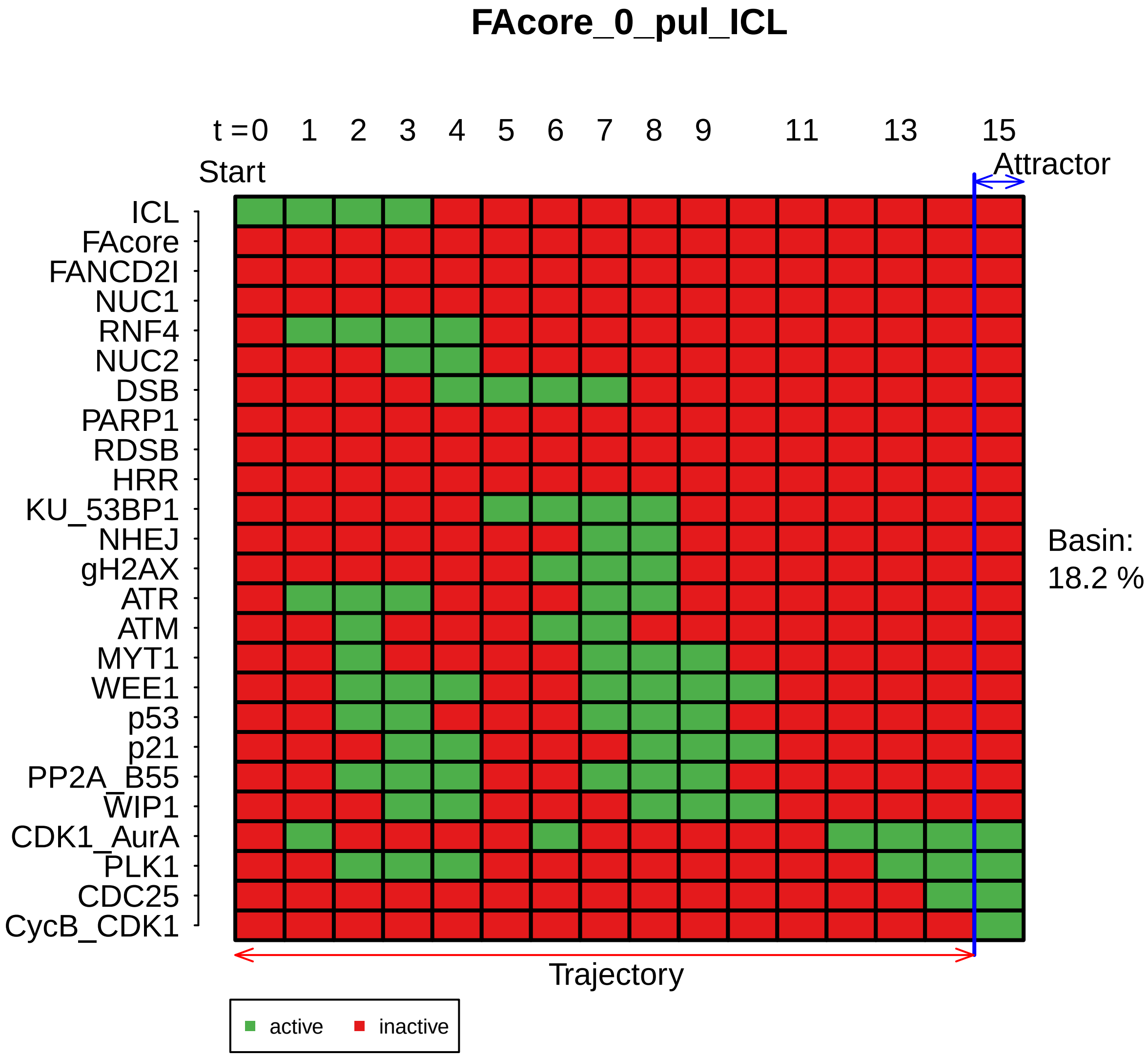

FAcore_0_pul_ICL
t =0 1 2 3 4 5 6 7 8 9 11 13 15
Start
Attractor
ICL
FAcore
FANCD2I
NUC1
RNF4
NUC2
DSB
PARP1
RDSB
HRR
KU_53BP1
NHEJ
gH2AX
ATR
ATM
MYT1
WEE1
p53
p21
PP2A_B55
WIP1
CDK1_AurA
PLK1
CDC25
CycB_CDK1
Basin:
18.2 %
Trajectory
active
inactive

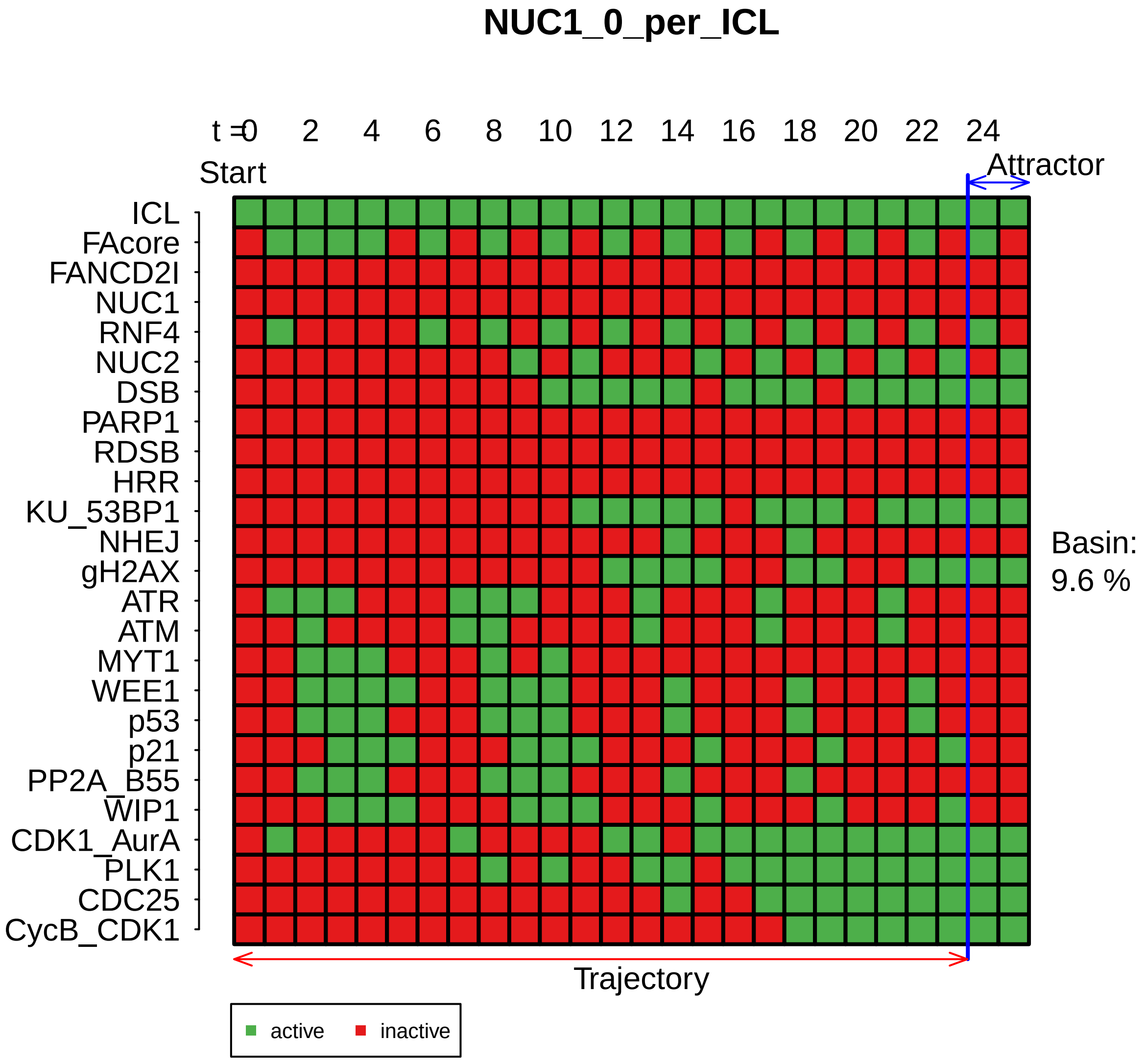
NUC1_0_per_ICL
t =
0
2
4
6
8
10
12
14
16
18
20
22
24
Start
Attractor
ICL
FAcore
FANCD2I
NUC1
RNF4
NUC2
DSB
PARP1
RDSB
HRR
KU_53BP1
NHEJ
gH2AX
ATR
ATM
MYT1
WEE1
p53
p21
PP2A_B55
WIP1
CDK1_AurA
PLK1
CDC25
CycB_CDK1
Basin:
9.6 %
Trajectory
active
inactive

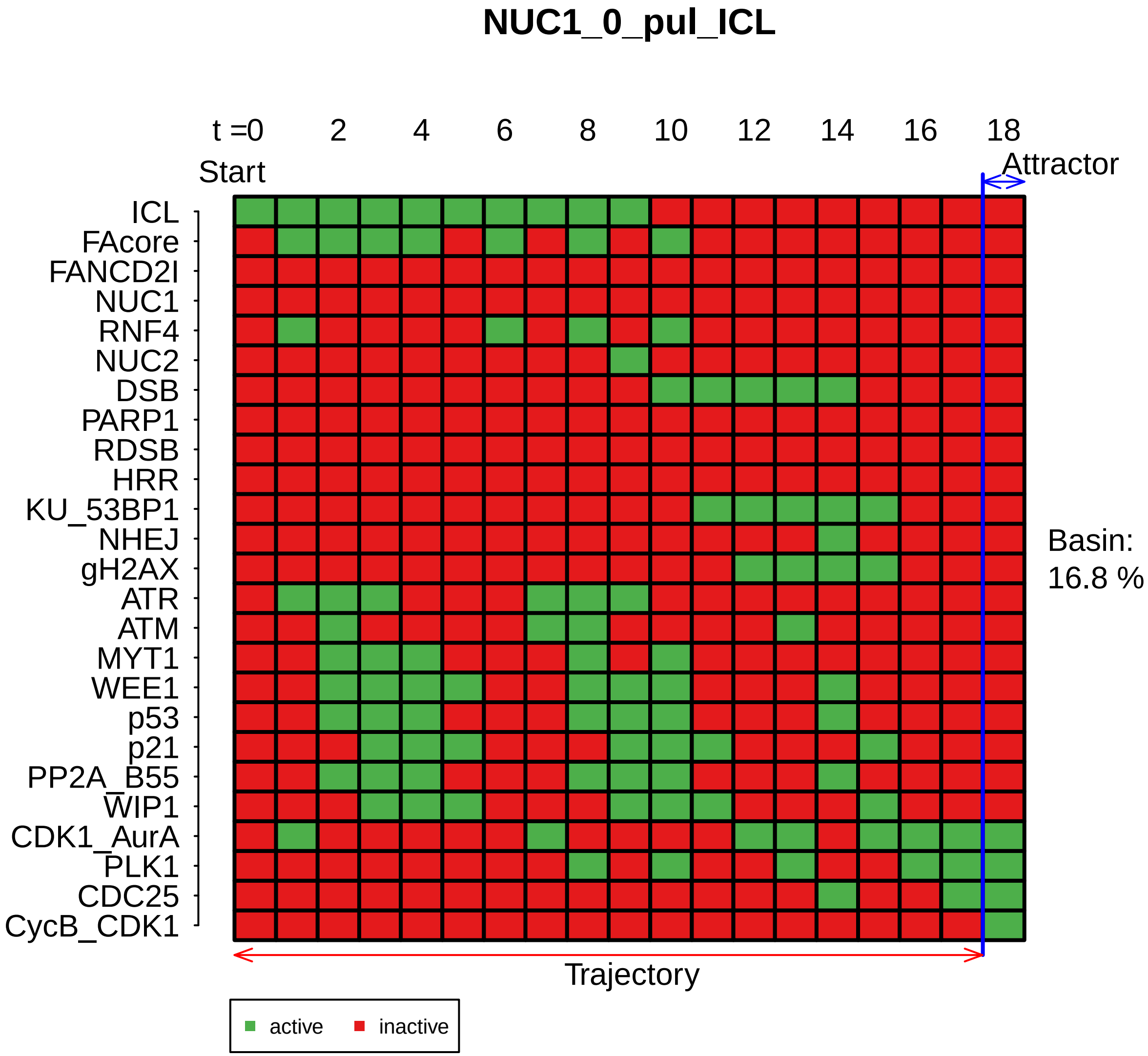

NUC1_0_pul_ICL
t =0
2
4
6
8
10
12
14
16
18
Start
Attractor
ICL
FAcore
FANCD2I
NUC1
RNF4
NUC2
DSB
PARP1
RDSB
HRR
KU_53BP1
NHEJ
gH2AX
ATR
ATM
MYT1
WEE1
p53
p21
PP2A_B55
WIP1
CDK1_AurA
PLK1
CDC25
CycB_CDK1
Basin:
16.8 %
Trajectory
active
inactive

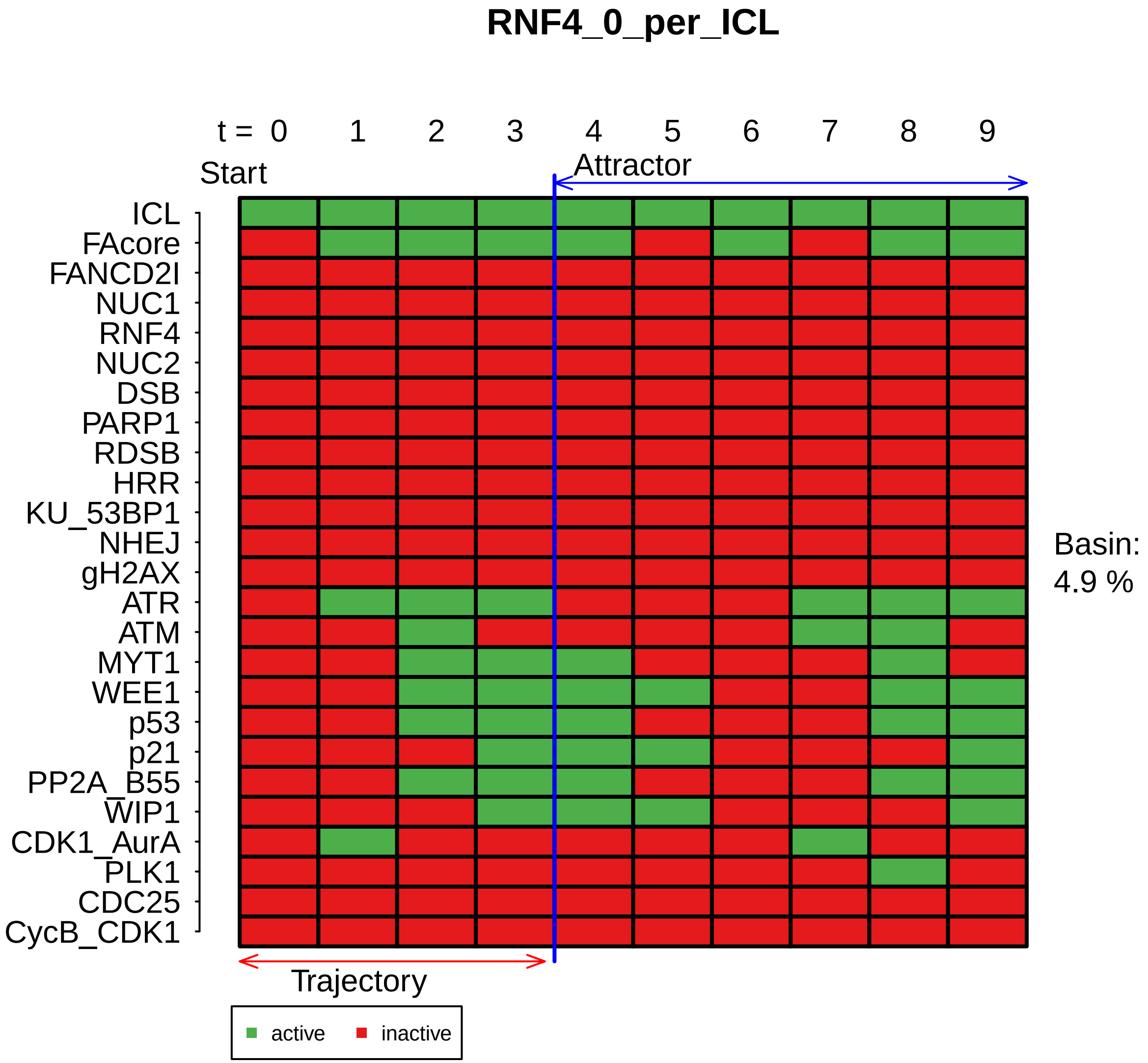

RNF4_0_per_ICL
t = 0
1
2
3
4
5
6
7
8
9
Start
Attractor
ICL
FAcore
FANCD2I
NUC1
RNF4
NUC2
DSB
PARP1
RDSB
HRR
KU_53BP1
NHEJ
gH2AX
ATR
ATM
MYT1
WEE1
p53
p21
PP2A_B55
WIP1
CDK1_AurA
PLK1
CDC25
CycB_CDK1
Basin:
4.9 %
Trajectory
active
inactive

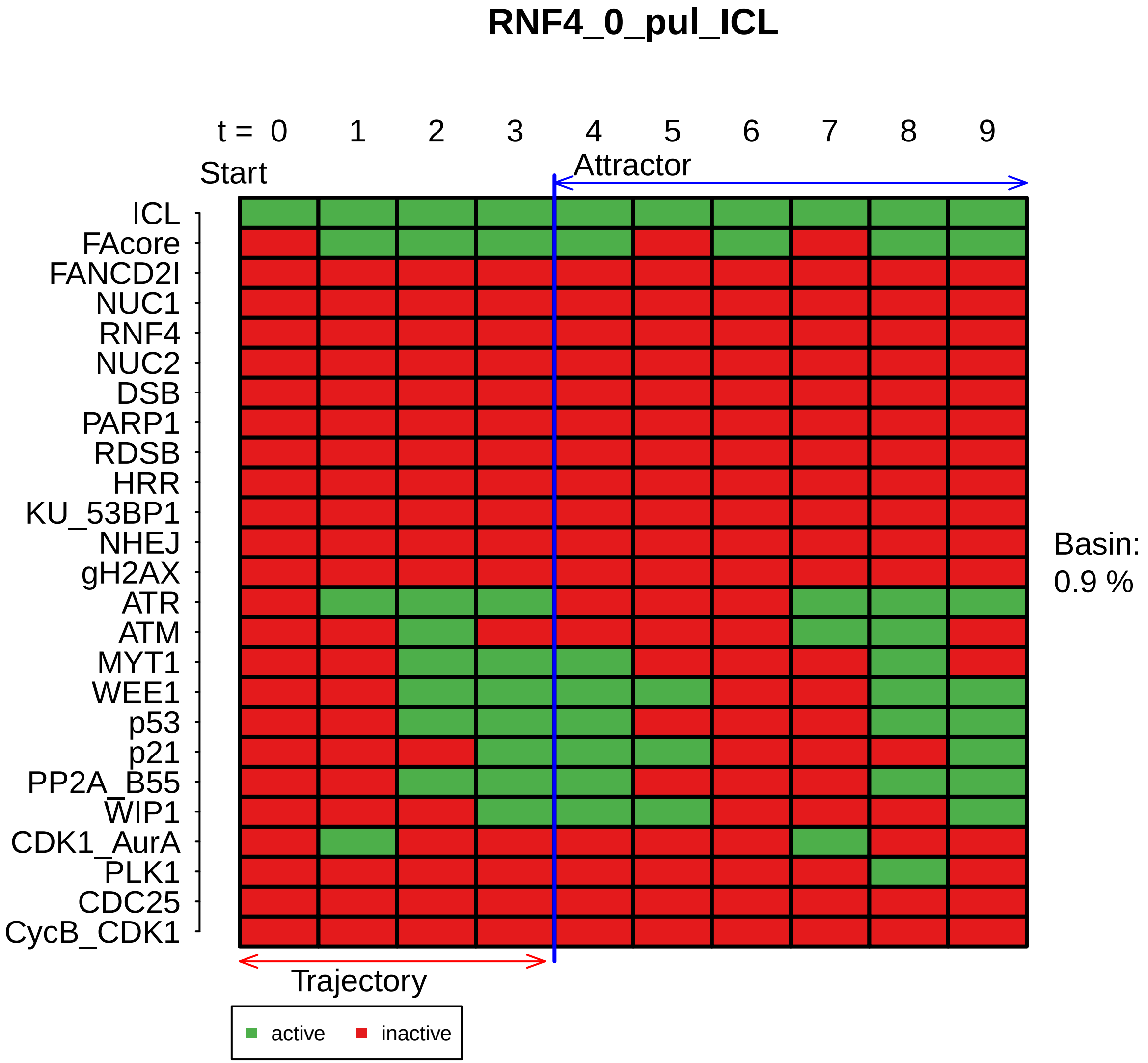
RNF4_0_pul_ICL
t = 0 1 2 3 4 5 6 7 8 9
Start
Attractor
ICL
FAcore
FANCD2I
NUC1
RNF4
NUC2
DSB
PARP1
RDSB
HRR
KU_53BP1
NHEJ
gH2AX
ATR
ATM
MYT1
WEE1
p53
p21
PP2A_B55
WIP1
CDK1_AurA
PLK1
CDC25
CycB_CDK1
Basin:
0.9 %
Trajectory
active
inactive

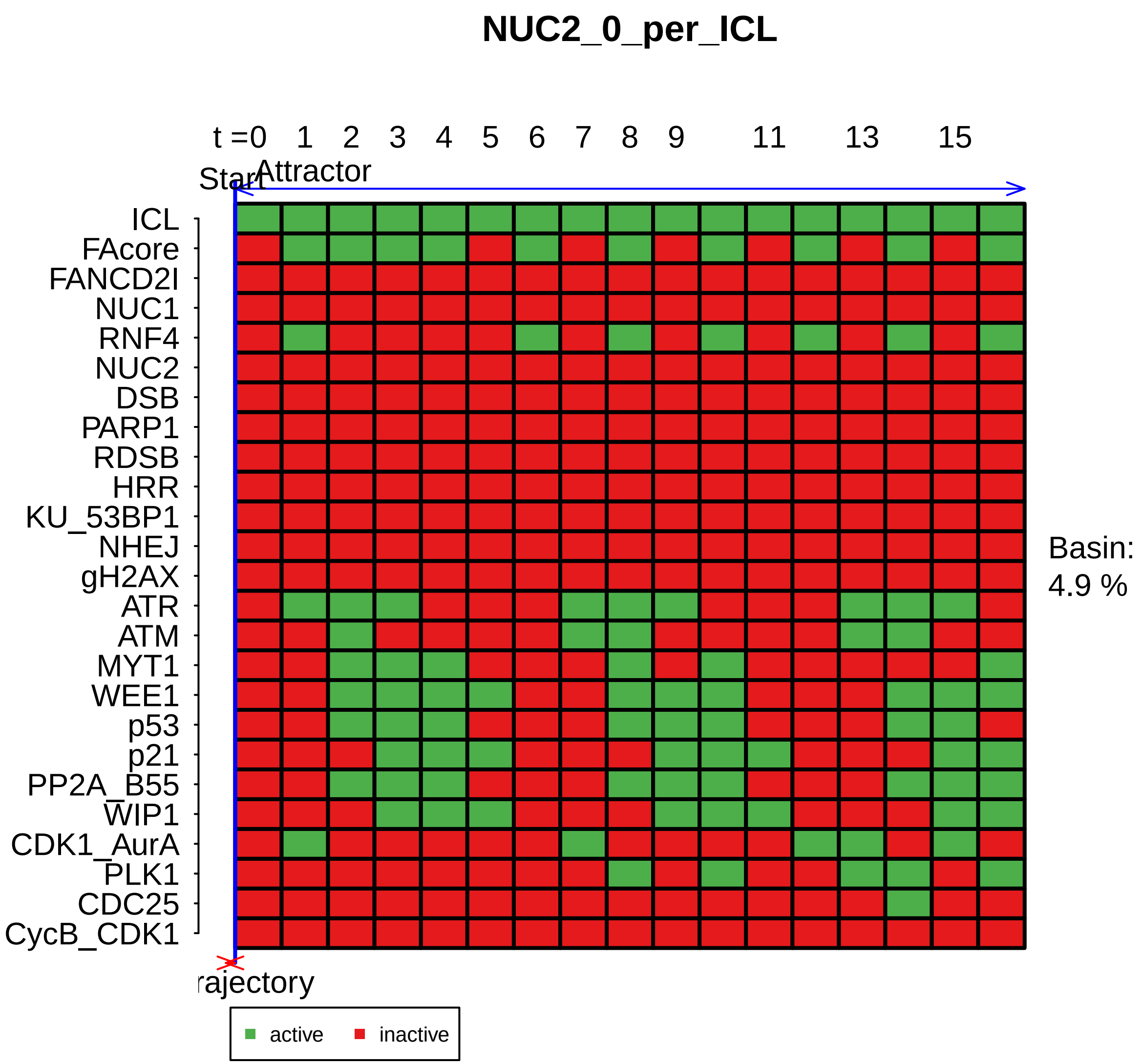
NUC2_0_per_ICL
t =0 1 2 3 4 5 6 7 8 9 11 13 15
Start
Attractor
ICL
FAcore
FANCD2I
NUC1
RNF4
NUC2
DSB
PARP1
RDSB
HRR
KU_53BP1
NHEJ
gH2AX
ATR
ATM
MYT1
WEE1
p53
p21
PP2A_B55
WIP1
CDK1_AurA
PLK1
CDC25
CycB_CDK1
Basin:
4.9 %
rajectory
active
inactive

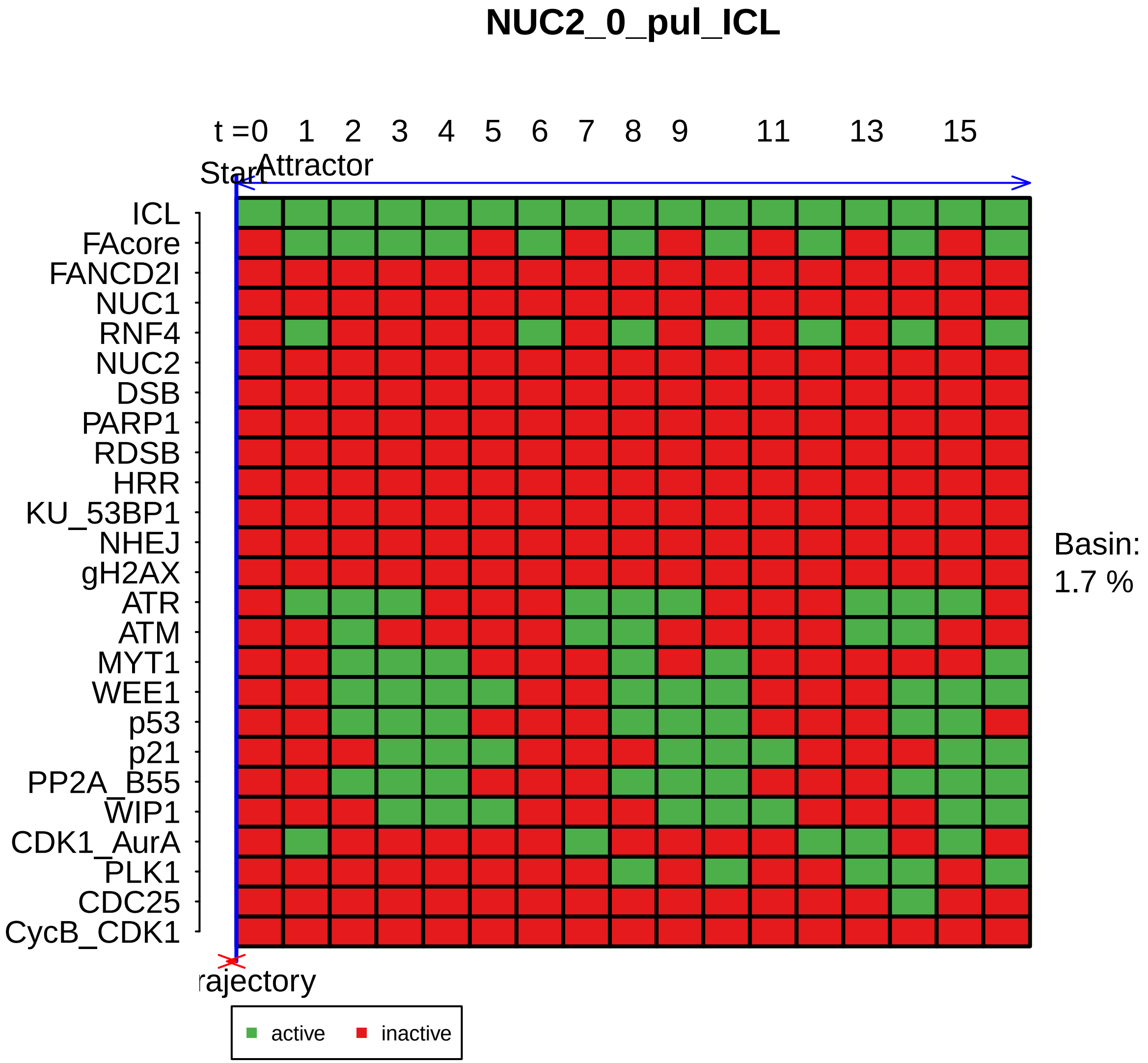

NUC2_0_pul_ICL
t =0 1 2 3 4 5 6 7 8 9 11 13 15
Start
Attractor
ICL
FAcore
FANCD2I
NUC1
RNF4
NUC2
DSB
PARP1
RDSB
HRR
KU_53BP1
NHEJ
gH2AX
ATR
ATM
MYT1
WEE1
p53
p21
PP2A_B55
WIP1
CDK1_AurA
PLK1
CDC25
CycB_CDK1
Basin:
1.7 %
rajectory
active
inactive

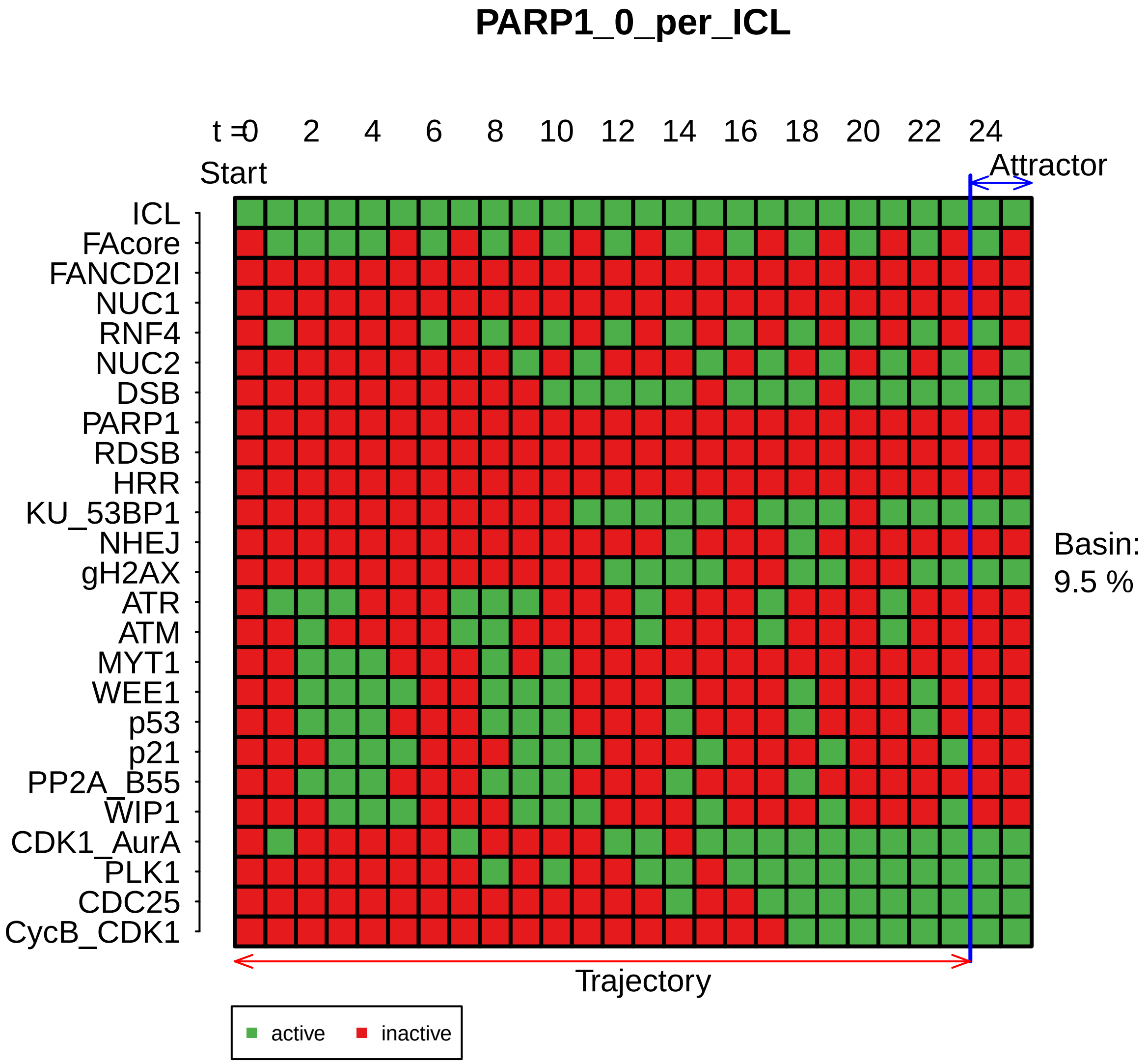
PARP1_0_per_ICL
t =
0
2
4
6
8
10
12
14
16
18
20
22
24
Start
Attractor
ICL
FAcore
FANCD2I
NUC1
RNF4
NUC2
DSB
PARP1
RDSB
HRR
KU_53BP1
NHEJ
gH2AX
ATR
ATM
MYT1
WEE1
p53
p21
PP2A_B55
WIP1
CDK1_AurA
PLK1
CDC25
CycB_CDK1
Basin:
9.5 %
Trajectory
active
inactive

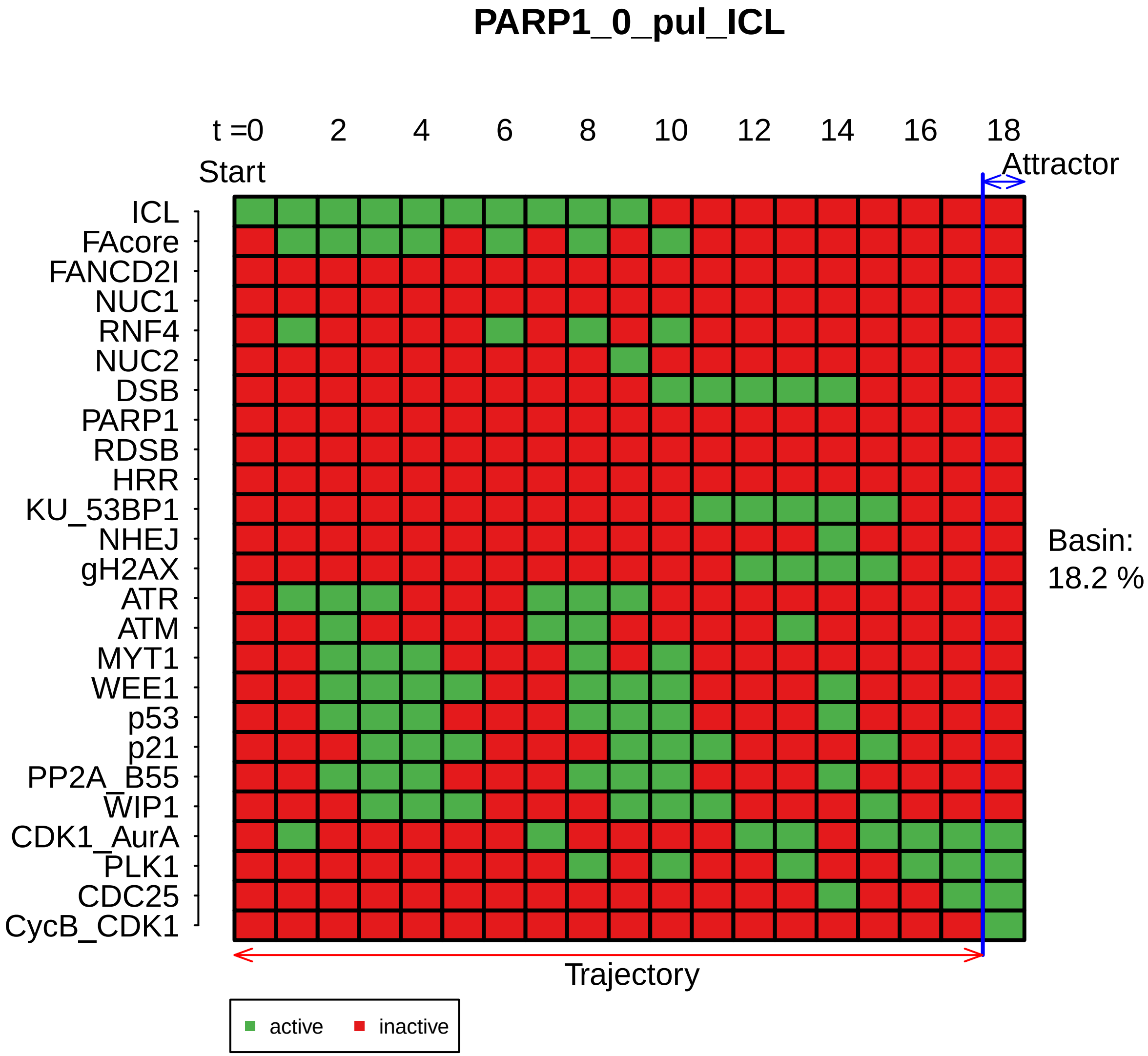
PARP1_0_pul_ICL
t =0
2
4
6
8
10
12
14
16
18
Start
Attractor
ICL
FAcore
FANCD2I
NUC1
RNF4
NUC2
DSB
PARP1
RDSB
HRR
KU_53BP1
NHEJ
gH2AX
ATR
ATM
MYT1
WEE1
p53
p21
PP2A_B55
WIP1
CDK1_AurA
PLK1
CDC25
CycB_CDK1
Basin:
18.2 %
Trajectory
active
inactive

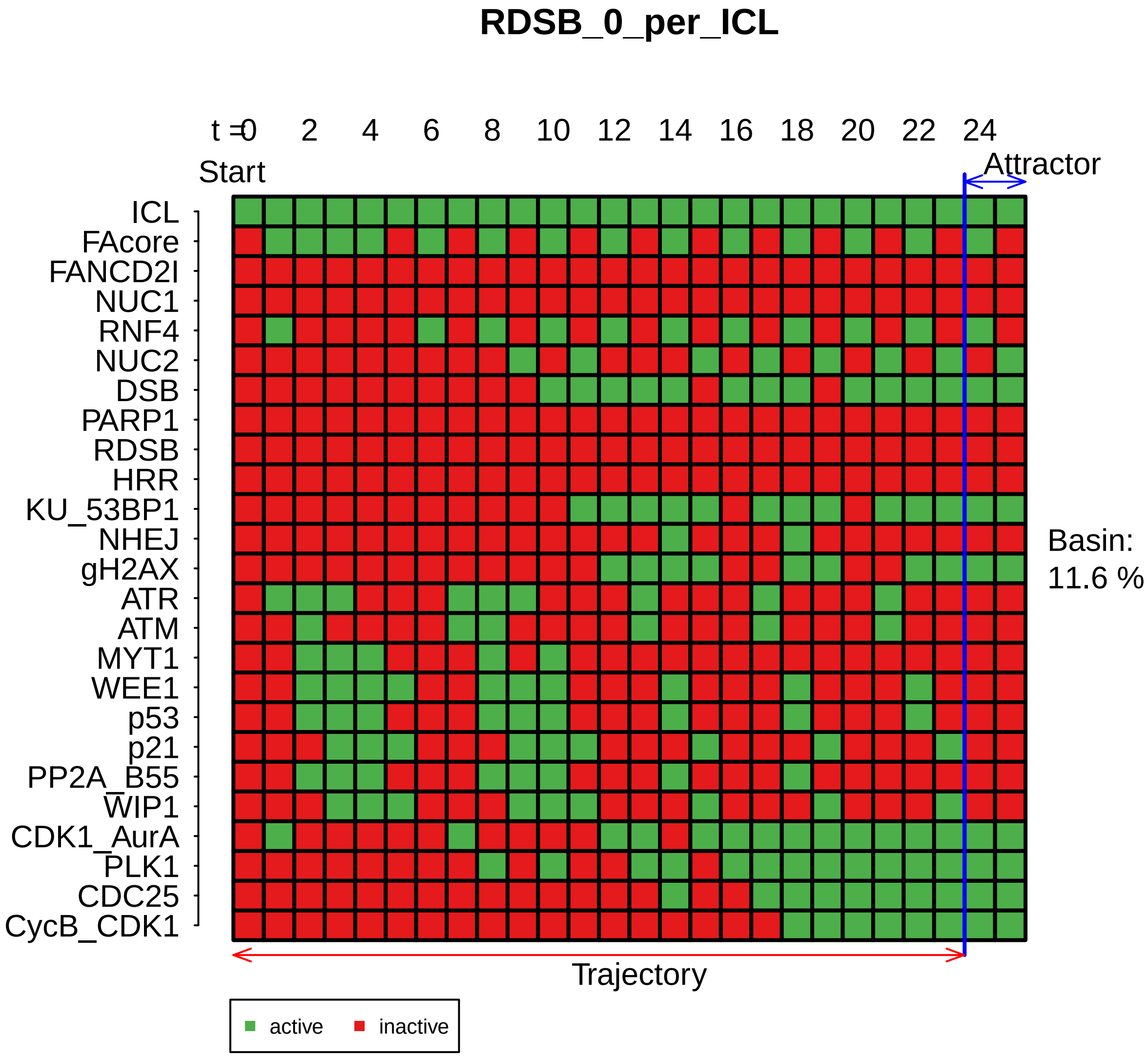

RDSB_0_per_ICL
t = 0 2 4 6 8 10 12 14 16 18 20 22 24
Start
Attractor
ICL
FAcore
FANCD2I
NUC1
RNF4
NUC2
DSB
PARP1
RDSB
HRR
KU_53BP1
NHEJ
gH2AX
ATR
ATM
MYT1
WEE1
p53
p21
PP2A_B55
WIP1
CDK1_AurA
PLK1
CDC25
CycB_CDK1
Basin:
11.6 %
Trajectory
active
inactive

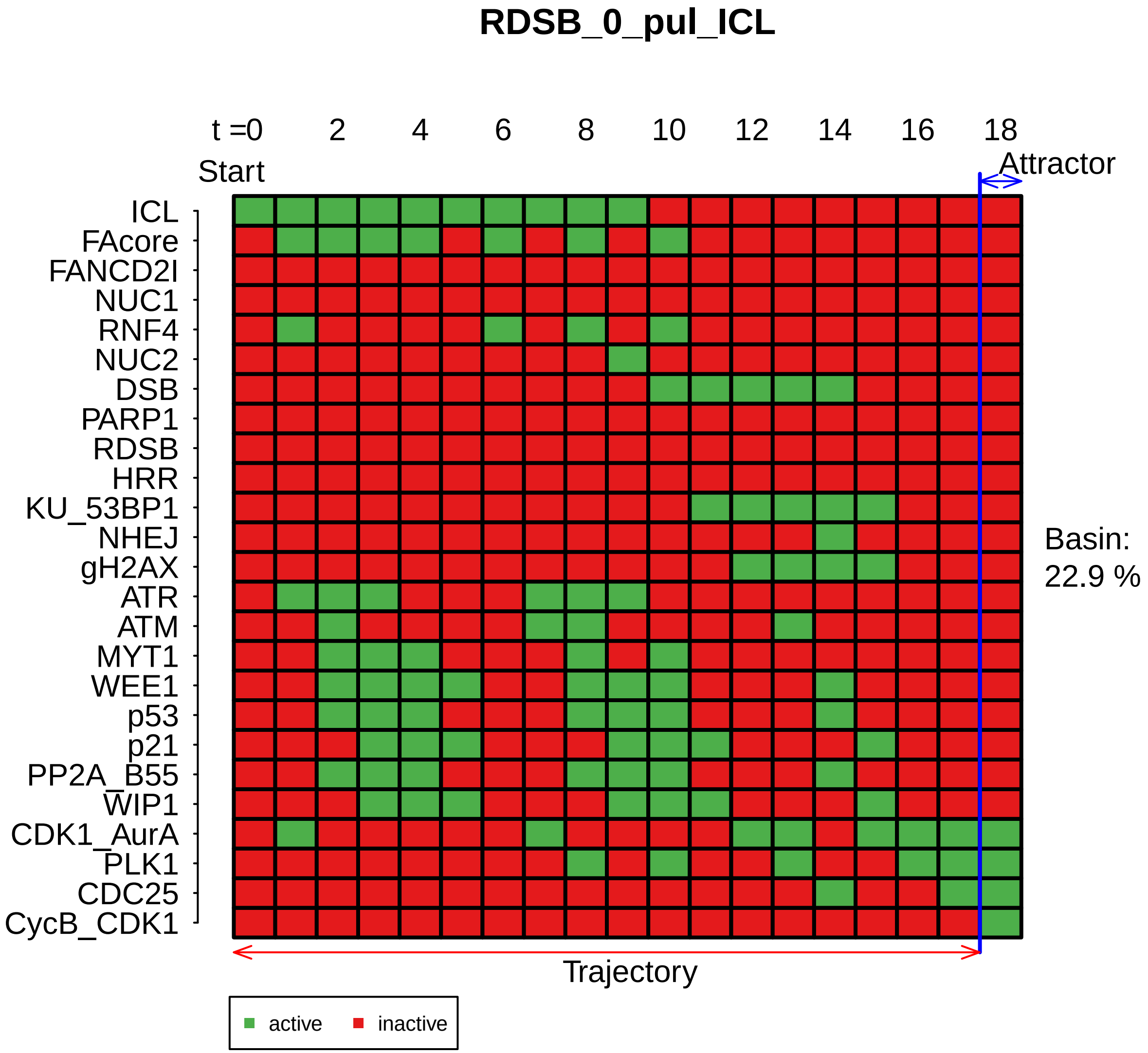
RDSB_0_pul_ICL
t =0
2
4
6
8
10
12
14
16
18
Start
Attractor
ICL
FAcore
FANCD2I
NUC1
RNF4
NUC2
DSB
PARP1
RDSB
HRR
KU_53BP1
NHEJ
gH2AX
ATR
ATM
MYT1
WEE1
p53
p21
PP2A_B55
WIP1
CDK1_AurA
PLK1
CDC25
CycB_CDK1
Basin:
22.9 %
Trajectory
active
inactive

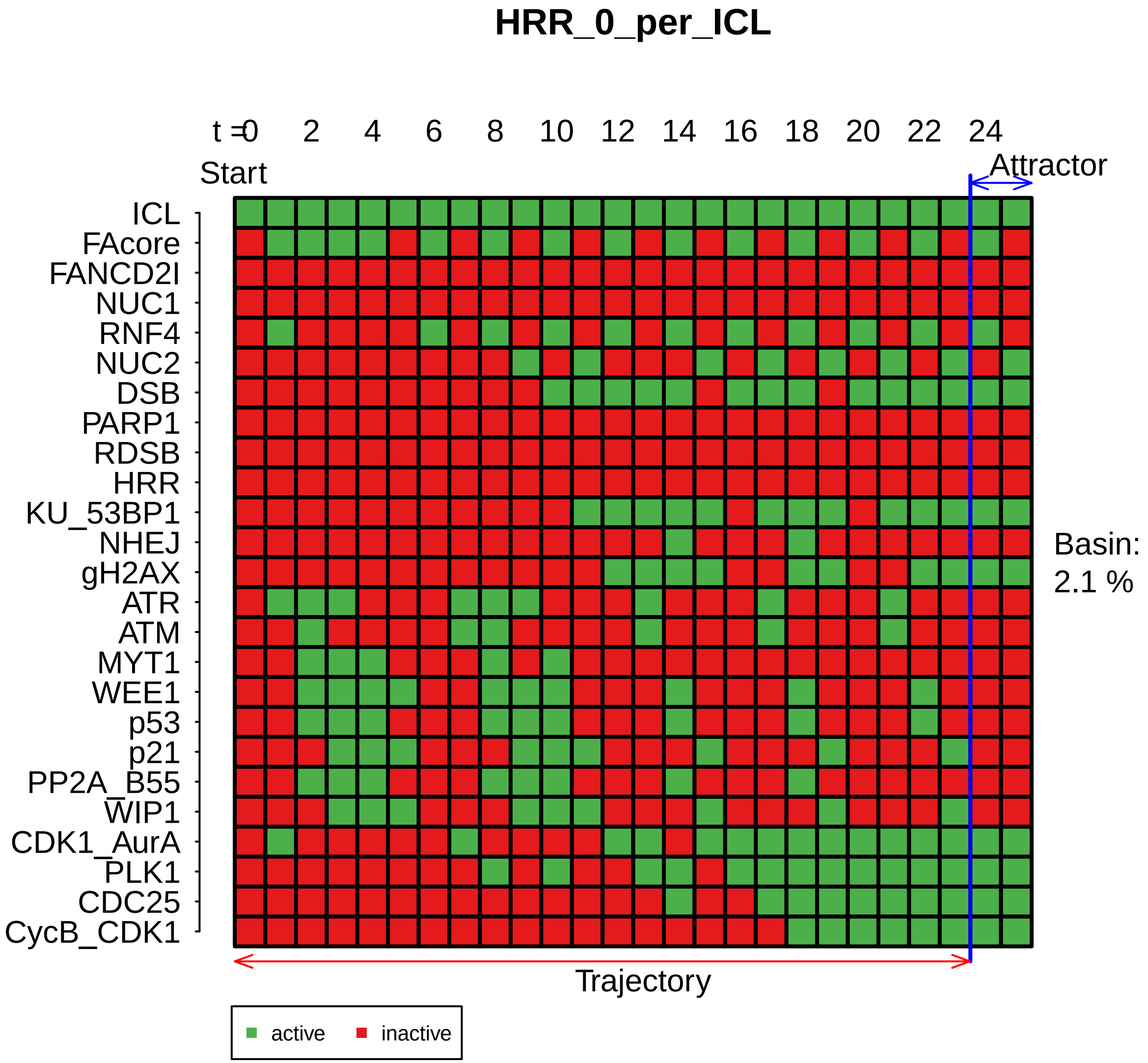
HRR_0_per_ICL
t = 0
2
4
6
8
10
12
14
16
18
20
22
24
Start
Attractor
ICL
FAcore
FANCD2I
NUC1
RNF4
NUC2
DSB
PARP1
RDSB
HRR
KU_53BP1
NHEJ
gH2AX
ATR
ATM
MYT1
WEE1
p53
p21
PP2A_B55
WIP1
CDK1_AurA
PLK1
CDC25
CycB_CDK1
Basin:
2.1 %
Trajectory
active
inactive

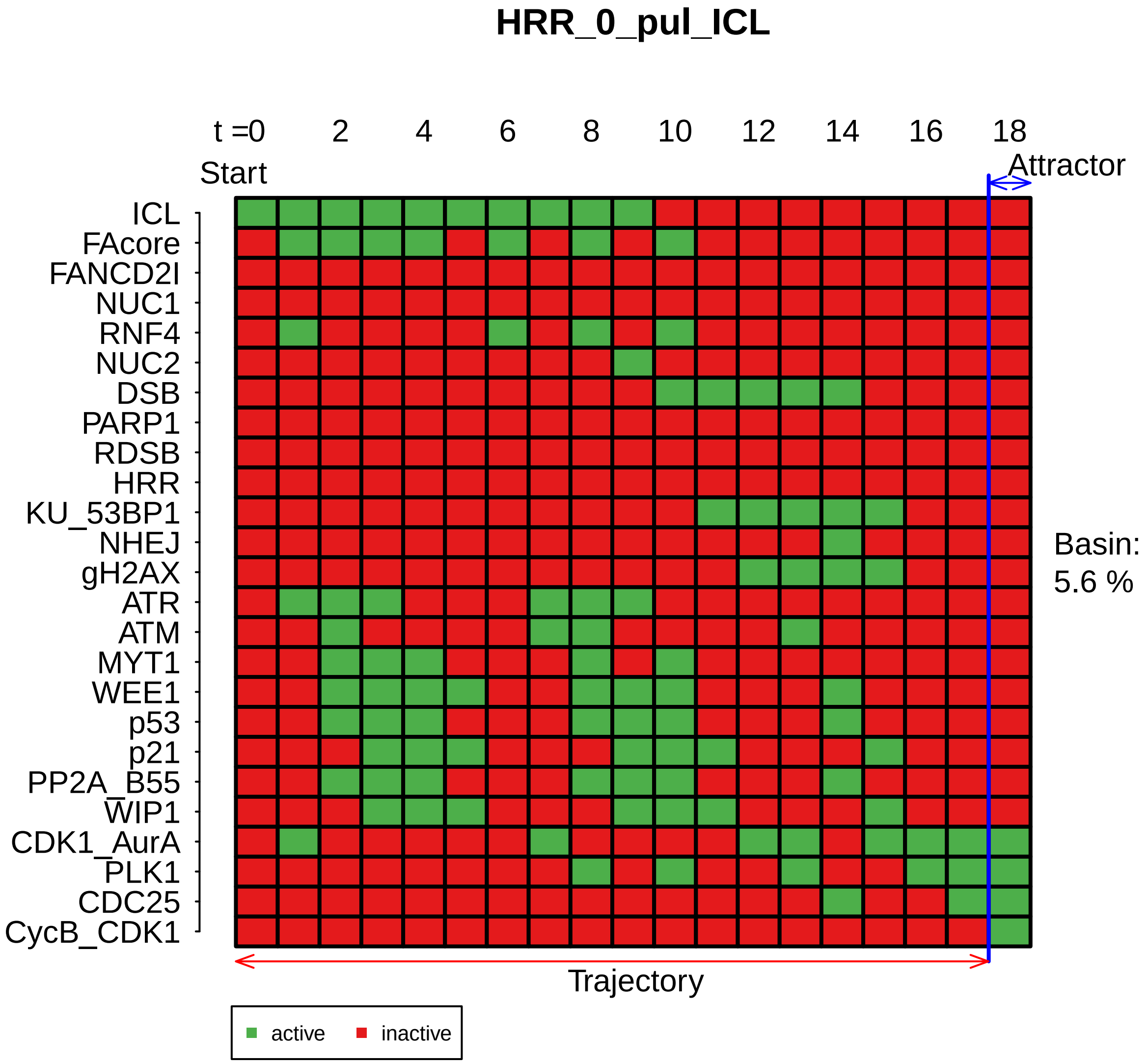
HRR_0_pul_ICL
t =0
2
4
6
8
10
12
14
16
18
Start
Attractor
ICL
FAcore
FANCD2I
NUC1
RNF4
NUC2
DSB
PARP1
RDSB
HRR
KU_53BP1
NHEJ
gH2AX
ATR
ATM
MYT1
WEE1
p53
p21
PP2A_B55
WIP1
CDK1_AurA
PLK1
CDC25
CycB_CDK1
Basin:
5.6 %
Trajectory
active
inactive

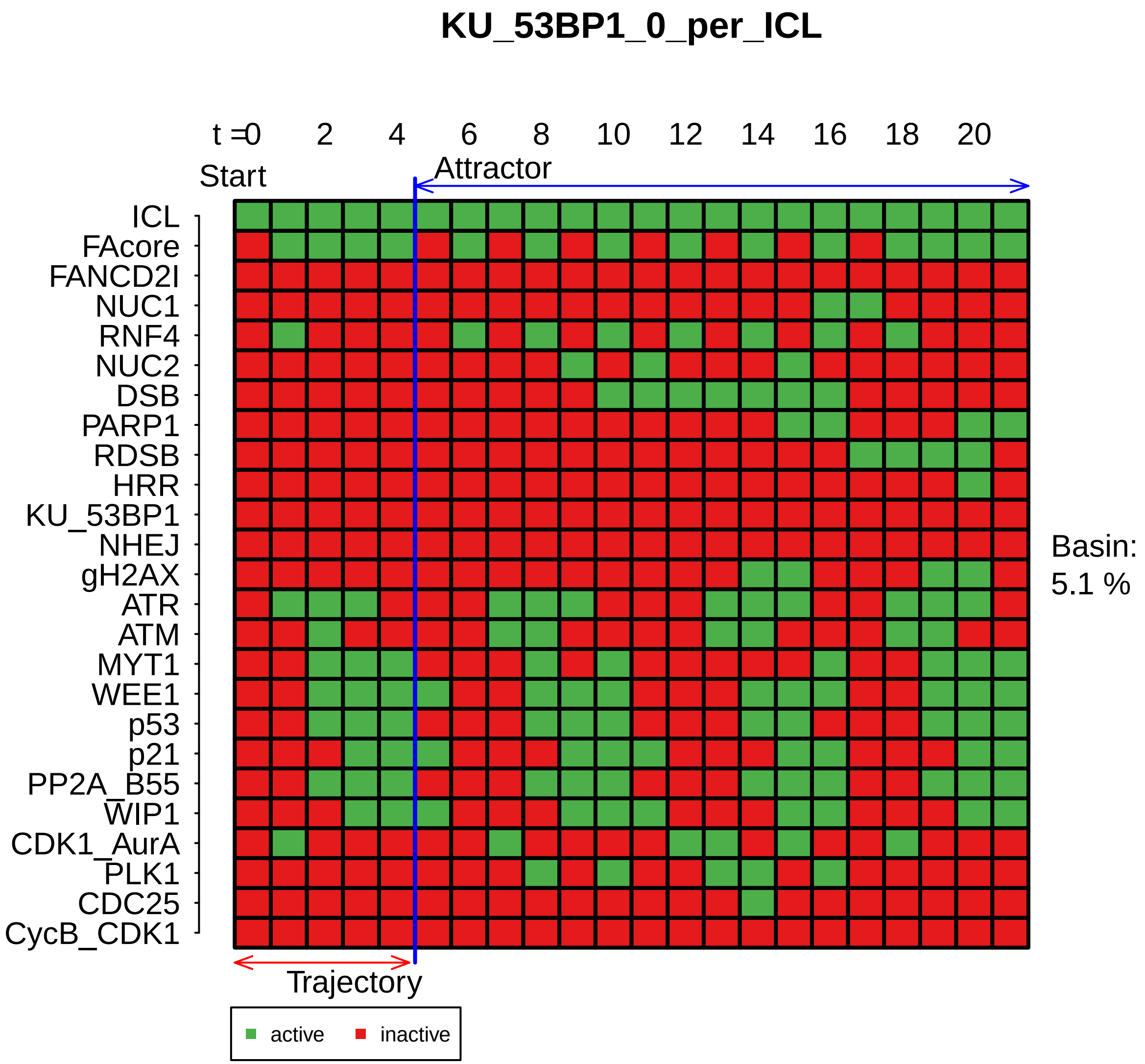
KU_53BP1_0_per_ICL
t = 0
2
4
6
8
10
12
14
16
18
20
Start
Attractor
ICL
FAcore
FANCD2I
NUC1
RNF4
NUC2
DSB
PARP1
RDSB
HRR
KU_53BP1
NHEJ
gH2AX
ATR
ATM
MYT1
WEE1
p53
p21
PP2A_B55
WIP1
CDK1_AurA
PLK1
CDC25
CycB_CDK1
Basin:
5.1 %
Trajectory
active
inactive

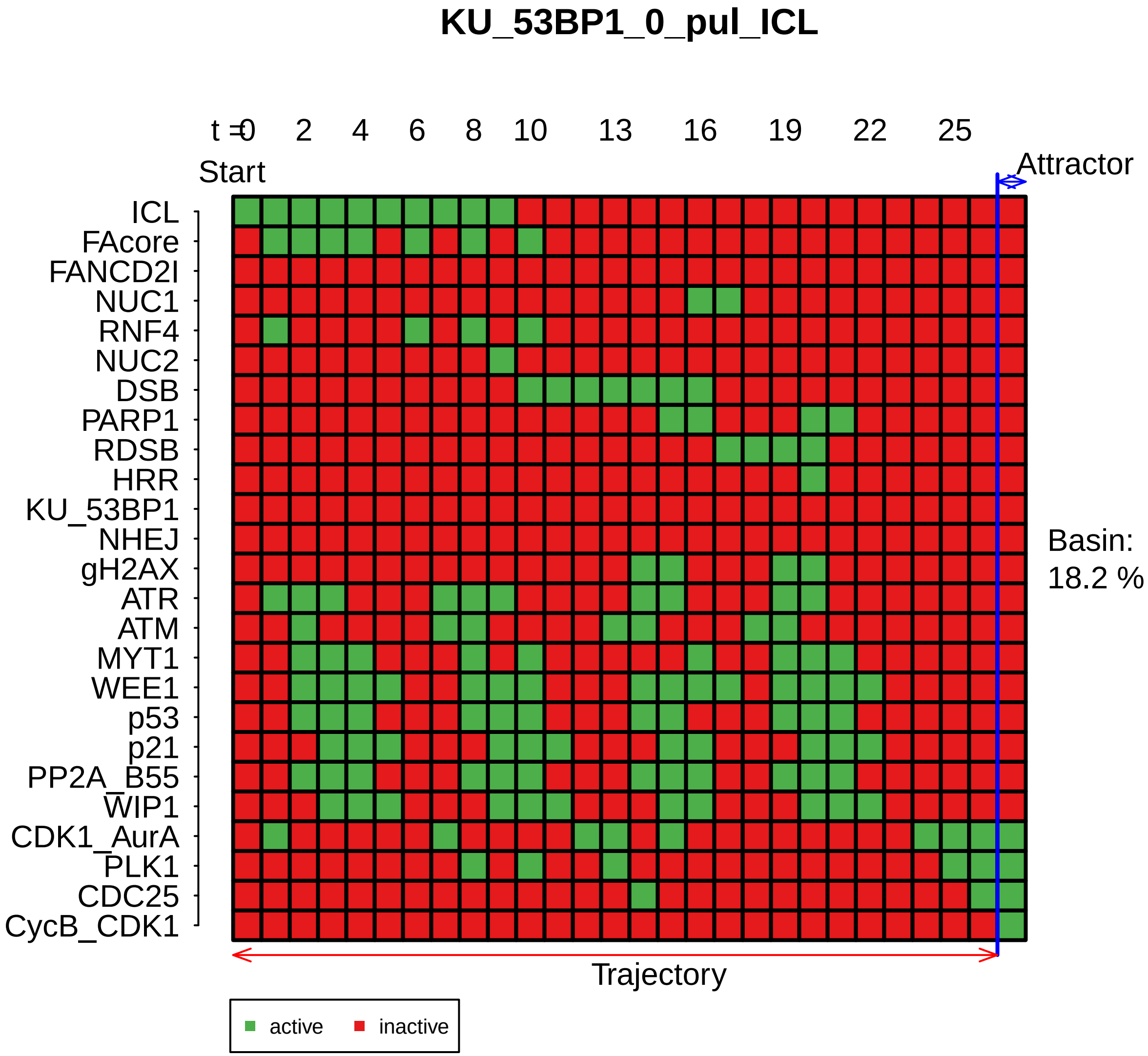
KU_53BP1_0_pul_ICL
t = 0 2 4 6 8 10 13 16 19 22 25
Start
Attractor
ICL
FAcore
FANCD2I
NUC1
RNF4
NUC2
DSB
PARP1
RDSB
HRR
KU_53BP1
NHEJ
gH2AX
ATR
ATM
MYT1
WEE1
p53
p21
PP2A_B55
WIP1
CDK1_AurA
PLK1
CDC25
CycB_CDK1
Basin:
18.2 %
Trajectory
active
inactive

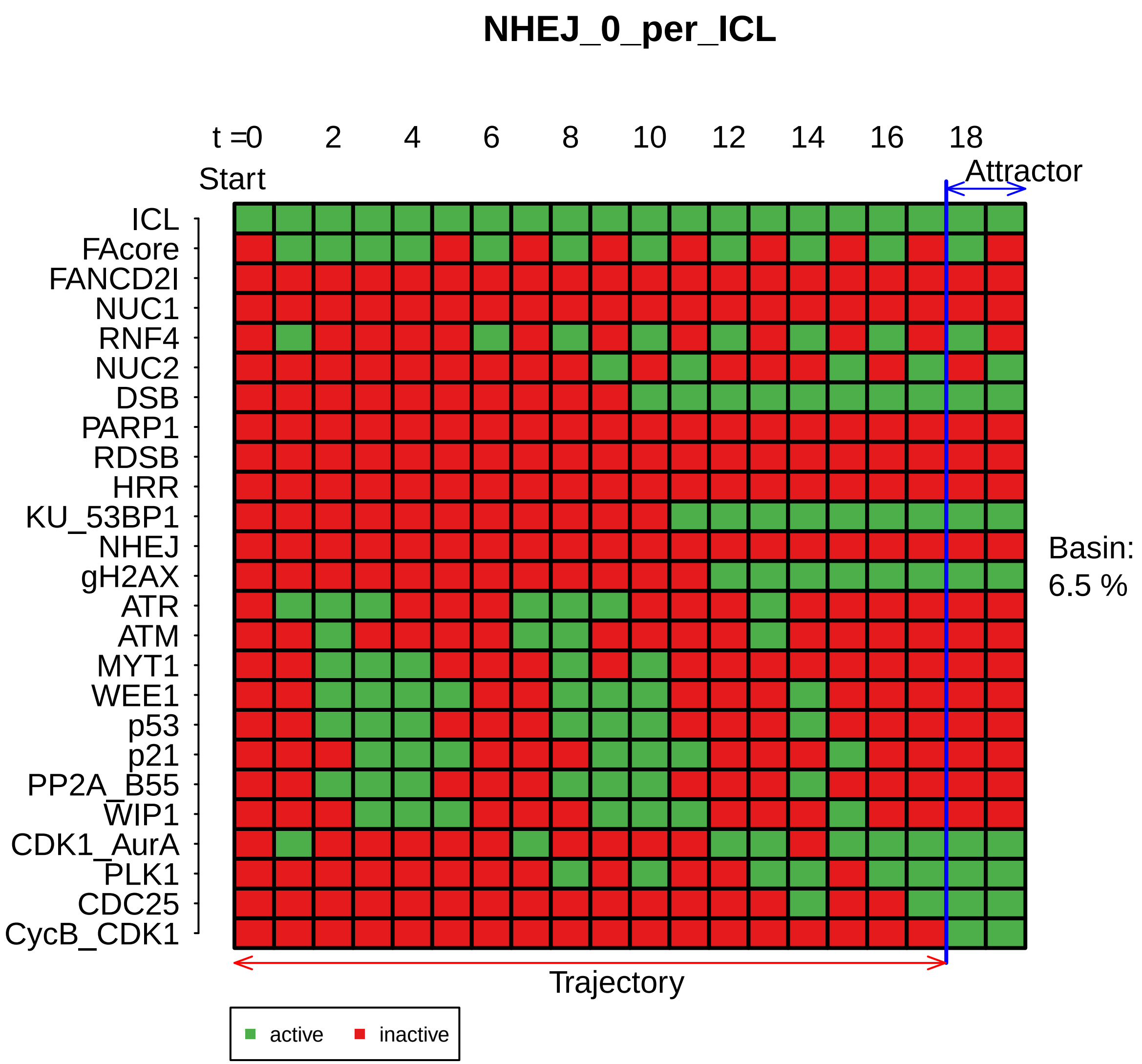

NHEJ_0_per_ICL
t =0
2
4
6
8
10
12
14
16
18
Start
Attractor
ICL
FAcore
FANCD2I
NUC1
RNF4
NUC2
DSB
PARP1
RDSB
HRR
KU_53BP1
NHEJ
gH2AX
ATR
ATM
MYT1
WEE1
p53
p21
PP2A_B55
WIP1
CDK1_AurA
PLK1
CDC25
CycB_CDK1
Basin:
6.5 %
Trajectory
active
inactive

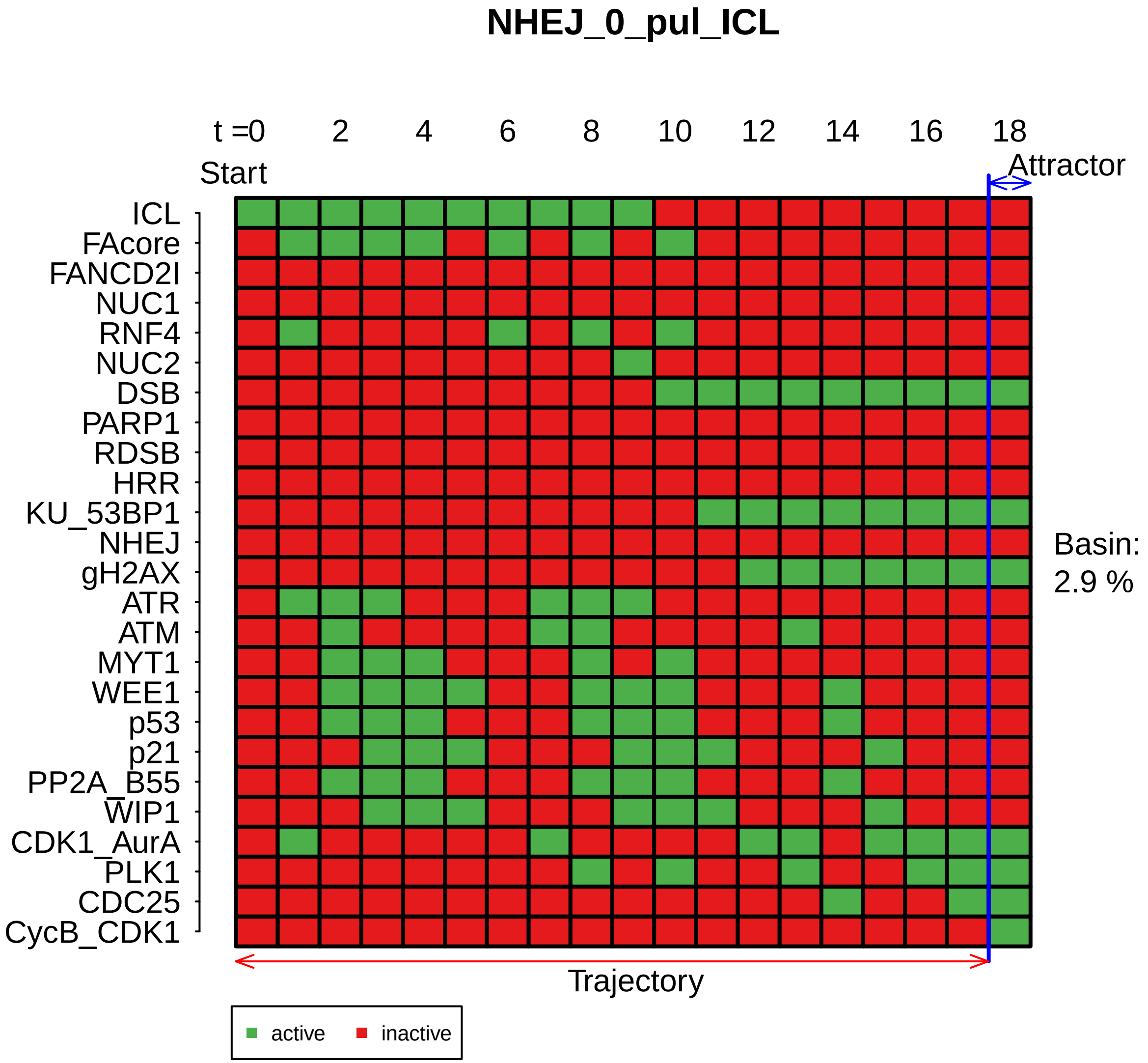
NHEJ_0_pul_ICL
t =0
2
4
6
8
10
12
14
16
18
Start
Attractor
ICL
FAcore
FANCD2I
NUC1
RNF4
NUC2
DSB
PARP1
RDSB
HRR
KU_53BP1
NHEJ
gH2AX
ATR
ATM
MYT1
WEE1
p53
p21
PP2A_B55
WIP1
CDK1_AurA
PLK1
CDC25
CycB_CDK1
Basin:
2.9 %
Trajectory
active
inactive

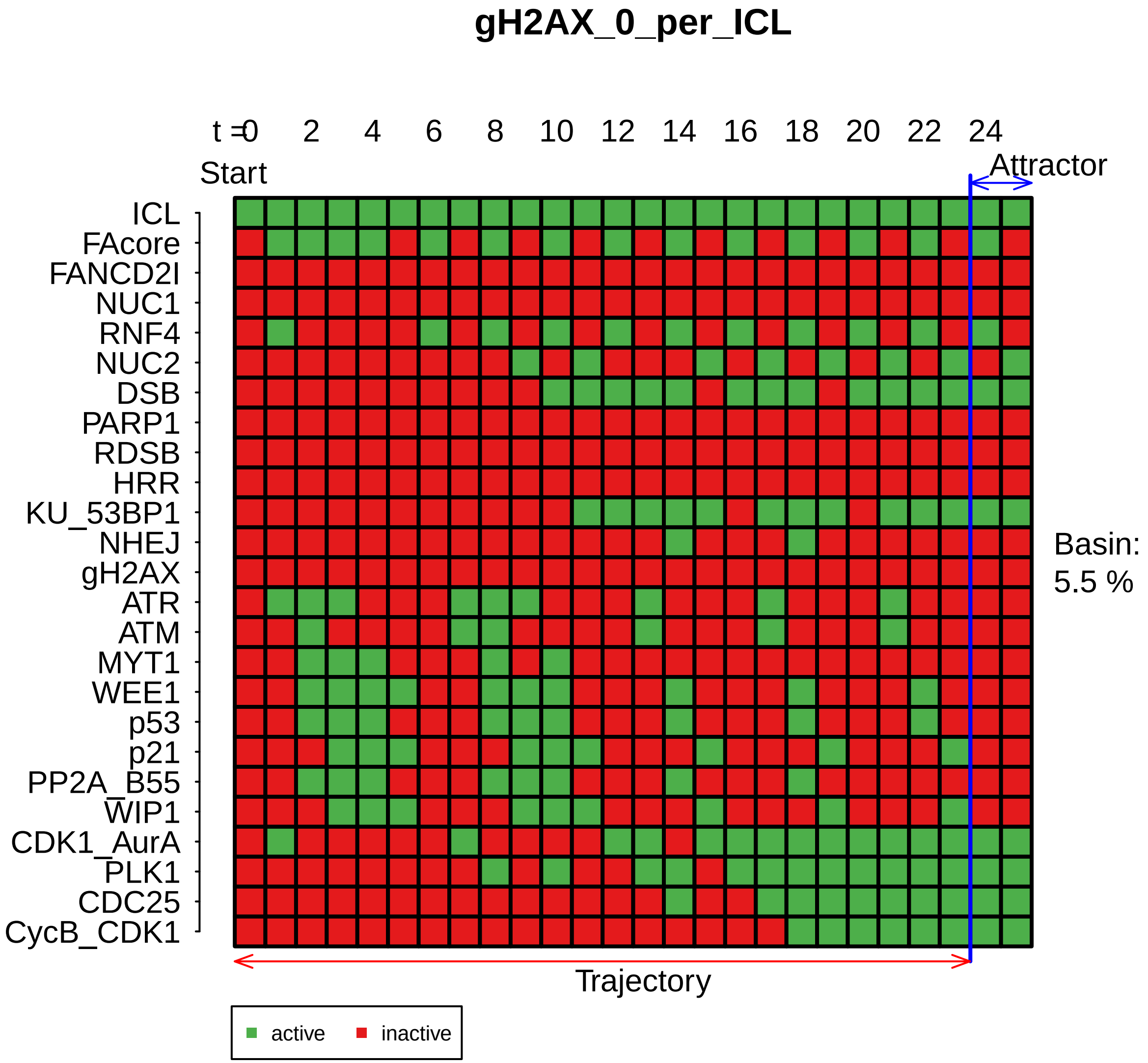

gH2AX_0_per_ICL
t = 0
2
4
6
8
10
12
14
16
18
20
22
24
Start
Attractor
ICL
FAcore
FANCD2I
NUC1
RNF4
NUC2
DSB
PARP1
RDSB
HRR
KU_53BP1
NHEJ
gH2AX
ATR
ATM
MYT1
WEE1
p53
p21
PP2A_B55
WIP1
CDK1_AurA
PLK1
CDC25
CycB_CDK1
Basin:
5.5 %
Trajectory
active
inactive

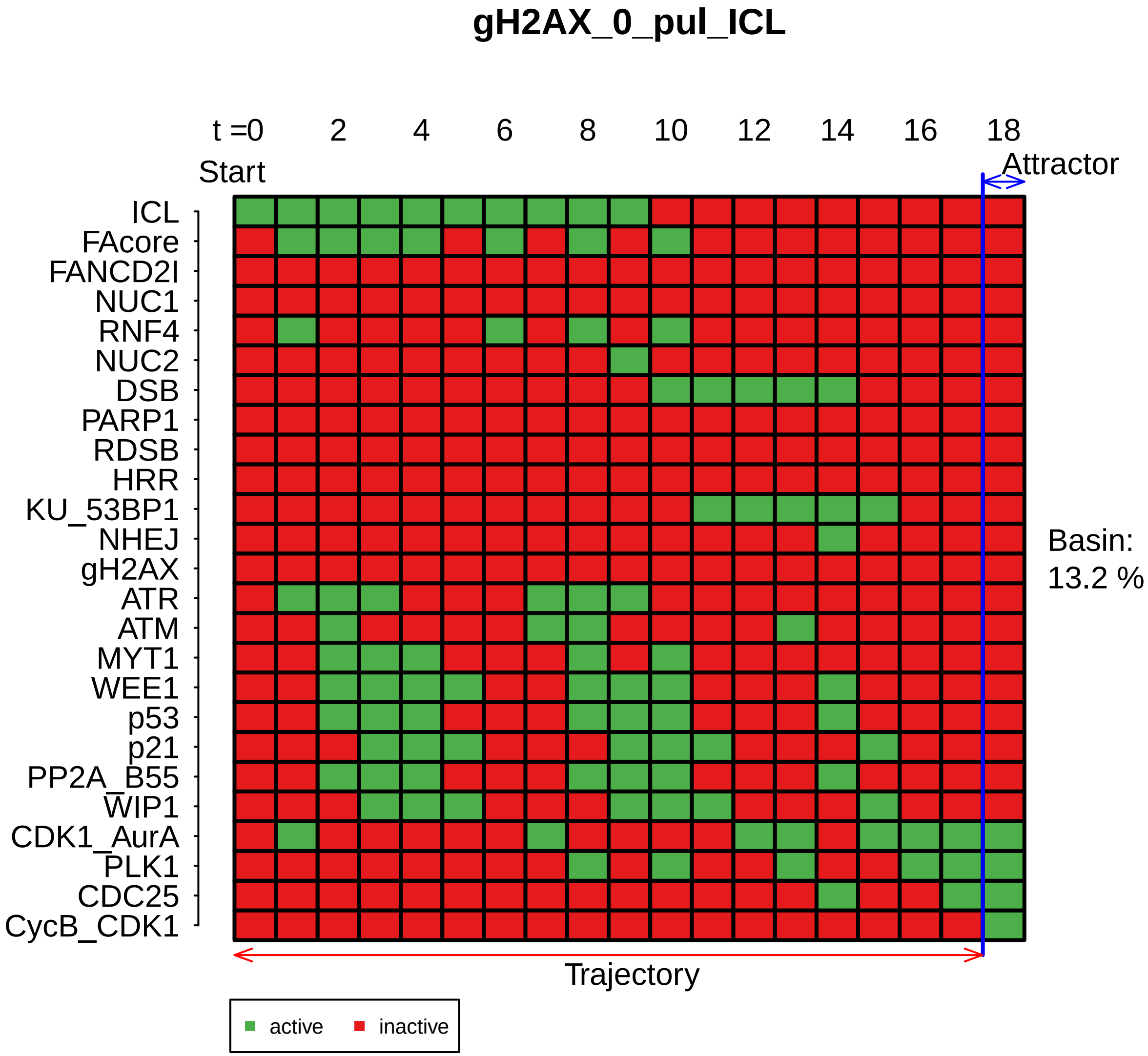
gH2AX_0_pul_ICL
t =0
2
4
6
8
10
12
14
16
18
Start
Attractor
ICL
FAcore
FANCD2I
NUC1
RNF4
NUC2
DSB
PARP1
RDSB
HRR
KU_53BP1
NHEJ
gH2AX
ATR
ATM
MYT1
WEE1
p53
p21
PP2A_B55
WIP1
CDK1_AurA
PLK1
CDC25
CycB_CDK1
Basin:
13.2 %
Trajectory
active
inactive

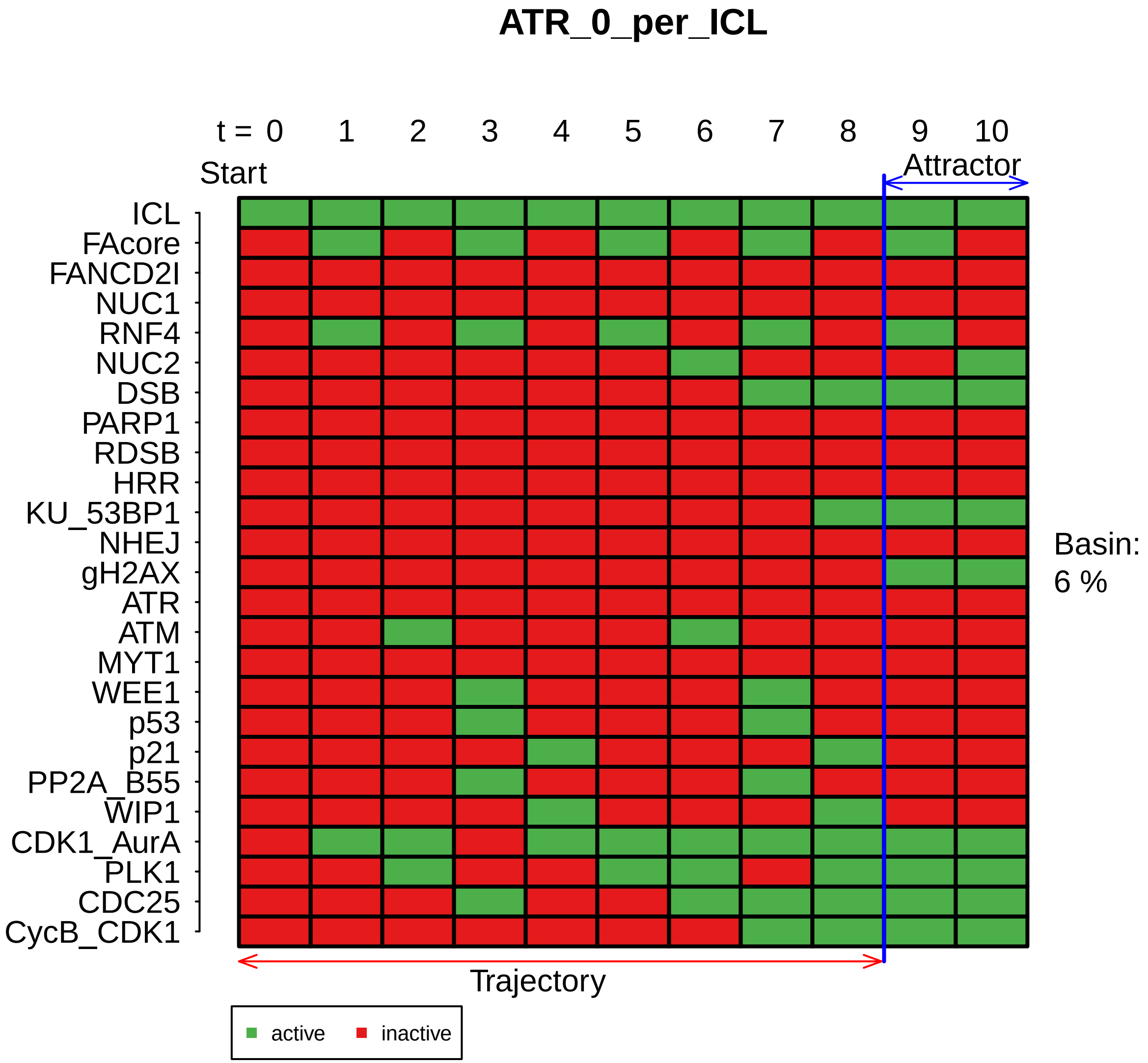
ATR_0_per_ICL
t = 0
1
2
3
4
5
6
7
8
9
10
Start
Attractor
ICL
FAcore
FANCD2I
NUC1
RNF4
NUC2
DSB
PARP1
RDSB
HRR
KU_53BP1
NHEJ
gH2AX
ATR
ATM
MYT1
WEE1
p53
p21
PP2A_B55
WIP1
CDK1_AurA
PLK1
CDC25
CycB_CDK1
Basin:
6 %
Trajectory
active
inactive

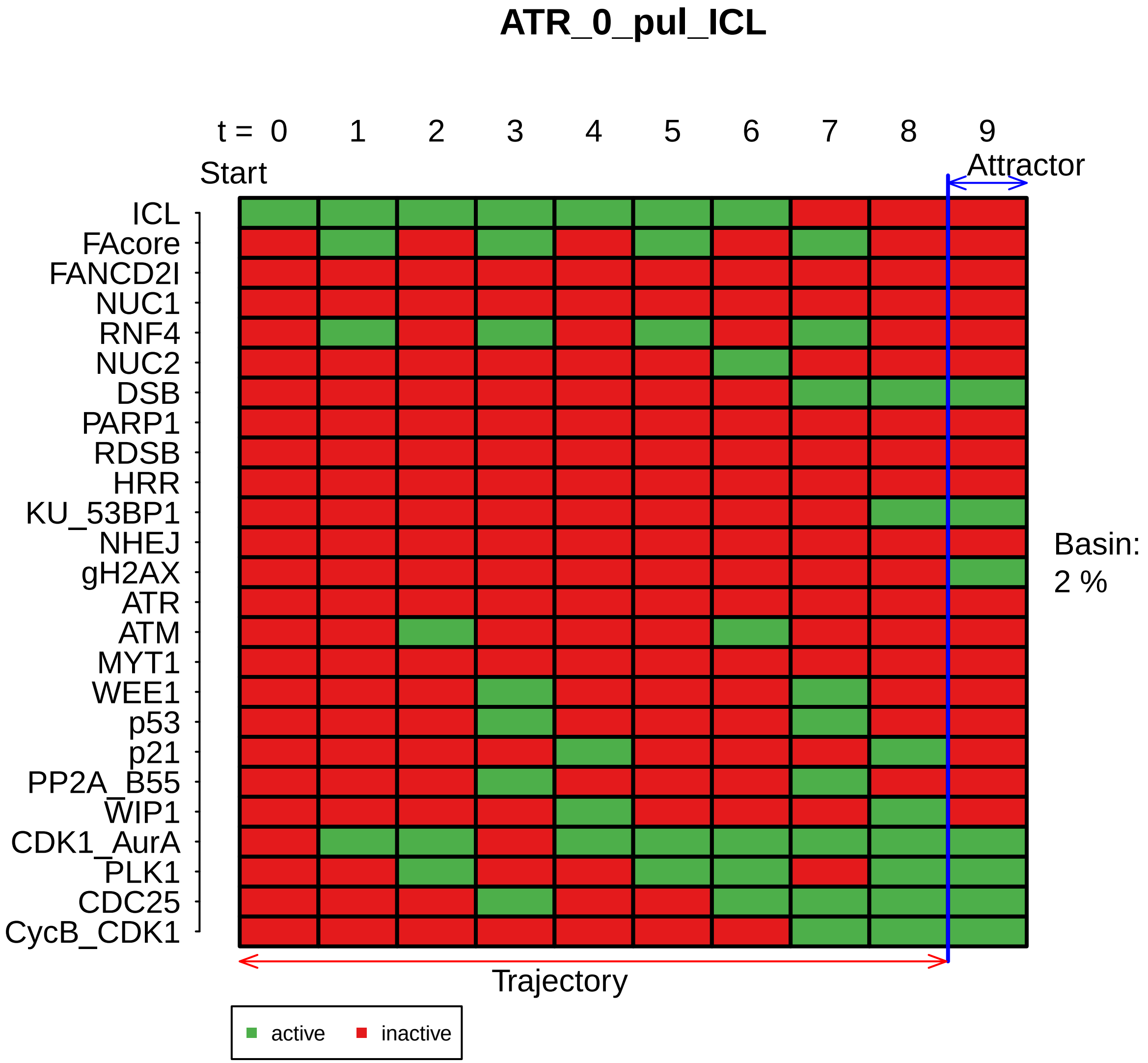

ATR_0_pul_ICL
t = 0 1 2 3 4 5 6 7 8 9
Start
Attractor
ICL
FAcore
FANCD2I
NUC1
RNF4
NUC2
DSB
PARP1
RDSB
HRR
KU_53BP1
NHEJ
gH2AX
ATR
ATM
MYT1
WEE1
p53
p21
PP2A_B55
WIP1
CDK1_AurA
PLK1
CDC25
CycB_CDK1
Basin:
2 %
Trajectory
active
inactive

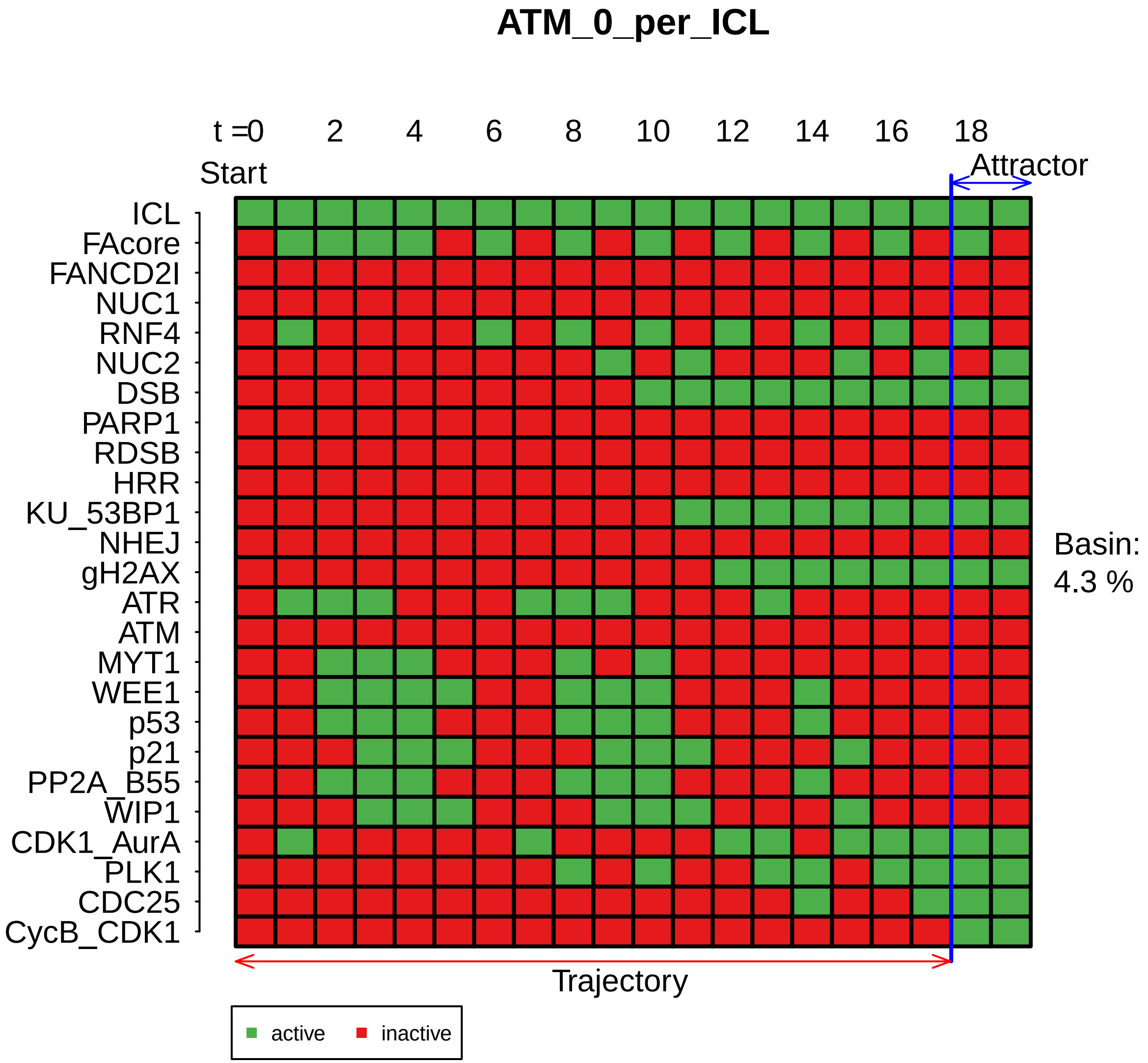
ATM_0_per_ICL
t =0
2
4
6
8
10
12
14
16
18
Start
Attractor
ICL
FAcore
FANCD2I
NUC1
RNF4
NUC2
DSB
PARP1
RDSB
HRR
KU_53BP1
NHEJ
gH2AX
ATR
ATM
MYT1
WEE1
p53
p21
PP2A_B55
WIP1
CDK1_AurA
PLK1
CDC25
CycB_CDK1
Basin:
4.3 %
Trajectory
active
inactive

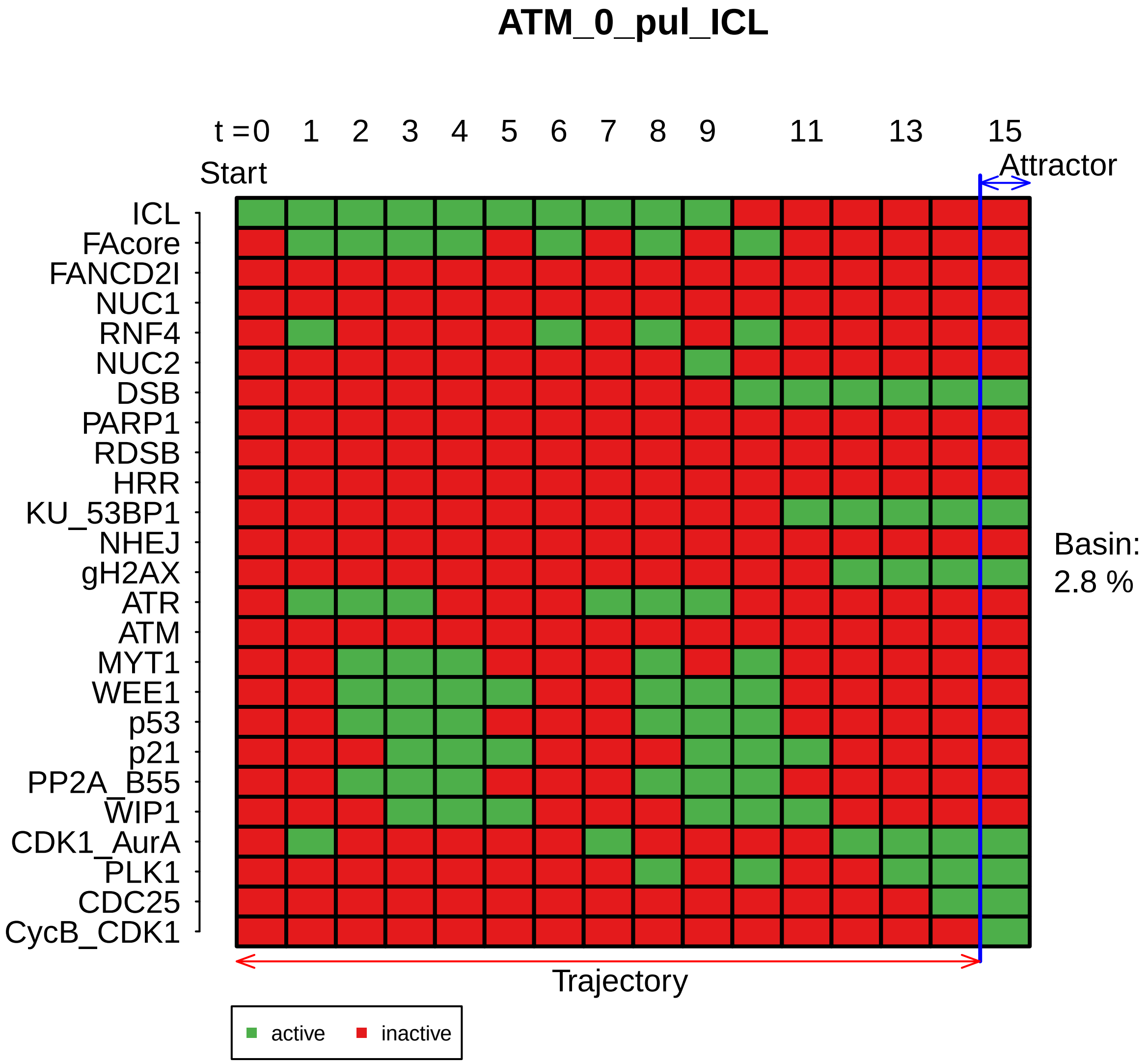

ATM_0_pul_ICL
t =0 1 2 3 4 5 6 7 8 9 11 13 15
Start
Attractor
ICL
FAcore
FANCD2I
NUC1
RNF4
NUC2
DSB
PARP1
RDSB
HRR
KU_53BP1
NHEJ
gH2AX
ATR
ATM
MYT1
WEE1
p53
p21
PP2A_B55
WIP1
CDK1_AurA
PLK1
CDC25
CycB_CDK1
Basin:
2.8 %
Trajectory
active
inactive

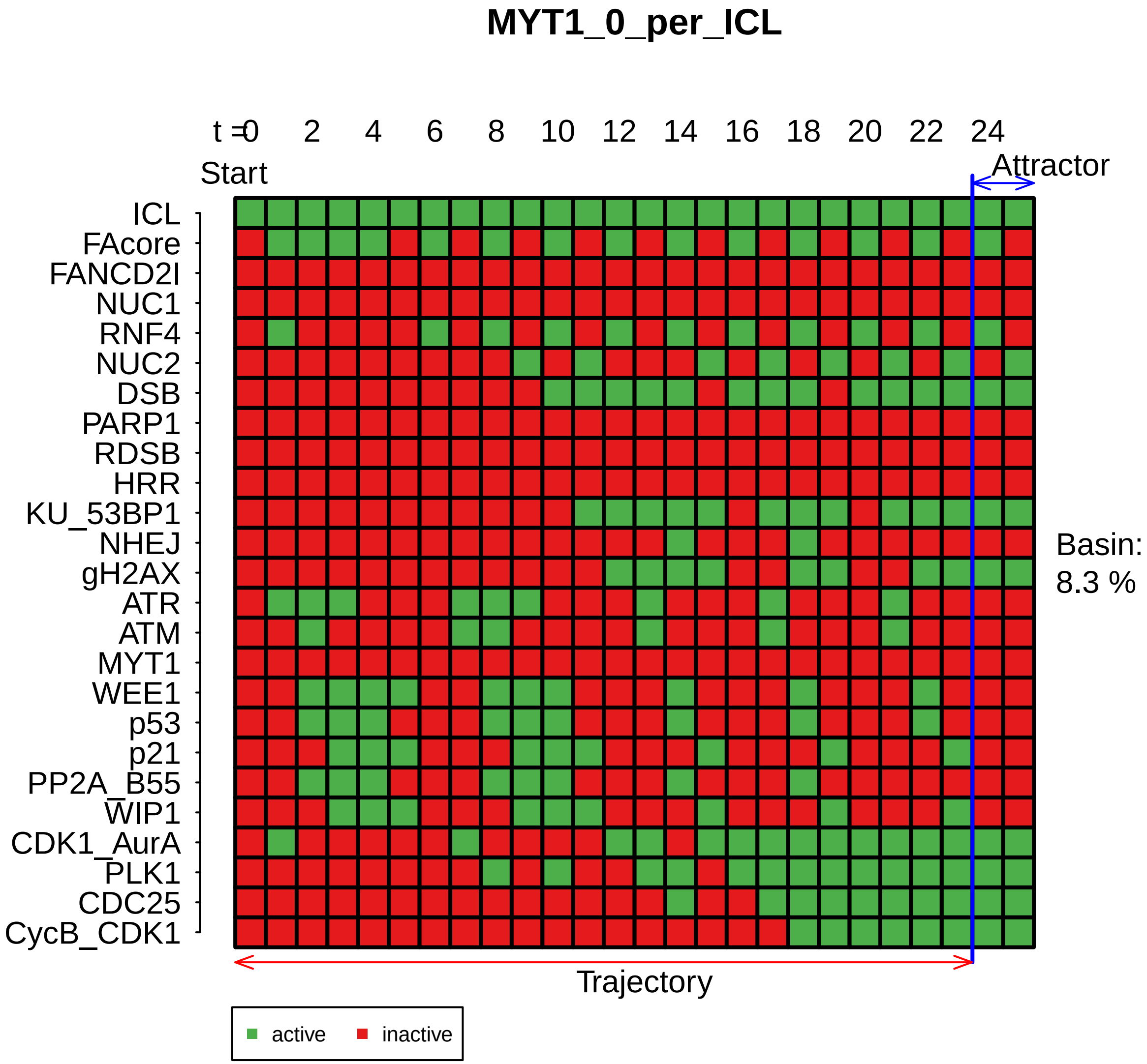

MYT1_0_per_ICL
t = 0 2 4 6 8 10 12 14 16 18 20 22 24
Start
Attractor
ICL
FAcore
FANCD2I
NUC1
RNF4
NUC2
DSB
PARP1
RDSB
HRR
KU_53BP1
NHEJ
gH2AX
ATR
ATM
MYT1
WEE1
p53
p21
PP2A_B55
WIP1
CDK1_AurA
PLK1
CDC25
CycB_CDK1
Basin:
8.3 %
Trajectory
active
inactive

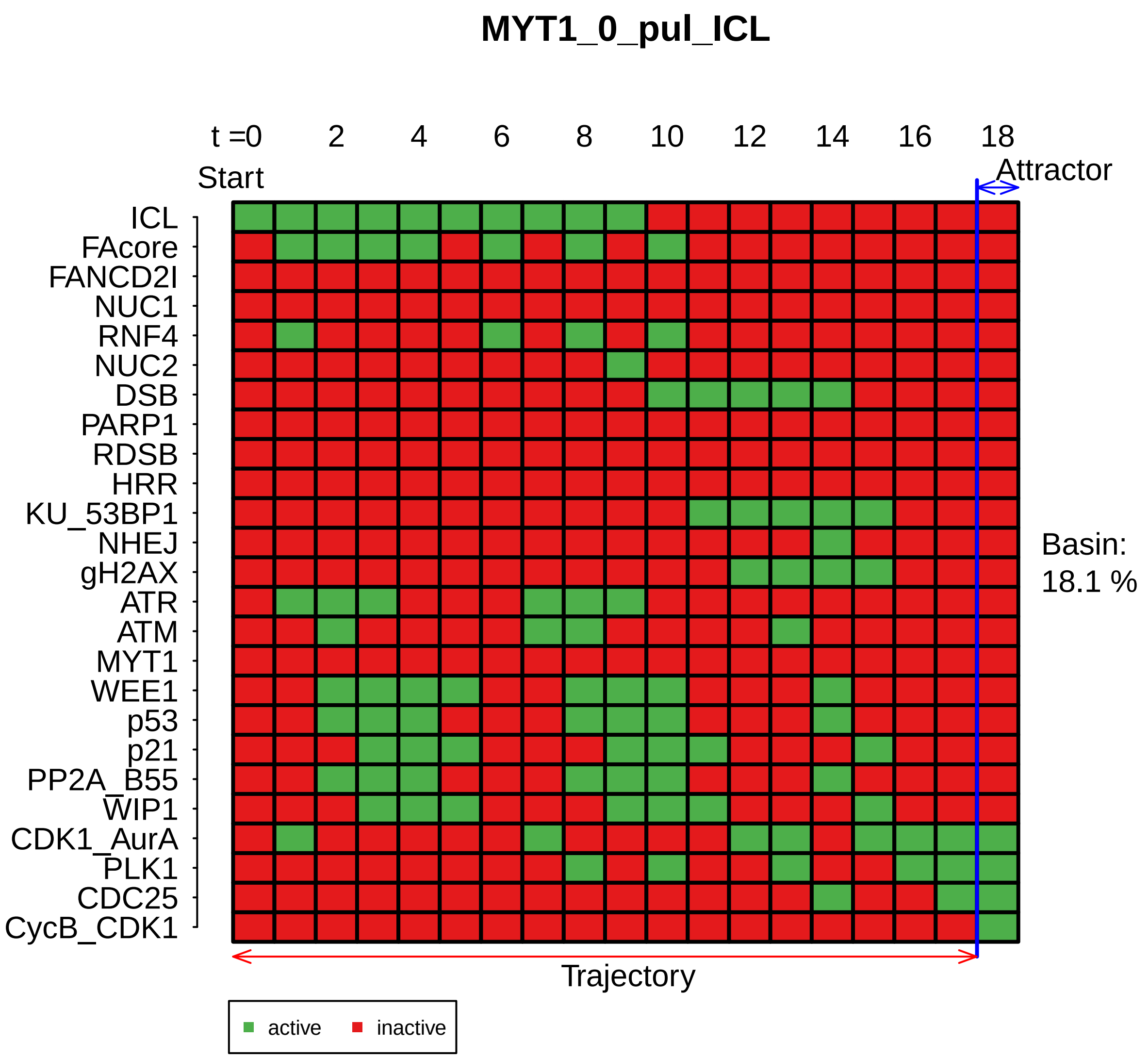

MYT1_0_pul_ICL
t =0
2
4
6
8
10
12
14
16
18
Start
Attractor
ICL
FAcore
FANCD2I
NUC1
RNF4
NUC2
DSB
PARP1
RDSB
HRR
KU_53BP1
NHEJ
gH2AX
ATR
ATM
MYT1
WEE1
p53
p21
PP2A_B55
WIP1
CDK1_AurA
PLK1
CDC25
CycB_CDK1
Basin:
18.1 %
Trajectory
active
inactive

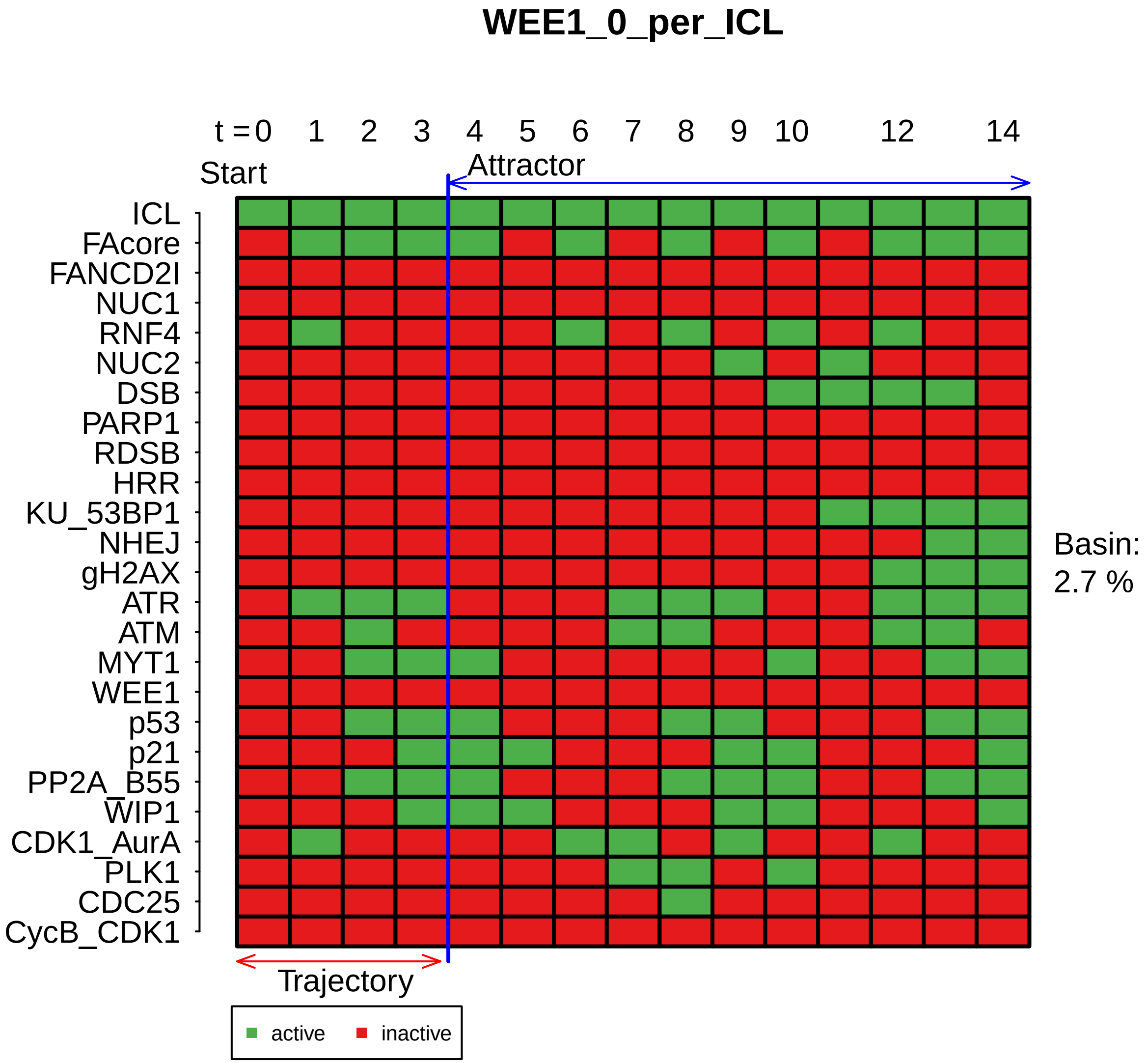
WEE1_0_per_ICL
t = 0 1 2 3 4 5 6 7 8 9 10 12 14
Start
Attractor
ICL
FAcore
FANCD2I
NUC1
RNF4
NUC2
DSB
PARP1
RDSB
HRR
KU_53BP1
NHEJ
gH2AX
ATR
ATM
MYT1
WEE1
p53
p21
PP2A_B55
WIP1
CDK1_AurA
PLK1
CDC25
CycB_CDK1
Basin:
2.7 %
Trajectory
active
inactive

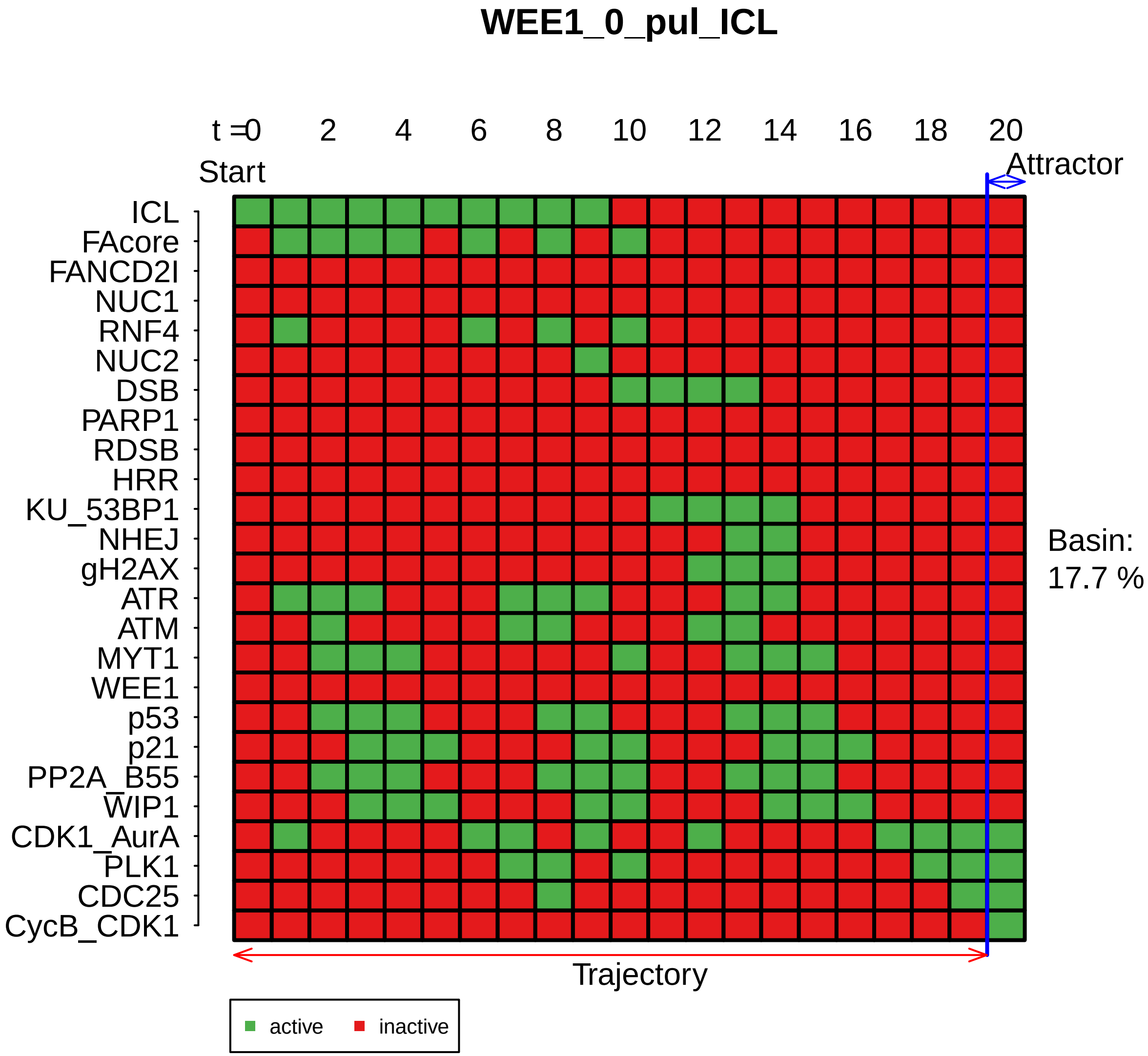

WEE1_0_pul_ICL
t = 0
2
4
6
8
10
12
14
16
18
20
Start
Attractor
ICL
FAcore
FANCD2I
NUC1
RNF4
NUC2
DSB
PARP1
RDSB
HRR
KU_53BP1
NHEJ
gH2AX
ATR
ATM
MYT1
WEE1
p53
p21
PP2A_B55
WIP1
CDK1_AurA
PLK1
CDC25
CycB_CDK1
Basin:
17.7 %
Trajectory
active
inactive

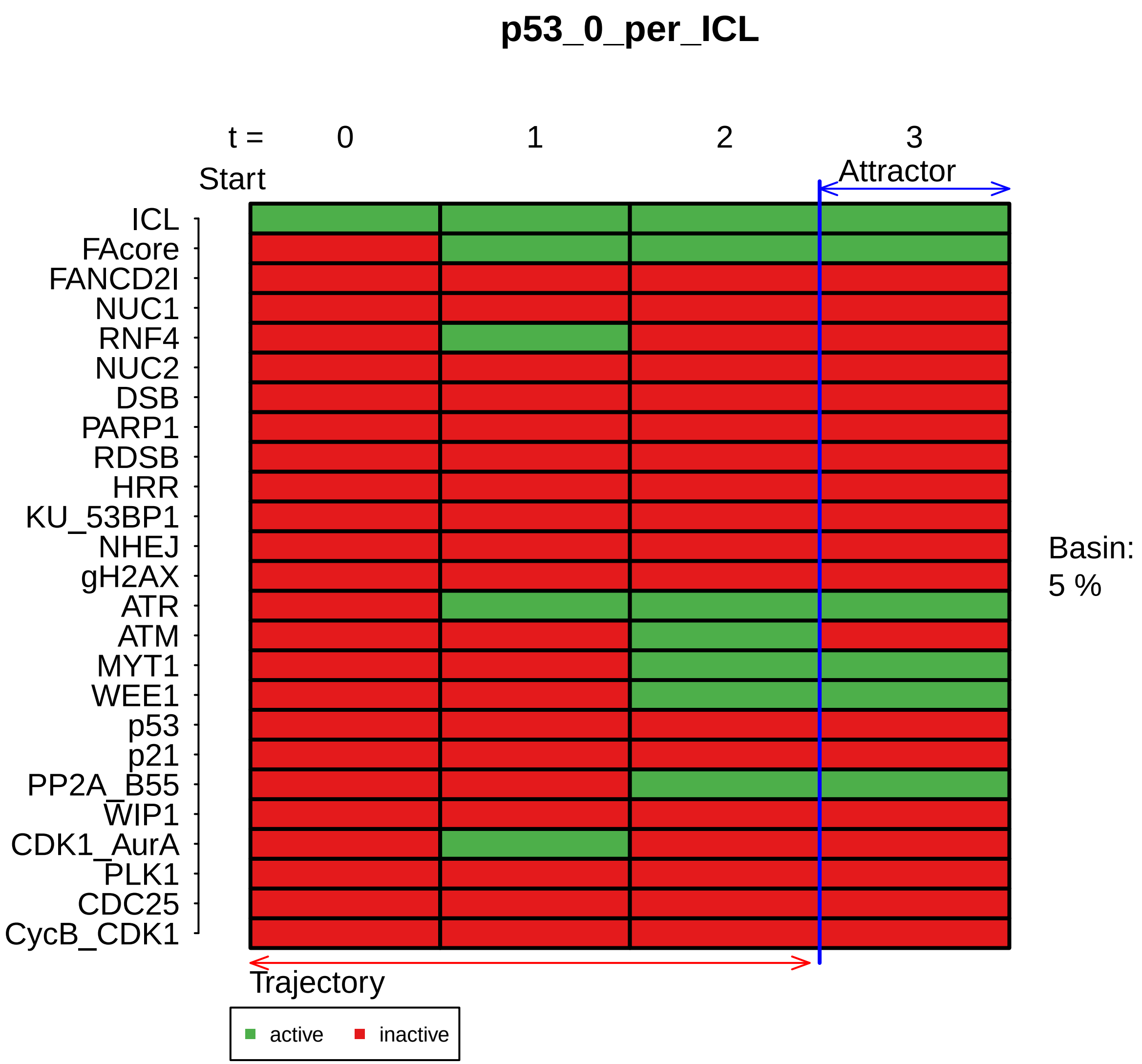
p53_0_per_ICL
t =
0
1
2
3
Start
Attractor
ICL
FAcore
FANCD2I
NUC1
RNF4
NUC2
DSB
PARP1
RDSB
HRR
KU_53BP1
NHEJ
gH2AX
ATR
ATM
MYT1
WEE1
p53
p21
PP2A_B55
WIP1
CDK1_AurA
PLK1
CDC25
CycB_CDK1
Basin:
5 %
Trajectory
active
inactive

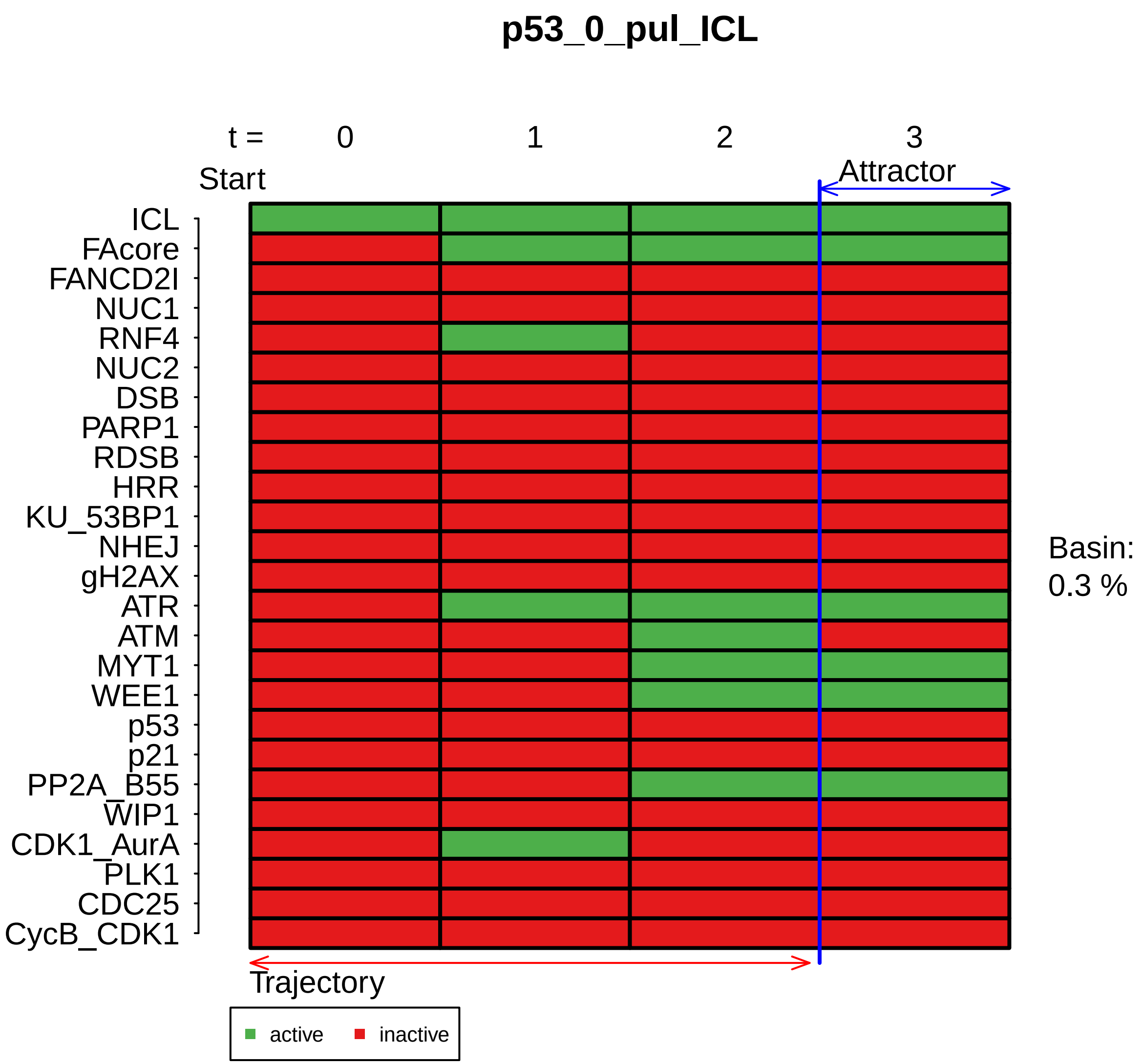
p53_0_pul_ICL
t =
0
1
2
3
Start
Attractor
ICL
FAcore
FANCD2I
NUC1
RNF4
NUC2
DSB
PARP1
RDSB
HRR
KU_53BP1
NHEJ
gH2AX
ATR
ATM
MYT1
WEE1
p53
p21
PP2A_B55
WIP1
CDK1_AurA
PLK1
CDC25
CycB_CDK1
Basin:
0.3 %
Trajectory
active
inactive

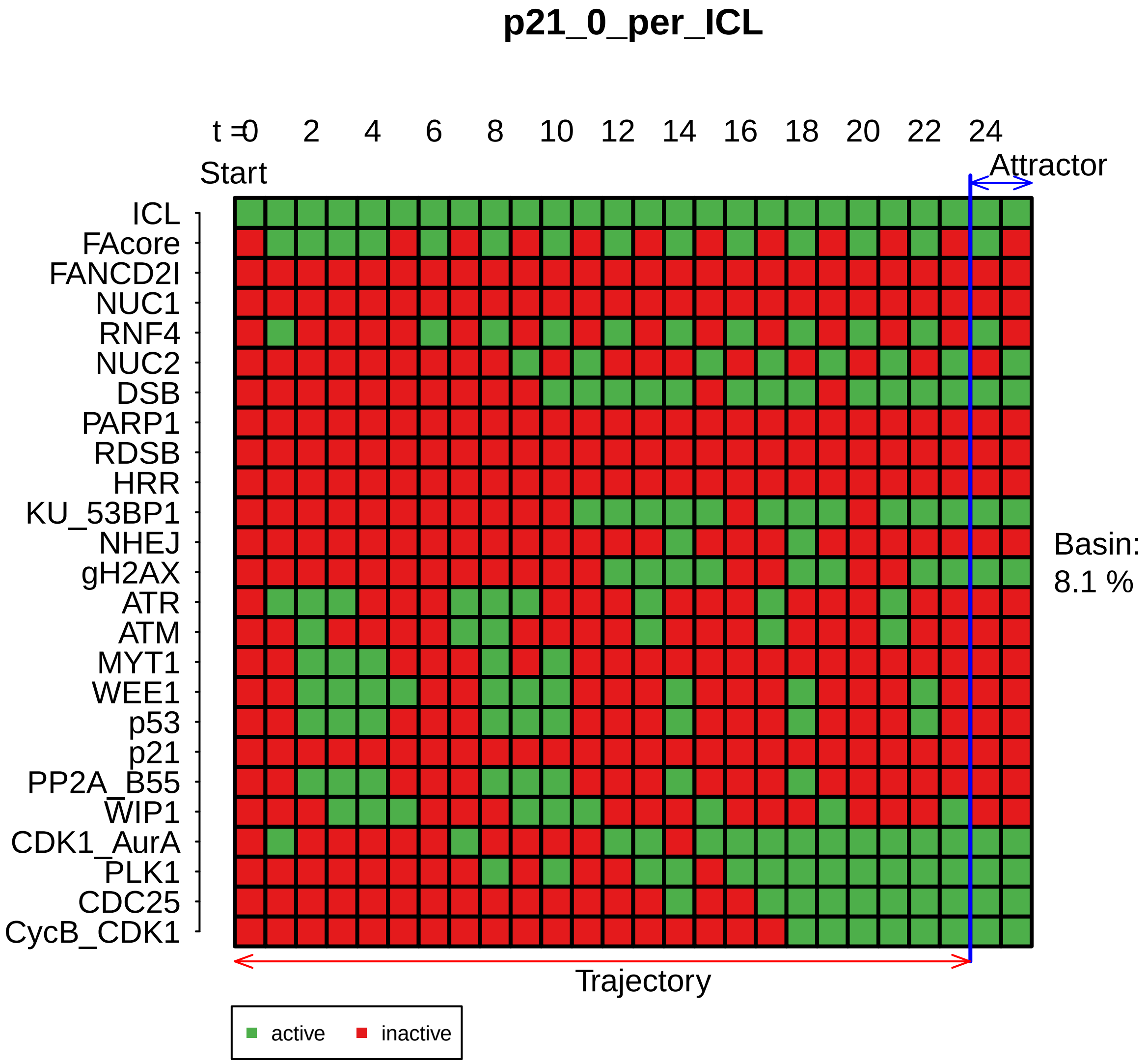
p21_0_per_ICL
t =
0
2
4
6
8
10
12
14
16
18
20
22
24
Start
Attractor
ICL
FAcore
FANCD2I
NUC1
RNF4
NUC2
DSB
PARP1
RDSB
HRR
KU_53BP1
NHEJ
gH2AX
ATR
ATM
MYT1
WEE1
p53
p21
PP2A_B55
WIP1
CDK1_AurA
PLK1
CDC25
CycB_CDK1
Basin:
8.1 %
Trajectory
active
inactive

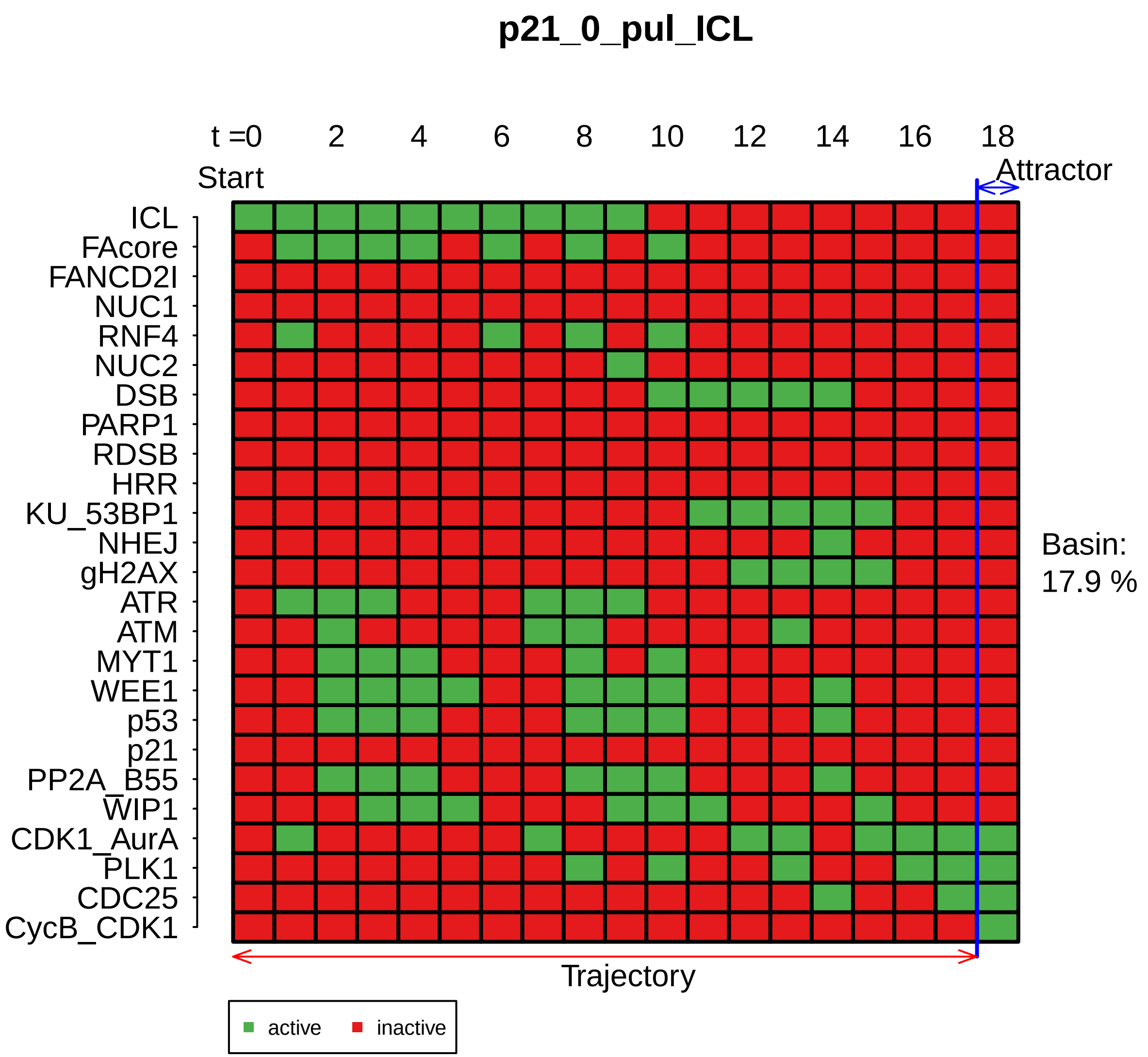

p21_0_pul_ICL
t =0
2
4
6
8
10
12
14
16
18
Start
Attractor
ICL
FAcore
FANCD2I
NUC1
RNF4
NUC2
DSB
PARP1
RDSB
HRR
KU_53BP1
NHEJ
gH2AX
ATR
ATM
MYT1
WEE1
p53
p21
PP2A_B55
WIP1
CDK1_AurA
PLK1
CDC25
CycB_CDK1
Basin:
17.9 %
Trajectory
active
inactive

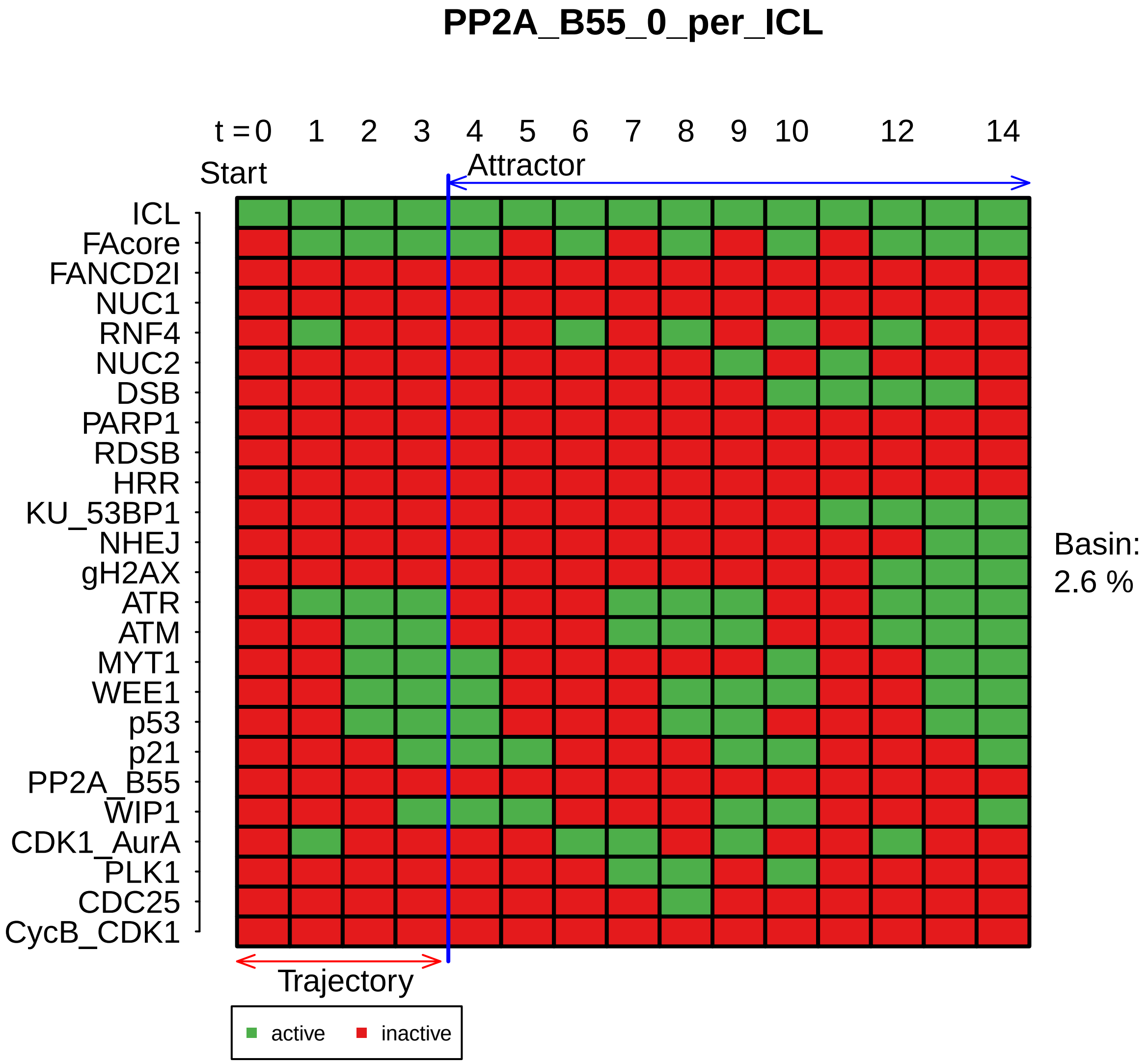
PP2A_B55_0_per_ICL
t = 0 1 2 3 4 5 6 7 8 9 10 12 14
Start
Attractor
ICL
FAcore
FANCD2I
NUC1
RNF4
NUC2
DSB
PARP1
RDSB
HRR
KU_53BP1
NHEJ
gH2AX
ATR
ATM
MYT1
WEE1
p53
p21
PP2A_B55
WIP1
CDK1_AurA
PLK1
CDC25
CycB_CDK1
Basin:
2.6 %
Trajectory
active
inactive

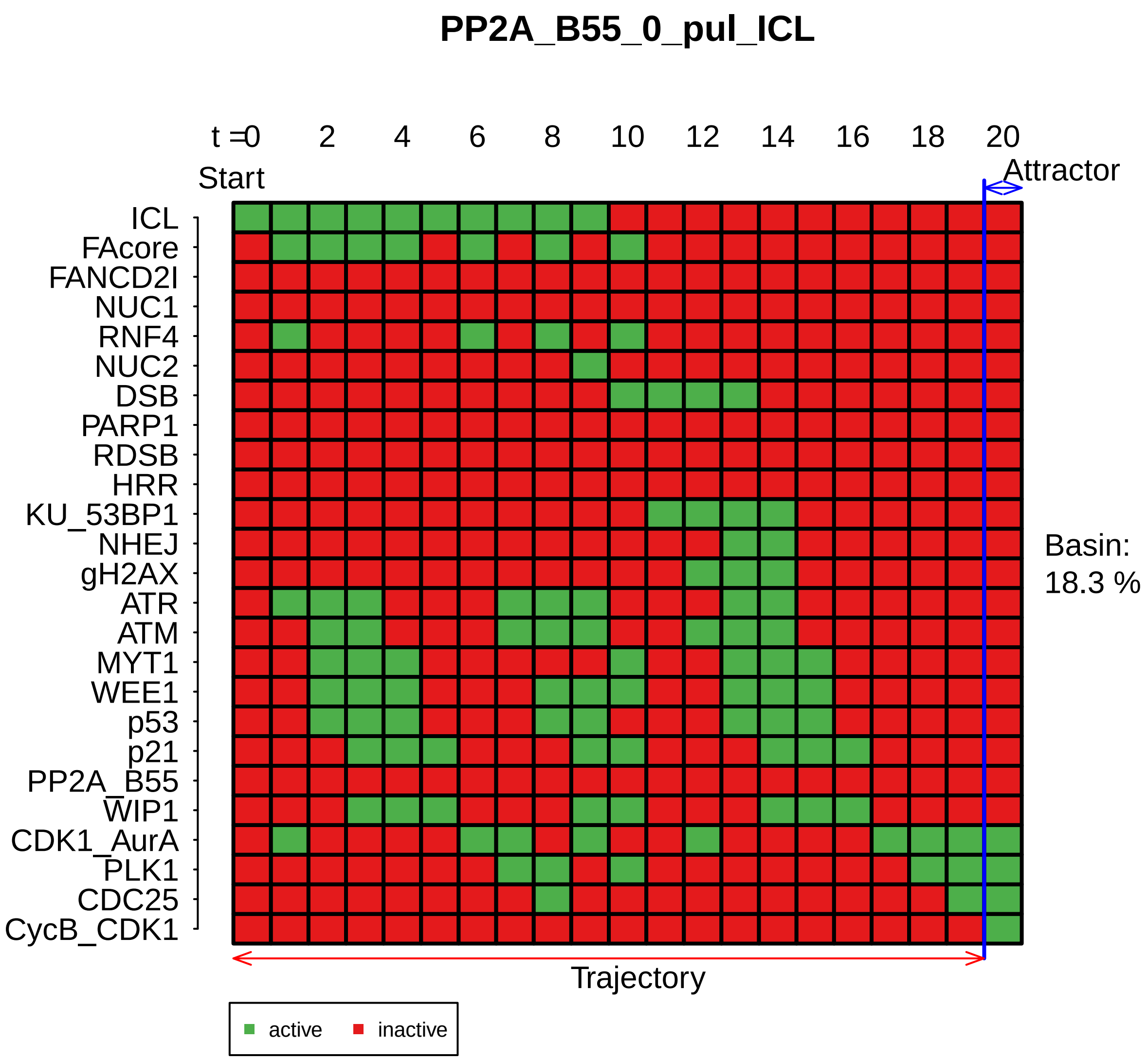

PP2A_B55_0_pul_ICL
t =0
2
4
6
8
10
12
14
16
18
20
Start
Attractor
ICL
FAcore
FANCD2I
NUC1
RNF4
NUC2
DSB
PARP1
RDSB
HRR
KU_53BP1
NHEJ
gH2AX
ATR
ATM
MYT1
WEE1
p53
p21
PP2A_B55
WIP1
CDK1_AurA
PLK1
CDC25
CycB_CDK1
Basin:
18.3 %
Trajectory
active
inactive

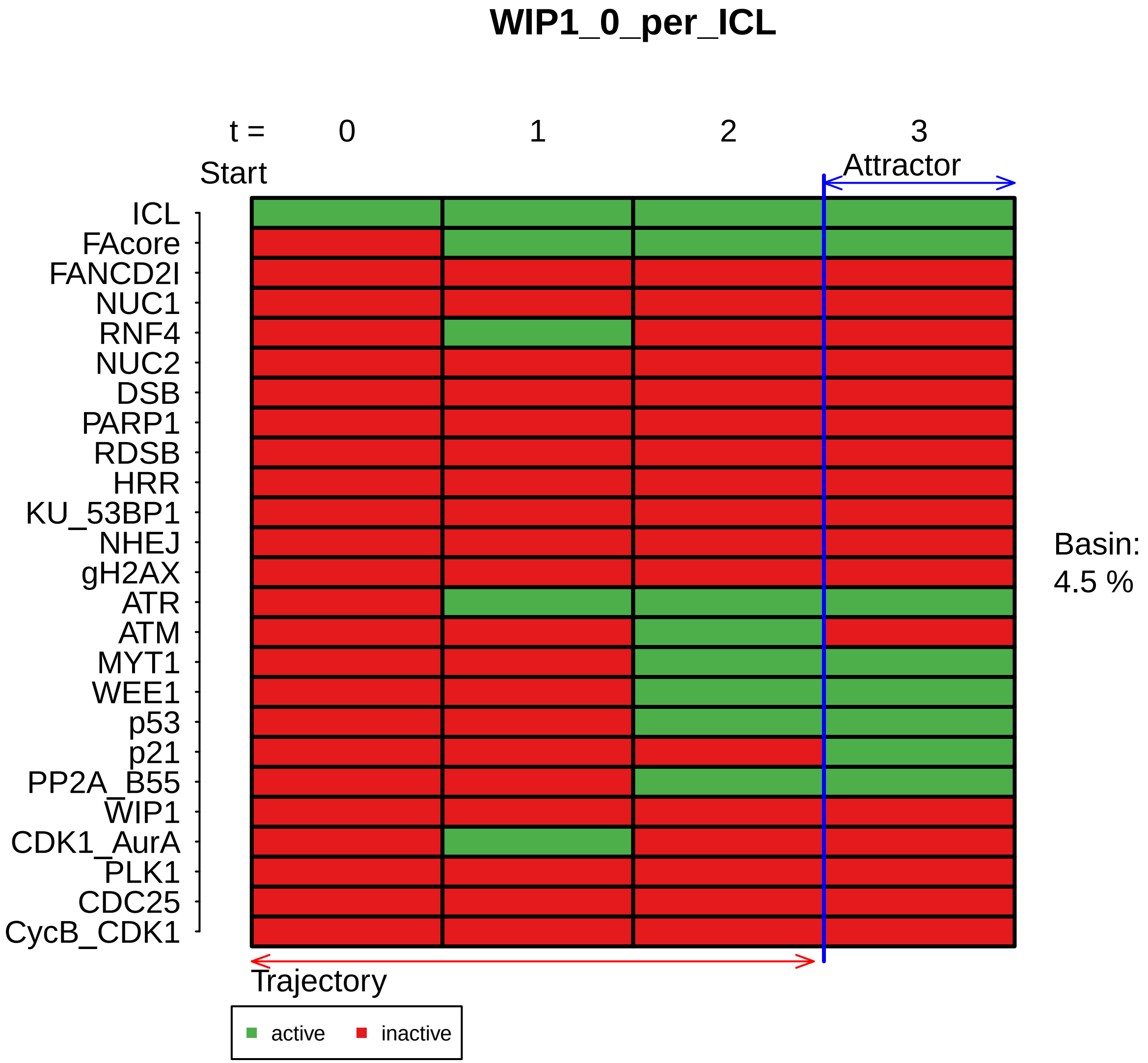
WIP1_0_per_ICL
t =
0
1
2
3
Start
Attractor
ICL
FAcore
FANCD2I
NUC1
RNF4
NUC2
DSB
PARP1
RDSB
HRR
KU_53BP1
NHEJ
gH2AX
ATR
ATM
MYT1
WEE1
p53
p21
PP2A_B55
WIP1
CDK1_AurA
PLK1
CDC25
CycB_CDK1
Basin:
4.5 %
Trajectory
active
inactive

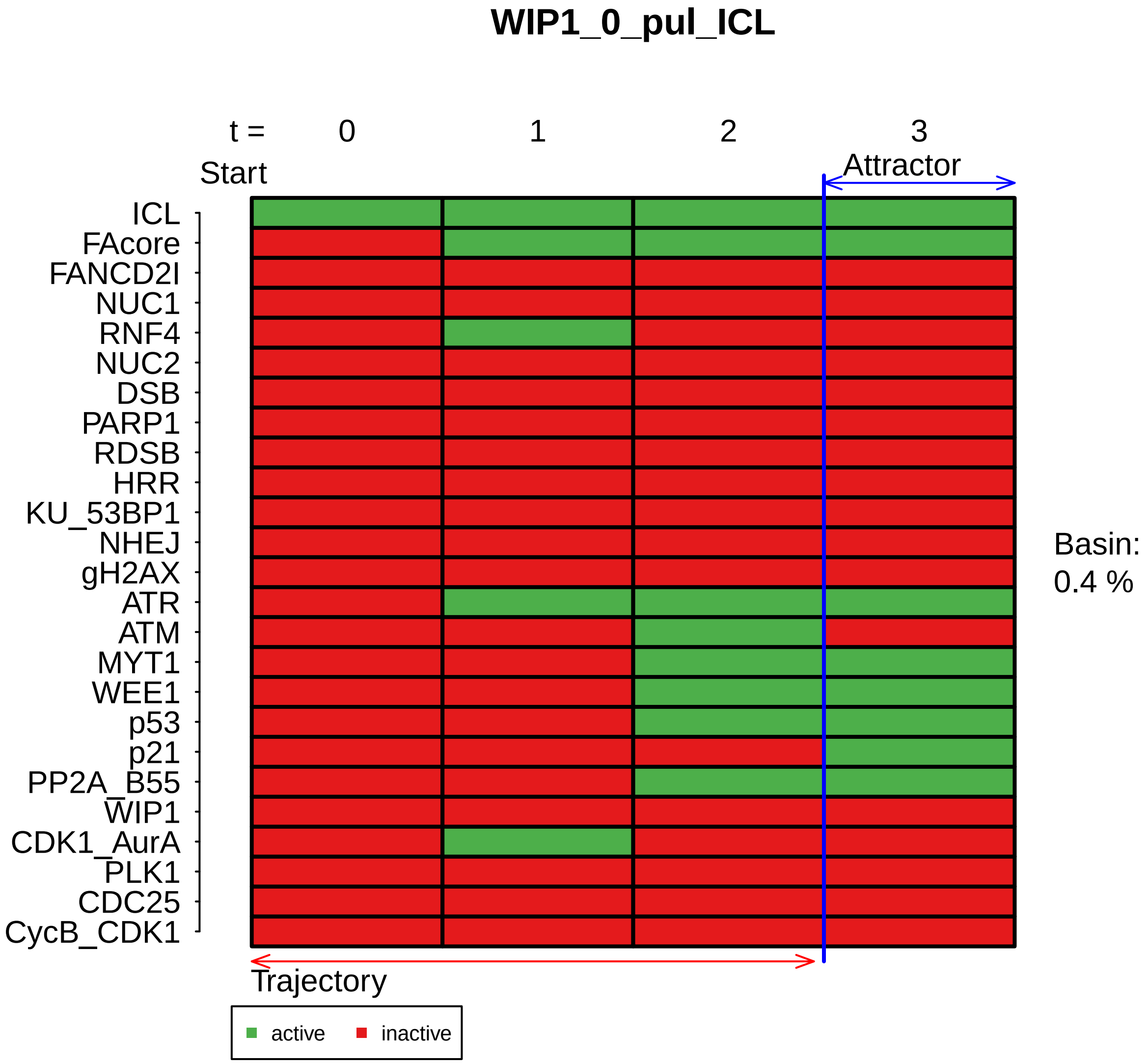
WIP1_0_pul_ICL
t =
0
1
2
3
Start
Attractor
ICL
FAcore
FANCD2I
NUC1
RNF4
NUC2
DSB
PARP1
RDSB
HRR
KU_53BP1
NHEJ
gH2AX
ATR
ATM
MYT1
WEE1
p53
p21
PP2A_B55
WIP1
CDK1_AurA
PLK1
CDC25
CycB_CDK1
Basin:
0.4 %
Trajectory
active
inactive

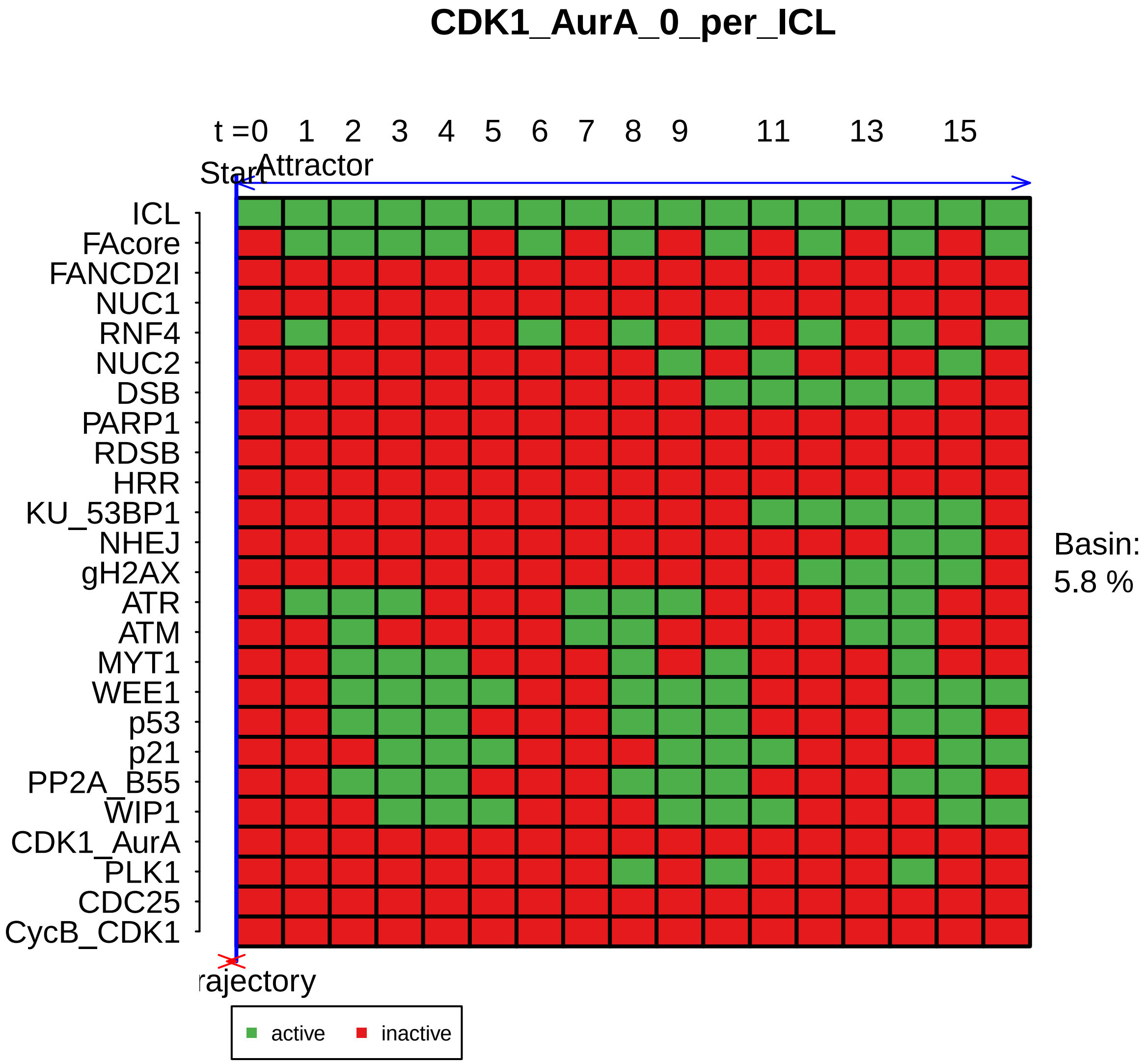
CDK1_AurA_0_per_ICL
t =0 1 2 3 4 5 6 7 8 9 11 13 15
Start
Attractor
ICL
FAcore
FANCD2I
NUC1
RNF4
NUC2
DSB
PARP1
RDSB
HRR
KU_53BP1
NHEJ
gH2AX
ATR
ATM
MYT1
WEE1
p53
p21
PP2A_B55
WIP1
CDK1_AurA
PLK1
CDC25
CycB_CDK1
Basin:
5.8 %
rajectory
active
inactive

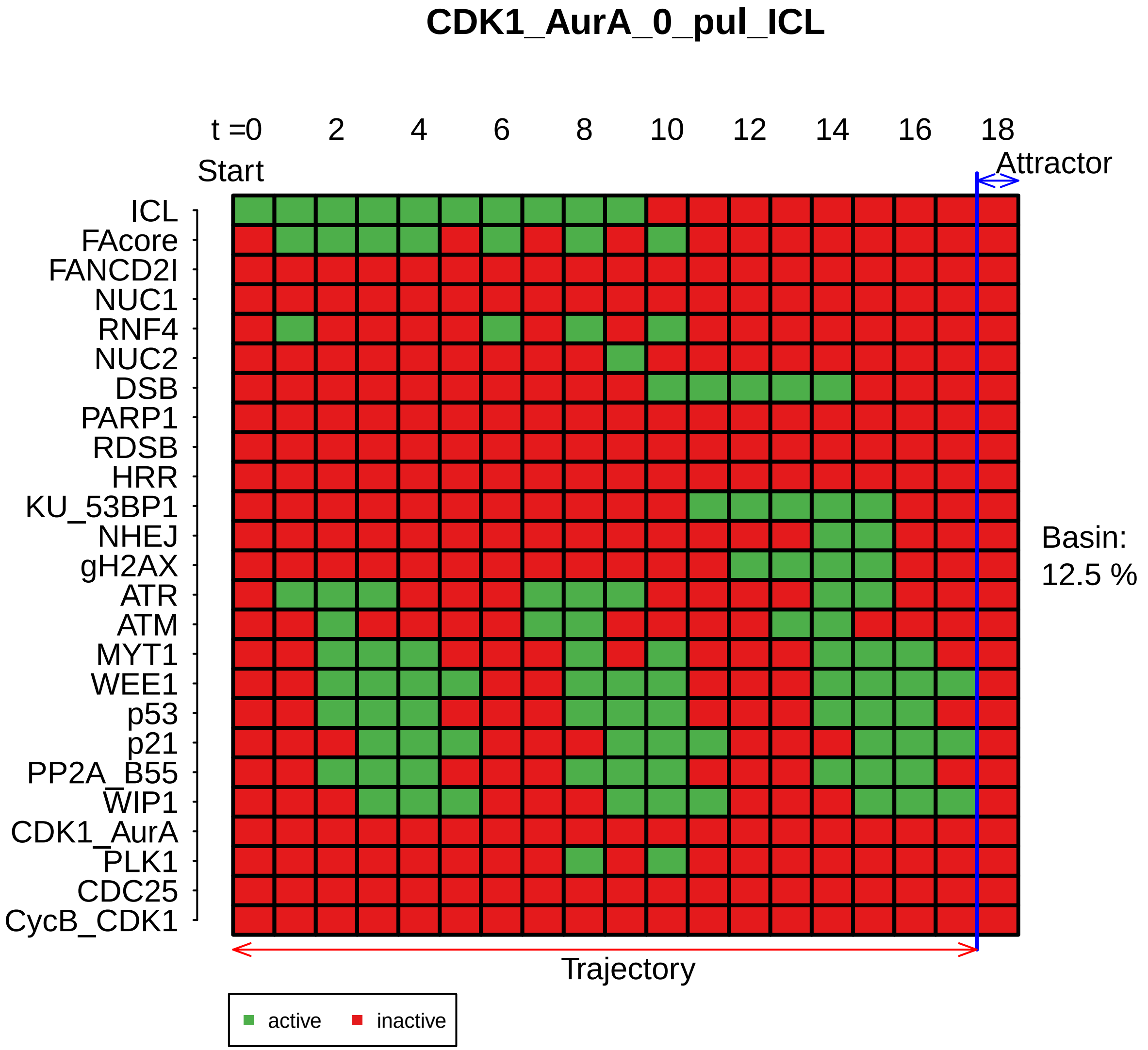
CDK1_AurA_0_pul_ICL
t =0
2
4
6
8
10
12
14
16
18
Start
Attractor
ICL
FAcore
FANCD2I
NUC1
RNF4
NUC2
DSB
PARP1
RDSB
HRR
KU_53BP1
NHEJ
gH2AX
ATR
ATM
MYT1
WEE1
p53
p21
PP2A_B55
WIP1
CDK1_AurA
PLK1
CDC25
CycB_CDK1
Basin:
12.5 %
Trajectory
active
inactive

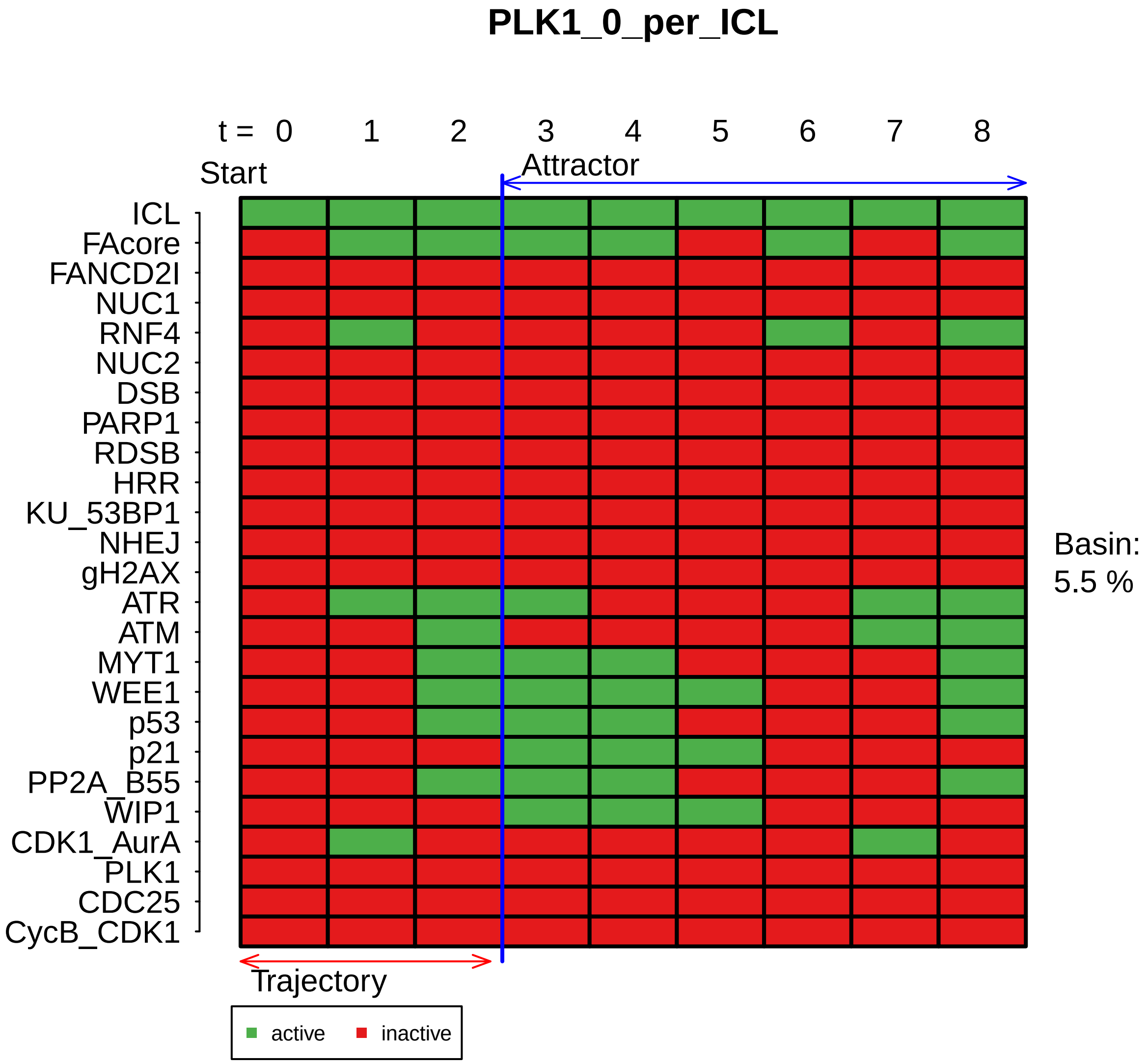
PLK1_0_per_ICL
t = 0 1 2 3 4 5 6 7 8
Start
Attractor
ICL
FAcore
FANCD2I
NUC1
RNF4
NUC2
DSB
PARP1
RDSB
HRR
KU_53BP1
NHEJ
gH2AX
ATR
ATM
MYT1
WEE1
p53
p21
PP2A_B55
WIP1
CDK1_AurA
PLK1
CDC25
CycB_CDK1
Basin:
5.5 %
Trajectory
active
inactive

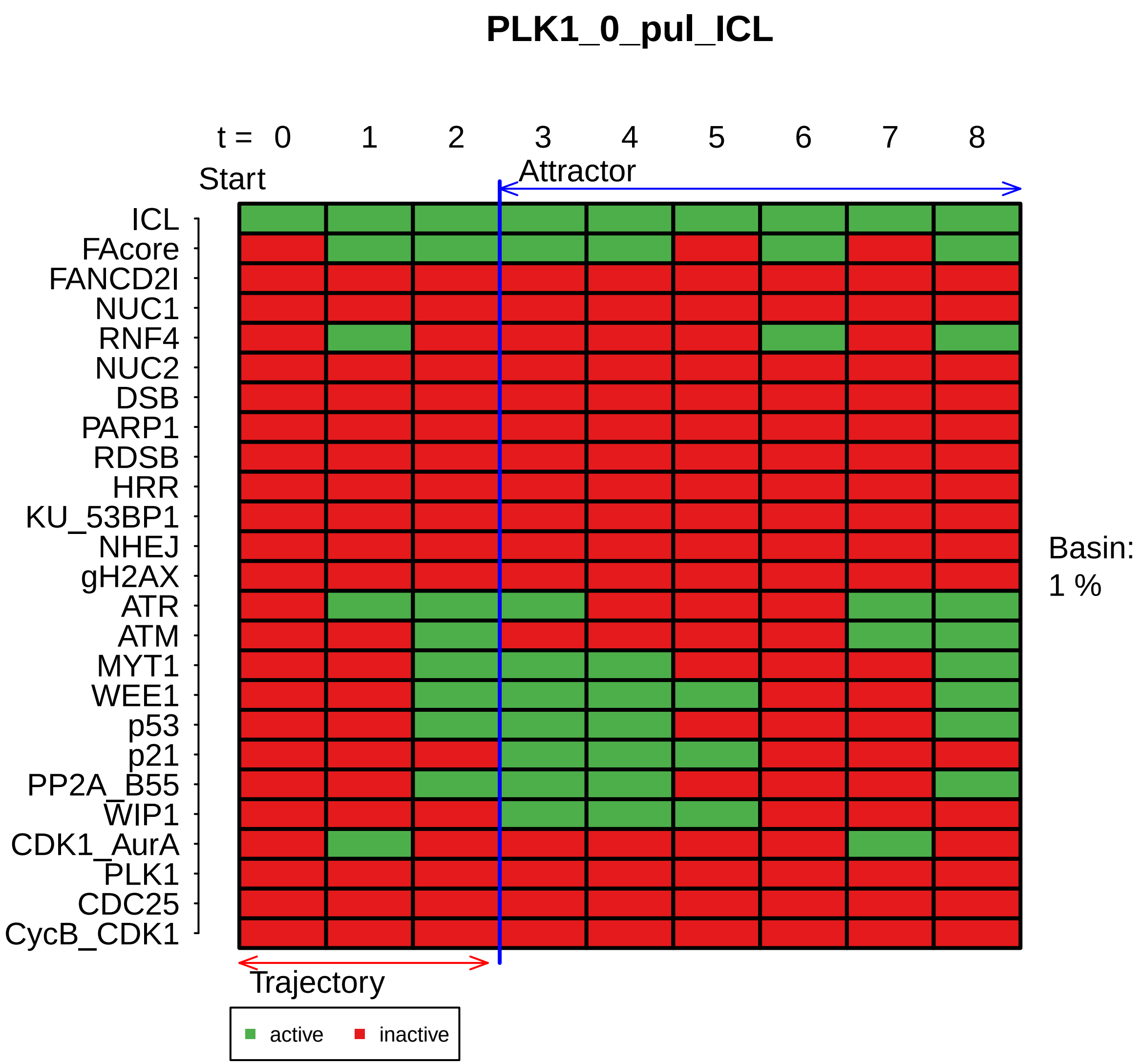
PLK1_0_pul_ICL
t = 0 1 2 3 4 5 6 7 8
Start
Attractor
ICL
FAcore
FANCD2I
NUC1
RNF4
NUC2
DSB
PARP1
RDSB
HRR
KU_53BP1
NHEJ
gH2AX
ATR
ATM
MYT1
WEE1
p53
p21
PP2A_B55
WIP1
CDK1_AurA
PLK1
CDC25
CycB_CDK1
Basin:
1 %
Trajectory
active
inactive

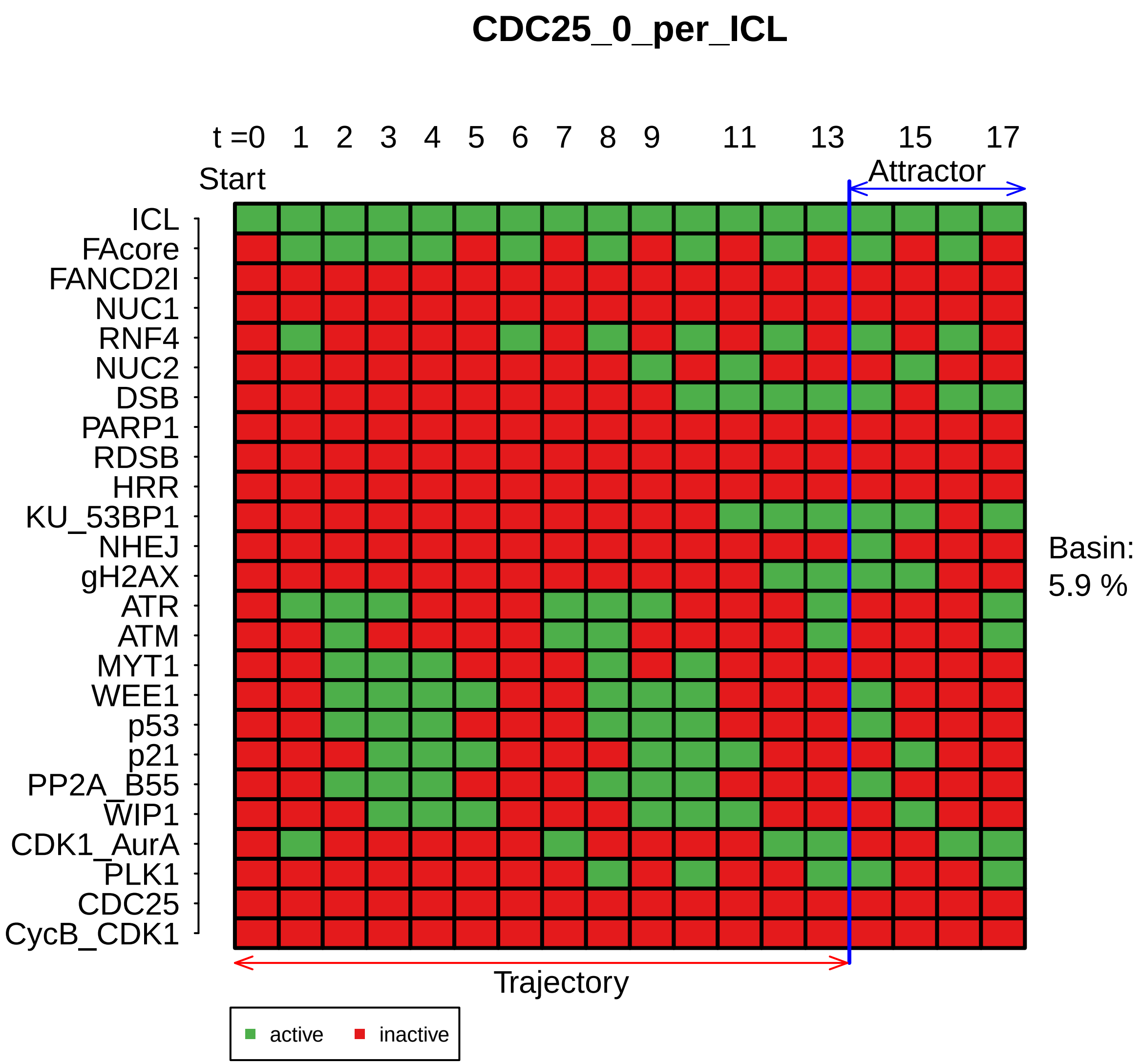
CDC25_0_per_ICL
t =0 1 2 3 4 5 6 7 8 9 11 13 15 17
Start
Attractor
ICL
FAcore
FANCD2I
NUC1
RNF4
NUC2
DSB
PARP1
RDSB
HRR
KU_53BP1
NHEJ
gH2AX
ATR
ATM
MYT1
WEE1
p53
p21
PP2A_B55
WIP1
CDK1_AurA
PLK1
CDC25
CycB_CDK1
Basin:
5.9 %
Trajectory
active
inactive

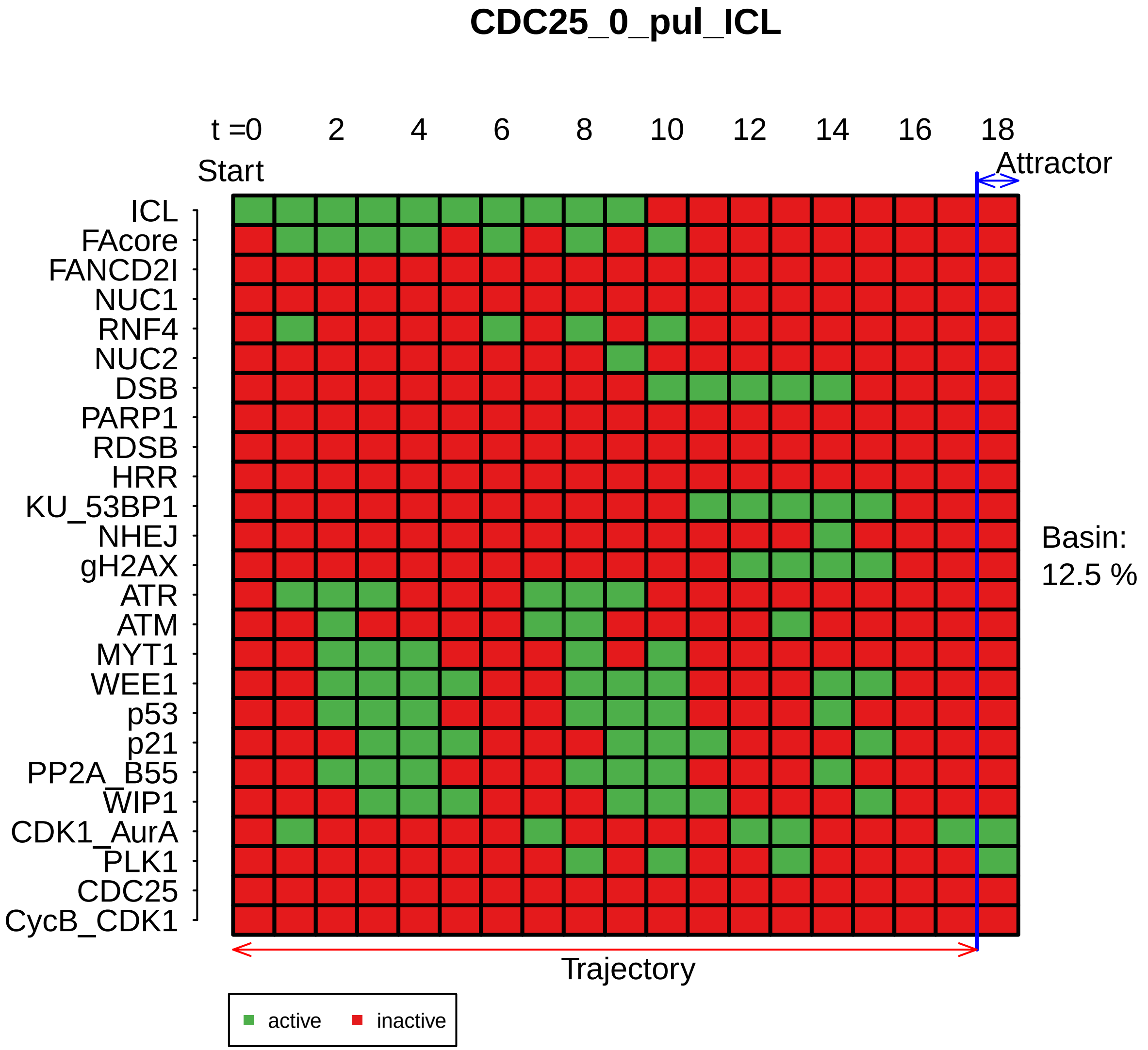
CDC25_0_pul_ICL
t =0
2
4
6
8
10
12
14
16
18
Start
Attractor
ICL
FAcore
FANCD2I
NUC1
RNF4
NUC2
DSB
PARP1
RDSB
HRR
KU_53BP1
NHEJ
gH2AX
ATR
ATM
MYT1
WEE1
p53
p21
PP2A_B55
WIP1
CDK1_AurA
PLK1
CDC25
CycB_CDK1
Basin:
12.5 %
Trajectory
active
inactive

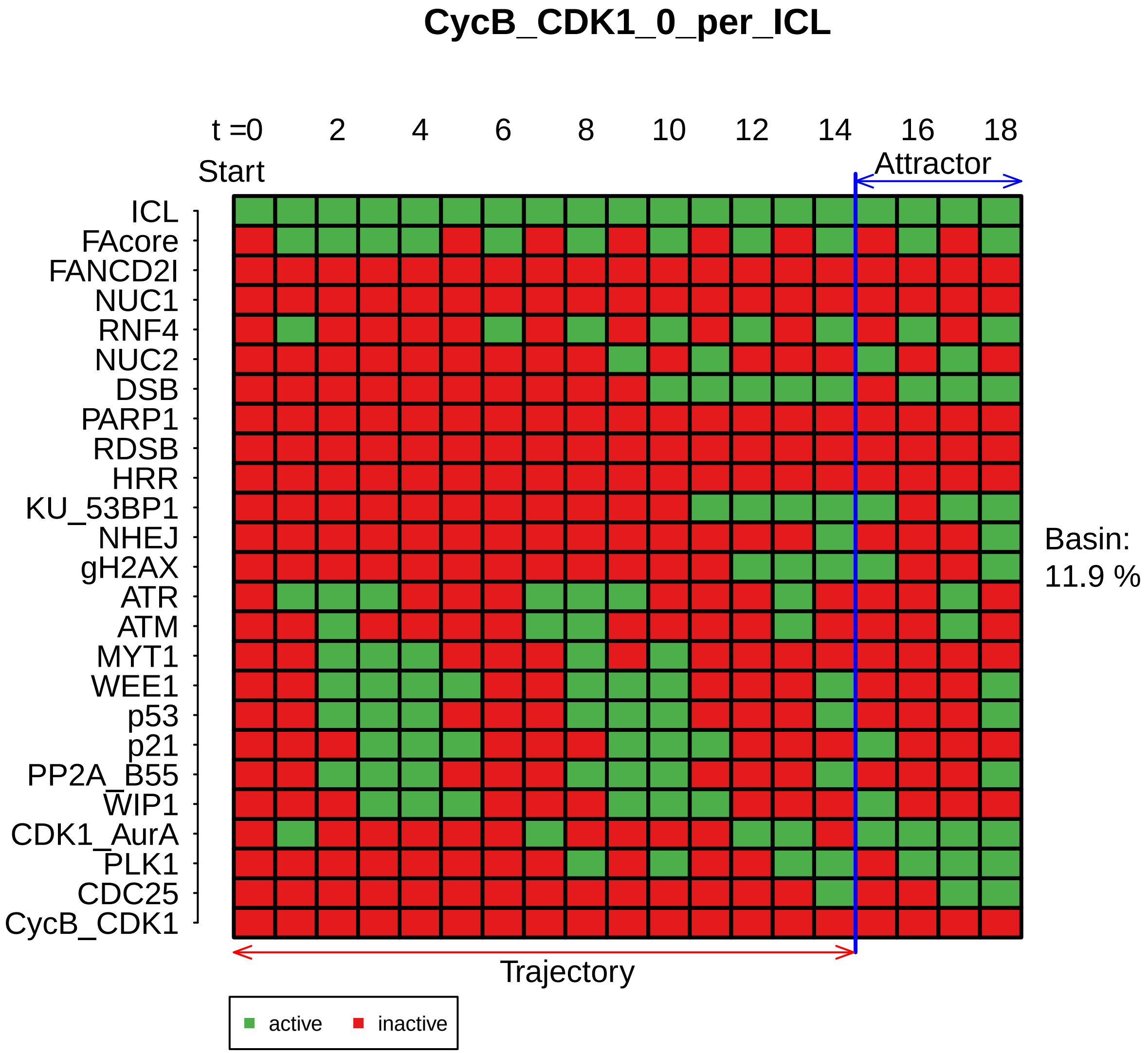

CycB_CDK1_0_per_ICL
t =0
2
4
6
8
10
12
14
16
18
Start
Attractor
ICL
FAcore
FANCD2I
NUC1
RNF4
NUC2
DSB
PARP1
RDSB
HRR
KU_53BP1
NHEJ
gH2AX
ATR
ATM
MYT1
WEE1
p53
p21
PP2A_B55
WIP1
CDK1_AurA
PLK1
CDC25
CycB_CDK1
Basin:
11.9 %
Trajectory
active
inactive

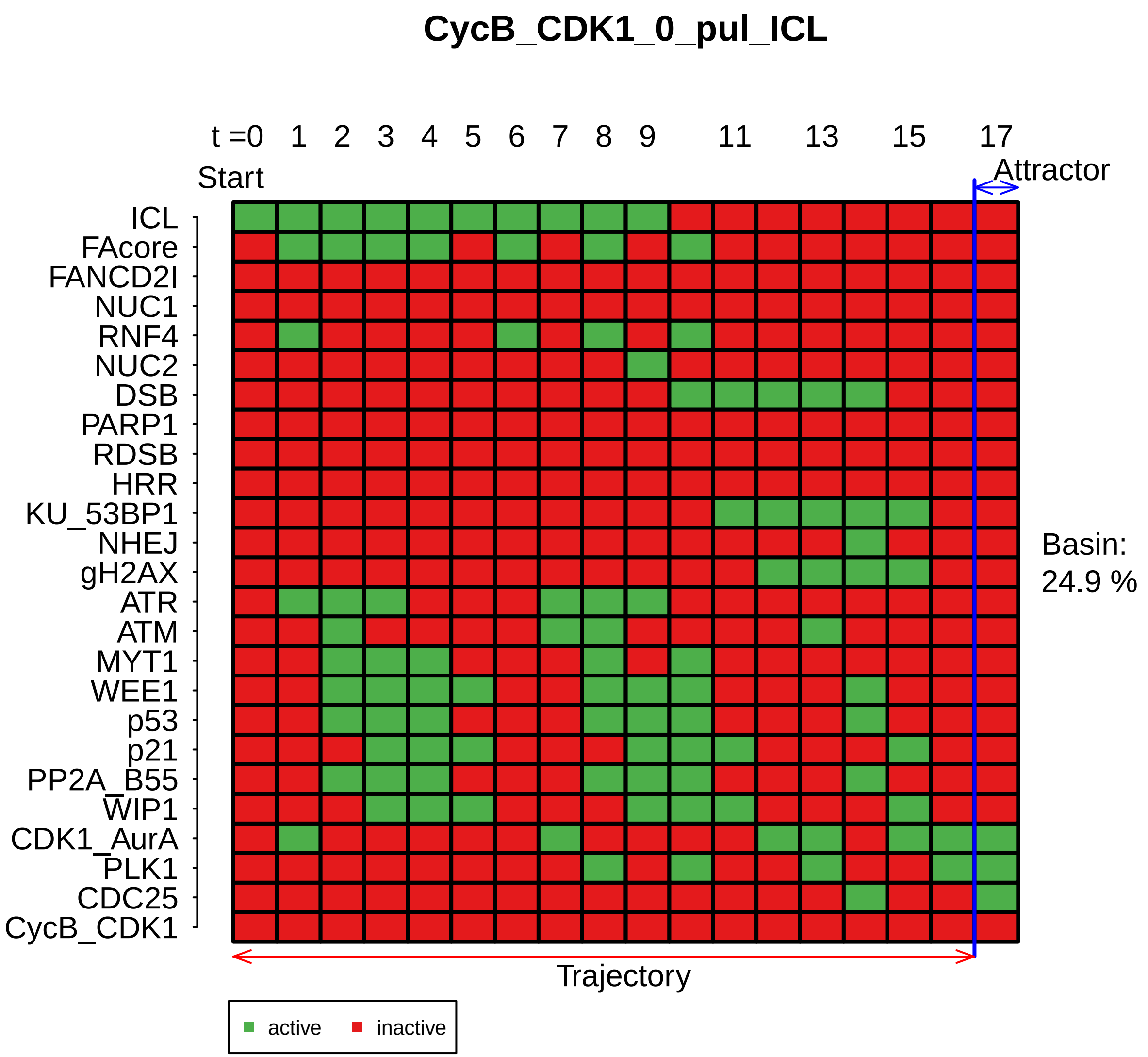
CycB_CDK1_0_pul_ICL
t =0
1
2
3
4
5
6
7
8
9
11
13
15
17
Start
Attractor
ICL
FAcore
FANCD2I
NUC1
RNF4
NUC2
DSB
PARP1
RDSB
HRR
KU_53BP1
NHEJ
gH2AX
ATR
ATM
MYT1
WEE1
p53
p21
PP2A_B55
WIP1
CDK1_AurA
PLK1
CDC25
CycB_CDK1
Basin:
24.9 %
Trajectory
active
inactive
