# Supplementary material for: WIP1 Contributes to the Adaptation of Fanconi Anemia Cells to DNA Damage as Determined by the Regulatory Network of the Fanconi Anemia and Checkpoint Recovery Pathways
Source: Front Genet. 2019 May 3;10:411. doi: 10.3389/fgene.2019.00411 (PMC6509935; doi:10.3389/fgene.2019.00411)
Supplement: Supplementary file 5 [file Data_Sheet_5.pdf]

```
targets, factors

ICL, ICL & ! (NUC1 | NUC2)

FAcore, ICL & ! (RNF4 & Plk1 | (FAcore & ! ATR))

FANCD2I, FAcore & (ATM | ATR) & ! FANCD2I

NUC1, (ICL & FANCD2I) | (DSB & PARP1)

RNF4, ICL & ! FAcore

NUC2, ICL & RNF4 & Plk1 & ! (RDSB | NUC1)

DSB, (DSB | (ICL & NUC2)) & ! (NHEJ | NUC1)

PARP1, (RDSB | DSB) & gH2AX & ! KU/53BP1

RDSB, (RDSB | ((ICL | DSB) & NUC1)) & ! HRR

HRR, RDSB & gH2AX & ATM & ! (Plk1 & CycB/CDK1)

KU/53BP1, DSB & ! PARP1

NHEJ, KU/53BP1 & DSB & ATM & ! (Plk1 & CycB/CDK1)

gH2AX, (DSB | RDSB) & (ATM | ATR | gH2AX | KU/53BP1) & ! (Wip1 & PP2A/
B55)

ATR, (ICL | ATM) & ! (Wip1 | (Plk1 & KU/53BP1))

ATM, (ATR | DSB | RDSB | NUC1 | FAcore) & ! (Wip1 | PP2A/B55 | (Plk1 &
KU/53BP1))

MYT1, (ATM | ATR) & ! (CDC25 | CycB/CDK1 | Plk1)

WEE1, (ATM | ATR | PP2A/B55 & ! (CDC25 | CycB/CDK1 | Plk1))

p53, (ATM | ATR) & ! (Wip1 & (Plk1 | CDK1/AurA))

p21, p53

PP2A/B55, (ATM | ATR) & ! CycB/CDK1

Wip1, p53

CDK1/AurA, CycB/CDK1 | CDC25 | ! (p21 & PP2A/B55) & ! (WEE1 | MYT1 |
ATM | ATR)

Plk1, CycB/CDK1 | (ICL & ATR & ! FAcore) | ((CDK1/AurA ) & ! (MYT1 |
```

```
WEE1 | ATR | ATM))

CDC25, CycB/CDK1 | (Plk1 & (CycB/CDK1 | CDK1/AurA) & ! ((WEE1 | MYT1)
& (PP2A/B55 | ATM | ATR)))

CycB/CDK1, CycB/CDK1 | (CDC25 & Plk1 & CDK1/AurA) & ! p21
```

CAPTION: Boolean functions for the nodes in the FA-CHKREC network as used in BoolNet.
